# Optical control of endogenous receptors and cellular excitability using targeted covalent photoswitches

**DOI:** 10.1038/ncomms12221

**Published:** 2016-07-20

**Authors:** Mercè Izquierdo-Serra, Antoni Bautista-Barrufet, Ana Trapero, Aida Garrido-Charles, Ariadna Díaz-Tahoces, Nuria Camarero, Silvia Pittolo, Sergio Valbuena, Ariadna Pérez-Jiménez, Marina Gay, Alejandro García-Moll, Carles Rodríguez-Escrich, Juan Lerma, Pedro de la Villa, Eduardo Fernández, Miquel À Pericàs, Amadeu Llebaria, Pau Gorostiza

**Affiliations:** 1 Institut de Bioenginyeria de Catalunya (IBEC), Barcelona 08028, Spain; 2 Institute of Chemical Research of Catalonia (ICIQ), The Barcelona Institute of Science and Technology, Tarragona 43007, Spain; 3 Institut de Química Avançada de Catalunya (IQAC-CSIC), Barcelona 08034, Spain; 4 Instituto de Bioingeniería, Universidad Miguel Hernández (UMH), Elche 03202, Spain; 5 Instituto de Neurociencias (CSIC-UMH), San Juan de Alicante 03550, Spain; 6 Institut de Recerca en Biomedicina (IRBB), Barcelona 08028, Spain; 7 Universidad de Alcalá de Henares (UAH), Alcalá de Henares 28871, Spain; 8 Centro de Investigación Biomédica en Red en Bioingeniería, Biomateriales y Nanomedicina (CIBER-BBN), Zaragoza 50018, Spain; 9 Departament de Química Inorgànica i Orgànica, Universitat de Barcelona (UB), Barcelona 08007, Spain; 10 Institució Catalana de Recerca i Estudis Avançats (ICREA), Barcelona 08010, Spain

## Abstract

Light-regulated drugs allow remotely photoswitching biological activity and enable plausible therapies based on small molecules. However, only freely diffusible photochromic ligands have been shown to work directly in endogenous receptors and methods for covalent attachment depend on genetic manipulation. Here we introduce a chemical strategy to covalently conjugate and photoswitch the activity of endogenous proteins and demonstrate its application to the kainate receptor channel GluK1. The approach is based on photoswitchable ligands containing a short-lived, highly reactive anchoring group that is targeted at the protein of interest by ligand affinity. These targeted covalent photoswitches (TCPs) constitute a new class of light-regulated drugs and act as prosthetic molecules that photocontrol the activity of GluK1-expressing neurons, and restore photoresponses in degenerated retina. The modularity of TCPs enables the application to different ligands and opens the way to new therapeutic opportunities.

## Abstract (web summary)

Biological activity can be photoswitched by light-regulated drugs, but so far only diffusible ligands have been shown to work on endogenous receptors. Here the authors develop targeted covalent photoswitches that couple to a protein target by ligand affinity, and demonstrate photocontrol of GluK1-expressing neurons.

## Graphical abstract

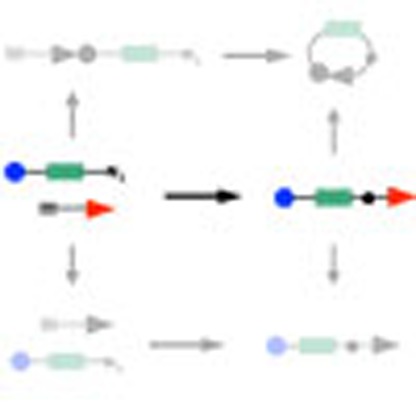

The manipulation of neuronal activity with light by means of optogenetics^1^ and photopharmacology^2^ has revolutionized experimental neurobiology and is causing a great impact on physiology. These scientific breakthroughs opened the way for therapeutic applications including vision restoration in degenerated retina^3^,^4^,^5^ and regulating pathological processes with light, such as the movement of paralysed muscles^6^ or the secretion of insulin^7^. Manipulation of neuronal activity by optogenetics is based on the expression of naturally light-sensitive proteins, whereas photopharmacology relies in the use of synthetic light-regulated bioactive ligands. In particular, photochromic ligands (PCLs) are freely diffusible small molecules that act directly on endogenous proteins and bear strong potential to be developed, validated and used as therapeutic or research drugs. However, PCLs often display low specificity for a given molecular target, photoswitching is limited to a narrow concentration range and dilution in tissue reduces their efficacy and causes off-target effects. To avoid these drawbacks of diffusible ligands, photocontrol can be confined to designated receptors and cells by means of photoisomerizable tethered ligands (PTLs) that are chemically attached to genetically engineered receptor proteins. This confinement comes at the cost of genetic manipulation, which poses other limitations: expression of membrane receptors can be low, slow, non-uniform or not available in some organisms. In addition, overexpression of exogenous proteins can hijack cellular expression machinery and disturb normal physiology, especially in protein-dense neuronal compartments, or even cause immune responses^8^.

We describe here a new strategy to photoswitch protein activity that has the advantages of covalent attachment to the target but can be applied to endogenous proteins without requiring genetic manipulation. The approach is based on a PTL containing a short-lived highly reactive anchoring group that, in analogy to the mechanism of targeted covalent drugs^9^, is driven to the protein of interest by its binding affinity. Our targeted covalent photoswitches (TCPs) afford kinetically controlled site-selective conjugation to lysine residues exposed on the protein surface in the vicinity of the ligand-binding site. TCP design and optimization is demonstrated in a glutamate receptor agonist and their modularity enables the application to other ligand-binding proteins. Illumination of the photoisomerizable tether at different wavelengths allows controlling the activation of GluK1 receptor and membrane depolarization. In this way, TCPs enable photocontrolling the activity of neurons that endogenously express GluK1 and restore robust and sustained photoresponses in degenerated retina without genetic manipulation.

## Results

### Design and synthetic approach to widely reactive PTLs

**Figure 1:**
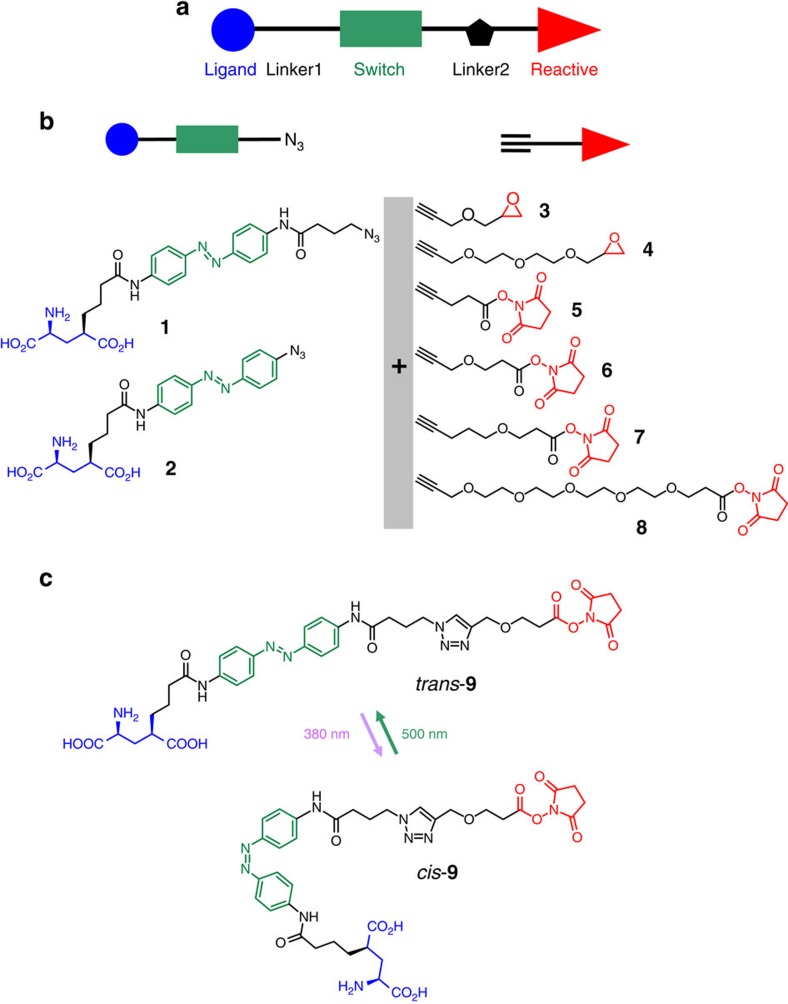
Architecture and synthetic design of photoswitchable tethered ligands (PTLs) to control ligand-gated receptor proteins. (**a**) Schematic representation of the PTL modular parts: ligand (blue), photoswitch (green) and reactive group (red). (**b**,**c**) Design of PTLs bearing electrophilic and nucleophilic groups to label endogenous kainate receptors (**b**) The ‘head' (glutamate-azo-azide, left) and ‘tail' molecules (alkyne-electrophile, right) are precursors of copper(I)-catalysed alkyne-azide cycloaddition and can be combined to yield PTLs of different lengths and reactive groups. (**c**) Example of full PTL (compound **9**) obtained from precursors **1** and **6**, and showing the photoisomerization between the *trans* (green light, *λ*=500 nm) and *cis* configurations (violet light, *λ*=380 nm).

**Figure 2:**
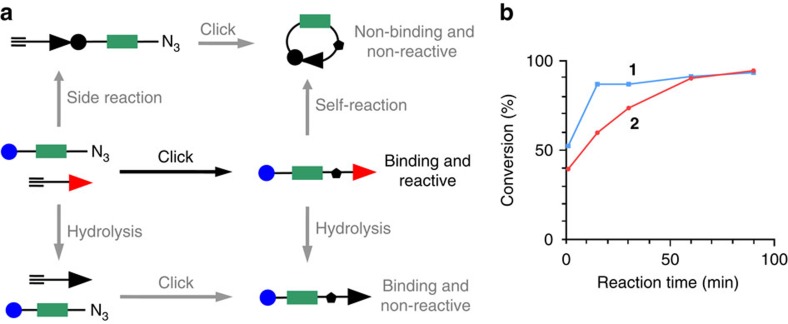
PTLs bearing electrophilic and nucleophilic groups are coupled by a click reaction that is faster than all competing reactions. (**a**) The head and tail PTL precursors (middle row, left) can lead to the desired product (middle row, right; reaction indicated by a black arrow) and to other products through several competing reactions (side reaction, self-reaction and hydrolysis; marked with grey arrows). The required product is a PTL bearing simultaneously a ligand moiety capable of binding to its receptor and a reactive group to covalently conjugate to nucleophilic amino acid side chains on the protein. (**b**) Percentage of azide conversion versus reaction time under the click coupling conditions with head compounds **1** and **2**, and 4-pentynoic acid as a tail model (see Supplementary Fig. 2 for optimization of the click conditions and Supplementary Figs 5-8 for characterization of full PTLs **9**, **10** and side-product identification).

PTLs bear a photoisomerizable group flanked by a pharmacological ligand and a reactive group used to covalently conjugate the PTL to the target protein (Fig. 1a). In general, azobenzene is the switch of choice due to its photophysical properties and synthetic accessibility of its derivatives^10^. The ligands can be neurotransmitters (Supplementary Fig. 1a) or other agonists, antagonists or modulators^2^. Mild electrophiles such as maleimide and halide acetamides have been convenient reactive groups due to their stability in aqueous solution and selectivity for cysteine residues. However, as reduced and solvent-exposed cysteine residues are relatively rare in proteins, in practice PTL conjugation requires mutating to cysteine a residue near the ligand-binding site and overexpressing the mutant protein. To fully exploit the advantages of optopharmacology in PTLs, the chemical promiscuity of the reactive group can be enhanced to make the PTL conjugate to wild-type proteins. A strong electrophilic moiety in the PTL would be coupled to reactive amines and hydroxyl groups, which are present in several amino acid side chains (Supplementary Fig. 1b). However, these groups are also present in many ligands including neurotransmitters (Supplementary Fig. 1a), and thus the synthesis and chemical stability of PTL compounds bearing simultaneously amine and amine-reactive moieties are compromised by their strong tendency to self-reactivity. To crack this problem, we devised a strategy to rapidly generate a PTL that is stable enough to react with the protein. We applied it to obtain a kainate receptor PTL^11^ from two separate precursor compounds that safely bear the two chemically incompatible moieties (ligand and reactive groups) (Fig. 1b). We designate them by ‘head' (including the ligand, a linker and the photoisomerizable group, compounds **1** and **2**) and ‘tail' (including a second linker and the reactive group, compounds **3** to **8**). We built a library of several precursors to exploit the versatility provided by the new synthetic approach and included molecules of different linker lengths and reactive groups (epoxide and *N*-hydroxysuccinimide esters, NHS). The synthesis and characterization of the precursors are described in Supplementary Note 1 and Supplementary Figs 24−42. Heads and tails were combined to form the final PTL using a fast version of the azide-alkyne Huisgen cycloaddition (copper(I)-catalysed alkyne-azide cycloaddition ‘click chemistry')^12^. Click chemistry offers three fundamental advantages as follows: it involves orthogonal coupling, gives a relatively small triazole group and can be made kinetically faster than (1) the conjugation of the ligand and the reactive group, (2) the hydrolysis of the reactive group and (3) the self-reactivity (Fig. 2a). The head–tail click reaction is actually completed in minutes using suitable copper catalysts at room temperature and 4-pentynoic acid as a tail model (Fig. 2b and Supplementary Fig. 2).

This modular approach has the advantage of affording multiple head–tail combinations that can be systematically tested to search for the best performing PTL (that is, the optimal reactive group and tether length). For example, **1** and **6** yield the full PTL compound **9** (Fig. 1c). Other combinations (compounds **10**–**20**) are shown and numbered in Supplementary Figs 3 and 4. The lengths of PTLs are calculated in Supplementary Table 1. As the NHS-based PTLs obtained by this method are very short-lived, we characterized the products of the click reaction crude of **9** (Supplementary Figs 5 and 6) and **10** (Supplementary Figs 7 and 8) after subsequent reaction with pure lysine as a mock protein residue. Detailed analysis by ultra performance liquid chromatography–mass spectrometry (UPLC–MS) confirmed the presence of the intended PTL-lysine adduct with an intact glutamate moiety. Some of the expected byproducts of Fig. 2a were also detected. Epoxide-based PTLs **11** and **12** were more stable and could be fully characterized (Supplementary Figs 9 and 10, respectively). We also verified that compounds **9** and **10** photoisomerized reversibly between 380 nm (violet) and 500 nm (green) illumination (Supplementary Fig. 11) with only minor differences in their absorption spectra and relaxation lifetime that can be attributed to the proximity between azobenzene and triazole^13^.

### Kainate receptors are photosensitized by NHS-based PTLs

We next aimed to quantify conjugation efficacy and photoresponses induced by compounds **9**, **10**, **11**, **13**, **14**, **15**, **18**, **19** and **20** in the wild-type kainate receptor GluK1, which displays high affinity for tethered glutamate ligands^14^. For that purpose, we performed electrophysiological recordings in a mammalian cell line under controlled illumination (see Methods). This set of PTLs spans different adduct lengths, linker variants and reactive groups, and allows sampling several side chains and conjugation sites on the protein surface. As these reactive groups are unstable in aqueous solution, PTL compounds were freshly prepared from the head and tail precursors of Fig. 1b. Cells expressing GluK1 were incubated at PTL concentrations between 12 and 25 μM (1–2% dimethyl sulfoxide, DMSO) during 2–10 min (see Methods) and were viable after washout. Following incubation with concanavalin A (Con A) to block GluK1 desensitization, PTL conjugation was assessed using whole-cell patch clamp recordings on perfusion of agonists, competitive antagonists, and violet and green illumination cycles to elicit photocurrents^15^. The GluK1 expression level in each cell was determined from the current evoked on perfusion of a full agonist, 300 μM glutamate, which was used to normalize responses. If a basal activation current was observed (evidenced by the holding current drop on perfusion of the competitive antagonist 6,7-dinitroquinoxaline-2,3-dione, DNQX) responses were then normalized to the sum of glutamate response and basal activation.

**Figure 3:**
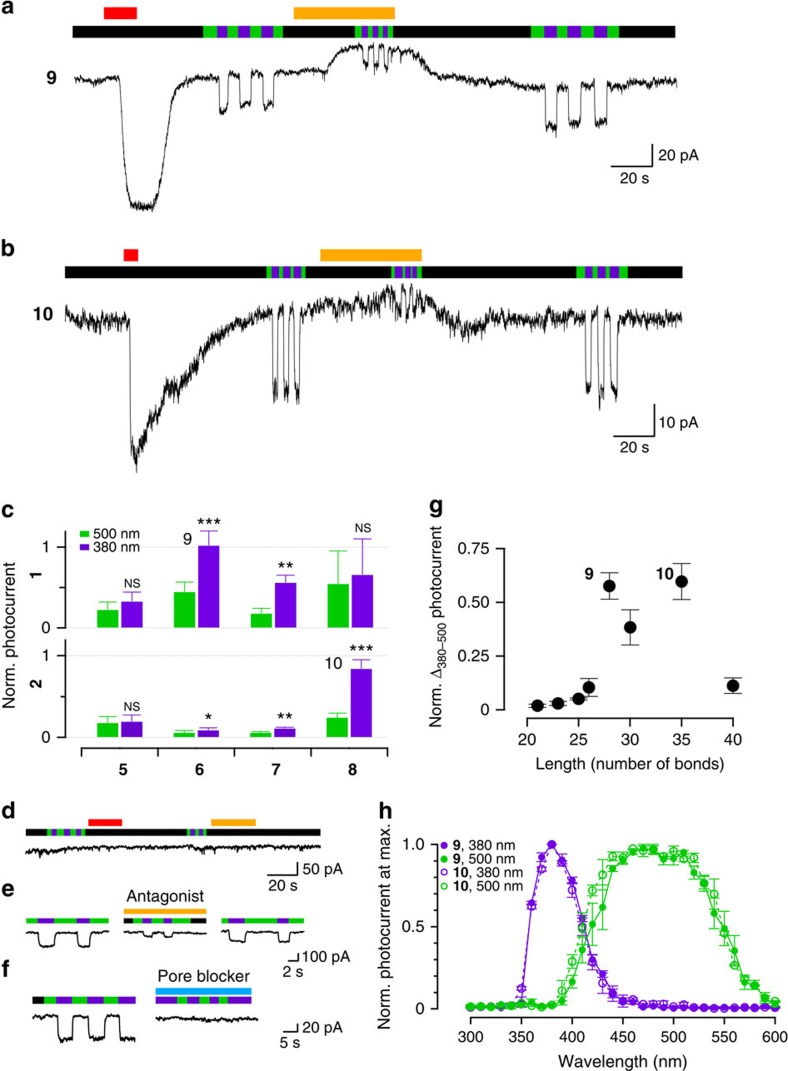
PTLs obtained by coupling different head and tail precursors can photosensitize wild-type GluK1 receptors. (**a**,**b**) Current recordings of cells overexpressing GluK1 receptors incubated with compounds **9** (**a**) and **10** (**b**). Channels open and close under violet (380 nm) and green (500 nm) illumination, respectively (black bars indicate no illumination). Perfusions of 300 μM glutamate are indicated by red bars. Reversible responses to 1 mM competitive antagonist DNQX (yellow bars) indicate covalent PTL conjugation and reveal a basal activation under green light and a high local effective PTL concentration under violet light. (**c**) Quantification of photocurrents of the corresponding PTLs (the different tail compounds (**5**–**8**) are indicated in the *x* axis, the upper row corresponding to combinations with compound **1** and the lower row to combinations with compound **2**). Compound **9**: *n*=5; compound **10**: *n*=7; the rest of combinations 2<*n*<5. Statistically significant differences from 500 nm photocurrent calculated by a paired *t*-test. ***P<0.001, **P<0.01 and *P<0.05. NS, not significant. (**d**) Cells not expressing GluK1 and incubated with compound **10** do not respond to light, glutamate and DNQX, thus discarding PTL unspecific effects (*n*=4). (**e**) Photocurrents from GluK1-expressing cells and conjugated with compound **10** (left) are reduced by perfusion of DNQX antagonist (middle) and recovered on washout (right; *n*=7). (**f**) Effect of a pore blocker on photocurrents recordered from cells expressing GluK1 conjugated with compound **9**. Left, light response before adding PhTX-433. Right, blocked photoresponses in the presence of PhTX-433 (3 μM, blue bar, *n*=3). (**g**) Photoresponses as a function of the PTL length (in number of bonds from the reactive carbonyl group to the C-4 of glutamate; see Supplementary Table 1). Photocurrent normalization is explained in the main text, equal *n* as in Fig. 3c. (**h**) Action spectra corresponding to compound **9** activation (
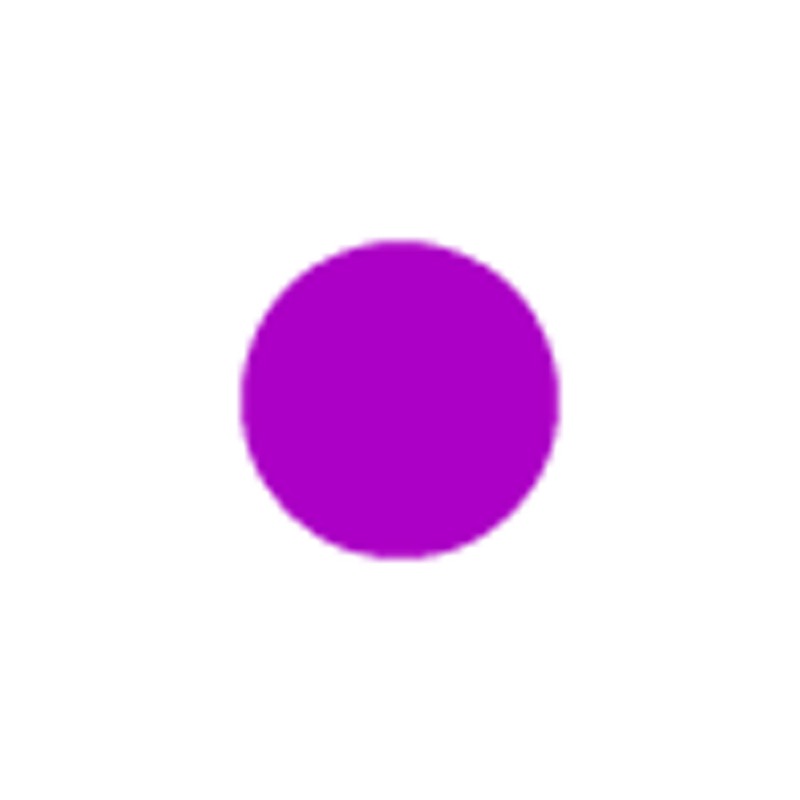
, *n*=3) and deactivation (
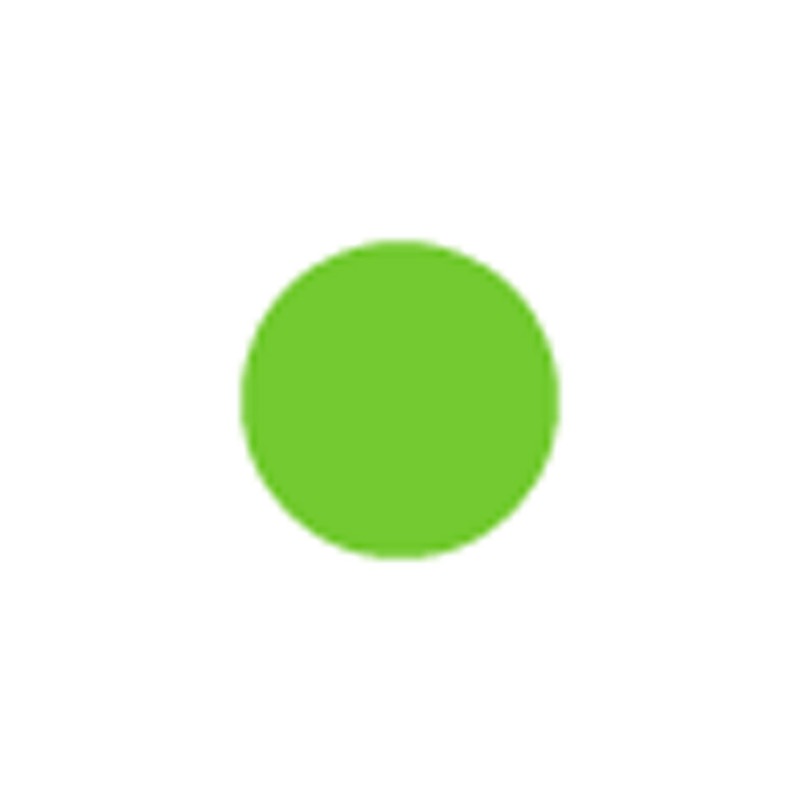
, *n*=5), and compound **10** activation (
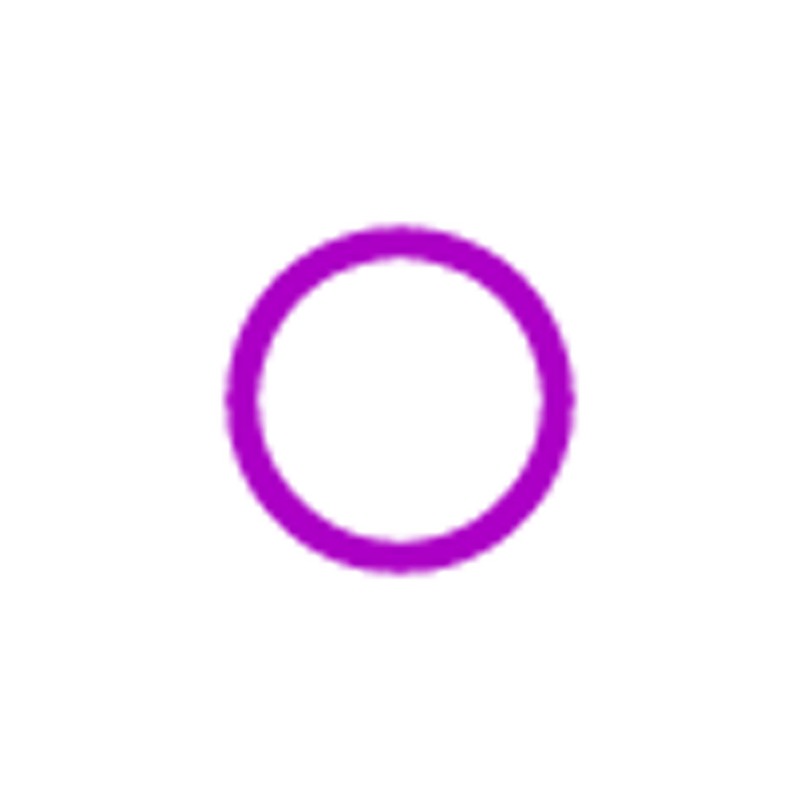
, *n*=2) and deactivation (
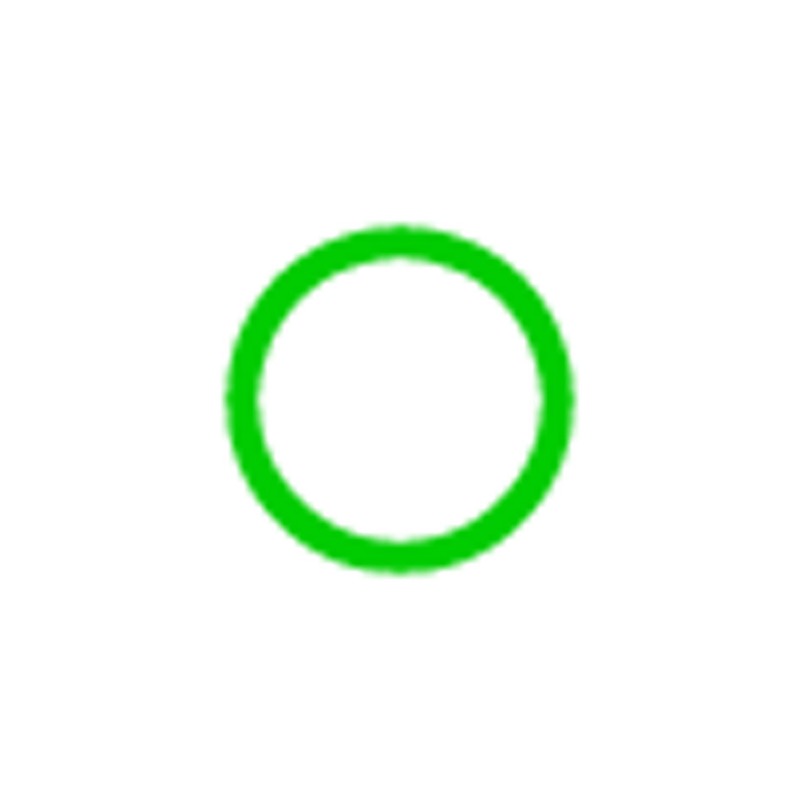
, *n*=2). Photocurrent normalized to the maximum photocurrent (see representative traces on Supplementary Fig. 14). Bars and data points are displayed as mean±s.e.m.

We first tested whether the selected electrophiles enabled efficient conjugation to GluK1 in our window of experimental conditions. Epoxide-terminated PTLs did not produce detectable light- or antagonist-dependent currents, suggesting poor conjugation to the receptor (Supplementary Fig. 12). In contrast, all NHS-based compounds in the panel enabled photocontrolling GluK1 currents to different extents, with **9** and **10** displaying large photocurrents indicative of compound conjugation (Fig. 3a,b, respectively). Photocurrent amplitude remained invariable along the experiment, despite the extensive washing after PTL incubation and the constant bath perfusion applied during all recordings, suggesting that the strong attachment of the photoswitch to the receptor is mediated by a covalent bond. To rule out membrane-related or other unspecific effects, we verified that photocurrents are not observed after incubating the compounds in cells not expressing GluK1 (Fig. 3d). In addition, DNQX antagonism of basal currents and photoresponses was fully reversible (Fig. 3e). The photocurrent was only partially reduced by DNQX, indicating a high local effective concentration of the covalently bound agonist when azobenzene is in the *cis* configuration^15^. To complete the characterization, we used the pore blocker philanthotoxin-433 (PhTX-433) that prevented the induction of photoresponses by compound **9** (Fig. 3f). Taken together, these results demonstrate that photocurrents elicited by NHS-based compounds are mediated by ligands covalently tethered to wild-type GluK1.

To identify the PTL producing optimal responses, we analysed the normalized photocurrent at 500 and 380 nm (Fig. 3c) for each compound in the library. Photocurrent change (Δ_380–500_) and basal current (500 nm photocurrent) are plotted as a function of the calculated compound length (Fig. 3g and Supplementary Fig. 13). Interestingly, the photocurrent change increases abruptly with compound length and is optimal between 28 and 35 bonds, which comprise compounds **9** and **10**. The basal activation under green light displays a weaker dependence with the PTL length and corresponds to partial binding of the ligand in the *trans* configuration of azobenzene^15^. Action spectra obtained from GluK1 photocurrents at different illumination wavelengths (Fig. 3h and Supplementary Fig. 14) are similar for **9** and **10** with on-switching peaks at 380 nm and a wider off-switching band centred around 500 nm, in agreement with the results of Supplementary Fig. 11 and other reports of similar azobenzenes^13^. The PTL shows a full agonist character (Supplementary Fig. 15) and photosensitivity (Supplementary Fig. 16) in line with previously described photoswitches.

### NHS-based PTLs are conjugated by affinity labelling

**Figure 4:**
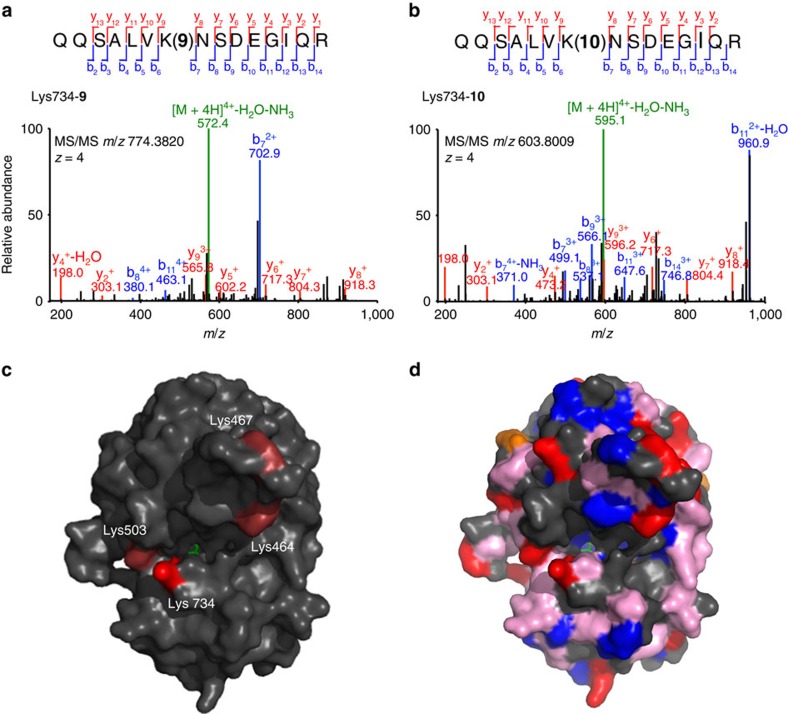
Identification of GluK1 Lys734 as main conjugation site of compounds 9 and 10 by LC–MS/MS. Representative MS/MS spectra showing the tryptic fragments conjugated to compound **9** (**a**) and compound **10** (**b**). The sequence, mass and the charge (*z*) of each precursor peptide ion are denoted in the figure. The *y*- and *b*-ion fragments that were assigned (coloured red and blue, respectively) are indicated along the peptide sequence backbone. Amino acid numbers refer to positions in the full-length GluK1 protein, in which Lys734 corresponds to Lys171 in the LBD of GluK1 (ref. 48). (**c**) Structure of the LBD of GluK1 (PDB 1txf) with glutamate bound (green) and the main residues targeted by compound **9**: Lys734 is shown in bright red colour (corresponding to a conjugation ratio of 5.9±0.90, *n*=3; see Supplementary Fig. 18) and Lys503, Lys467 and Lys464 are shown in light red colour (with a compound **9** conjugation ratio below 0.05). (**d**) Structure of the GluK1 LBD highlighting all solvent-exposed residues bearing nucleophilic groups that could potentially react with the electrophile NHS ester^49^,^50^,^51^. They include ɛ-amino groups of lysine side chains (red), hydroxyl groups of serine, threonine and tyrosine (blue) whose reactivity is increased by the close proximity of histidines (orange), and weaker nucleophilic groups such as methionine, tryptophan, glutamine, asparagine and arginine (pink).

**Figure 5:**
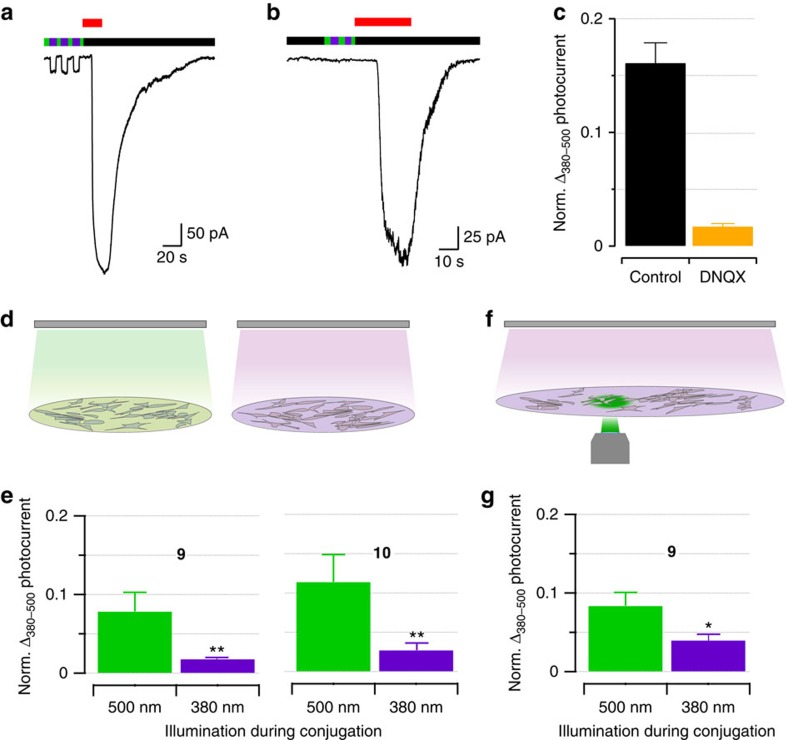
PTL conjugates to GluK1 by affinity labelling. (**a**–**c**) PTL conjugation to GluK1 is reduced by a competitive ligand. (**a**,**b**) Current recordings of GluK1-expressing cells incubated with compound **9** (2.5 μM, 10 min at pH 8) in the absence (**a**) or the presence of competitive antagonist DNQX (1 mM) (**b**). Green, violet and black bars indicate illumination at 500 nm, 380 nm and no illumination, respectively. Glutamate responses (300 μM, red bar) were used to normalize photocurrents to GluK1 expression level. (**c**) Quantification of normalized photocurrent difference after incubation in compound **9** with DNQX (orange, *n*=8; black is control, *n*=5). (**d**–**g**) PTL conjugation is favoured under green illumination. (**d**) Cells were conjugated to PTLs under green light (left) or under violet light (right). (**e**) Normalized photocurrent difference produced by compound **9** and **10** (left and right panels, respectively) incubated under green and violet light (respectively 0.018±0.002, *n*=4 and 0.08±0.02, *n*=4 for compound **9**, and 0.027±0.009, *n*=6 and 0.11±0.02, *n*=7 for compound **10**). Significance calculated by Mann–Whitney *U*-test, **P<0.01. (**f**) Compound **9** conjugation is favoured in regions of the coverslip illuminated with green light (through a × 40/0.65 objective from below the sample, whereas a violet LED lamp was applied from above; see Methods). (**g**) Normalized photocurrent difference produced by compound **9** in green- and violet-illuminated regions (respectively 0.08±0.02, *n*=3 and 0.04±0.008, *n*=6). Significance calculated by Mann–Whitney *U*-test, *P<0.05. All bars are mean±s.e.m.

The strategy of rapidly clicking amine-bearing head compounds and amine-reactive tails is remarkably successful to obtain a combinatorial library of PTLs to be tested in wild-type receptors. It allows sampling and optimizing design parameters such as the nature of the reactive group and the linker properties, and affords almost complete conjugation and photoswitching by kinetically controlling the coupling steps. However, it is not obvious why a non-selective electrophile such as NHS ester^16^ can lead to the seemingly specific photoresponses of Fig. 3a,b. Indeed, we identified four lysine residues as the main sites for conjugation of **9** and **10** to GluK1 (primarily Lys734 and, to a lesser extent, Lys503, Lys467 and Lys464; see Fig. 4, Supplementary Figs 17 and 18 black bars and Supplementary Tables 2–4), despite the great variety of nucleophilic reactive sites over the protein surface (Fig. 4d). Even if NHS esters reacted preferentially to the primary amine in lysine, there are 20 such residues exposed on the surface of the GluK1 ligand-binding domain (LBD). One possible explanation of the site-selective conjugation of the PTL is that the reaction is guided to its target by the interaction between the glutamate moiety in the ligand and its binding site in the protein, that is, by ‘affinity labelling'^17^, as suggested by the proximity of the identified lysines to the glutamate-binding site (Fig. 4c). To test this hypothesis, we interfered with PTL binding to the receptor by two means: using competitive antagonists or photoisomerizing the PTL. In the first case, we quantified photoresponses after incubating GluK1-expressing cells in diluted (2.5 μM) compound **9** alone (Fig. 5a) or under excess of DNQX competitor (1 mM; Fig. 5b). After washout, cells incubated in DNQX displayed reduced photocurrents (Fig. 5c), thus confirming that reversible binding of the PTL to the glutamate binding site is required for efficient irreversible conjugation to the receptor. In the second case, we reasoned that as binding of the glutamate moiety of the PTL to the receptor active site presumably helps targeting it to a reactive side chain in the vicinity (Fig. 4c,d), then it should be possible to observe differences in the conjugation of the PTL when the molecule is extended (*trans* configuration) or bent (*cis* configuration). Hence, we incubated GluK1-expressing cells in 2.5 μM PTL (**9** or **10**) under 500 or 380 nm illumination using an light emitting diode (LED) array (Fig. 5d). After washout, quantification of the photocurrents showed that PTL conjugation can be controlled with light: cells incubated under green light display significantly higher photoresponses than cells incubated under violet light (Fig. 5e). In the purified LBD, only minor changes in PTL conjugation with light could be detected (Supplementary Fig. 18 violet bars and Supplementary Tables 5 and 6). We discarded that the reduced photoresponse of cells incubated under violet light (*cis* state) is due to light-triggered hydrolysis of the NHS group or PTL cyclization, as the same chemical species were identified by UPLC–MS regardless of the illumination conditions. Instead, the light dependence of PTL conjugation could be caused by structural differences, such as the effective shortening of the PTL on isomerization (∼0.82 nm or 6–7 bonds), which corresponds to weaker photoswitching in agreement with the results of Fig. 3g. Another possibility is that violet light reduces the binding affinity of the freely diffusible, reactive PTL, thereby decreasing the effective concentration of the NHS ester group in the vicinity of the lysines and hence reducing the conjugation of the PTL to the protein. The free-ligand behaviour of compound **10** supports this explanation (Supplementary Fig. 19 and ref. 14)^18^. Besides, we also mutated the main lysine but this does not abolish photoresponses after conjugation to **9**, suggesting that other residues can act as surrogate conjugation sites of the photoswitch (Supplementary Fig. 20). In summary, our new PTLs feature a high-affinity receptor ligand and an unselective electrophilic group, but this group does not react with all nucleophiles exposed on the protein surface. Instead, covalent bond formation is kinetically favoured over competing reactions (Fig. 2) after ligand binding to the receptor site, probably by the proximity of the reactive tail to a suitable nucleophile. To account for this characteristic mechanism, we refer to these compounds as TCPs^9^.

Beyond the mechanistic insight, light-regulated labelling conditions can be exploited to spatially control TCP conjugation. To demonstrate light-patterned conjugation, the coverslip was incubated with 2.5 μM compound **9** and placed on an inverted microscope where the cells within the focused field area were illuminated at 500 nm and high intensity through the objective (to favour conjugation), and the entire coverslip was illuminated at 380 nm from above with an LED array (Fig. 5f). After 10 min, cells were washed out and photocurrents were recorded from cells located in violet- and green-illuminated regions. Cells exposed to **9** under 500 nm illumination displayed photoresponses that doubled those from cells incubated under 380 nm (Fig. 5g). In this way, light-dependent conjugation allows selecting the areas to be labelled with the photoswitch^15^ and introduces a new way to localize the modification of wild-type receptors.

### TCPs control neuronal activity with light

**Figure 6:**
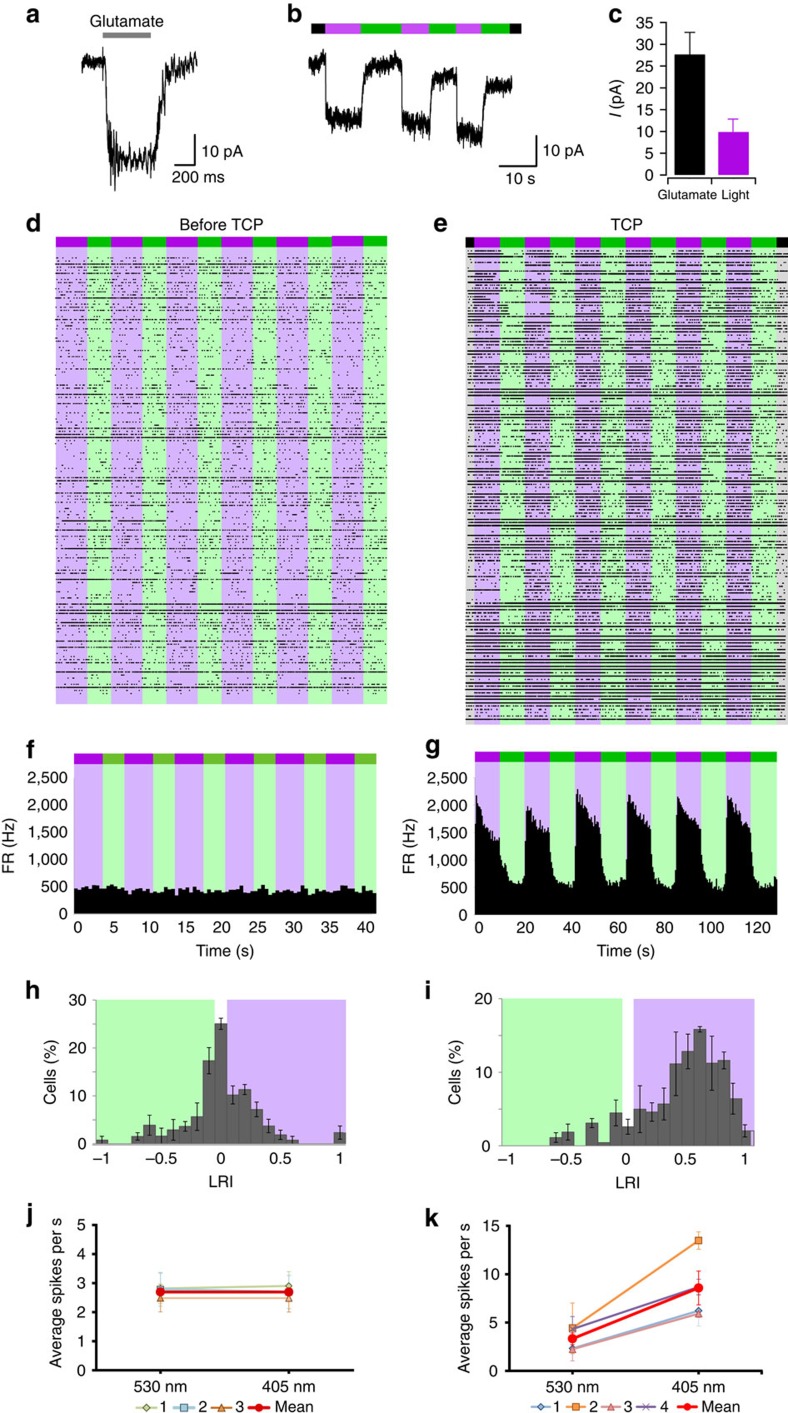
TCPs directly photosensitize DRG neurons and restore photoresponses in degenerated retina. (**a**,**b**) Whole-cell currents recorded in voltage-clamp mode at −60 mV from untransfected DRG neurons incubated with 6 μM of compound **9** and Con A. Neurons that responded to fast glutamate perfusion (grey bar labelled 10 mM, **a**) also displayed robust photoresponses to violet light followed by a green-light pulse (indicated by violet and green bars, **b**). These responses were absent in neurons not responding to glutamate (Supplementary Fig. 21). (**c**) Mean current amplitude in response to glutamate and light for DRG neurons incubated with 6 μM of compound **9** (*n*=21 for glutamate responses and *n*=5 for photoresponses). (**d**–**k**) Compound **9** was further tested in degenerated retina from *rd10* mice, which is not responsive to light of 380/500 nm as shown in the raster plot of a multi-electrode array (MEA) from flat-mounted retinas (**d**). The integrated time course of the firing rate (FR, **f**), the LRI (**h**) and the average firing rate of the control experiments (marked red in **j**, *n*=3 retinas) do not display any photoresponses. In contrast, incubation of the retina with compound **9** for 3 min is enough to robustly photosensitize the degenerated retina, as shown in the full raster plot (**e**) and the integrated firing rate of four experiments (**g**). The LRI histogram (**i**) is shifted towards positive values corresponding to violet light and the average firing rate (red in **k**, *n*=4 retinas) is significantly higher under violet light than under green light. Bars are mean± s.e.m.

To demonstrate that TCPs can be conjugated to native receptors in genetically unmodified cells, we assayed them on dorsal root ganglion (DRG) neurons, where GluK1 is the major expressed glutamate subunit^19^. Dissociated DRG neuron cultures were incubated with compound **9** and Con A, and washed out. Whole-cell currents were recorded while clamping the neuron membrane potential at −60 mV. Neurons that responded to fast glutamate perfusion (Fig. 6a) were exposed to a violet light pulse followed by a green light pulse. A rapid and reversible inward photocurrent corresponding to 26% of the 10 mM glutamate-evoked current was observed (Fig. 6b,c) and was absent in neurons not responding to glutamate (Supplementary Fig. 21).

TCPs were further tested in a mice model of retinal degeneration. We used retinal degeneration 10 (*rd10)* mice, which carry a spontaneous mutation of the rod-phosphodiesterase gene, leading to a rod degeneration that starts around P18. Later, cones are also lost. The electroretinogram response in these animals is usually undetectable at 2 months of age and all our experiments were performed at 9 months of age^20^. Retinal bipolar (RB) cells are non-photosensitive neurons that get their synaptic input from photoreceptor cells in the healthy retina. The major synaptic receptor in RB cells is GluK1 (refs 21, 22) and its expression persists under photoreceptor degeneration^23^. Extracellular recordings were obtained using a multi-electrode array, which enabled to record simultaneously the electric activity of many retinal ganglion (RG) cells as a function of the illumination conditions (dark, violet or green light, see Methods). The degenerated retina of *rd10* mice is insensitive to illumination, as can be observed in the full raster plot of Fig. 6d and in the integrated time course of the firing rate (Fig. 6f). In consequence, the light response index (LRI^5^) is distributed around zero (Fig. 6h) and the average firing rate does not change with light (Fig. 6j). Application of freshly prepared TCP **9** for 3 min followed by mild rinse (see conditions in Methods) is enough to robustly photosensitize the degenerated retina, as shown in the raster plot and the integrated firing rate (Fig. 6e,g, respectively). The LRI histogram of Fig. 6i is strongly shifted towards positive values (corresponding to violet light) and the average firing rate is significantly higher under violet light than under green light (Fig. 6k). Retinal photoresponses were reliably observed for several hours after the 3-min incubation with TCP **9** and the subsequent washing (Supplementary Fig. 22).

## Discussion

A convergent synthetic method to obtain amine-bearing, amine-reactive compounds has been developed and demonstrated by covalently conjugating a PTL to a wild-type kainate receptor. A previous report of a photoswitch tethered to a wild-type receptor took advantage of a disulfide bond near the ligand-binding site that was reduced before conjugation^24^. Reducing agents cannot be applied *in vivo* but enable the use of mild, cysteine-selective electrophiles (alkyl halides and maleimide). Acrylamide and epoxide groups have also been tested in PTLs of potassium channels^25^ but were later found to be not covalently bound (that is, to work as PCLs^26^). Thus, TCPs are the first PTLs that can be used directly in untreated endogenous receptors and introduce a new class of photopharmacological covalent drugs.

This chemical method uses highly reactive electrophiles and, under proper conditions, is not limited by the presence of nucleophiles in the ligand moiety. A rapid coupling of two precursors enables freshly preparing the short-lived reactive ligands and testing different combinations of tethers and reactive groups, which is essential to optimize TCP performance. A common limitation of reactive chemical methods for biological applications is their reduced biological specificity^27^. Here we take advantage of the ligand moiety as a ‘biospecific tag' that targets the TCP to the receptor of interest before covalent bond formation. This property is also exploited by targeted covalent drugs, a family of highly effective and successful medicaments including aspirin, penicillin, omeprazole or clopidogrel^9^. The affinity labelling process followed by TCPs meets the need for site-specifically tethering ligands in wild-type proteins and should bear general application (for example, in fluorescent or radioactive affinity tags).

Interestingly, in the case of GluK1, solution-exposed lysines in the LBD are evolutionary conserved (Supplementary Fig. 23). Therefore, the TCPs presented here might work in a variety of organisms including human and important translational models (chicken, pig, cat, dog and macaque) for which limited (opto)genetic manipulation techniques are currently available^28^. This prospect makes the results on retina photosensitization especially appealing. Compared with available methods to restore retinal photoresponses such as retinal implants, TCPs can help avoid surgery and provide better coupling to photostimulation than a physical device, as well as shortening rehabilitation by taking advantage of natural processing in the retina. TCPs provide similar photoresponses to LiGluR^4^,^29^ and quaternary ammonium photoswitches^5^, without requiring genetic manipulation and targeting synaptic receptors rather than potassium channels involved in action potential propagation. Synaptically expressed receptors such as GluK1 in RB cells^21^,^22^ offer potentially better spatial resolution for light stimulation than proteins expressed in axonal processes, which extend over relatively large distances in the case of RG cells. Unlike RG cells, which are located downstream in the signalling circuit and project directly to the brain through the optic nerve, RB cells are postsynaptic to photoreceptors (that is, located upstream in the network) and have been recently targeted by optogenetic strategies^29^,^30^ to take advantage of signalling in the surviving retina. However, GluK1 can be also expressed in RG cells and probably in some amacrine cells^31^, and TCPs might activate other receptors as well. Thus, more selective compounds would be desirable.

The photocontrolled conjugation of TCPs shown in Fig. 5f,g could be used to ‘paint in' molecular photoswitches with different colour sensitivities in spatial patterns that recreate the specific distribution of photoreceptor cells (cones and rods). TCPs could also be developed for other neurotransmitters (Fig. 1), to wirelessly reconnect injured neurons with light, remotely regulating pain^32^ or secretion in neuroendocrine cells^7^,^33^. Thus, TCP molecules can be regarded as nanoprostheses to remotely drive the endogenous receptors that remain in the cells to be treated. The therapeutic opportunities of photoregulating endogenous proteins with photoswitchable compounds would not require microbial protein overexpression or gene therapy and would only be subject to conventional drug assessment tests *in vitro* and in a variety of animal models.

## Methods

### General procedure for click reaction

To a solution of azide (compounds **1** or **2**, 1 mg, 1 equiv), NaAsc (4 equiv) and Cu_2_O (2.4 equiv) in water (14 μl) was added a solution of alkyne (compounds **3**–**8**, 1.1 equiv) in tetrahydrofuran (THF) (4 μl) and the reaction was stirred for 30 min at room temperature. For the use of compounds generated in cells, the reaction mixture was immediately diluted ten times with DMSO, with a final concentration of compound **1** or **2** of 12 mM.

### Compound and side product characterization

After the general procedure for click reaction, L-lysine (2 equiv) in water (5.3 μl) was added to the reaction mixture. After 30 min, 1 μl of the reaction mixture was removed and diluted with 1,000 μl of a mixture CH_3_CN:DMSO (1:1) and analysed by high-resolution MS.

High-resolution MS analyses were carried out at the IQAC Mass Spectroscopy Facility, using a UPLC–electrospray ionization–time of flight equipment (Acquity UPLC BEH C18 1.7 mm, 2.1 × 100 mm, LCT Premier Xe, Watters). The two mobile phases were A: CH_3_CN and B: H_2_O; both contained 20 mM HCOOH as well. The flow rate was 0.3 ml min^−1^. A linear gradient was programmed as follows: 0.0 min: 95% B; 2.69 min: 95% B; 8.69 min: 5% B; 10.94 min: 5% B; 11.69 min: 95% B; ad 13.19 min: 95% B.

### Photoactivation of GluK1 in cell line

HEK293 tsA201 cell line (SV40-transformed, human embryonic kidney 293 cells) was maintained at 37 °C in a 5% CO_2_ humid incubator with DMEM medium/nutrient mixture F-12 media (1:1, Invitrogen) supplemented with 10% fetal bovine serum (Biological Industries) and 1% penicillin/streptomycin. Cells transiently expressed the receptor subunit GluK1(Q)-2b(GGAA), kindly provided by G. Swanson (Northwestern University Feinberg School of Medicine), with the endoplasmic reticulum retention motif of the carboxy-terminal mutated to increase surface expression^34^. GluK1(Q)-2b-GGAA plasmid was co-transfected with peGFP using X-tremGENE 9 Transfection Reagent (Roche) following the manufacturer's protocol, with a Transfection Reagent:GluK1:eGPF ratio of 3:1:0.1. Cells were detached and freshly plated into a 12-mulltiwell plate at a density of 3 × 10^5^ cells before the DNA-Transfection Reagent mix was added dropwise into each well. Experiments were performed after 48–72 h, and the day before the experiment cells were plated at low density on 15-mm coverslips treated with collagen.

Before each experiment, coverslips were incubated with the compound freshly prepared for 2 min in pH 9 bath solution or 10 min in pH 8 bath solution, at final concentrations ranging between 2.5 and 25 μM. After compound incubation, cells were washed three times with physiological bath solution and incubated 10 min with 0.3 mg ml^−1^ Con A, to block GluK1 desensitization. To avoid membrane depolarization due to ion flux through desensitized receptors, Con A was diluted in a Na^+^-free solution based on NMDG^+^ and composed by (in mM): 110 NMDG^+^, 2.5 KCl, 1 MgCl_2_, 10 HEPES and 10–20 mM glucose to fix osmolarity to 300 mOsm kg^−1^, pH 7.4 adjusted with HCl. Before placing the coverslip to the recording chamber, cells were extensively washed with physiological bath solution.

*Conditions of regular incubation*. Cells were incubated with compounds **9**–**20** at a concentration between 12 and 25 μM (1% DMSO) for 2 min, in the absence of light and in pH 9 bath solution composed by (in mM): 100 NaCl, 1 MgCl_2_, 2.5 KCl, 2.5 CaCl_2_, 10 glucose and 50  sodiumcarbonate/sodiumbicarbonate, 310 mOsm kg^−1^, pH 9 adjusted with NaOH.

*Conditions of affinity labelling incubation*. Cells were incubated with 2.5 μM of compound **9**, 1% DMSO in pH 8 bath solution alone or in competition with 1 mM DNQX for 10 min (Fig. 5a–c). Composition of pH 8 bath solution is the same as the bath solution used for recordings, with pH adjusted at 8 with NaOH.

*Conditions of conjugation under illumination*. Cells were incubated with compound **9** or **10** (2.5 μM, 1% DMSO) in bath solution at pH 8 for 10 min and under 500 or 380 nm light illumination (Fig. 5d–g). Conjugation under illumination was done with 500 or 380 nm LED array (FCTecnics) placed at 2.5 cm from the coverslip, with light intensity of 0.9 and 1.7 mW mm^−2^, respectively. The same 380 nm LED array was used for the spatial controlled conjugation (Fig. 5f,g). It was positioned at 2.5 cm above the coverslip placed on the microscope, whereas cells were illuminated at 500 nm through the objective.

Voltage-clamp recordings under whole-cell configuration were done using an EPC-10 amplifier and data at 1 kHz was acquired using a free-run protocol with amplifier's software Patch Master (HEKA). Cell membrane was held at −70 mV. Borosilicate glass pipettes were pulled with a typical resistance of 4–6 MΩ and filled with pipette solution containing (in mM): 120 cesium methanosulfonate, 10 TEA-Cl, 5 MgCl_2_, 3 Na_2_ATP, 1 Na_3_GTP, 20 HEPES, 0.5 EGTA and 290 mOsm kg^−1^, and pH 7.2 was adjusted with CsOH. Cells were continuously perfused with bath solution composed of (in mM): 140 NaCl, 1 MgCl_2_, 2.5 KCl, 10 HEPES, 2.5 CaCl_2_ and 10–20 glucose to fix osmolarity to 310 mOsm kg^−1^, and pH 7.42 adjusted with NaOH. Fast switching of solutions was done with VC-6 six-channel valve controller (Warner Instruments Corp.). To activate and inhibit GluK1, solutions of 300 μM glutamate or 1 mM DNQX in bath solution were perfused.

Photostimulation during recordings was done by illumination of the entire focused field using a Polychrome V monochromator (TILL Photonics) connected through the back port of an IX71 inverted microscope (Olympus) with a CP-ACHROMAT × 40/0.65 objective (Zeiss) and a fully reflective aluminum mirror (Chroma). The monochromator was connected to a personal computer, and shutter and wavelength were controlled using PloyCon software (TILL Photonics). Light power measured with the Newport 1916-C light meter after the objective was 1.6 mW mm^−2^ for 425 nm, 0.8 mW mm^−2^ for 380 nm and 1.8 mW mm^−2^ for 500 nm

All data were exported to calculate current amplitudes with IgorPro (Wavemetrics). All statistics were done with Microsoft Excel (Microsoft), except statistical tests, which were performed with Matlab (Mathworks). Displayed whole-cell current traces have been filtered using the inifinte impulse response digital filter from IgorPro (low-pass filter with cutoff of 50 Hz).

### Site-directed mutagenesis

Amino acid substitutions of lysine 734 and 497 to alanine (Lys734Ala–Lys497Ala) into the GluK1(Q)-2b-GGAA plasmid were done by using the QuikChange Lightning Multi Site-directed mutagenesis (Agilent) following the manufacturer's guidelines. Successful mutagenesis was confirmed by DNA sequencing.

### Expression and purification of GluK1 S1S2 LBD

The pET-22b GluK1 S1S2 plasmid was provided by M. Mayer (National Institutes of Health). The recombinant GluK1 S1S2 protein encodes the segments S1 and S2 that conform the LBD of GluK1; it was expressed and purified with some modifications to the original protocol^35^. Transformed Origami B DE3 cells were grown at 37 °C to a *D*_600nm_ of 1 in Luria-Bertani medium (LB medium) and then cooled on ice to 19 °C. Protein expression was induced by addition of a final concentration of 30 μM isopropyl β-D-thiogalactoside o/n at 18 °C. After 20 h of induction, cells were harvested by centrifugation and pellets were freezed at −80 °C for at least 1 h. Pellets were then resuspended in 10 ml l^−1^ culture of phosphate buffer (50 mM Na_2_HPO_4_ pH 7.0, 300 mM NaCl, 5 mM MgCl_2_ and 2 mM glutamate) containing a cocktail of protease inhibitors (Sigma, catalogue number P8849) and lysed by sonication. The lysate was cleared by centrifugation and the supernatant applied to gravity flow column containing 1 ml of TALON Co^2+^ resin slurry (Clontech). The resin was washed with phosphate buffer containing 10 mM imidazole and eluted with a linear gradient of imidazole (from 50 to 200 mM) in phosphate buffer. Eluates were extensively dialysed againts HEPES buffer (100 mM pH 7.5) and followed by thrombin cleavage of the histidine tag (CleanCleavageTM Kit). Purified protein solution contained 100 mM HEPES pH 7.5, 100 mM NaCl, 1 mM EDTA and 10% glycerol (wt/vol). Purity and integrity of the recombinant protein was analysed by SDS–PAGE.

### Conjugation of purified GluK1 S1S2 to reactive photoswitches

To conjugate the GluK1 LBD to compounds **9** and **10**, recombinant GluK1 S1S2 protein was incubated with a 25–30 molar excess of each compound, at a final protein concentration of 4 μM (for LC–MS intact protein analysis) and 2 μM (for MS/MS analysis) in HEPES buffer (100 mM pH 7.5) for 20 min at room temperature. Control reactions were incubated with equivalent amounts of DMSO. The excess reagents were removed and the buffer exchanged to 100 mM NH_4_AcO using Amicon Ultra centrifugal filters (Millipore).

Gluk1 conjugation under illumination was done in the presence of a 3-molar excess of compound **9** at a final protein concentration of 2 μM. Reactions were incubated for 20 min at room temperature at dark or illuminated with a 380-nm LED array (FCTecnics) as described above.

### LC–MS analysis of GluK1 S1S2 conjugates

A 1% in volume of formic acid (FA) was added to each sample. Protein samples were injected automatically to a BioSuite pPhenyl 1000 (Waters Corp., Milford, MA, 10 μm RPC 2.0 × 75 mm) column at a flow rate of 100 μl min^−1^ using a Finnigan, Mod. Surveyor MS chromatographic system (Thermo Electron Corporation) provided with an autosampler Finnigan, Mod. MicroAS. Samples were eluted using a linear gradient from 5 to 60% *B* in 55 min and from 60 to 80% *B* in 5 min (*A*=0.1% FA in water, *B*=0.1% FA in CH_3_CN). The column outlet was directly connected to an AdvionTriVersa NanoMate (Advion) fitted on an LTQ-FT Ultra mass spectrometer (Thermo). The Nanomate was used as an interface to perform nano-electrospray ionization through chip immunoprecipitation technology and as a flow diverter (1:250 Split). Sprayvoltage in the NanoMate source was set to 1.70 kV. The mass spectrometer acquired full MS scans (400–2,000 *m*/z) in the FT with the resolution (R) set to 100,000 (R is defined by *mD* μm^−1^ 50% at 400 *m*/z). Ion transmission into the FTICR cell was automatically controlled for optimal performance of the analyser by setting the charge capacity to a 1,000,000 counts target value. Capillary voltage and tube lens on the LTQ-FT were tuned to 40 and 120 V. The spectrometer was working in positive polarity mode.

Data were acquired with Xcalibur software (vs. 2.02 SR2) and MS spectra showing protein-charged species were deconvoluted using ProMass software vs 2.8 (Thermo Scientific) to the zero-charged monoisotopic masses.

### LC–MS/MS analysis

Gluk1 S1S2 conjugation products were digested with trypsin following In-Liquid digestion protocol^36^. Samples were diluted to 0.12 μg μl^−1^ with 50 mM NH_4_HCO_3_, reduced with dithiothreitol 2 mM for 1 h and carbamidomethylated for 30 min in the dark with IAA 5 mM. The reaction was quenched with dithiothreitol 2 mM and proteins digested with trypsin (2% wt/wt) at 37 °C overnight. The digestion was stopped by adding formic acid to a final concentration of 1% in volume. Samples were diluted to 20 μl of 1% formic acid aqueous solution to 1 pmol μl^−1^ and 1 pmol on column was injected. For the LC–MS/MS analysis, digested peptides were loaded to a 180 μm × 2 cm C18 Symmetry trap column (Waters Corp.) at a flow rate of 15 μl min^−1^ using a nanoAcquity Ultra Performance LCTM chromatographic system (Waters Corp.). Peptides were separated using a C18 analytical column (BEH130 C18 75 mm × 25 cm, 1.7 μm, Waters Corp.) with a 90-min run, comprising consecutive steps with linear gradients from 1 to 35% *B* in 60 min, from 35 to 50% *B* in 5 min (*A*=0.1% FA in water, *B*=0.1% FA in CH_3_CN). The column outlet was directly connected to an Advion TriVersa NanoMate (Advion) fitted on an LTQ-FT Ultra mass spectrometer (Thermo). Spray voltage in the NanoMate source was set to 1.70 kV. Capillary voltage and tube lens on the LTQ-FT were tuned to 40 and 120 V. The spectrometer was working in positive polarity mode and singly charge-state precursors were rejected for fragmentation. At least one blank run before analysis was performed, to ensure the absence of cross-contamination from previous samples.

A first analysis to identify digested peptides was performed in a data-dependent acquisition mode. Survey MS scans were acquired in the FT with the resolution (defined at 400 *m*/z) set to 100,000. Up to six of the most intense ions per scan were fragmented and detected in the linear ion trap. The ion count target value was 1,000,000 for the survey scan and 50,000 for the MS/MS scan. Target ions already selected for MS/MS were dynamically excluded for 15 s. Minimal signal required to trigger MS to MS/MS switch was set to 1,000 and activation *Q* was 0.250.

A database search was performed with Proteome Discoverer software v1.4 (Thermo) using Sequest HT search engine and SwissProt database (rat, ecoli and common repository of adventitious proteins databases with user protein). Searches were run against targeted and decoy databases to determine the false discovery rate. Search parameters included trypsin enzyme specificity, allowing for two missed cleavage sites, methionine oxidation, carbamidomethyl and compound **9** (+739.318 Da) and compound **10** (+648.266 Da) in lysine as dynamic modifications. Peptide mass tolerance was 10 p.p.m. and the MS/MS tolerance was 0.6 Da. Peptides with a *q*-value <0.1 and a false discovery rate <1% were considered as positive identifications with a high confidence level.

Data-dependent acquisition information was used to select 11 peptides, which contain modified lysines of interest, for targeted MS/MS analysis (Supplementary Tables 4–6). Three bioreplicates per condition (dark, violet) and two technical replicates were analysed. The analysis was performed by using the same nano-LC–MS/MS system described above. The spectrometric analysis was done in a targeted mode, acquiring a full MS/MS scan of the selected precursor ions. To obtain good extract ion chromatograms, retention time-range windows were selected and a maximum of five ions were acquired in each window. Quantitative targeted MS/MS analysis was performed using Skyline v3.1.0.7382, an open source software project^37^. A spectral library was generated in Skyline from database searches of the targeted MS/MS raw files with Proteome Discoverer v.1.4 (Thermo). The final selected peptides were manually imported within Skyline. Peaks were picked in an automated manner using the default Skyline peak picking model, using Savitzky–Golay smoothing. Integrated peak areas were based on extracted ion chromatograms of up to three highest ranked MS/MS fragment ions masses, typically *y*- and *b*-ions, matching to specific peptides present in the spectral library. All transitions, peak areas and Comp 9 assignments were manually validated. Ratios of peptides containing K-comp9/K-No modified were calculated from peak areas for both technical replicates of all samples. These ratios were used for statistical analysis without normalization. Data were first transformed to log2 scale, to apply a linear model with random effects. Groups (violet, dark) were selected as fixed effect and technical replicates were set as random effects. Model fitting was accomplished with the lme4 function of the lme4 package^38^,^39^. Comparison between groups was done to find out which species significantly changed. Confident interval selected at 95% and *P*-values were also calculated from the linear model.

### Photoresponses in DRG neurons

Experimental procedures were performed in accordance with the guidelines of the European Commission (86/609/CEE) and were supervised by the veterinary officer at the Instituto de Neurociencias de Alicante. Cell culture of dissociated DRG neurons were prepared from postnatal day 0 P0-C57 male and female mice^40^. Dorsal root ganglia were dissected out and enzymatically digested with collagenase (1.25 mg μl^−1^), DNase (50 μg ml^−1^) and trypsin (0.125%) in Hank's balanced salt solution for 40 min at 37 °C. DRG ganglia were sedimented and Hank's balanced salt solution was exchanged with fresh DRG culture media without nerve growth factor and with DNase (75 μg ml^−1^). Next, DRG neurons were dissociated with a flame-polished Pasteur pipette. Cells were centrifuged and resuspend in complete DRG culture media. Dissociated cells were plated at a cell density of 2,500 cell·per cm^2^ on poly-L-lysine/laminin-treated coverslips. Neurons were maintained at 37 °C in a 5% CO_2_ humid incubator with complete DRG culture media consisting of DMEM supplemented with 10% FCS, 100 U ml^−1^ penicillin, 100 g ml^−1^ streptomycin, 10 ng ml^−1^ nerve growth factor and 20 ng ml^−1^ brain-derived neurotrophic factor. DRG neurons were used after 20–36 h in culture.

Before each experiment, coverslips were incubated for 2 min with compound **9** freshly prepared in pH 9 bath solution and then 10 min with Con A solution, following the same procedure described above for cell lines at a concentration of 1, 3, 6 and 24 μM of compound **9**.

Whole-cell currents were recorded in DRG neurons, while holding membrane voltage at −60 mV using a List EPC-7 amplifier (HEKA) and pClamp software (AXON Instruments). Currents were filtered at 1 kHz (2-pole Butterworth filter, −12 dB/octave) and transferred at a sampling rate of 10 kHz to a personal computer for analysis and display purposes using Clampfit software (AXON Instruments). Borosilicate glass pipettes were used with resistance of 2–5 MΩ and series resistance was compensated by 60–80%. The pipette solution contained the following (in mM): 137 cesium methanosulfonate, 10 CsCl, 0.3 EGTA, 10 NaCl and 10 HEPES pH 7.4. The bath solution for neurons was composed of (in mM): 140 NaCl, 2.5 KCl, 1.8 CaCl_2_, 1 MgCl_2_, 10 HEPES and 15 glucose pH 7.4. To activate GluK1 in control conditions, a bath solution with 10 mM of glutamate was applied for 500 ms using fast local perfusion. Cells were rapidly perfused using a linear array of eight glass tubes placed 200–300 μm from the cell body. Control and glutamate solutions flowed from adjacent barrels and solution changes were achieved by displacing the whole perfusion array laterally using a motorized device controlled by a personal computer^41^. For light stimulation, current was acquired using a free-run protocol using pClamp.Violet and green illumination of the focused field was done with arc lamp mounted on the microscope and switching between violet and green excitation filter, respectively.

### Photoresponse in degenerated retina

Extracellular recordings were obtained from RG cell populations in the isolated mouse retina using an array of one hundred 1.5-mm-long electrodes (inter-electrode distance=400 μm)^42^,^43^,^44^,^45^. Briefly, after enucleation of the eye the eyeball was hemisected with a razor blade, and the cornea and lens were separated from the posterior half under dim red illumination. The retinas were then carefully removed from the remaining eyecup with the pigment epithelium and placed on a glass lens. Each retina was incubated with 24 μM of freshly prepared TPC **9** in a volume of 500 μl in pH 9 (2% DMSO) during 1 min, after which the solution was removed and the incubation repeated with fresh compound for two more times. After that, retinas were washed three times in 500 μl Ringer medium and kept in Ringer medium until used for recordings. A retina was then mounted on a thick agarose gel (1.5% in Ringer medium) ganglion cell side up and covered with a Millipore filter with a rectangular opening for exposure of the retina to the recording electrodes array. This preparation was then mounted on a recording chamber, superfused with warm (36–37 °C) Ringer medium and dark adapted for 30 min. Ringer medium included (in mM): 124 NaCl, 2.5 KCl, 2 CaCl_2_, 2 MgCl_2_, 1.25 NaH_2_PO_2_, 26 NaHCO_3_ and 22 glucose. Although stable recordings could be made using this preparation in retinas that had been isolated for hours, the retinas used in these experiments were typically limited to 4 h post isolation.

Photostimulation was done by illumination of the entire retina with green (532 nm, 50 mW) and violet (405 nm, 50 mW) lasers (Lazerer Electronic Technology Co. Ltd). The incident light intensity was 23 mW cm^−2^ for the 532-nm light (green) and 8 mW cm^−2^ for the 405-nm light (violet). Illumination at 405 nm wavelength induces 80% of the maximal photocurrent, Fig. 3h. A typical multi-electrode array stimulation protocol consisted of six cycles of alternating 11-s 532-nm light stimulation and 11-s 405-nm light stimulation.

The electrode array was connected to a 100-channel amplifier (low and high corner frequencies of 250 and 7500 Hz) and a digital signal processor-based data acquisition system. All the selected channels of data, as well as the state of the visual stimulus, were digitized with a resolution of 16 bits at a sampling rate of 30 kHz with a commercial multiplexed A/D board data acquisition system (Bionic Technologies, Inc.) and stored digitally. Neural spike events were detected by comparing the instantaneous electrode signals with level thresholds set for each data channel using standard procedures described elsewhere^45^,^46^,^47^. When a supra-threshold event occurred, the signal window surrounding the event was time-stamped and stored together with the state of the visual stimulus for later offline analysis. Single unit classification was accomplished with an unsupervised principal component analysis method^42^,^46^. All resulting waveforms were reviewed and appropriate assignment of individual waveforms to distinct cells was confirmed further by analysis of the corresponding spike trains. Time stamps for action potentials of each sorted unit were used to generate Interspike interval histograms, peristimulus time histograms and peristimulus spike rasters, and for autocorrelation and cross-correlation analysis using NeuroExplorer (version 4) as well as customized software^42^. To normalize light-elicited changes in firing rate of individual RG cells, we calculated the LRI. Following ref. 5, this index was defined as: LRI=(mean firing rate in the violet light–mean firing rate in green light)/(mean firing rate in violet light+mean firing rate in green light).

A total of seven mice (control, *n*=3; treated, *n*=4) were tested at 9 months of age. The final number of analysed cells was 101 in untreated/control retinas and 159 in *rd10* retinas.

All reagents are from Sigma-Aldrich, unless otherwise specified. All *n* not specified on the text correspond to number of cells.

### Data availability

The data that support the findings of this study are available from the corresponding authors upon request.

## Additional information

**How to cite this article**: Izquierdo-Serra, M. *et al*. Optical control of endogenous receptors and cellular excitability using targeted covalent photoswitches. *Nat. Commun.* 7:12221 doi: 10.1038/ncomms12221 (2016).

## Supplementary Material

Supplementary Information Supplementary Figures 1-42, Supplementary Tables 1-6, Supplementary Note 1, Supplementary References
