# Supplementary material for: Optical control of endogenous receptors and cellular excitability using targeted covalent photoswitches
Source: Nat Commun. 2016 Jul 20;7:12221. doi: 10.1038/ncomms12221 (PMC4961765; doi:10.1038/ncomms12221)
Supplement: Supplementary Information — Supplementary Figures 1-42, Supplementary Tables 1-6, Supplementary Note 1, Supplementary References [file ncomms12221-s1.pdf]

Supplementary Figure 1. Neurotransmitter and amino acid structures

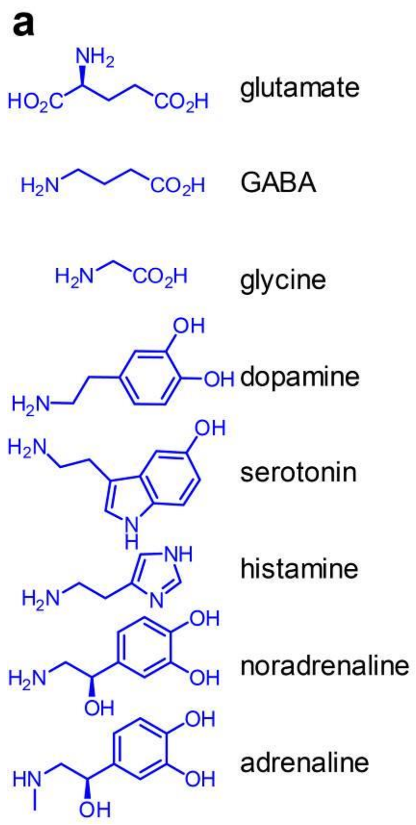

(**a**) Neurotransmitters to be used as ligands in neuronal receptor PTLs contain amine and/or hydroxyl moieties susceptible of reacting with an electrophilic reactive group. (**b**) Structure of nucleophilic amino acids (protein side chains) targeted by electrophilic reactive groups.

Supplementary Figure 2. Optimization of the Click Reactions conditions

Conditions A (Cu_2O and NaAsc): to a solution of azide (compounds **1** or **2**, 1 mg, 1 equiv), NaAsc (4 equiv), Cu_2O (2.4 equiv) in water (30 μL) was added a solution of 4-pentynoic acid (1.1 equiv) in THF (4 μL) and the reaction was stirred at RT. The conversion of the reaction was determined by HPLC.

Conditions B (Cu_2O): To a solution of azide (compounds **1** or **2**, 1 mg, 1 equiv), Cu_2O (2.4 equiv) in water (30 μL) was added a solution of 4-pentynoic acid (1.1 equiv) in THF (4 μL) and the reaction was stirred at rt. The conversion of the reaction was determined by HPLC.

Conditions C ($CuSO_4$ and NaAsc): To a solution of azide (compounds **1** or **2**, 1 mg, 1 equiv), NaAsc (4 equiv), $CuSO_4$ (2.4 equiv) in water (30 μL) was added a solution of 4-pentynoic acid (1.1 equiv) in THF (4 μL) and the reaction was stirred at rt. The conversion of the reaction was determined by HPLC.

HPLC analyses were performed on an Alliance 2695 system with UV detection at 360 nm using a ZORBAX Eclipse Plus C18 (4.6 mm × 75 mm, 3.5 μm) column under the following chromatography conditions: mobile phase A, H_2O with 0.2% HCO_2H; mobile phase B, CH_3CN with 0.2% HCO_2H; flow rate, 1.0 mL min^{-1}; injection volume, 5 μL; elution gradient, 0.0−5.0 min, 5−90% B; 5.0−7.0min, 90% B; 7.0−8.0 min, 90−100% B; 8.0−10.0 min, 100% B; 10.0−11.0 min, 100−5% B; 11.0−15.0 min, 5% B.

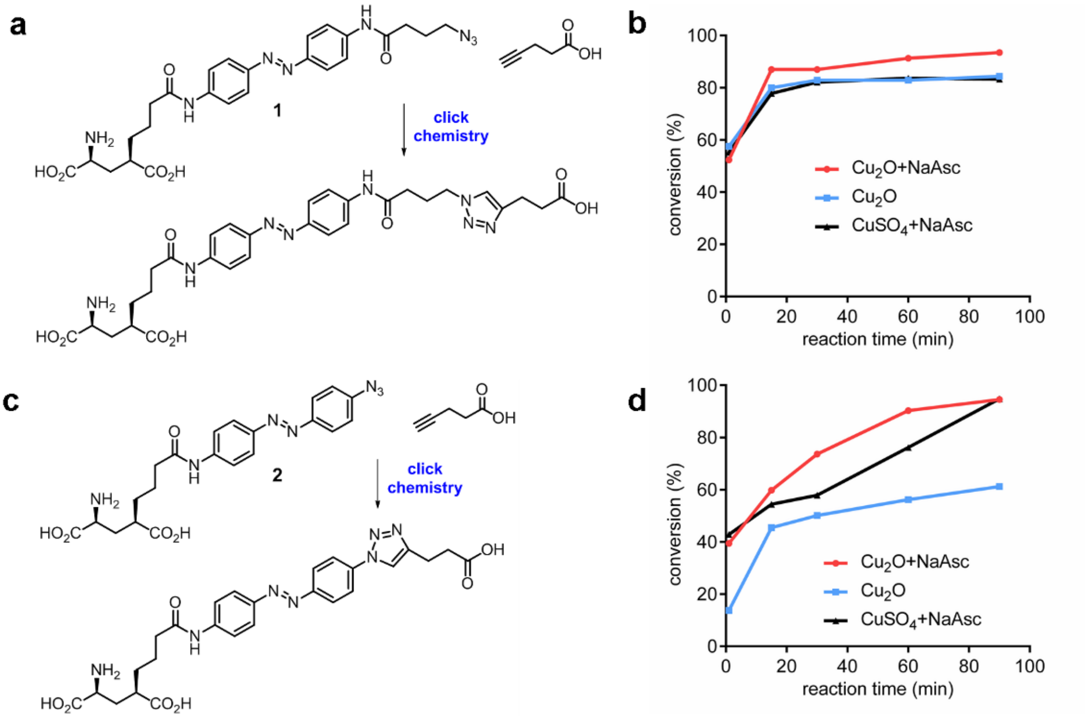

Progression of click reaction monitored by HPLC (see text above section for details). **(a)** Scheme of the reaction between azide **1** and 4-pentynoic acid. **(b)** Percentage of azide **1** conversion versus reaction time under different reaction conditions. **(c)** Scheme of the reaction between azide **2** and 4-pentynoic acid. **(d)** Percentage of azide **2** conversion versus reaction time under different reaction conditions.

Supplementary Figure 3. Structures of Compounds Obtained by Click Reaction with azide 1

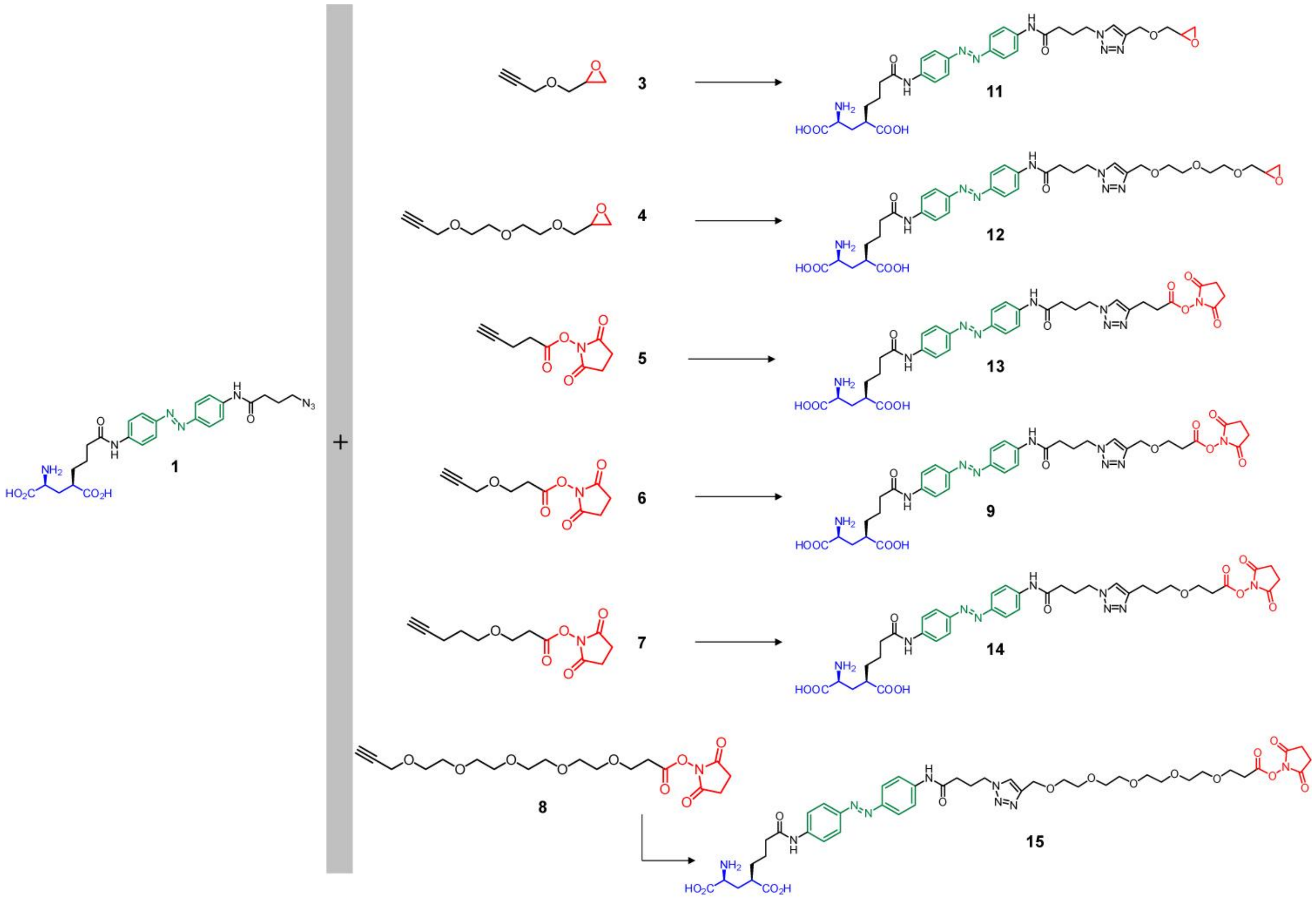

Supplementary Figure 4. Structures of Compounds Obtained by Click Reaction with azide 2

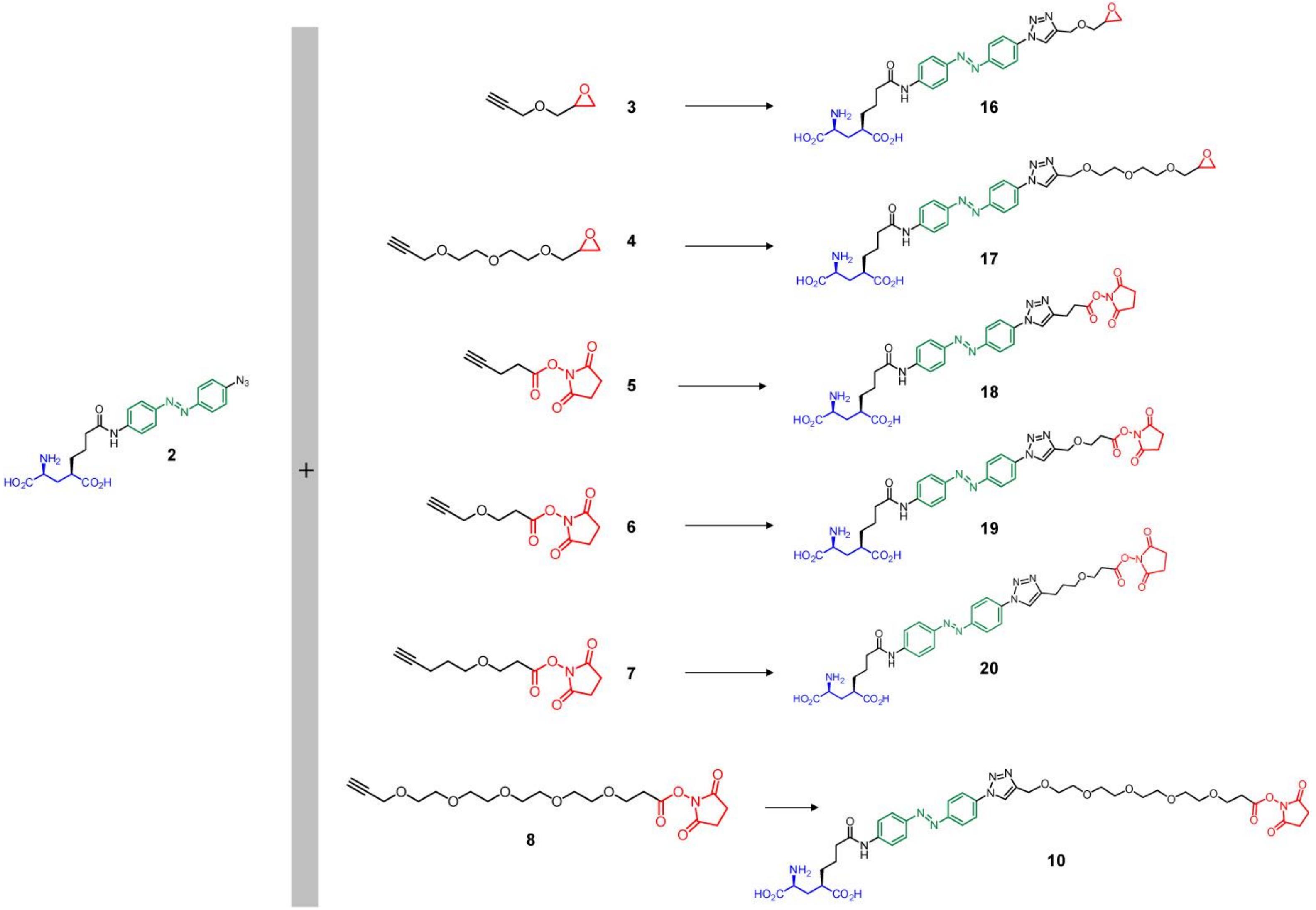

Supplementary Figure 5. Generation of compound 9 and side product characterization by UPLC-MS analysis

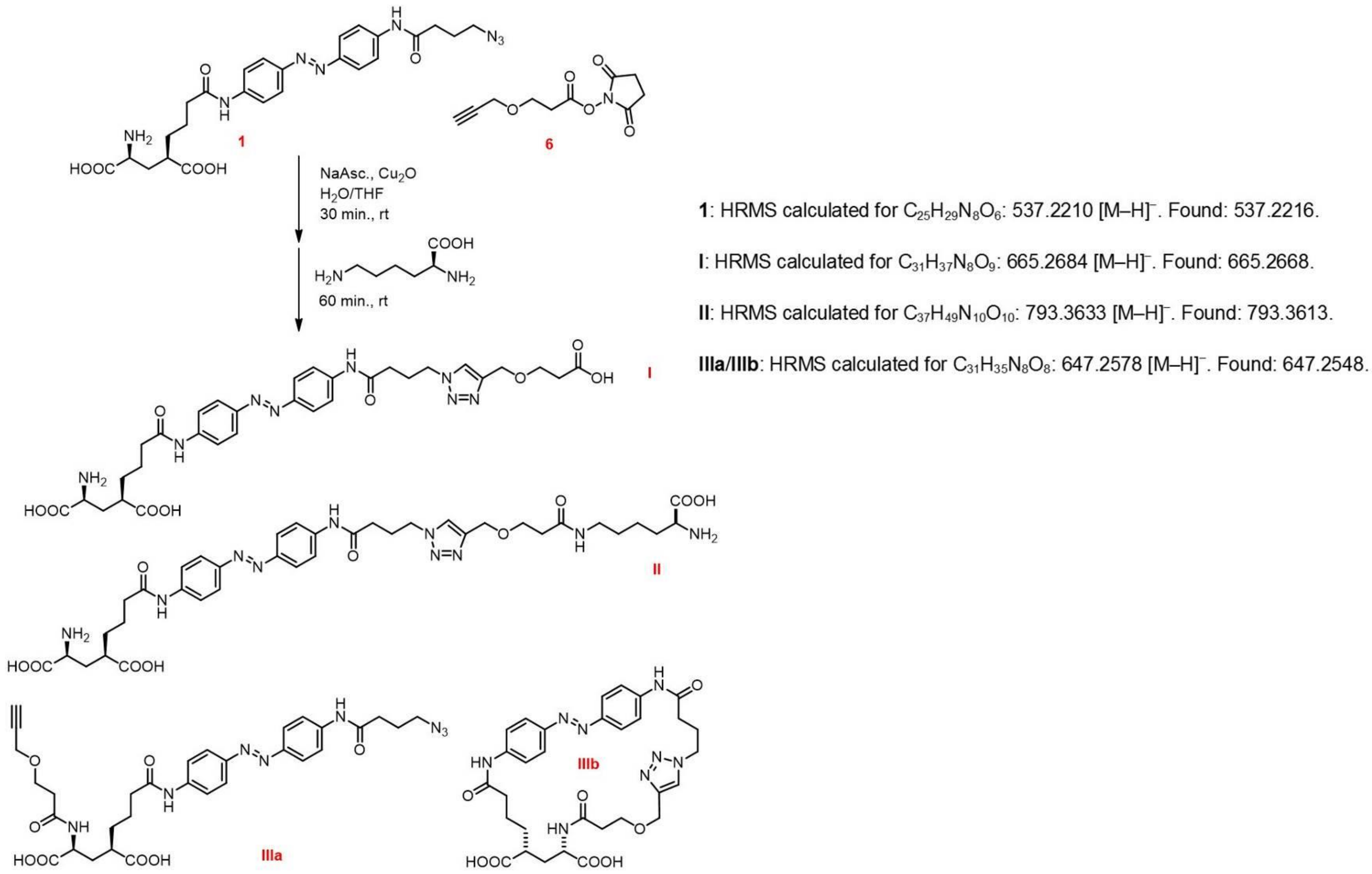

1: HRMS calculated for $C_{25}H_{29}N_8O_6$: 537.2210 [M–H]$^-$. Found: 537.2216.

I: HRMS calculated for $C_{31}H_{37}N_8O_9$: 665.2684 [M–H]$^-$. Found: 665.2668.

II: HRMS calculated for $C_{37}H_{49}N_{10}O_{10}$: 793.3633 [M–H]$^-$. Found: 793.3613.

IIIa/IIIb: HRMS calculated for $C_{31}H_{35}N_8O_8$: 647.2578 [M–H]$^-$. Found: 647.2548.

Scheme of the reaction between azide **1** and NHS-alkyne **6**

Supplementary Figure 6. Generation of compound 9 and side product characterization by UPLC-MS analysis

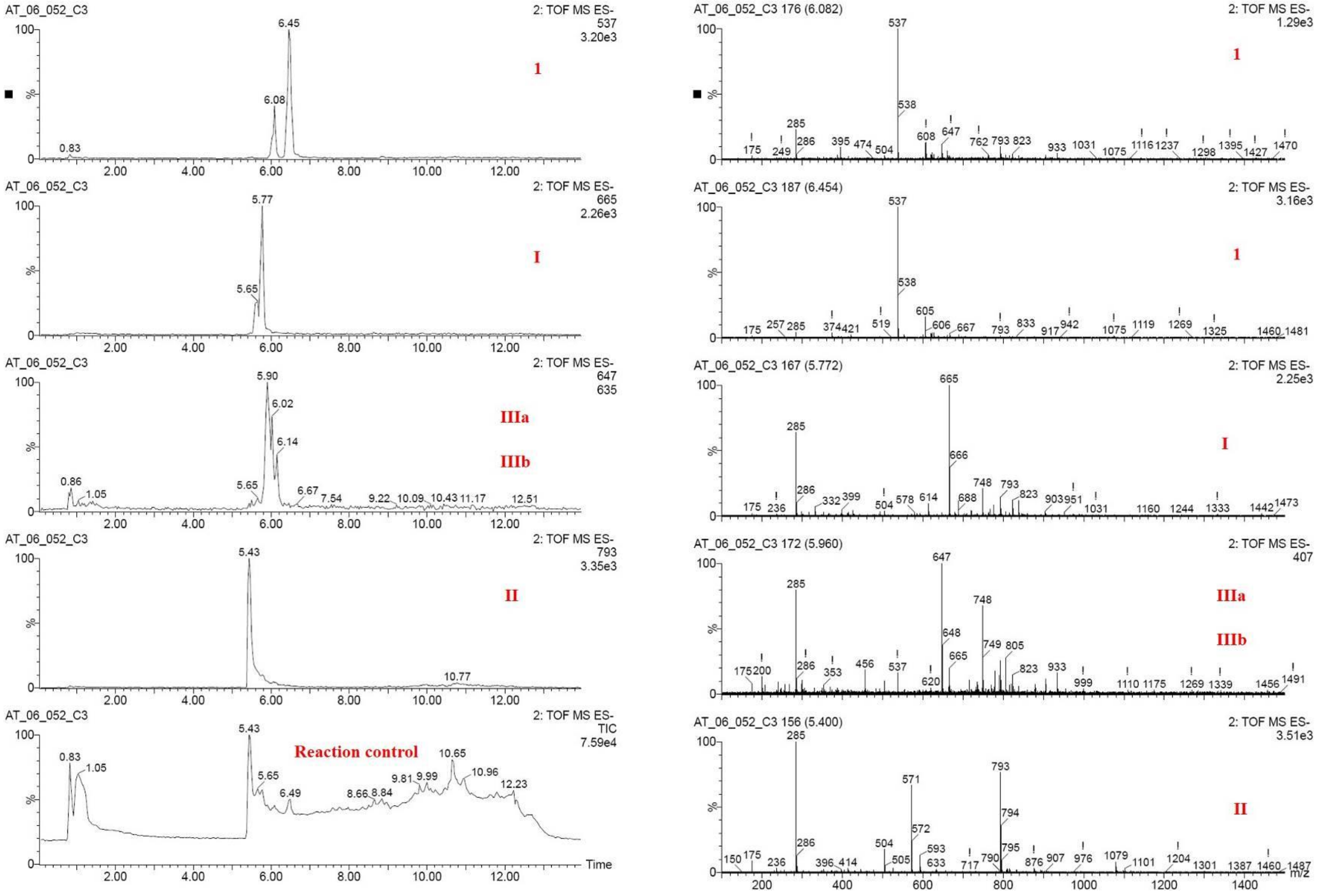

UPLC-MS analysis of the reaction mixture from **Supplementary Figure 5** at 60 min after the addition of lysine

Supplementary Figure 7. Generation of compound 10 and side product characterization by UPLC-MS analysis

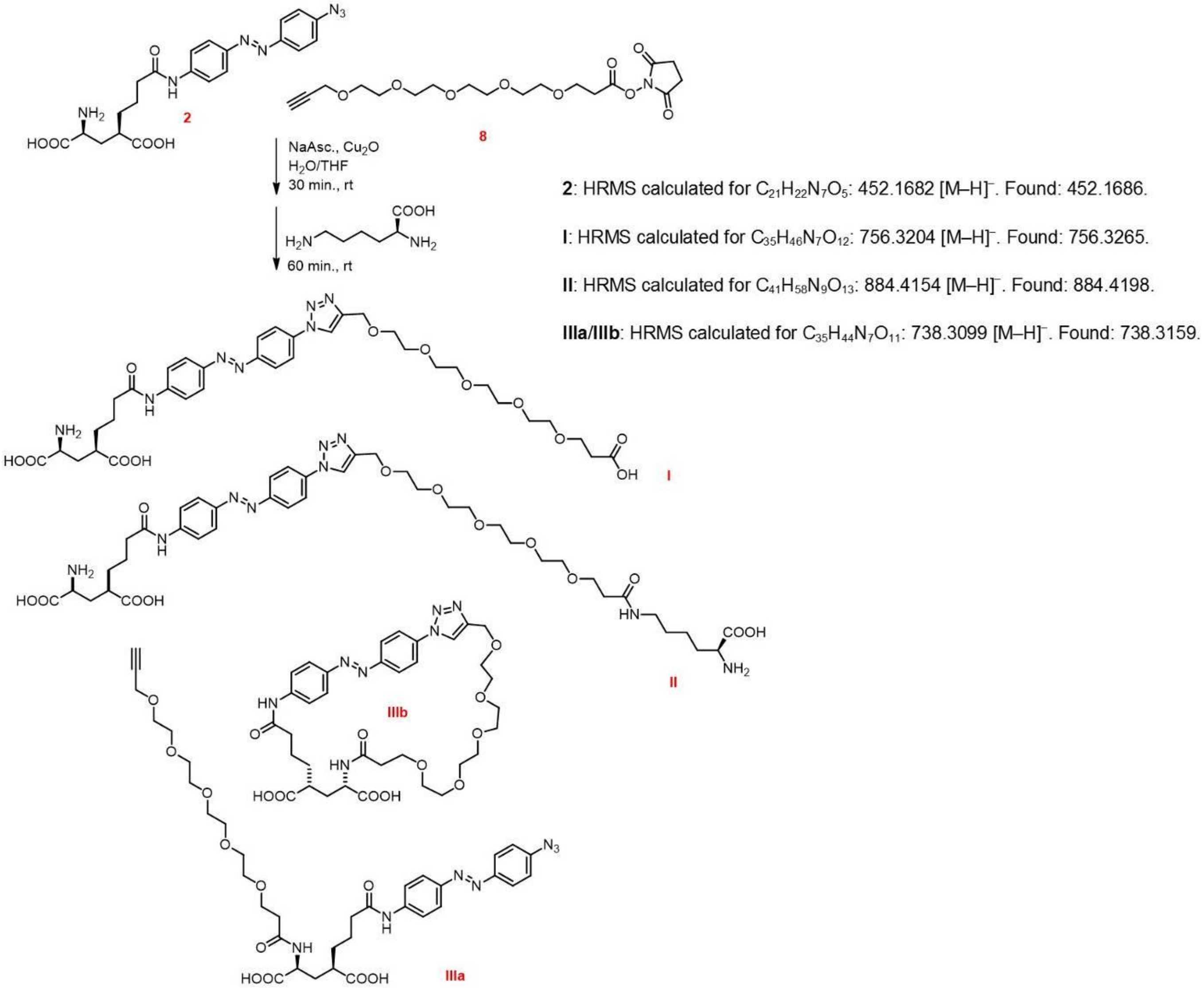

Scheme of the reaction between azide **2** and NHS-alkyne **8**.

Supplementary Figure 8. Generation of compound 10 and side product characterization by UPLC-MS analysis

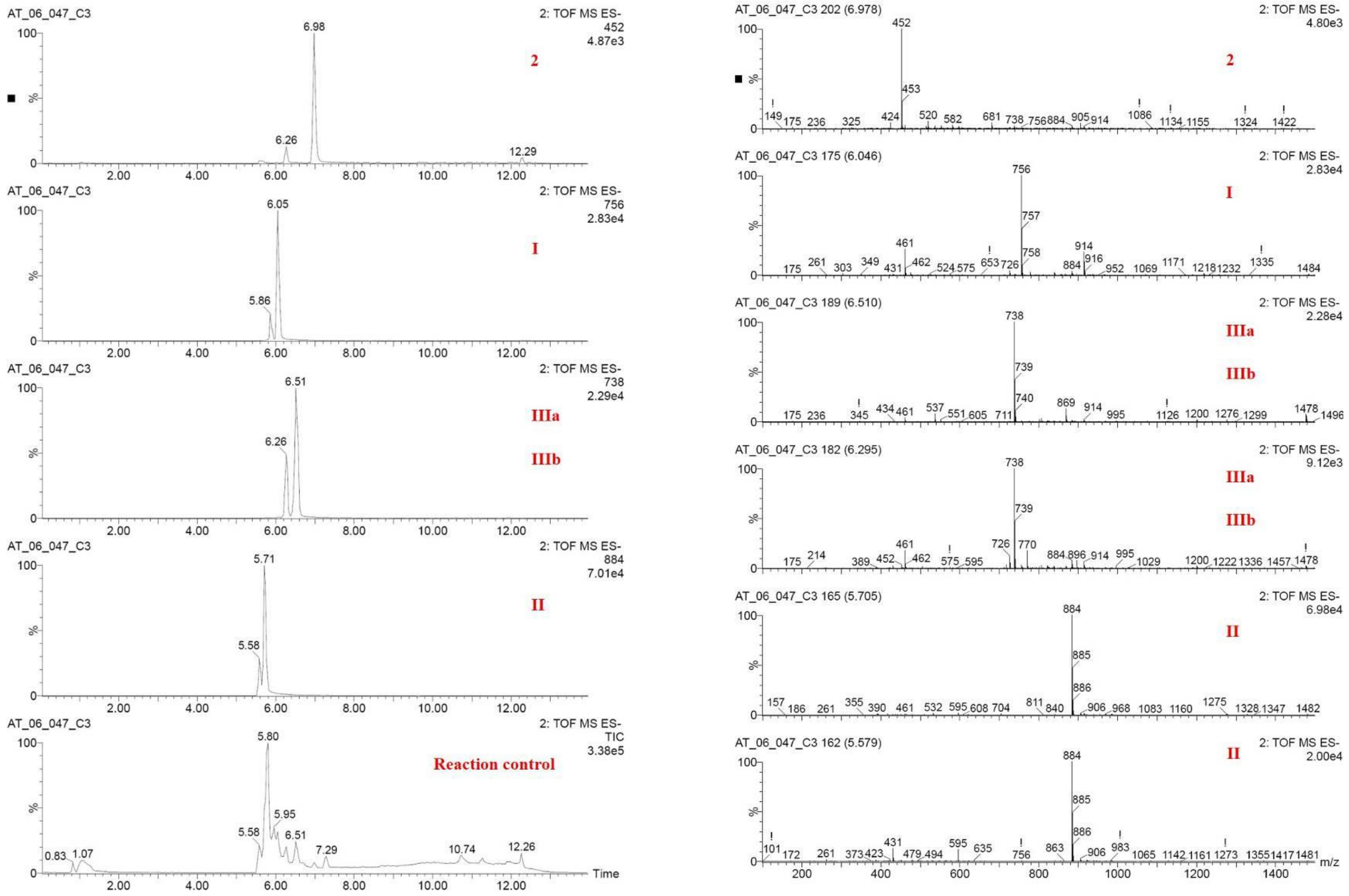

UPLC-MS analysis of the reaction mixture from **Supplementary Figure 7** at 60 min after the addition of lysine

Supplementary Figure 9. Generation of compound 11 and HPLC analysis.

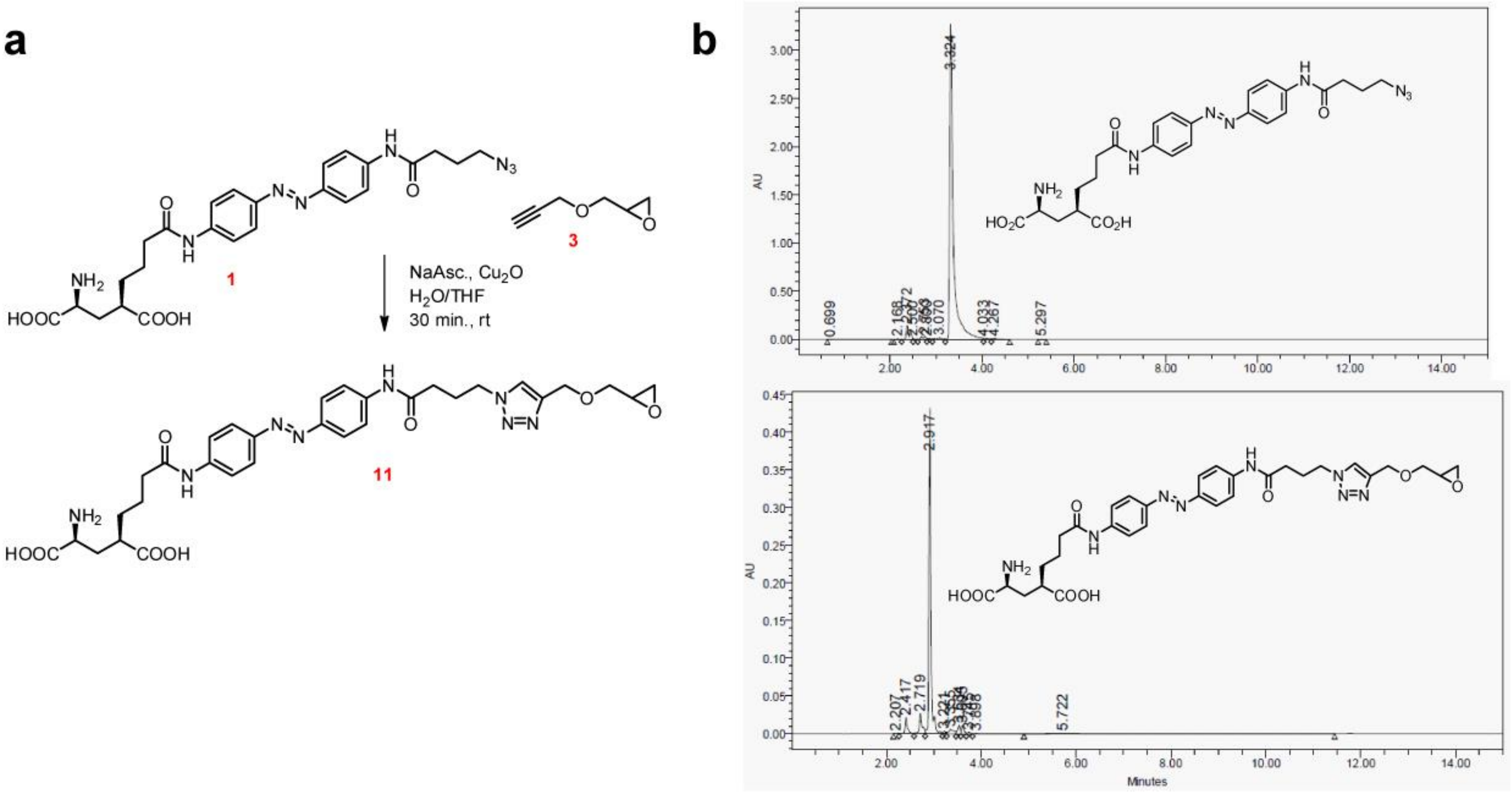

(**a**) Scheme of the reaction between azide **1** and epoxide-alkyne **3** to yield **11**. (**b**) HPLC chromatograms of azide **1** and reaction mixture at 30 min

Supplementary Figure 10. Generation of compound 12 and UPLC-MS analysis

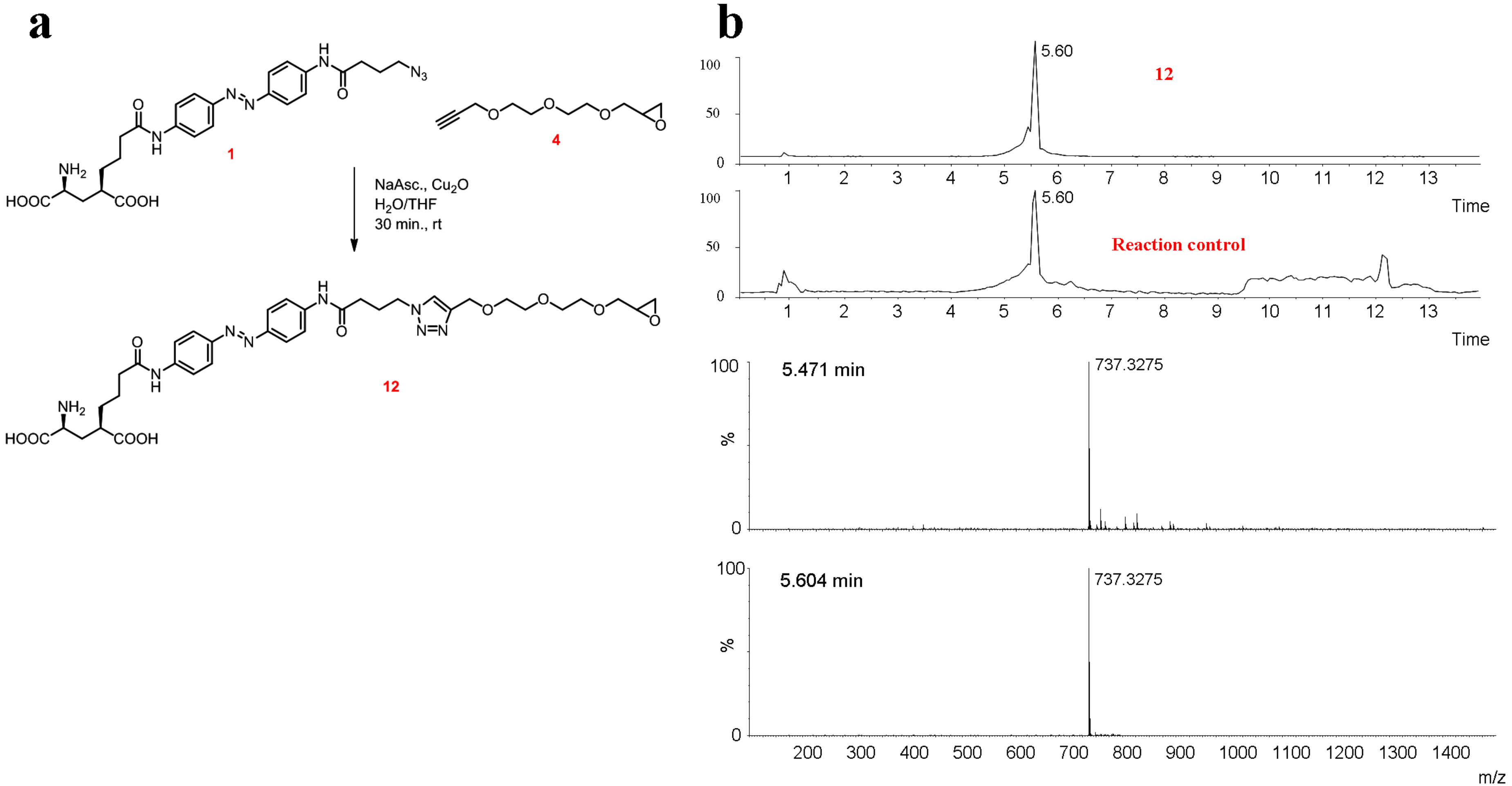

12: HRMS calculated for $C_{35}H_{45}N_8O_{10}$: 737.3259 $[M–H]^-$. Found: 737.3275

(**a**) Scheme of the reaction between azide **1** and epoxide-alkyne **4** to yield **12**. (**b**) UPLC-MS analysis of the reaction mixture at 30 min

Supplementary Figure 11. UV-Visible absorption spectra and photochromism of compounds 9 and 10.

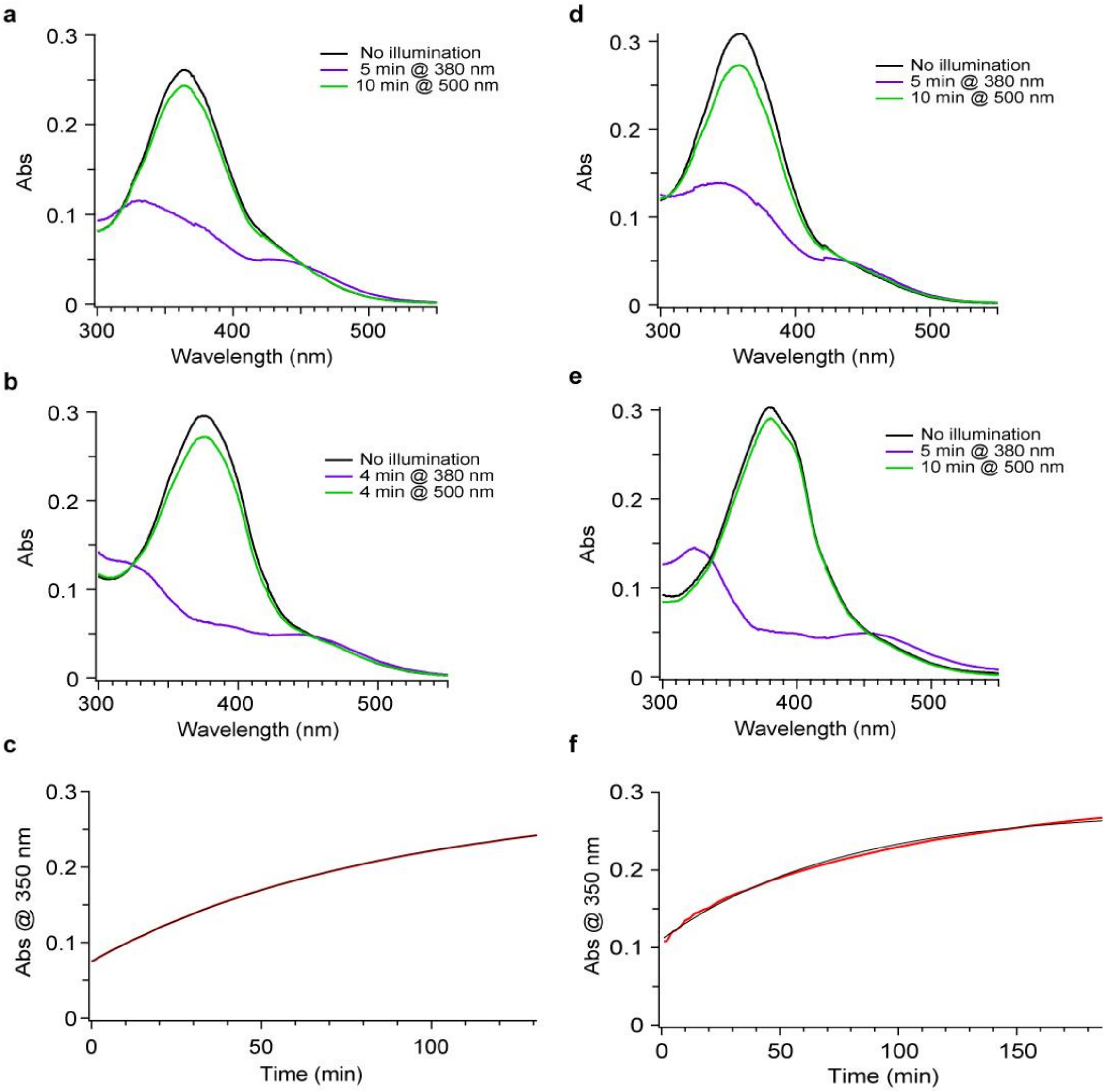

(**a-b** and **d-e**) Absorption spectrum of non-illuminated compounds (black), after 5 min of illumination at 380 nm (violet) and after 5 min at 500 nm (green). (**a**) Absorption spectra of compound **9** (15 µM) in 1% DMSO and 99% PBS and (**b**) in 100% DMSO. (**c**) Time course of the absorption at 350 nm of compound **9** (15 µM, 25 ºC) in the dark after 5 min of illumination with 380 nm light. The calculated relaxation lifetime is 84.07 min. (**d**) Absorption spectra of compound **10** (15 µM) in 1% DMSO and 99% PBS and (**e**) in 100% DMSO. (**f**) Time course of the absorption at 350 nm of compound **10** (15 µM, 25 ºC) in the dark after 5 min of illumination with 380 nm light. The relaxation lifetime of compound **10** (15 µM) is 74.82 min. For absorbance measurements, we used compounds **9** and **10** previously prepared and immediately stored in 100% DMSO at -20 ºC.

Supplementary Figure 12. Epoxide-based PTLs assayed on GluK1.

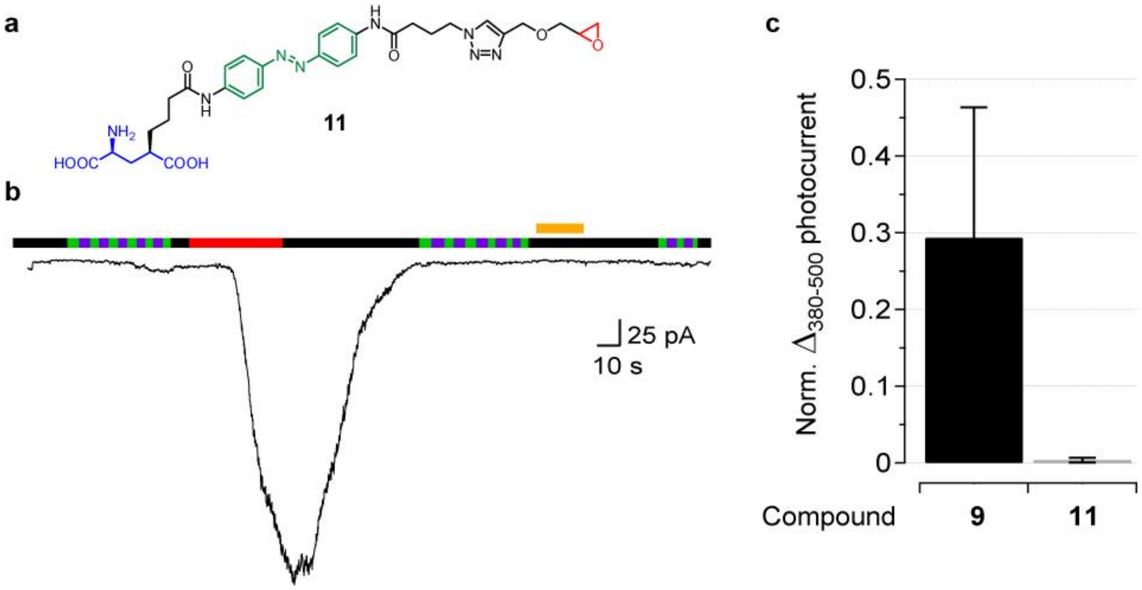

(**a**) Structure of compound **11** bearing an epoxide moiety as a reactive group (in red) (**b**). Whole-cell current from HEK293 cells expressing GluK1 and incubated with **11**. Green bar indicates 500 nm illumination, and violet 380 nm. Red bars indicate when the bath solution perfused contained 300 µM glutamate and yellow 1 mM DNQX. (**c**) Quantification of the variation of photocurrent for cells incubated with compound **11**, and compound **9** as a positive control for light activation. Photocurrent is normalized by current induced by perfusion of glutamate. Bars are mean ± s.e.m, n=5 for compound **9** and n=6 for compound **11**.

Supplementary Figure 13. Basal *(trans)* photocurrents.

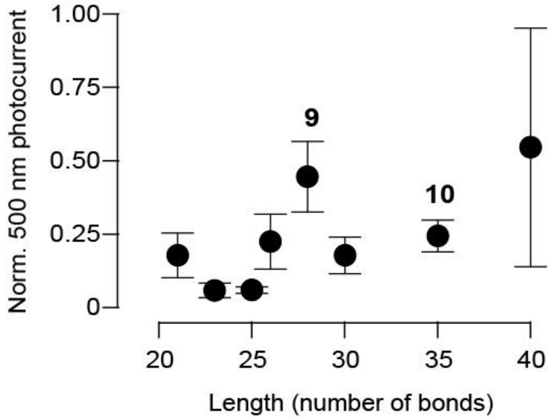

Current induced by the *trans* conformation of the compounds versus its length, in number of bonds from the reactive carbonyl group to the *C*-4 of glutamate (see **Supplementary Table 1**). Compounds **9** and **10** have a length of 28 and 35 bonds, respectively. Data points are indicated as mean ± s.e.m, equal n’s as in **Figure 3c**.

Supplementary Figure 14. Action spectra traces

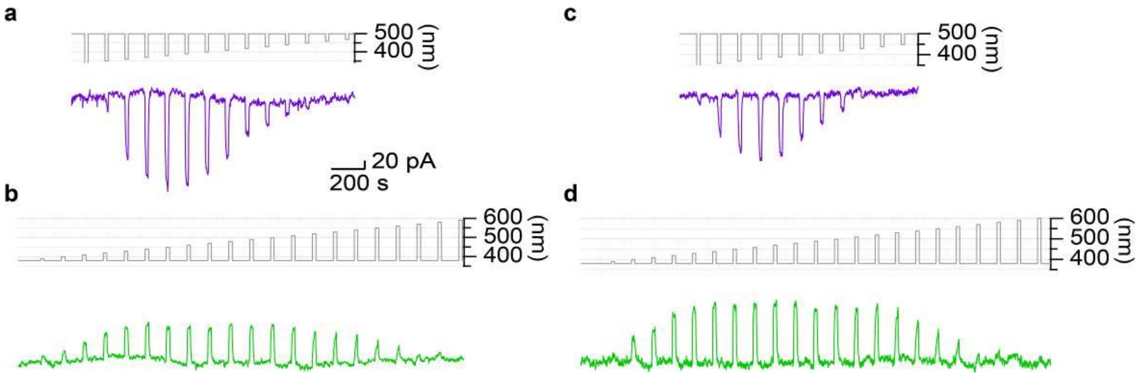

(**a-d**) Whole cell recordings after HEK239 cells expressing GluK1 were incubated with compounds **9** or **10**. (**a** and **c**) Representative activation spectra of compound **9** (**a**) and **10** (**c**) assayed by measuring photocurrent in response to light pulses ranged from 350 to 470 nm, while resting wavelength was set to 500 nm (maximal deactivation). (**b** and **d**) Representative deactivation spectra of compound **9** (**b**) and **10** (**d**) assayed by measuring photocurrent in response to light pulses ranged from 380 to 600 nm, while resting wavelength was set to 380 nm (maximal activation). Quantification is shown in **Figure 3h**.

Supplementary Figure 15. PTL is a full agonist.

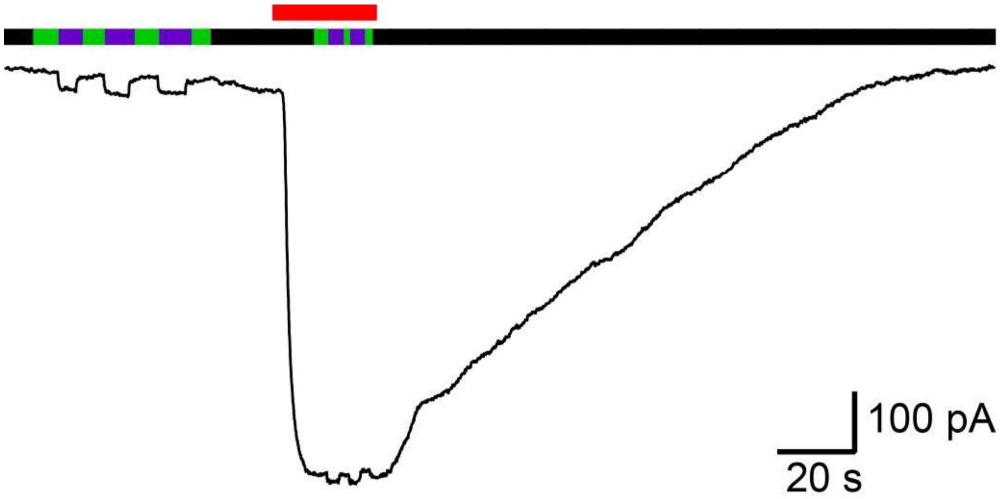

Whole cell current recording from a HEK293 cell expressing GluK1 and incubated with compound **9**. We compared binding of a full agonist (red bar, 300 µM glutamate) and binding of the PTL by photoswitching between the activating and deactivating configurations (violet and green bars, respectively). The binding of the PTL under violet light leads to higher activation of the receptor than free glutamate, indicating that the compound acts as full agonist[6].

Supplementary Figure 16. Photosensitivity

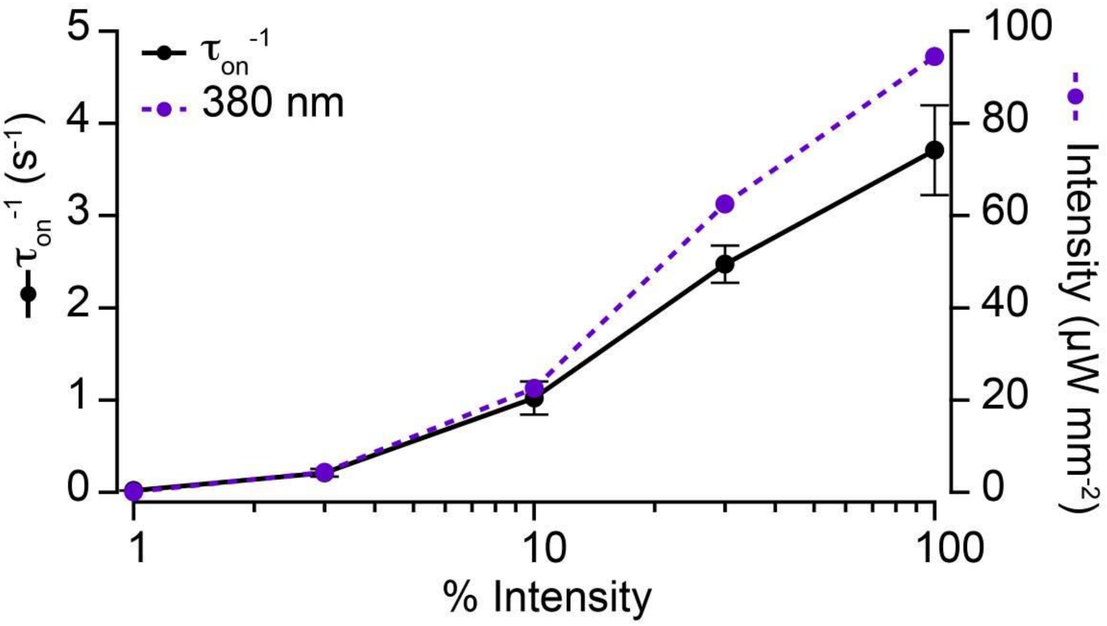

Photosensitivity of compound **9** was determined from the dependence of τ_{on} of the photocurrent with light intensity. In HEK293 cells expressing GluK1 and incubated with compound **9**, photocurrents were elicited at different intensities of violet light (purple trace, right axis). To calculate τ_{on}^{-1}(black trace, left axis.), the photocurrent onset was fitted to an exponential function. Dots are mean ± s.e.m, n=1 photocurrent for 0% intensity, n=2 photocurrents for 3% intensity and n=3 photocurrents for 10 to 100% intensity.

Supplementary Figure 17. Mass spectrometry characterization of purified intact S1S2 GluK1 domain protein.

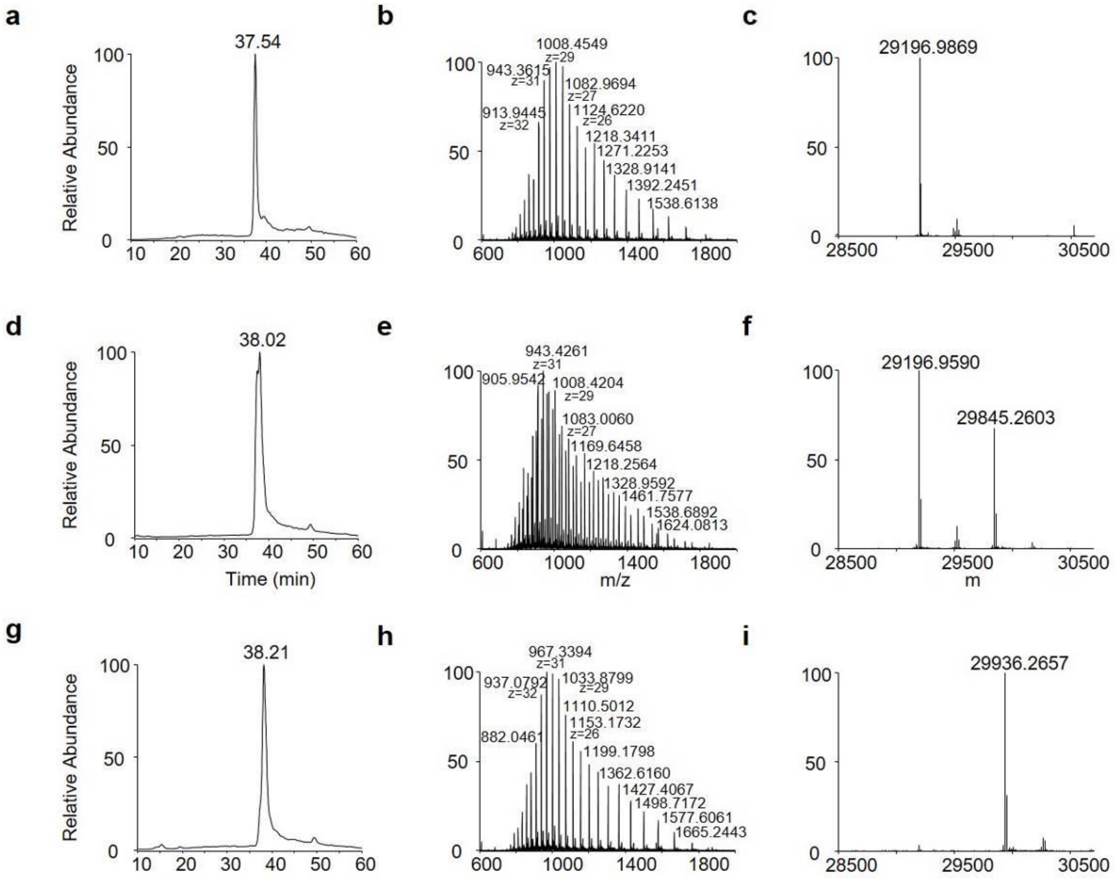

Mass spectrometry characterization of purified intact S1S2 GluK1 domain protein (**a**, **b**, **c**) and after conjugation with compound **9** (**d**, **e**, **f**) and compound **10** (**g**, **h**, **i**). Total ion current chromatograms of intact protein (**a**) and after conjugation to compound **9** (**d**) and compound **10** (**g**). Mass spectra of intact protein (**b**) and after conjugation to compound **9** (**e**) and compound **10** (**h**). The deconvoluted monoisotopic mass spectra of the protein prior to conjugation (**c**) displays a single peak corresponding to the intact protein (theoretical mass 29196.8892 Da, mass error 3.35 ppm). The deconvoluted monoisotopic mass spectra of the protein conjugated to compound **9** (**f**) displays two peaks corresponding to the unconjugated (mass 29196.9590) and conjugated protein (mass 29845.2603). The deconvoluted monoisotopic mass spectra of the protein conjugated to compound **10** (**i**) displays a single peak that demonstrates almost complete conjugation (theoretical mass 29936.2069 Da, mass error 1.96 ppm). Results in (**f**) and (**i**) indicate that the stoichiometric ratio of S1S2 GluK1 conjugation to compounds **9** and **10** is 1:1.

Supplementary Figure 18. PTL conjugation to S1S2 GluK1 domain protein and effect of illumination.

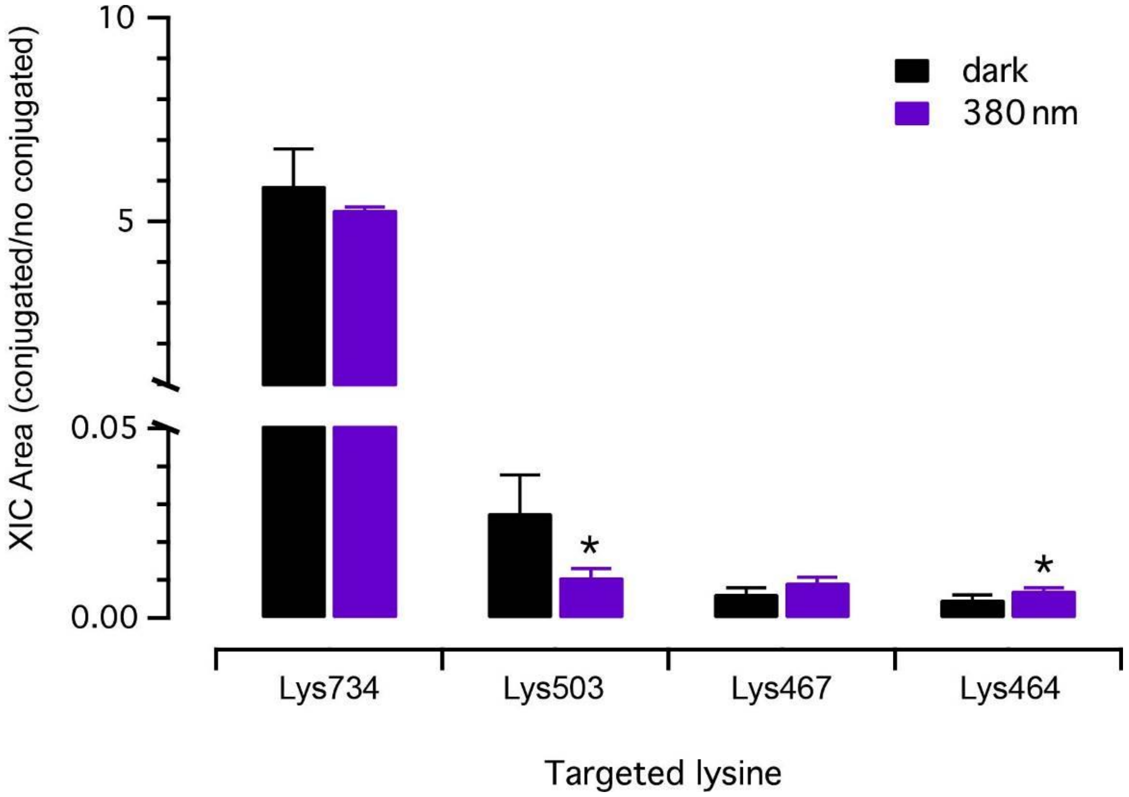

Relative quantification by targeted MS/MS analysis of compound **9** lysine conjugation in S1S2 GluK1 protein domain under 380nm illumination. For each conjugated lysine, ratio peak areas of the extracted ion chromatograms (XIC) of conjugated peptides vs non-conjugated peptides were represented for dark and violet illumination. Significance was calculated by a linear mixed effects model, * $p<0.05$. N = 3 conjugation experiments, bars are mean ± standard deviation.

Supplementary Figure 19. Photoswitchable activity of free, hydrolyzed PTL compounds.

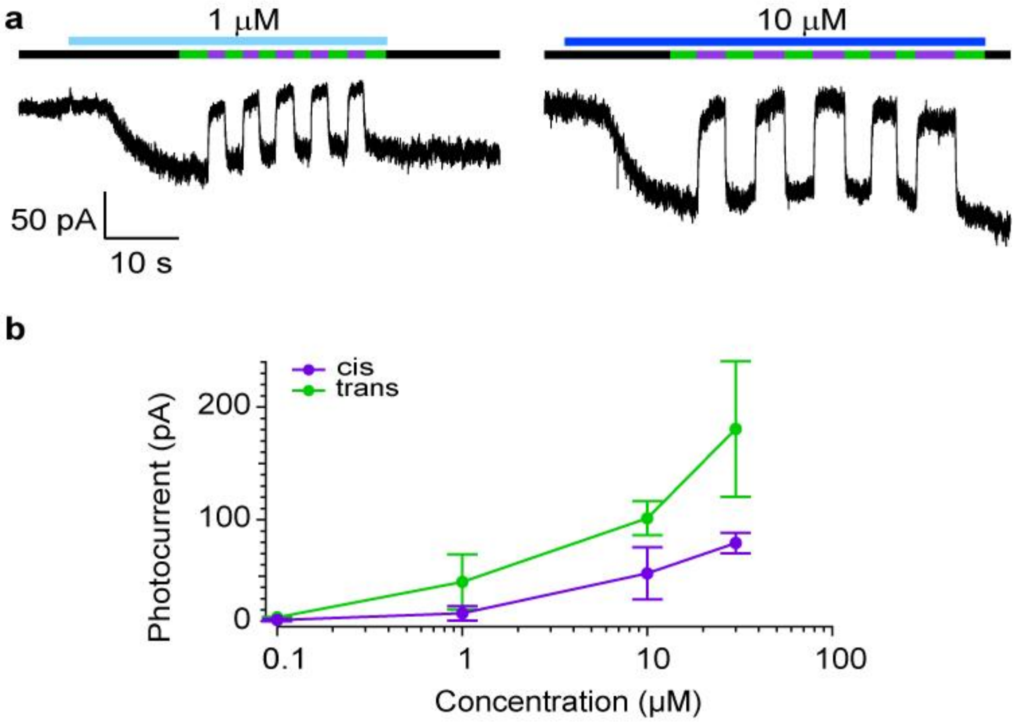

(**a**) Whole-cell current recording in HEK239 cells expressing GluK1 perfused with compound **10** at 1 μM (light blue bar) and 10 μM (dark blue bar). Light pulses are indicated in green (500 nm) and violet (380 nm), absence of light is colored in dark. (**b**) Quantification of the photoresponse obtained in *cis* and *trans* conformations when compound **10** was perfused at 0.1 μM (n=2), 1 μM (n=3), 10 μM (n=4) and 30 μM (n=3). Points are mean ± s.e.m.

Supplementary Figure 20. Photoresponses of GluK1 Lys734Ala-Lys497Ala mutant

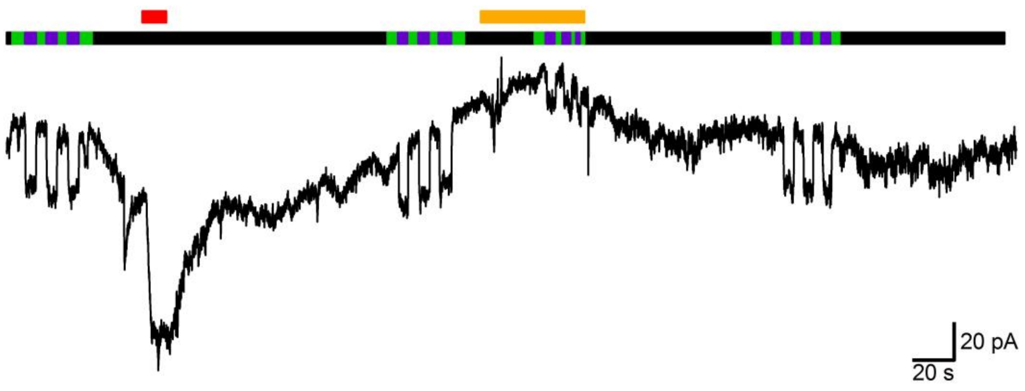

Whole cell current recording from a HEK cell expressing GluK1-Lys734Ala-Lys497Ala.

We mutated to alanine (which does not bear nucleophilic reactive groups) the main lysine involved in conjugation (Lys734Ala, see **Fig. 4** and **Supplementary Figs. 17** and **18**) as well as another residue located near the glutamate binding site that was identified as a partial conjugation site in preliminary assays (Lys497Ala). The double mutant Lys734Ala-Lys497Ala expresses at a similar level to the wildtype, but after conjugation to compound **9** photoresponses are not abolished in the absence of these two lysines. This result suggests that one or more of the 18 remaining lysines exposed on the surface of the GluK1 LBD (**Fig. 4f**) can act as surrogate conjugation sites of compound **9** in the absence of Lys497 and Lys734. Red and orange marks indicate glutamate and DNQX perfusion respectively; green, violet and black marks correspond to illumination at 500 nm, 380 nm and absence of illumination.

Supplementary Figure 21. Photocurrents in DRG neurons

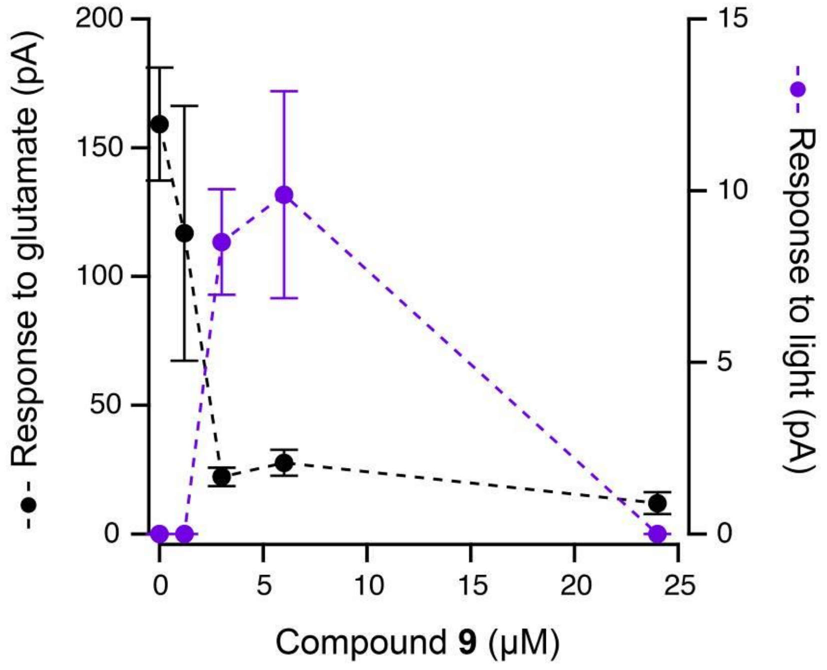

Quantification of current responses induced by glutamate (black dots, left axis) and light (violet dots, right axis) in DRG neurons incubated with 0, 1, 3, 6 or 24 µM of compound **9** and Con A, previous to the experiment. Incubation with increasing concentration of compound **9** reduces response to glutamate and increases photoresponses. We determined an optimal concentration window (3 to 6 uM of compound **9**) in which photoresponses are maximal and glutamate response is still present. Dots are mean ± s.e.m, for glutamate responses n ranges from 6 to 21, for photoresponses: n=7 for incubation with 3 µM of compound **9** and n=5 for incubation with 6 µM of compound **9**.

Supplementary Figure 22. Retinal photoresponses are stable in time

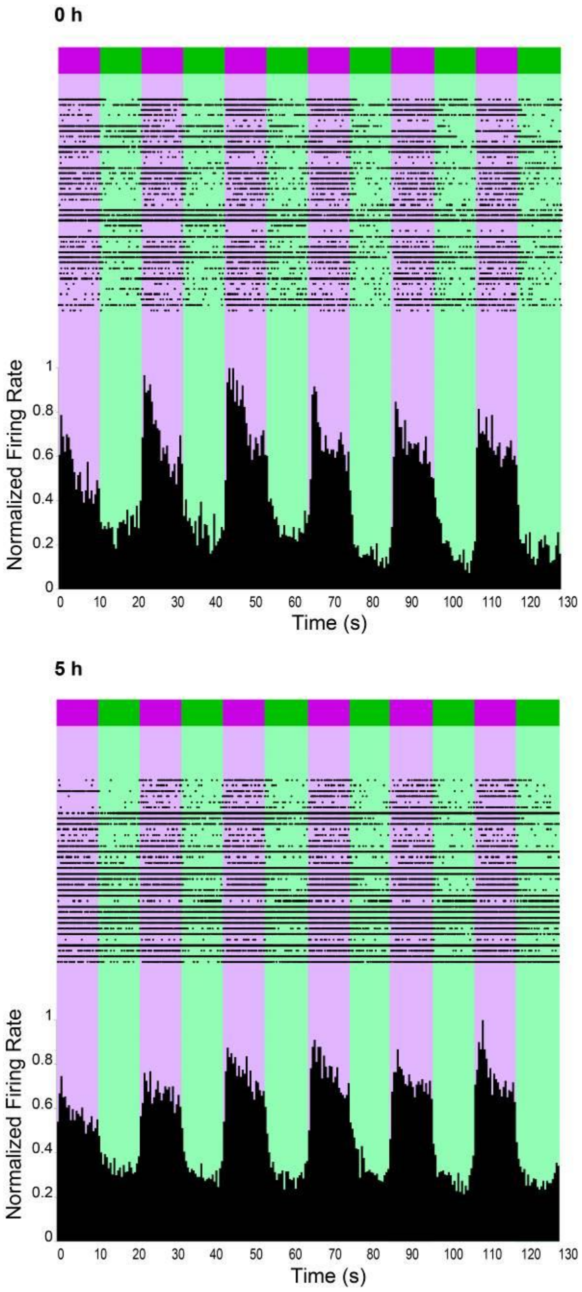

Raster plot and integrated time course of the firing rate of MEA from flat-mounted degenerated retinas from *rd10* mice incubated with compound **9**. On top, photoresponse of a retina right after the incubation with the TCP. On bottom, retinal photoresponses are still observed after 5 h of the TCP treatment.

Supplementary Figure 23. GluK1 ligand binding domain sequence alignment

```
                     GluK1 Ligand Binding Domain Segment 1

R.norvegicus         NRTLIVTTILEEPYVMYRKSDKPLYGNDRFEGYCLDLLKELSNILGFLYDVKLVPDGKYG 505
M.musculus           NRTLIVTTILEEPYVMYRKSDKPLYGNDRFEGYCLDLLKELSNILGFLYDVKLVPDGKYG 490
M.mulatta            NRTLIVTTILEEPYVMYRKSDKPLYGNDRFEGYCLDLLKELSNILGFIYDVKLVPDGKYG 505
P.troglodytes        NRTLIVTTILEEPYVMYRKSDKPLYGNDRFEGYCLDLLKELSNILGFIYDVKLVPDGKYG 505
H.sapiens            NRTLIVTTILEEPYVMYRKSDKPLYGNDRFEGYCLDLLKELSNILGFIYDVKLVPDGKYG 505
F.catus              NRTLIVTTILEEPYVMYRKSDKPLYGNDRFEGYCLDLLKELSNILGFIYDVKLVPDGKYG 490
C.lupus              NRTLIVTTILEEPYVMYRKSDKPLYGNDRFEGYCLDLLKELSNILGFIYDVKLVPDGKYG 505
S.scrofa             NRTLIVTTILEEPYVMYRKSDKPLYGNDRFEGYCLDLLKELSNILGFIYDVKLVPDGKYG 490
O.aries              NRTLIVTTILEEPYVMYRKSDKPLYGNDRFEGYCLDLLKELSNILGFIYDVKLVADGKYG 490
G.gallus             NRTLIVTTILEDPYVMYKKSDKPLYGNDRFEGYCLDLLKELSNILGFIYEVKLVSDGKYG 479
D.rerio              NRTLIVTTILENPYVMYKKSDKPLYGNDRFEGYCLDLLKELSNILGFSYEVKLVTDGKYG 530
                     ***********:*****:**************************** *:****.*****

R.norvegicus         AQNDKGEWNGMVKELIDHRADLAVAPLTITYVREKVIDFSKPFMTLGISILYRK 559
M.musculus           AQNDKGEWNGMVKELIDHRADLAVAPLTITYVREKVIDFSKPFMTLGISILYRK 544
M.mulatta            AQNDKGEWNGMVKELIDHRADLAVAPLTITYVREKVIDFSKPFMTLGISILYRK 559
P.troglodytes        AQNDKGEWNGMVKELIDHRADLAVAPLTITYVREKVIDFSKPFMTLGISILYRK 559
H.sapiens            AQNDKGEWNGMVKELIDHRADLAVAPLTITYVREKVIDFSKPFMTLGISILYRK 559
F.catus              AQNDKGEWNGMVKELIDHKADLAVAPLTITYVREKVIDFSKPFMTLGISILYRK 544
C.lupus              AQNDKGEWNGMVKELIDHKADLAVAPLTITYVREKVIDFSKPFMTLGISILYRK 559
S.scrofa             AQNDKGEWNGMVKELIDHKADLAVAPLTITYVREKVIDFSKPFMTLGISILYRK 544
O.aries              AQNDKGEWNGMVKELIDHKADLAVAPLTITYVREKVIDFSKPFMTLGISILYRK 544
G.gallus             AQNDKGEWNGMVKELIDHKADLAVAPLTITYVREKVIDFSKPFMTLGISILYRK 533
D.rerio              AQNDKGEWNGMVRELIDHVADLAVAPLTITYVREKVIDFSKPFMTLGISILYHK 584
                     ************:***** *********************************:*


                     GluK1 Ligand Binding Domain Segment 2

R.norvegicus         PIDSADDLAKQTKIEYGAVRDGSTMTFFKKSKISTYEKMWAFMSSRQQSALVKNSDEGIQ 741
M.musculus           PIDSADDLAKQTKIEYGAVRDGSTMTFFKKSKISTYEKMWAFMSSRQQSALVKNSDEGIQ 726
M.mulatta            PIDSADDLAKQTKIEYGAVRDGSTMTFFKKSKISTYEKMWAFMSSRQQTALVRNSDEGIQ 741
P.troglodytes        PIDSADDLAKQTKIEYGAVRDGSTMTFFKKSKISTYEKMWAFMSSRQQTALVRNSDEGIQ 741
H.sapiens            PIDSADDLAKQTKIEYGAVRDGSTMTFFKKSKISTYEKMWAFMSSRQQTALVRNSDEGIQ 741
F.catus              PIDSADDLAKQTKIEYGAVRDGSTMTFFKKSKISTYEKMWAFMSSRQQTALVKNSDEGIQ 726
C.lupus              PIDSADDLAKQTKIEYGAVRDGSTMTFFKKSKISTYEKMWAFMSSRQQTALVKNSDEGIQ 741
S.scrofa             PIDSADDLAKQTKIEYGAVRDGSTMTFFKKSKISTYEKMWAFMSSRQQTALVKNSDEGIQ 726
O.aries              PIDSADDLAKQTKIEYGAVRDGSTMTFFKKSKISTYEKMWAFMSSRQQTALVKNSDEGIH 726
G.gallus             PIDSADDLAKQTKIEYGAVRDGSTMTFFKKSKISTYEKMWAFMSSRQQTALVKNNDEGIQ 715
D.rerio              PIDSADDLAKQTKIEYGAVRDGSTMTFFKKSKISTYEKMWAFMSSRKNTALVKNNREGIQ 766
                     **********************************************:::***:*. ***:

R.norvegicus         RVLTTDYALLMESTSIEYVTQRNCNLTQIGGLIDSKGYGVGTPIGSPYRDKITIAILQLQ 801
M.musculus           RVLTTDYALLMESTSIEYVTQRNCNLTQIGGLIDSKGYGVGTPIGSPYRDKITIAILQLQ 786
M.mulatta            RVLTTDYALLMESTSIEYVTQRNCNLTQIGGLIDSKGYGVGTPIGSPYRDKITIAILQLQ 801
P.troglodytes        RVLTTDYALLMESTSIEYVTQRNCNLTQIGGLIDSKGYGVGTPIGSPYRDKITIAILQLQ 801
H.sapiens            RVLTTDYALLMESTSIEYVTQRNCNLTQIGGLIDSKGYGVGTPIGSPYRDKITIAILQLQ 801
F.catus              RVLTTDYALLMESTSIEYVTQRNCNLTQIGGLIDSKGYGVGTPIGSPYRDKITIAILQLQ 786
C.lupus              RVLTTDYALLMESTSIEYVTQRNCNLTQIGGLIDSKGYGVGTPIGSPYRDKITIAILQLQ 801
S.scrofa             RVLTTDYALLMESTSIEYVTQRNCNLTQIGGLIDSKGYGVGTPIGSPYRDKITIAILQLQ 786
O.aries              RVLTTDYALLMESTSIEYVTQRNCNLTQIGGLIDSKGYGVGTPIGSPYRDKITIAILQLQ 786
G.gallus             RVLTTDYALLMESTSIEYVTQRNCNLTQIGGLIDSKGYGVGTPIGSPYRDKITIAILQLQ 775
D.rerio              RVLTTDYALLMESTSIEYISQRNCNLTQIGGLIDSKGYGVGTPIGSPYRDKVTIAILQLQ 826
                     ******************::*******************************:********

R.norvegicus         EEGKLHMMKEKWWRGNGCPE 821
M.musculus           EEGKLHMMKEKWWRGNGCPE 806
M.mulatta            EEGKLHMMKEKWWRGNGCPE 821
P.troglodytes        EEGKLHMMKEKWWRGNGCPE 821
H.sapiens            EEGKLHMMKEKWWRGNGCPE 821
F.catus              EEGKLHMMKEKWWRGNGCPE 806
C.lupus              EEGKLHMMKEKWWRGNGCPE 821
S.scrofa             EEGKLHMMKEKWWRGNGCPE 806
O.aries              EEGKLHMMKEKWWRGNGCPE 806
G.gallus             EEGKLHMMKEKWWRGNGCPE 795
D.rerio              EEGKLHMMKEKWWRGNGCPE 846
                     ********************
```

Sequence alignment using ClustalW2 of the GluK1 ligand binding domain segments from several species frequently used as animal models for retina[7]. Segment 1 comprises amioacids 446 to 559, and segment 2 aminoacids 682 to 821 of the *R. norvegicus* gene, access number NP_001104587.1. The alignment shows that GluK1 ligand binding domain amino acid sequence is highly conserved across species, including lysines were the TCP conjugates. Lysines and arginines are highlighted in red.

NMR spectra

Supplementary Figure 24. ^{1}H **NMR (500 MHz,** CD_3OD**) and** ^{13}C **NMR (125 MHz,** CD_3OD**) spectra of 21**

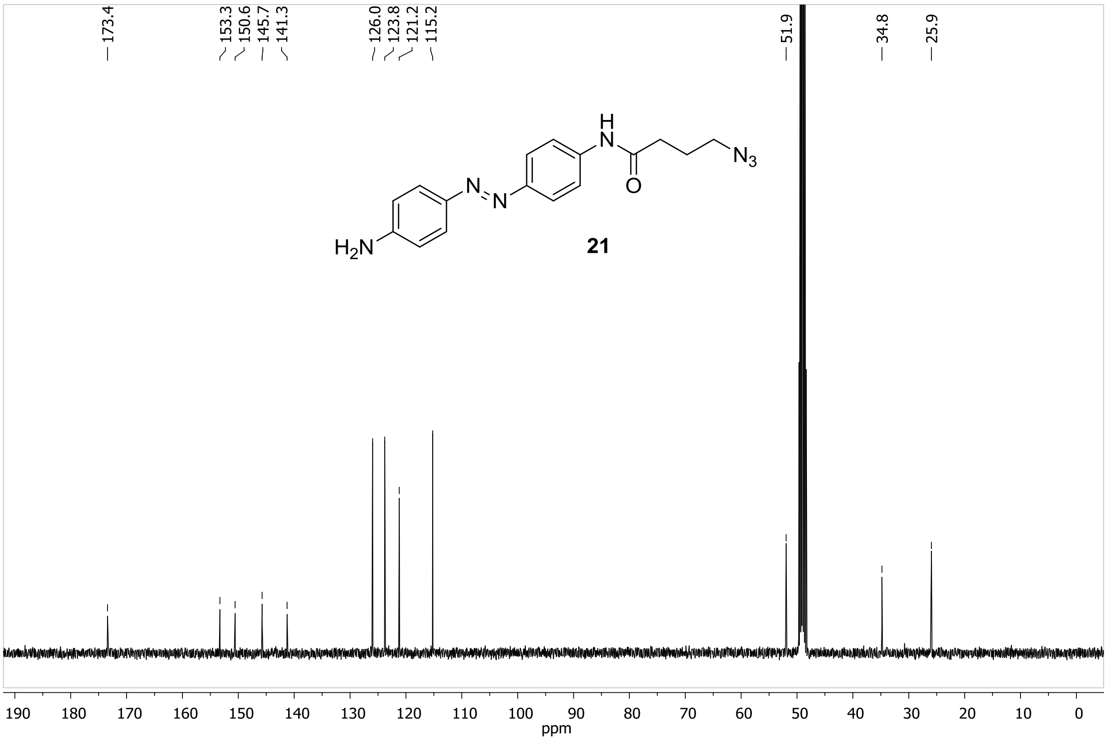

Supplementary Figure 25. **^{1}H NMR (400 MHz, $CDCl_3$) and ^{13}C NMR (100 MHz, $CDCl_3$) spectra of 26**

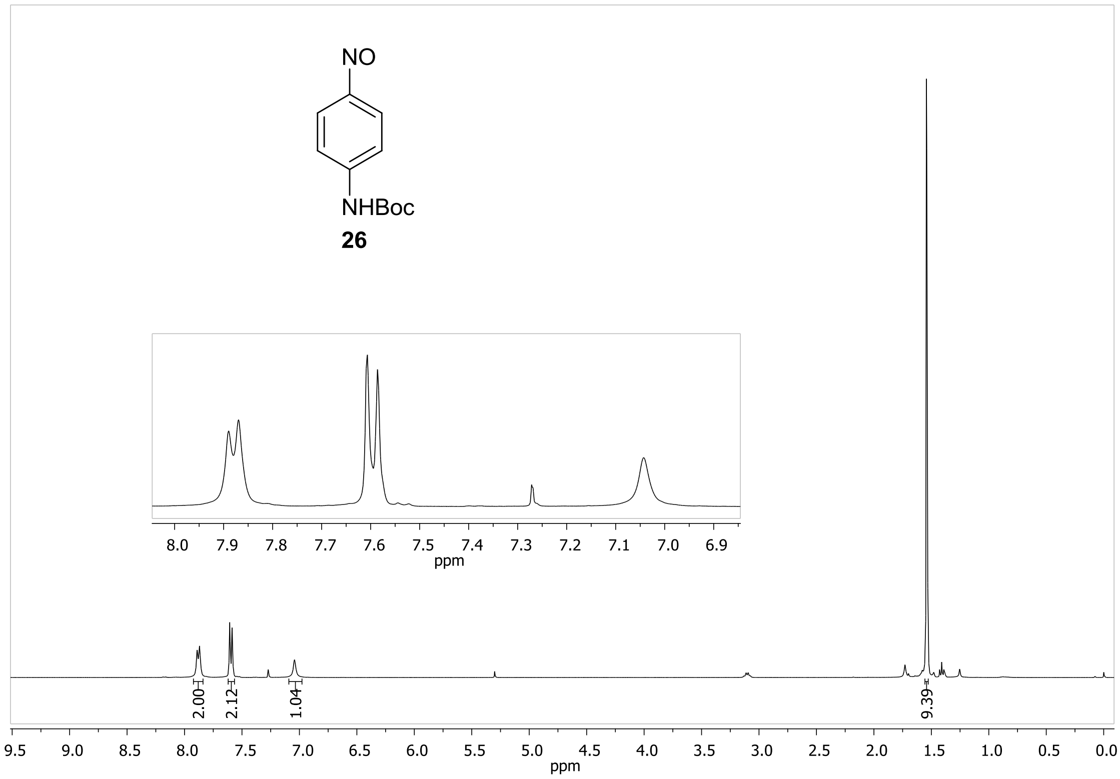

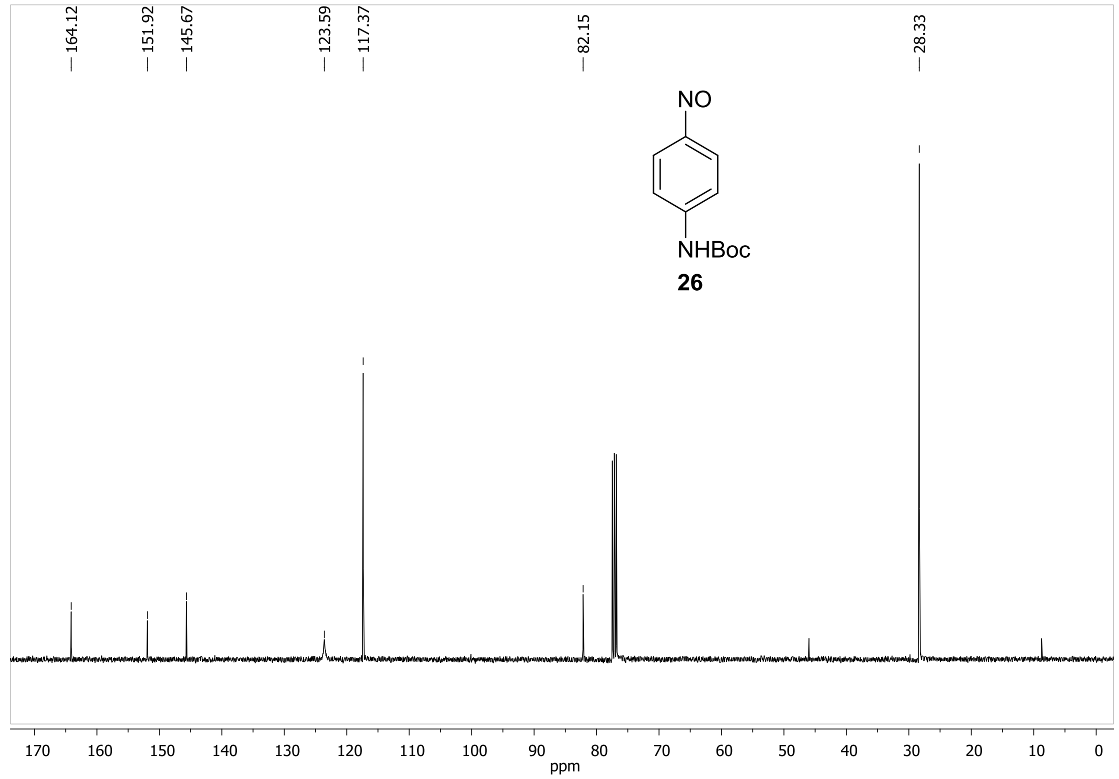

Supplementary Figure 26. ^{1}H NMR (400 MHz, $CDCl_3$) and ^{13}C NMR (100 MHz, $CDCl_3$) spectra of 27

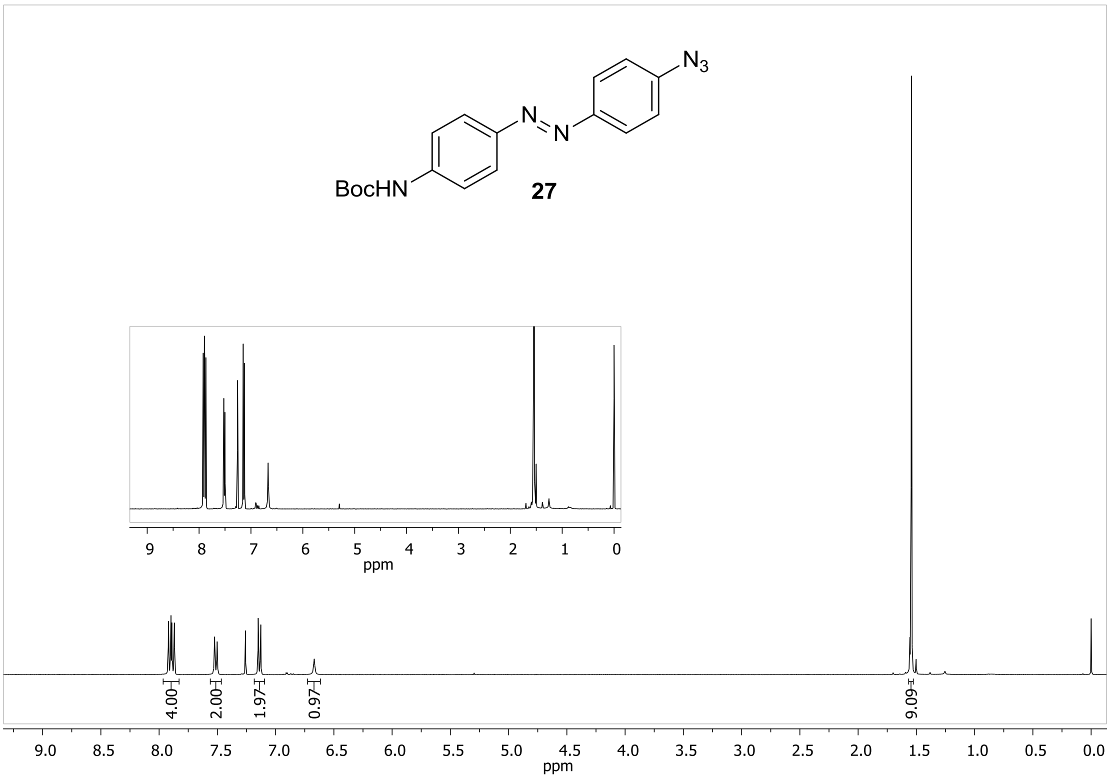

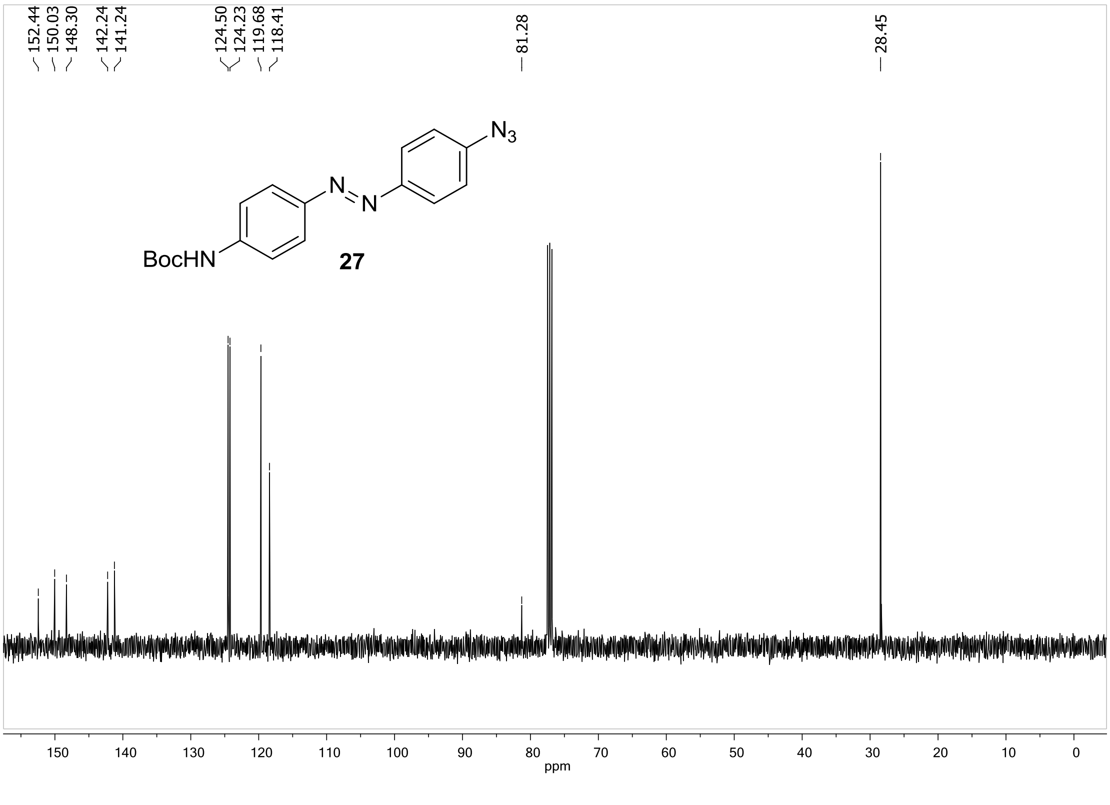

Supplementary Figure 27. ^1H **NMR (400 MHz, DMSO-d_6) and** ^{13}C **NMR (100 MHz, DMSO-d_6) spectra of 22**

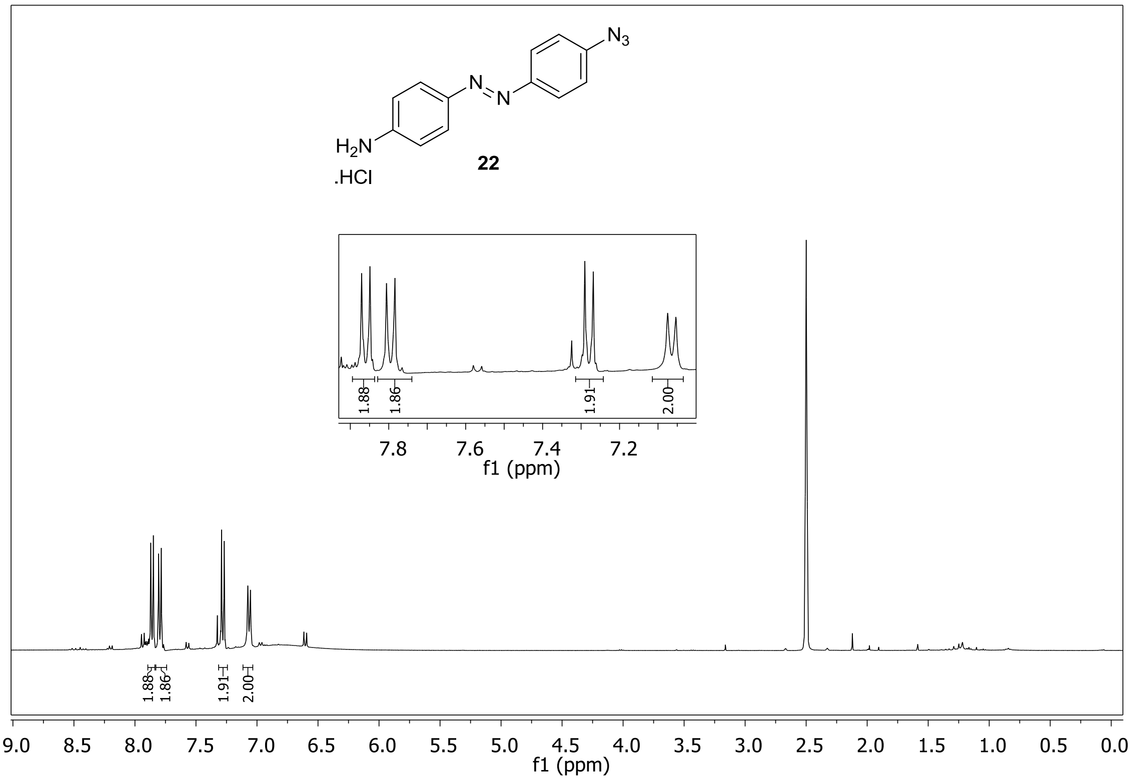

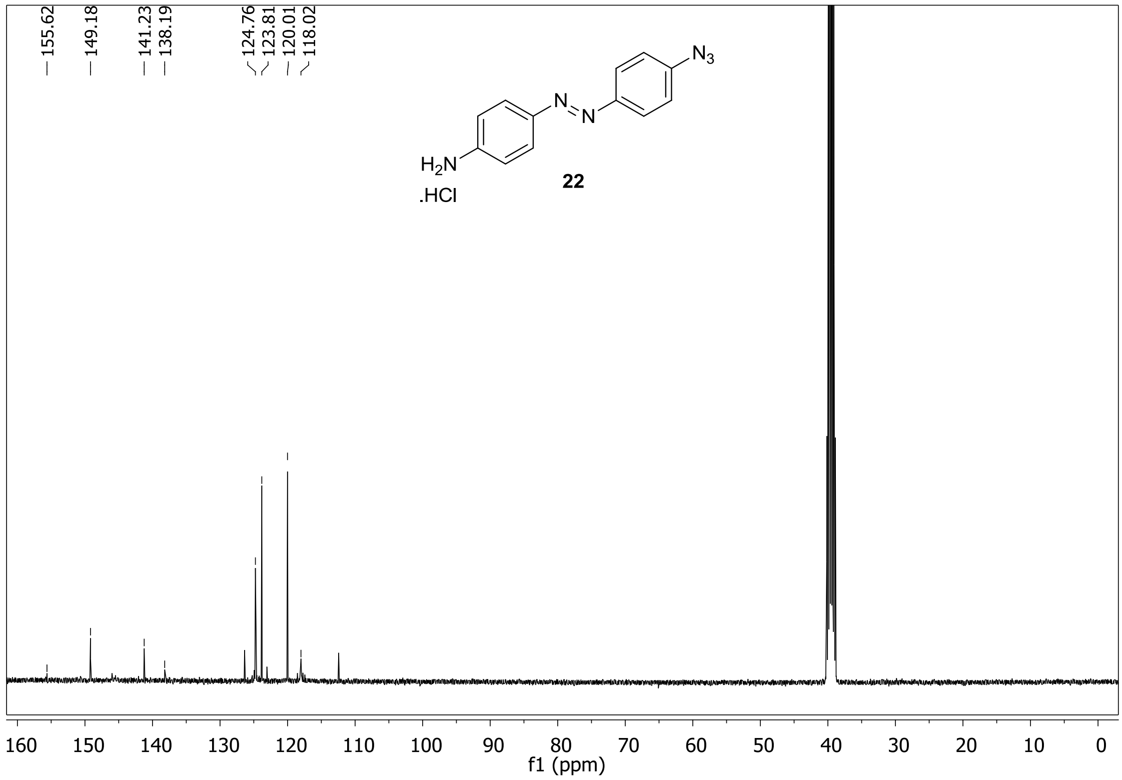

Supplementary Figure 28. ^{1}H NMR (400 MHz, $CDCl_3$) and ^{13}C NMR (100 MHz, $CDCl_3$) spectra of 23

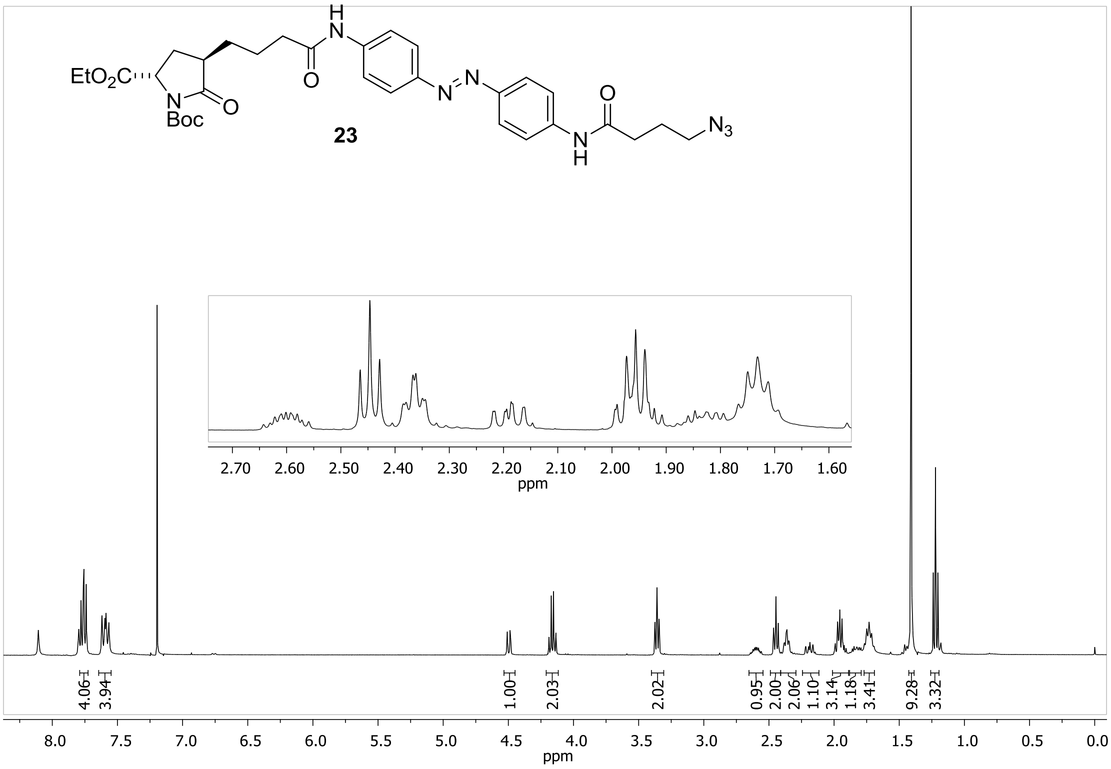

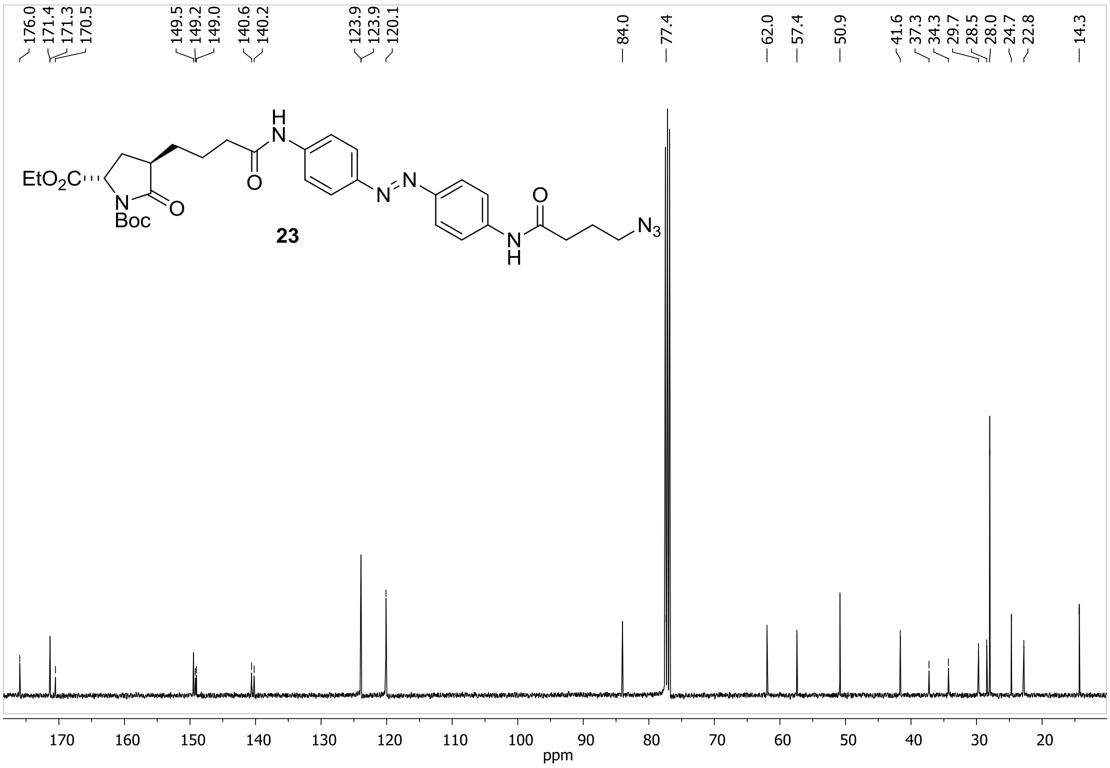

Supplementary Figure 29. ^{1}H NMR (400 MHz, $CDCl_3$) and ^{13}C NMR (100 MHz, $CDCl_3$) spectra of **24**

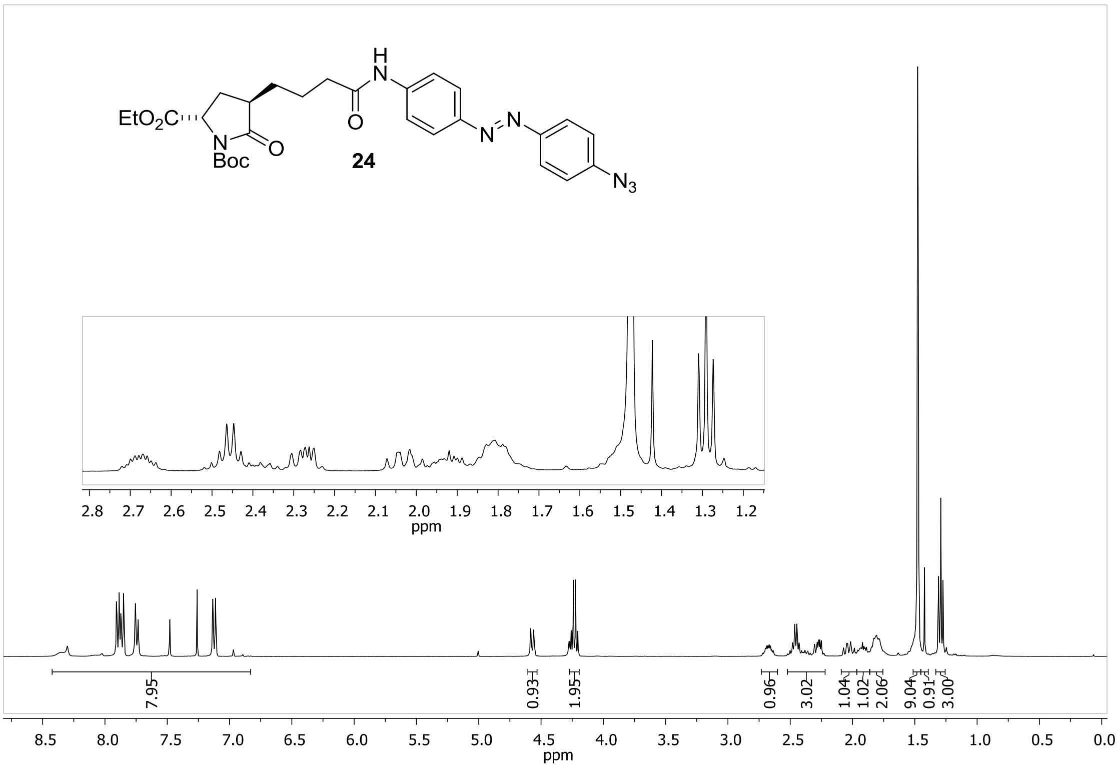

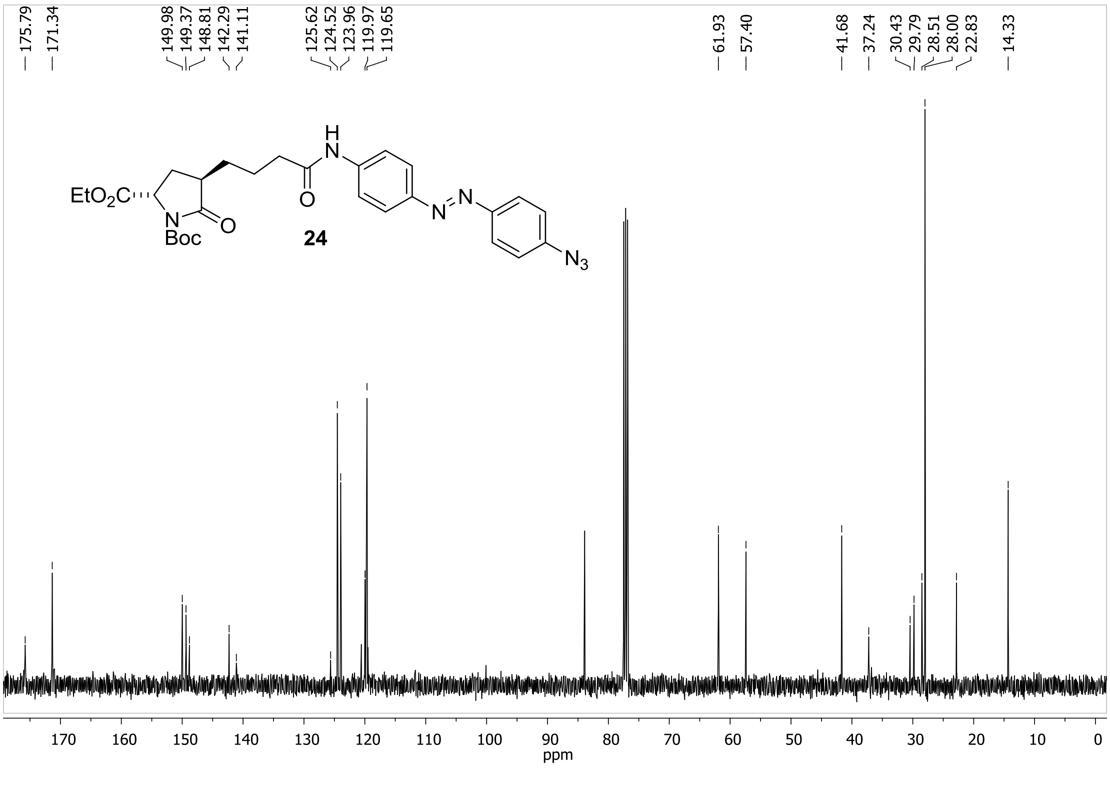

Supplementary Figure 30. ^{1}H NMR (400 MHz, CD_3OD) and ^{13}C NMR (100 MHz, CD_3OD) spectra of 28

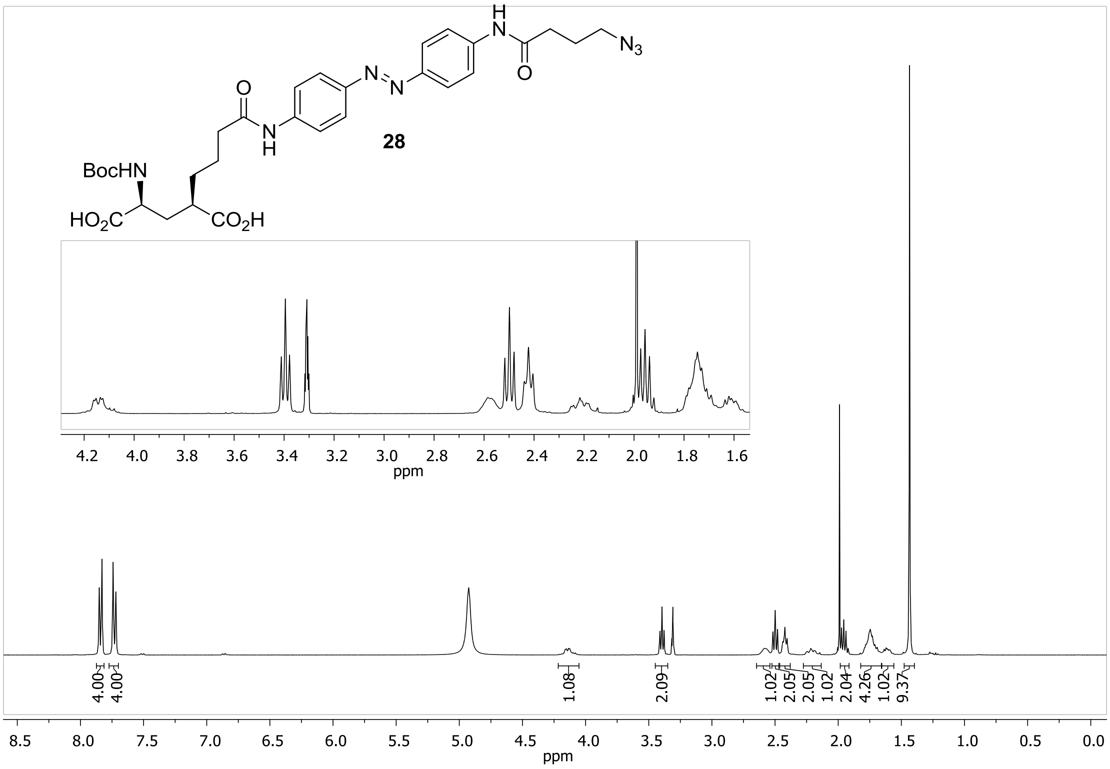

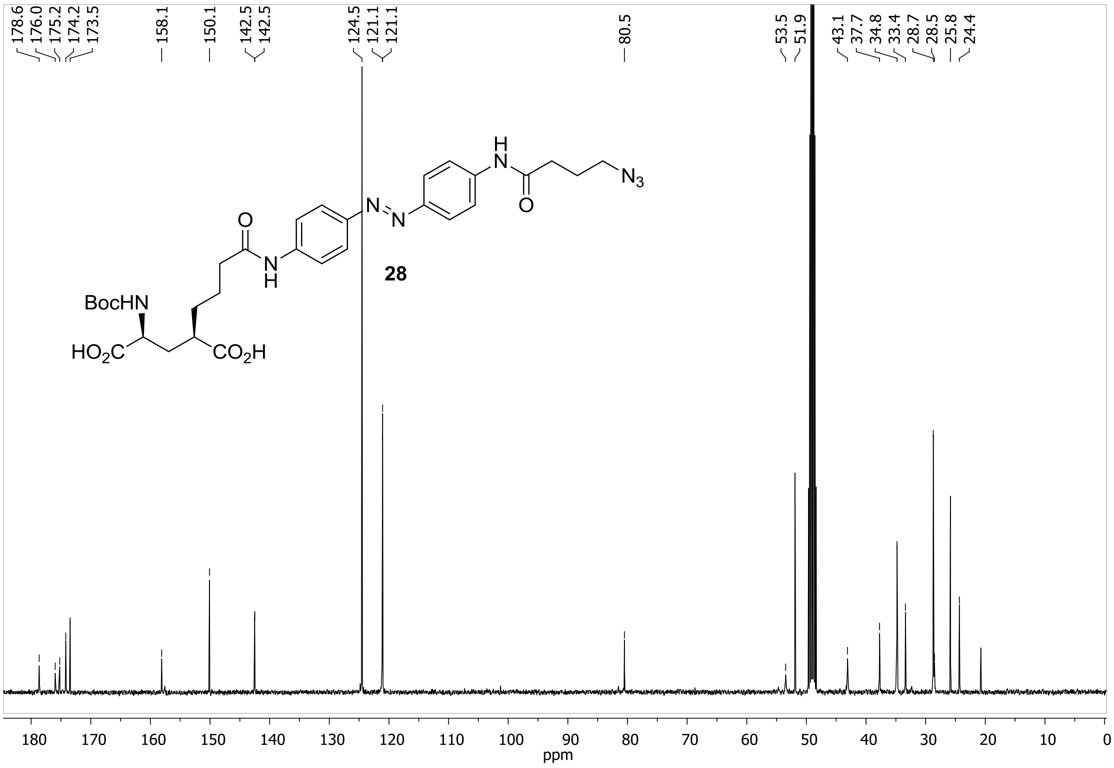

Supplementary Figure 31. ^{1}**H NMR (400 MHz,** CD_3OD**) and** ^{13}**C NMR (100 MHz,** CD_3OD**) spectra of 29**

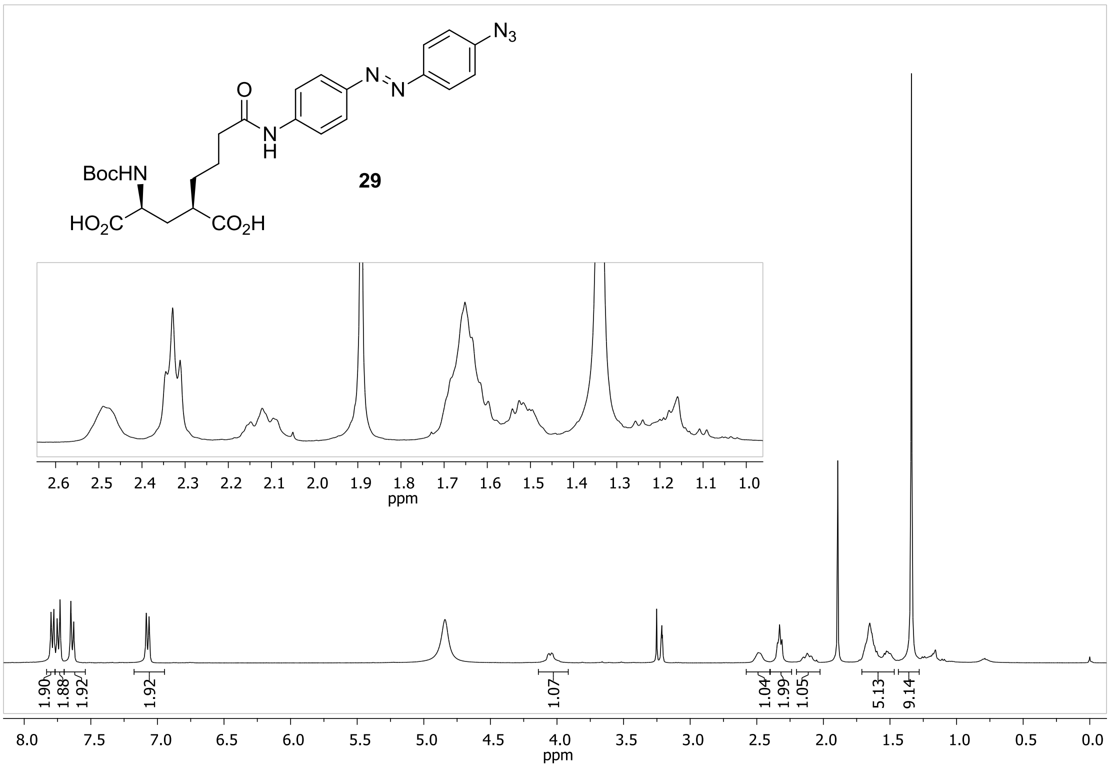

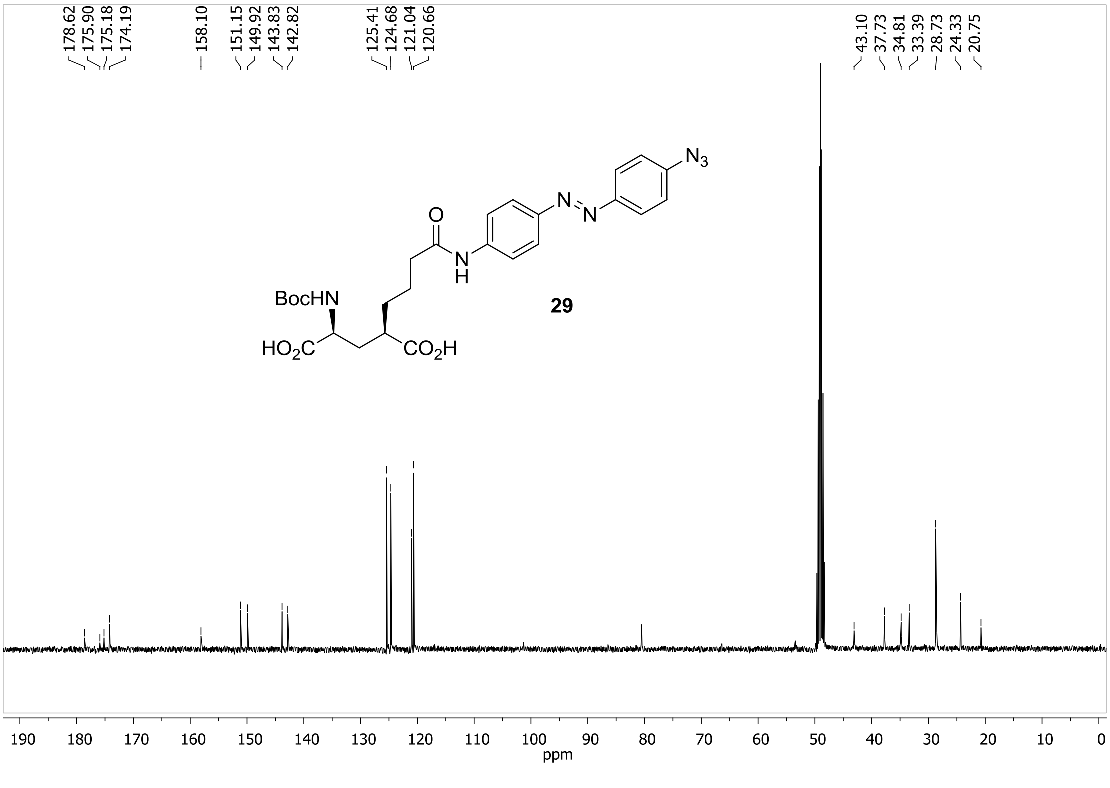

Supplementary Figure 32. ^{1}H NMR (400 MHz, CD_3OD) and ^{13}C NMR (100 MHz, CD_3OD) spectra of 1

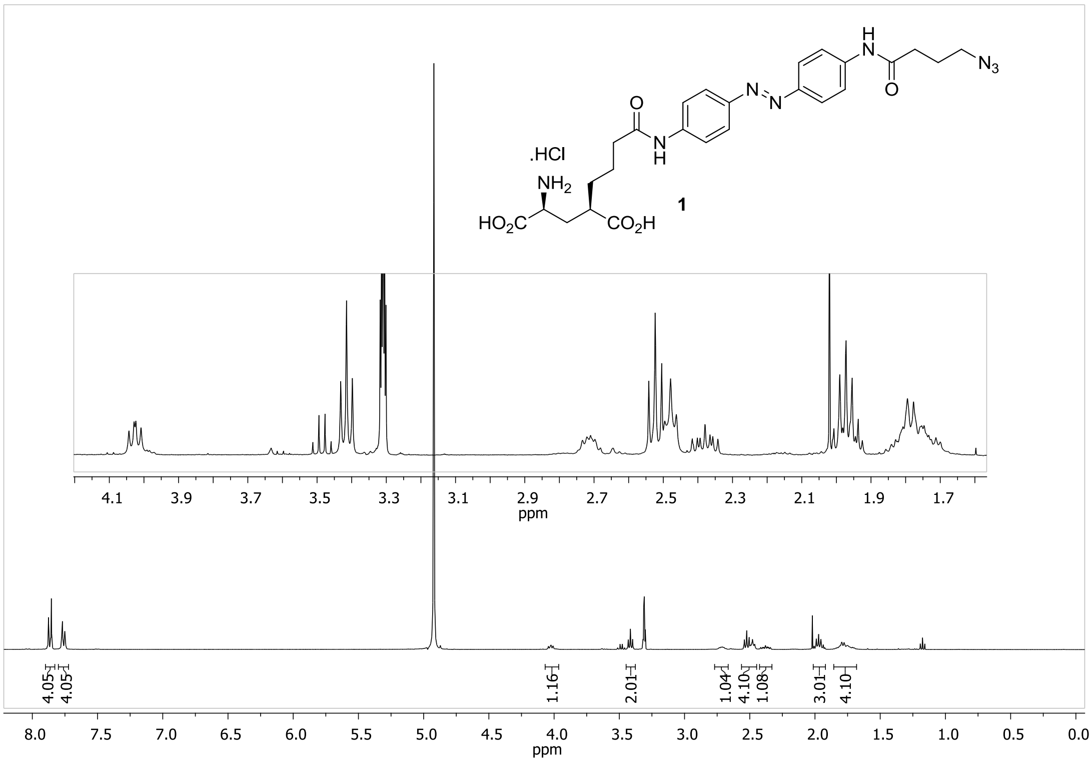

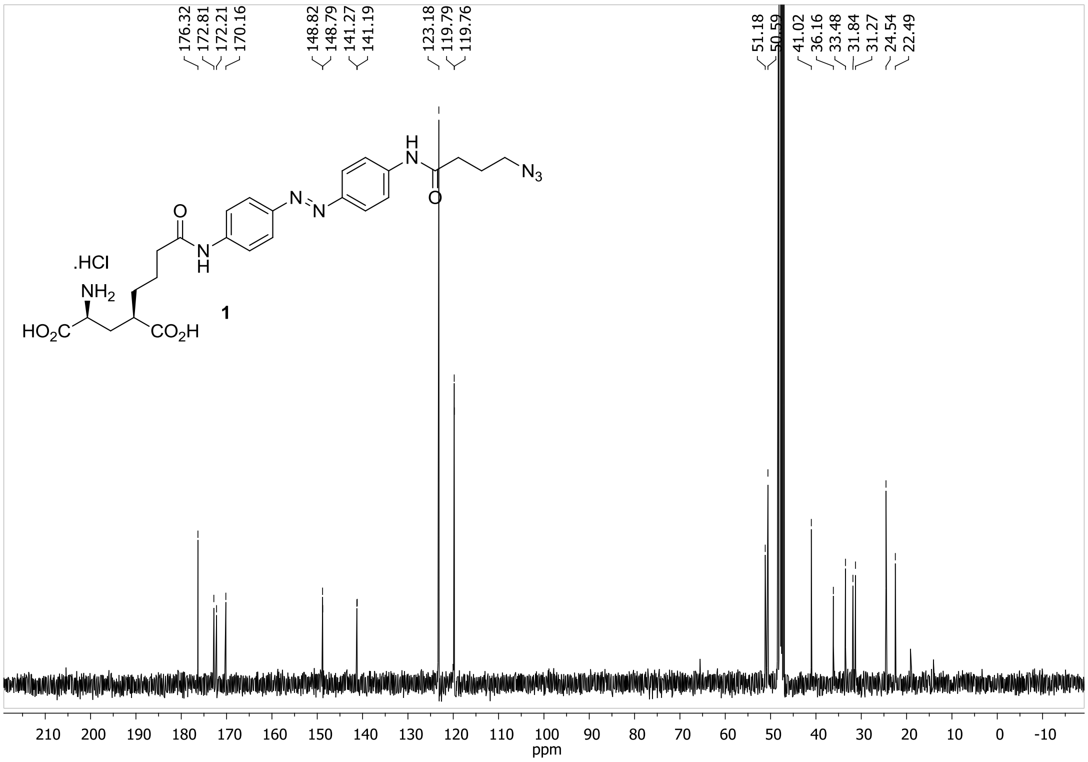

Supplementary Figure 33. ^{1}H NMR (400 MHz, CD_3OD) and ^{13}C NMR (100 MHz, CD_3OD) spectra of 2

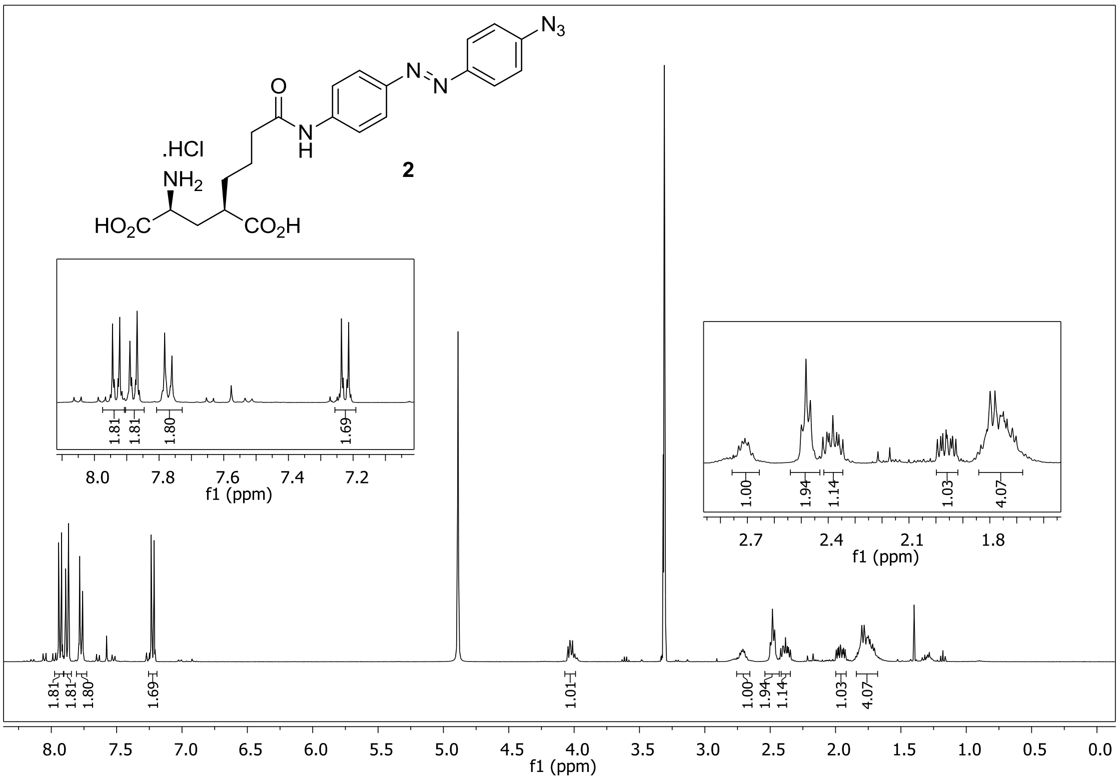

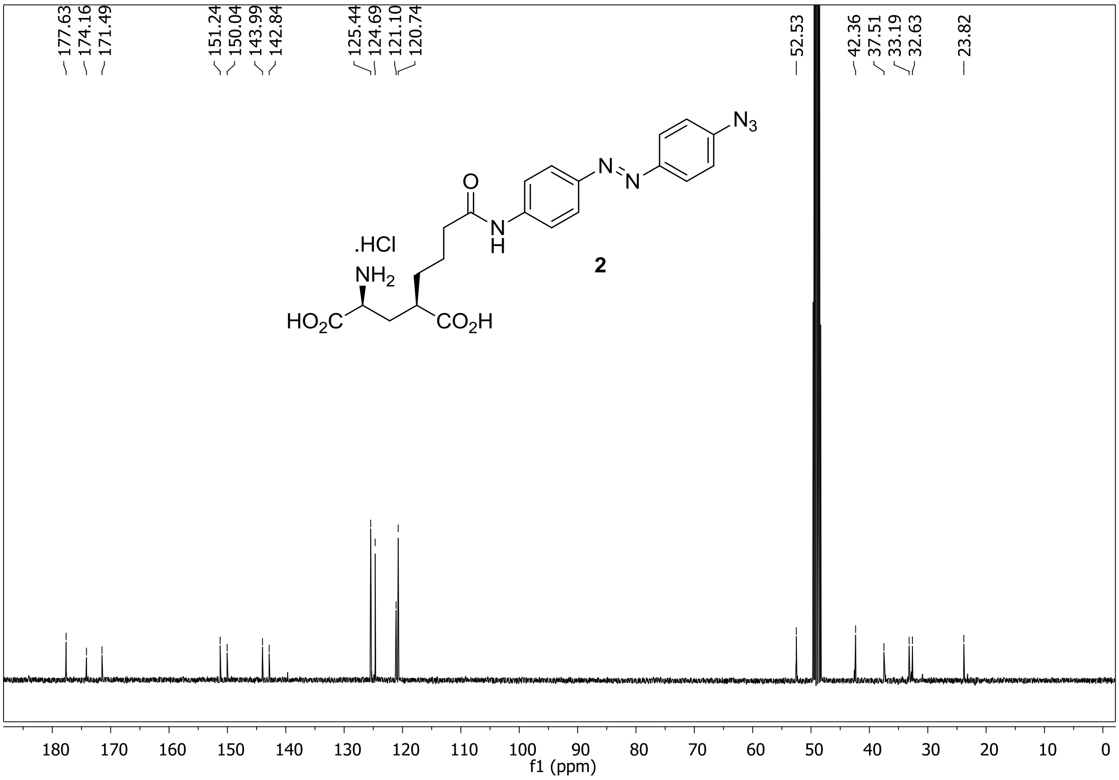

Supplementary Figure 34. ^{1}H NMR (400 MHz, $CDCl_3$) and ^{13}C NMR (100 MHz, $CDCl_3$) spectra of 4

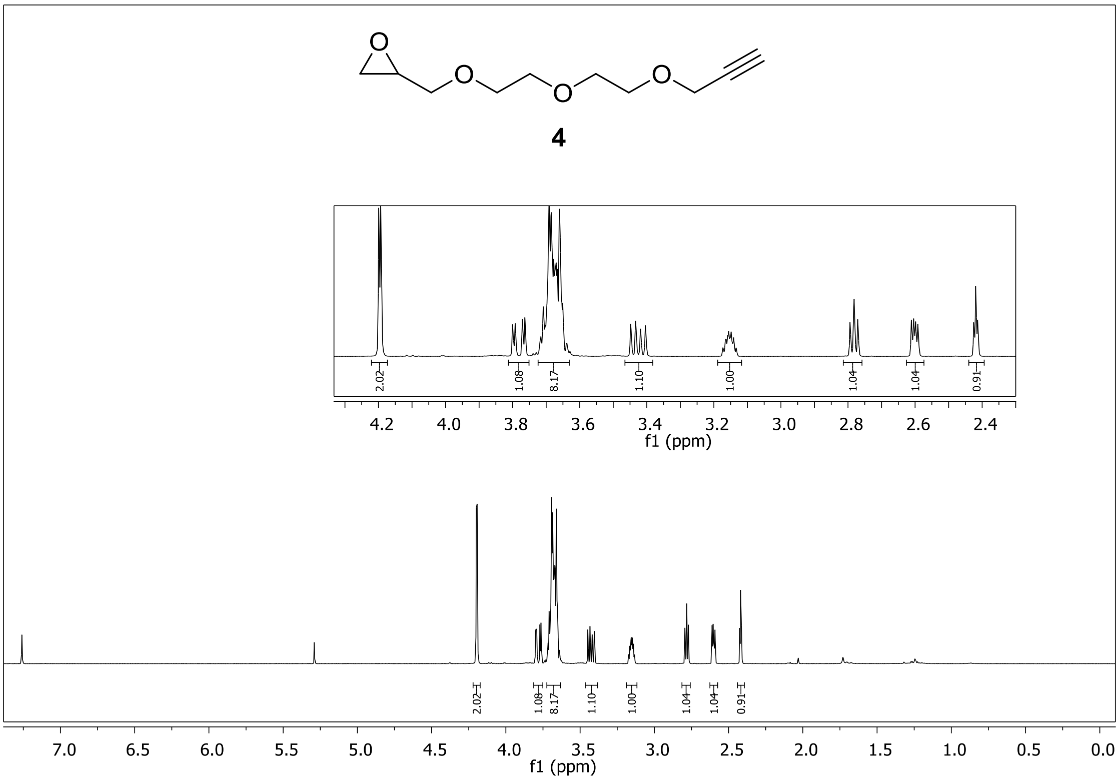

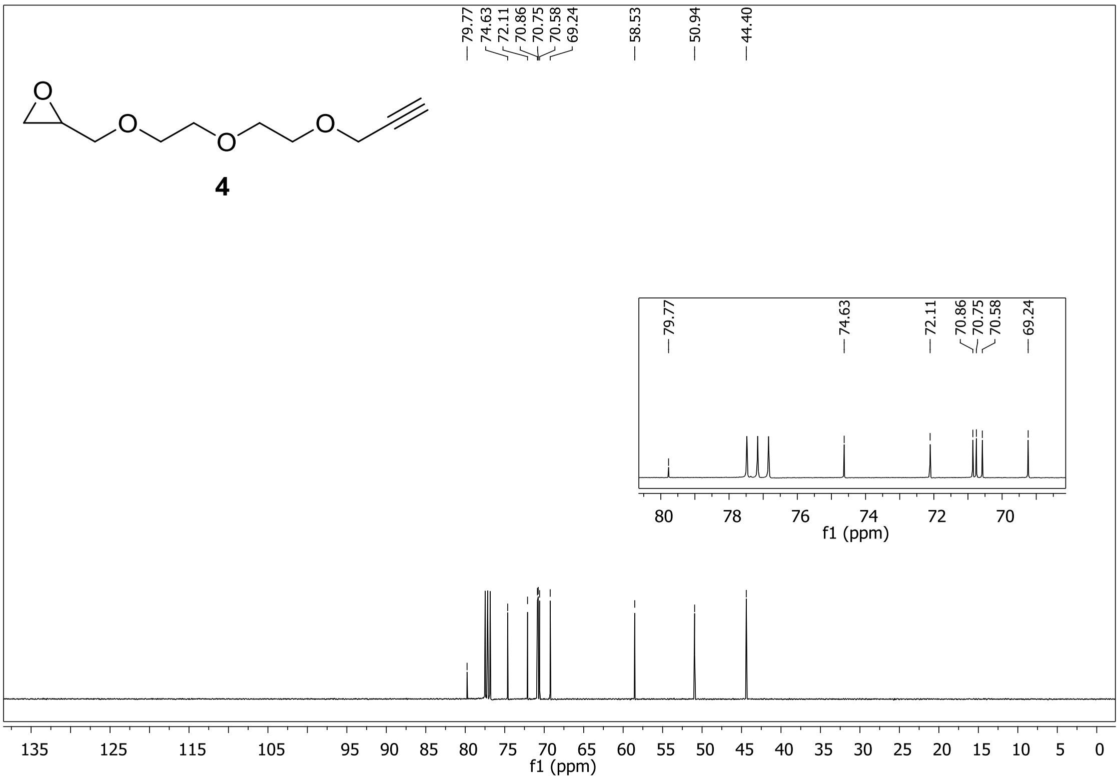

Supplementary Figure 35. ^{1}H NMR (400 MHz, $CDCl_3$) and ^{13}C NMR (100 MHz, $CDCl_3$) spectra of 31

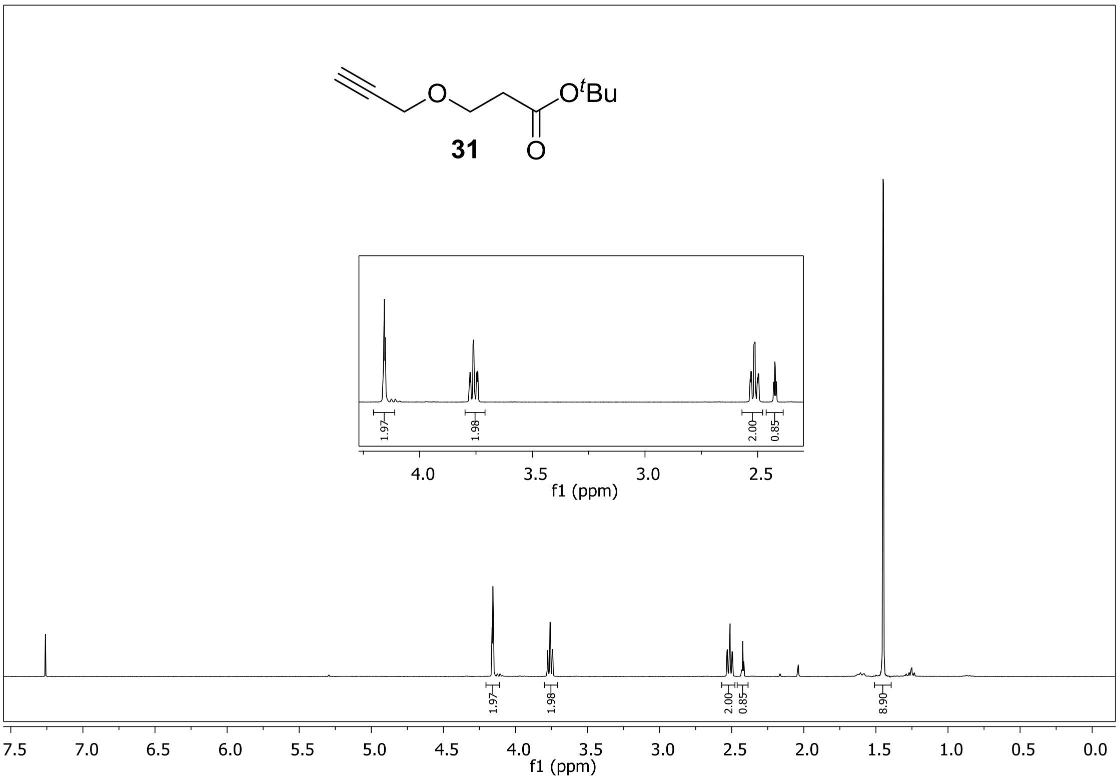

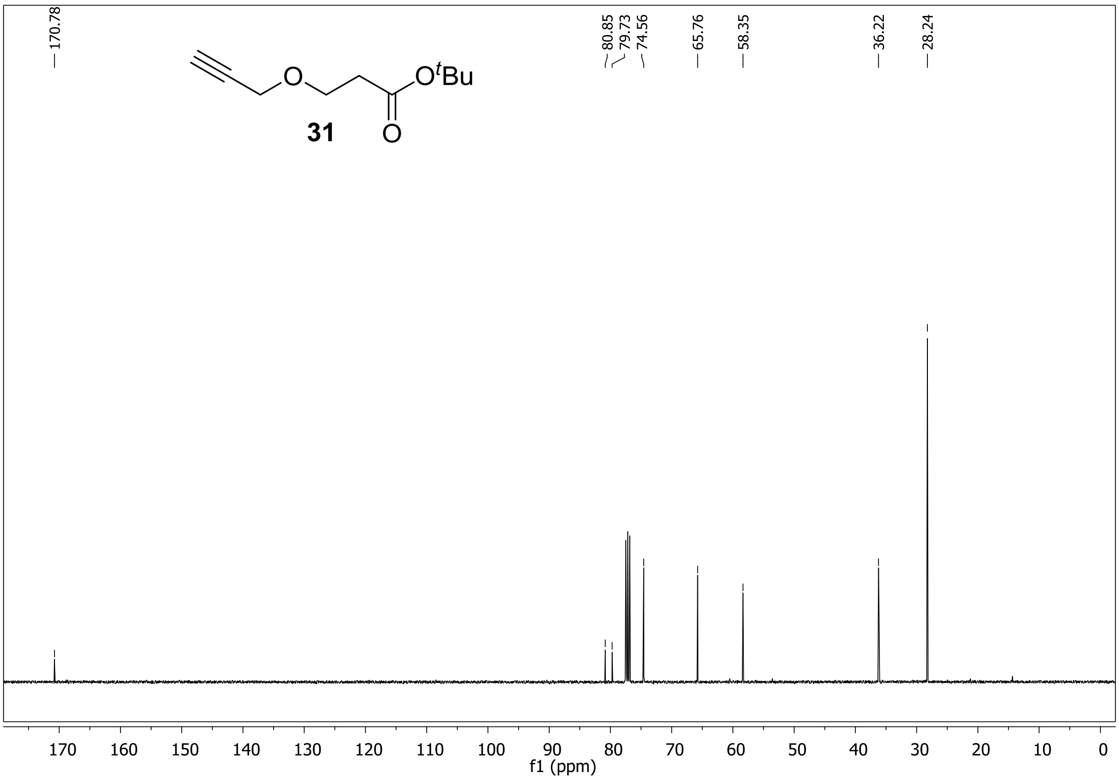

Supplementary Figure 36. ^{1}H NMR (400 MHz, $CDCl_3$) and ^{13}C NMR (100 MHz, $CDCl_3$) spectra of 32

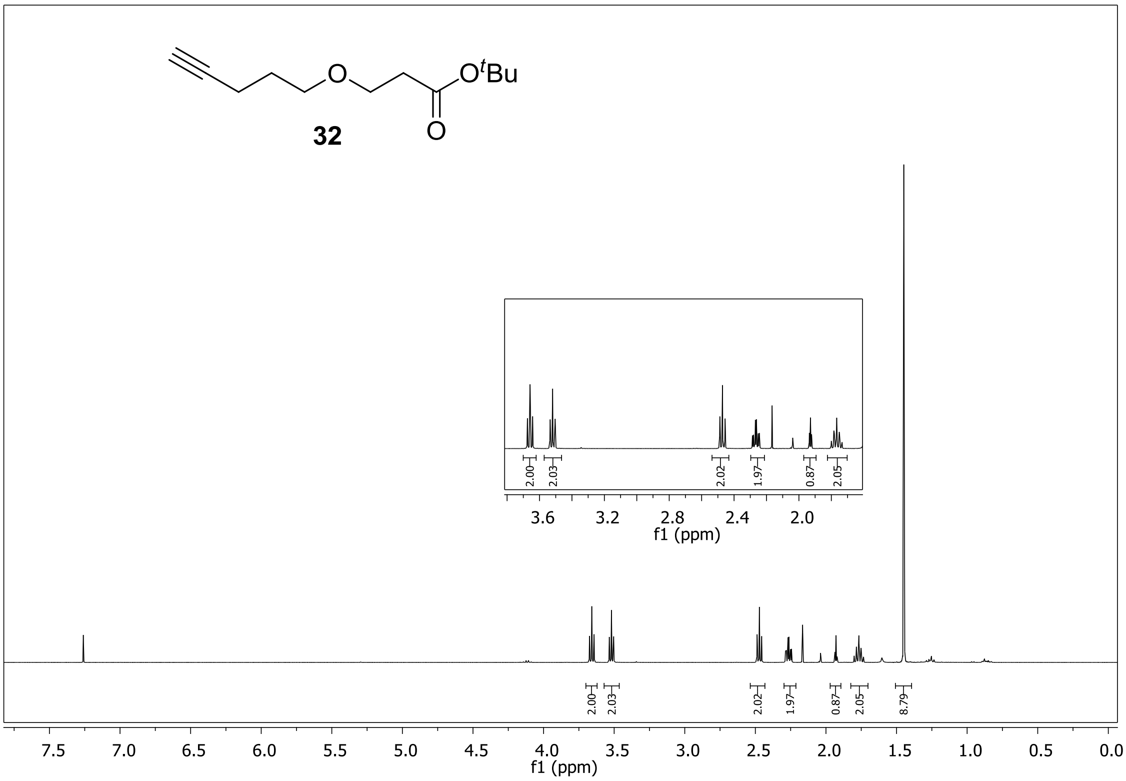

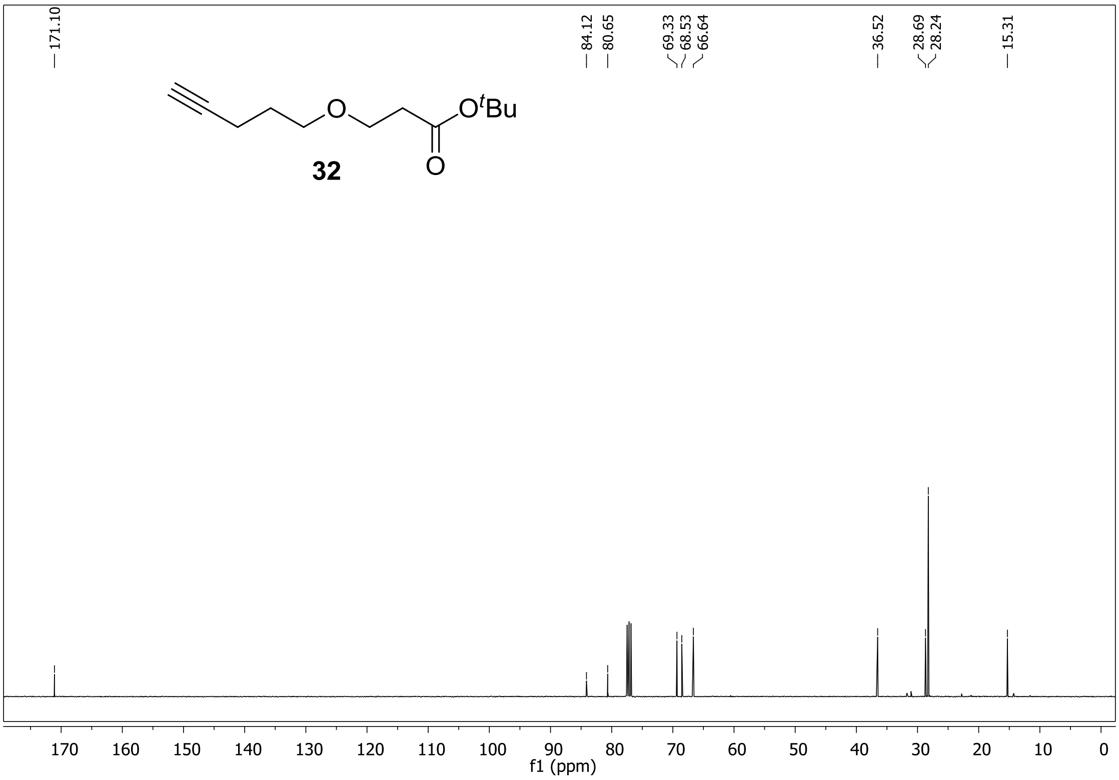

Supplementary Figure 37. **^{1}H NMR (400 MHz, $CDCl_3$) and ^{13}C NMR (100 MHz, $CDCl_3$) spectra of 33**

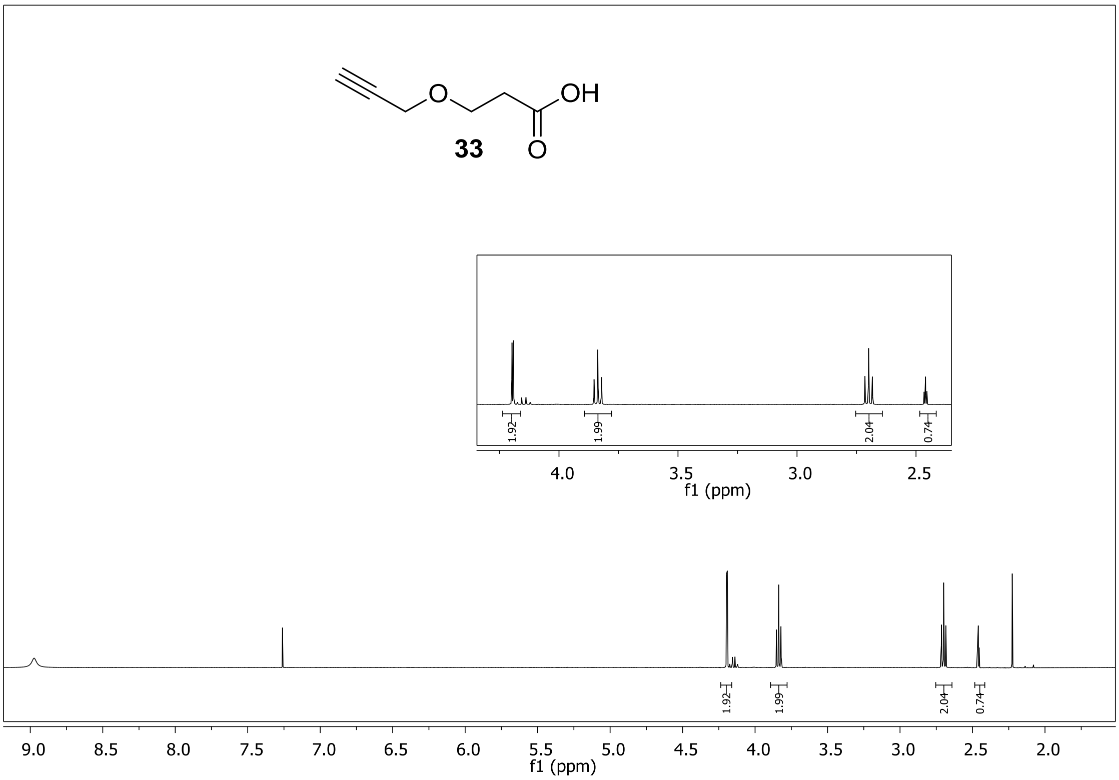

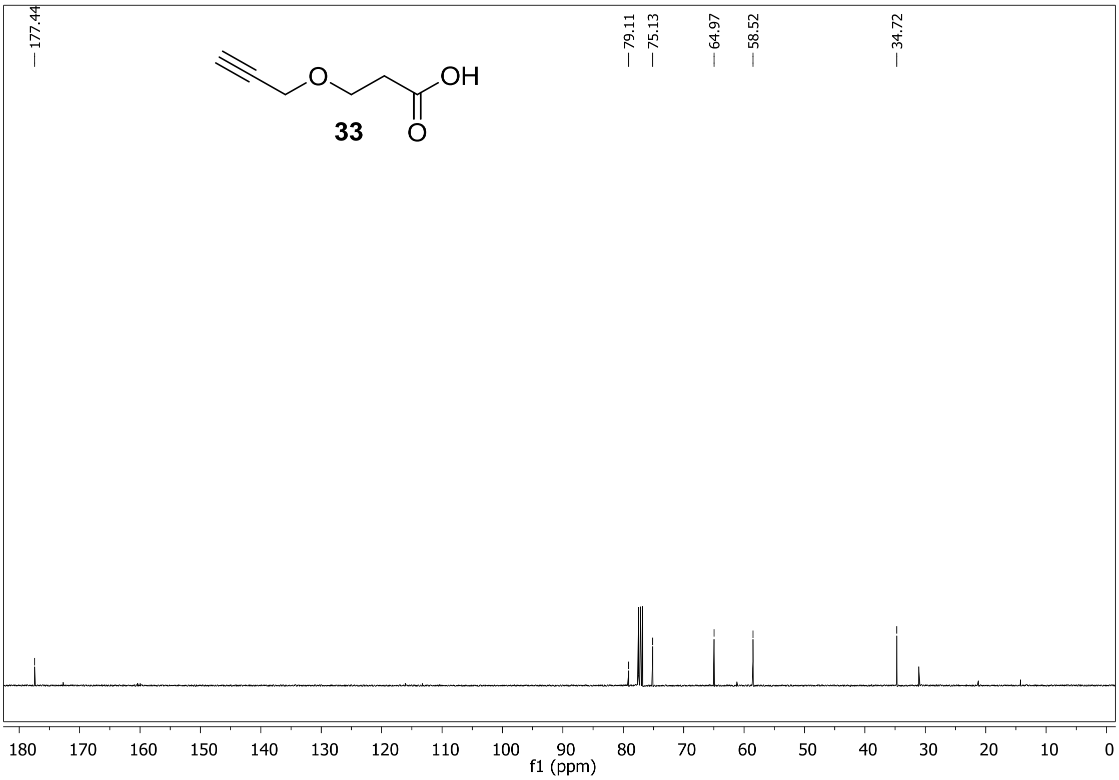

Supplementary Figure 38. ^{1}**H NMR (400 MHz,** $CDCl_3$**) and** ^{13}**C NMR (100 MHz,** $CDCl_3$**) spectra of 34**

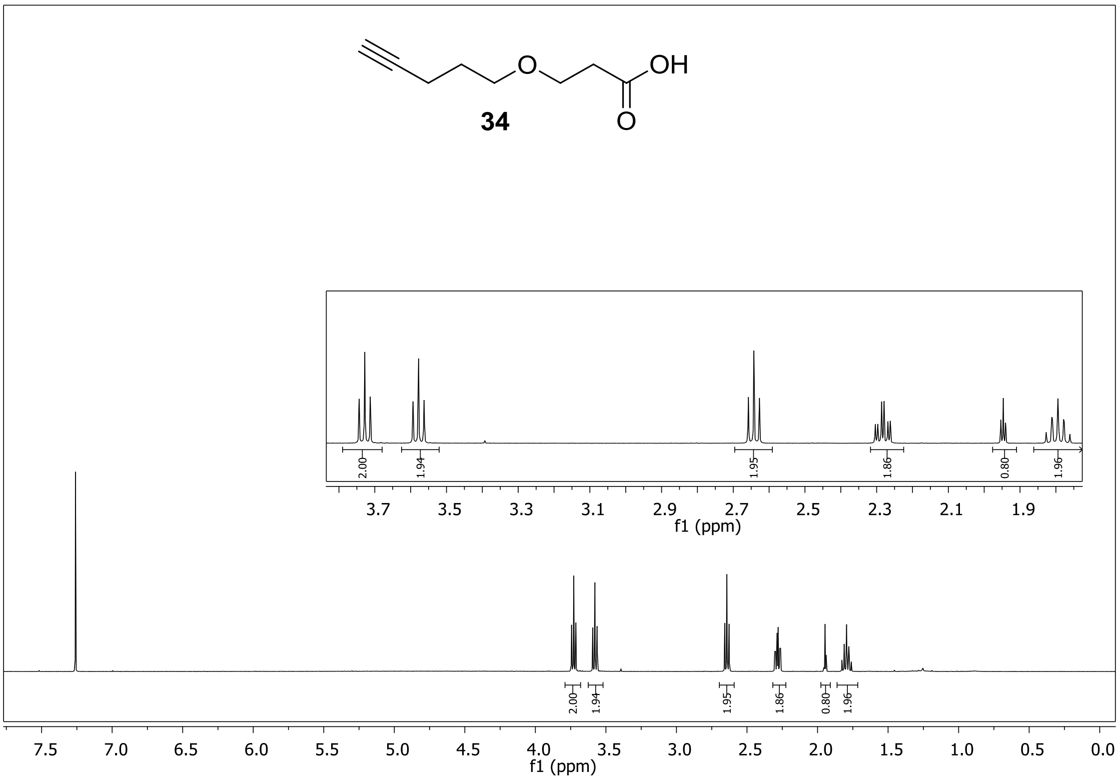

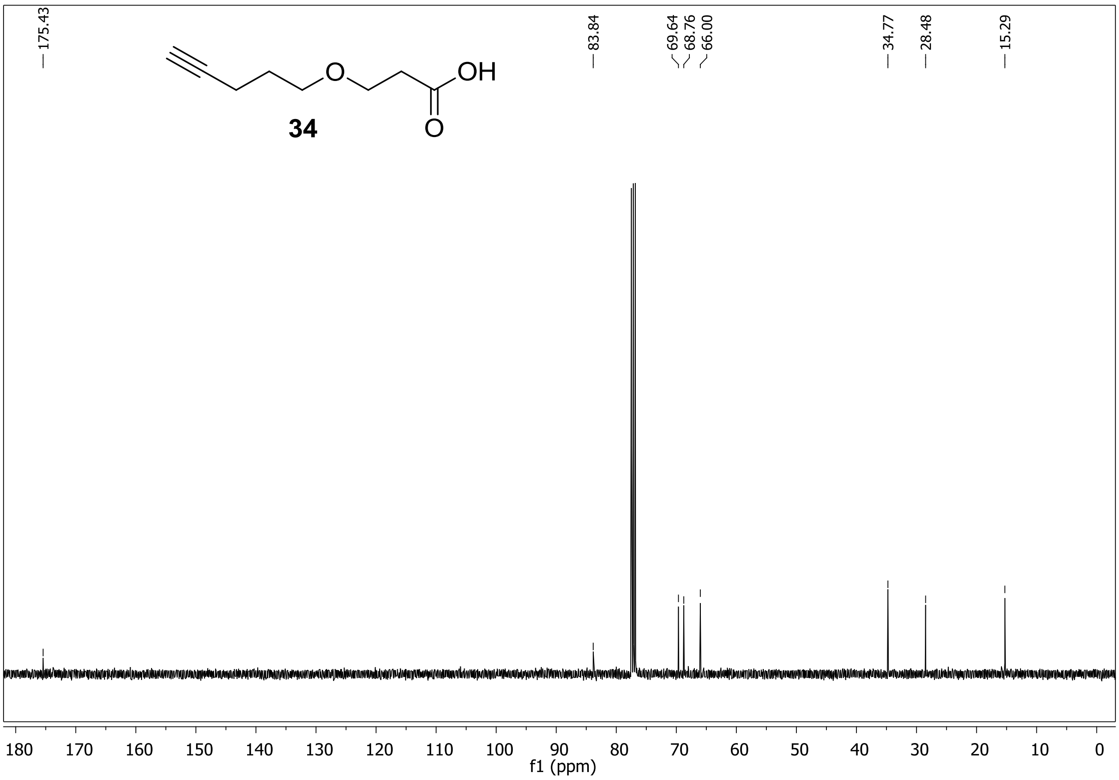

Supplementary Figure 39. ^{1}**H NMR (400 MHz, CDCl**$_3$**) and** ^{13}**C NMR (100 MHz, CDCl**$_3$**) spectra of 5**

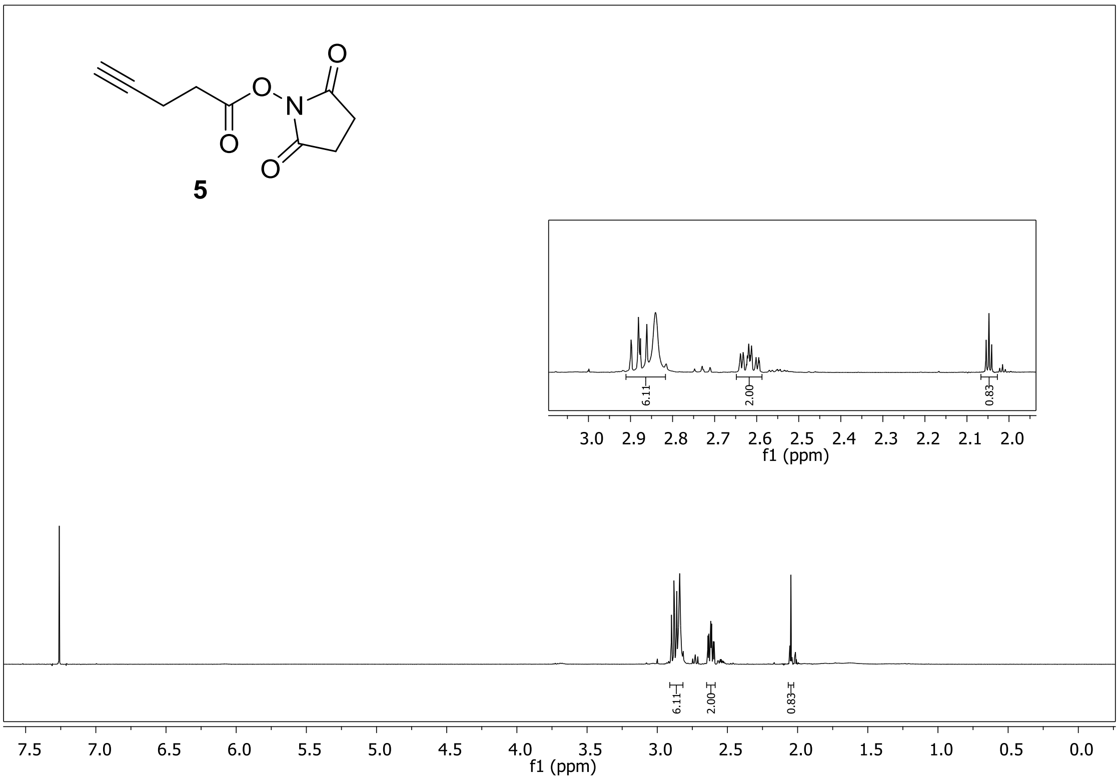

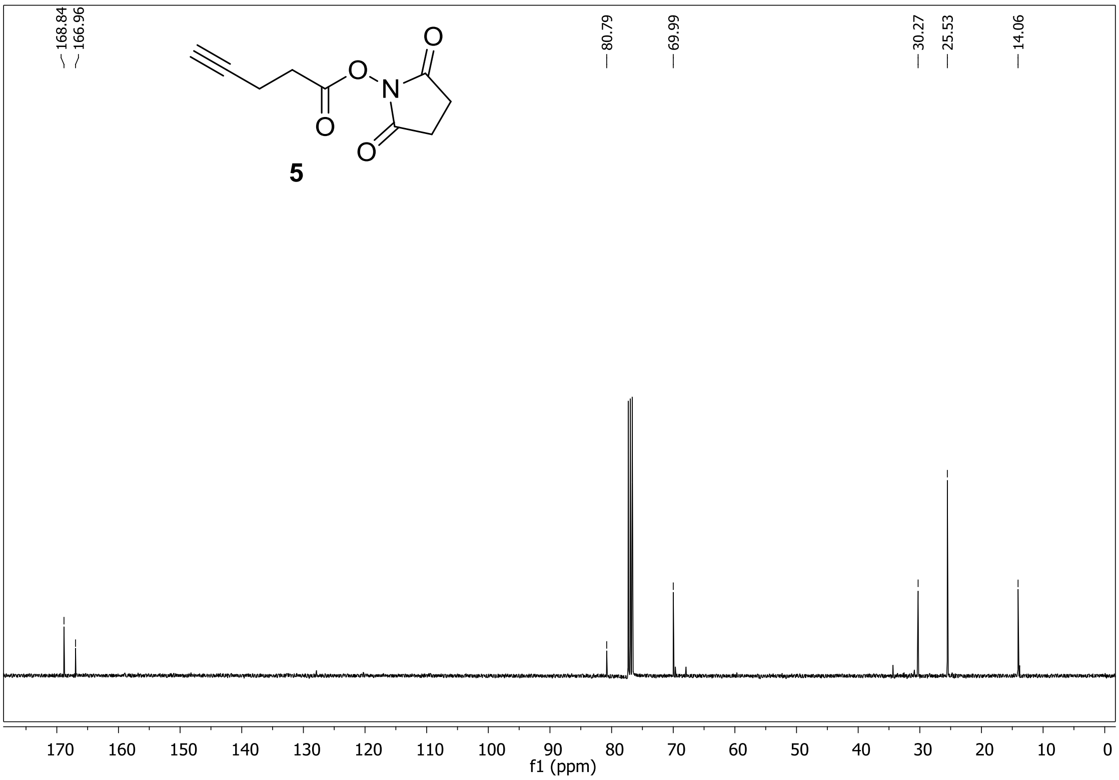

Supplementary Figure 40. **^{1}H NMR (400 MHz, $CDCl_3$) and ^{13}C NMR (100 MHz, $CDCl_3$) spectra of 6**

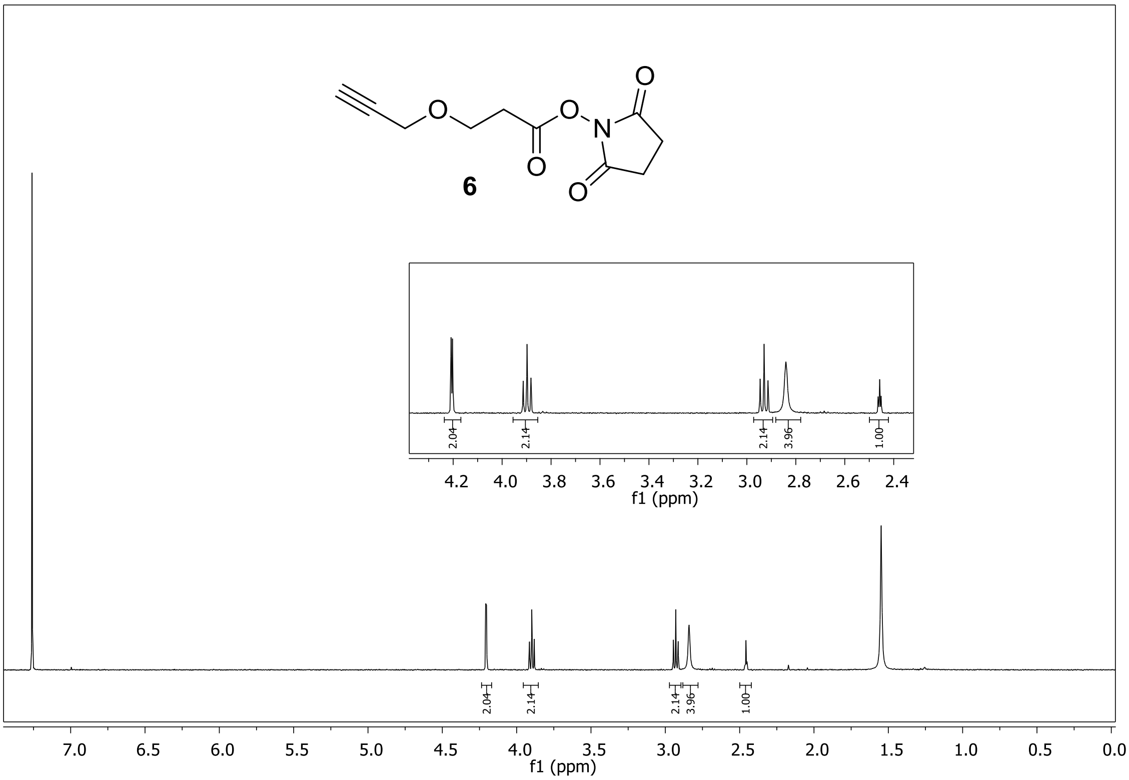

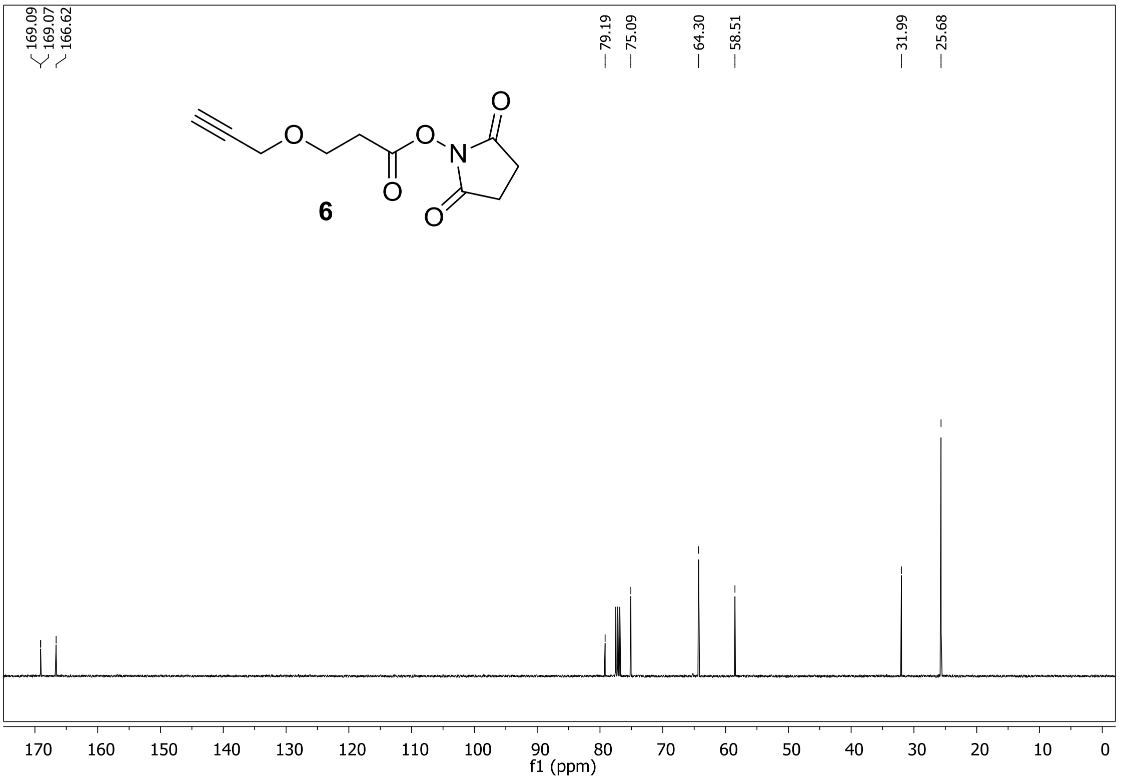

Supplementary Figure 41. ^{1}H **NMR (400 MHz,** $CDCl_3$**) and** ^{13}C **NMR (100 MHz,** $CDCl_3$**) spectra of 7**

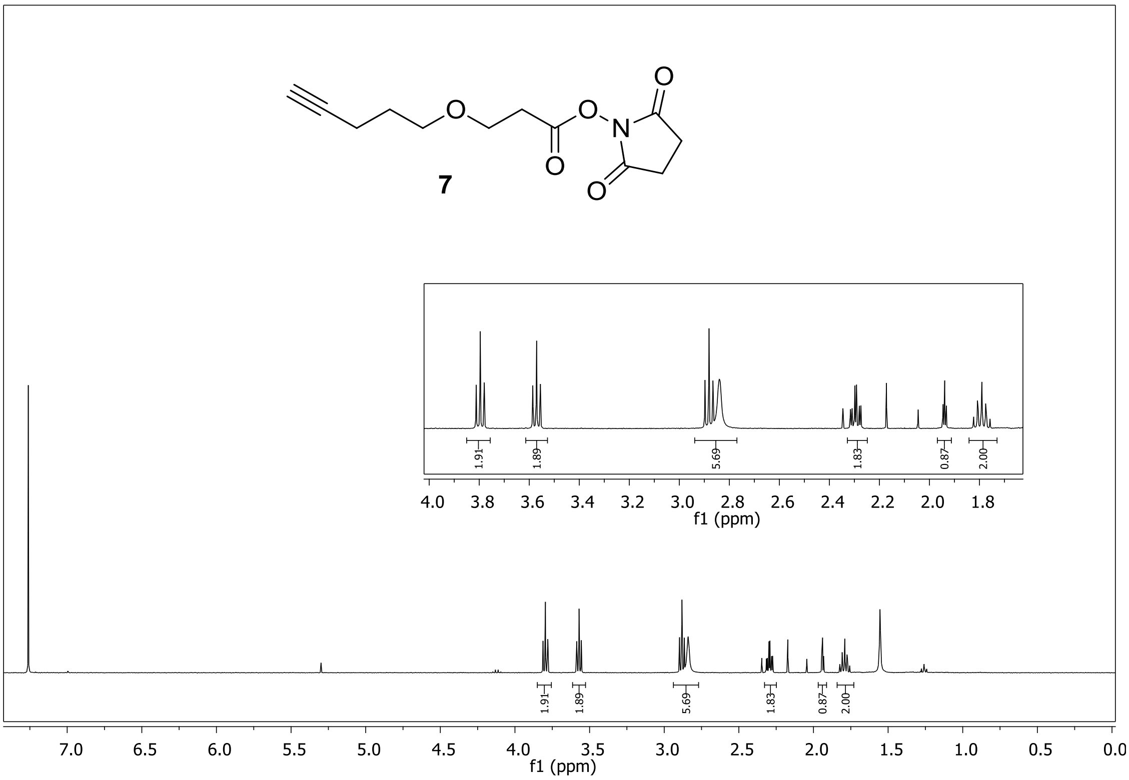

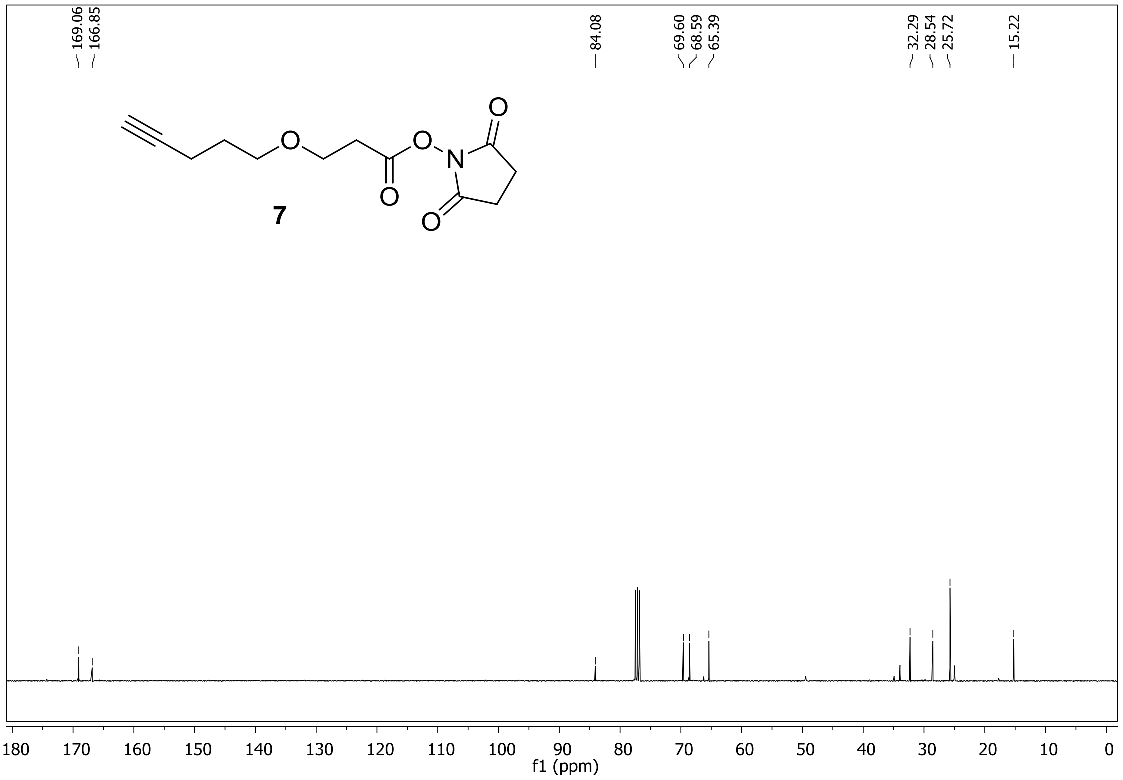

HPLC Chromatograms of compounds 1 and 2

The purity of compounds **1** and **2** was >95% as determined by HPLC on an Alliance 2695 system with UV detection at 360 nm using a ZORBAX Eclipse Plus C18 (4.6 mm × 75 mm, 3.5 μm) column under the following chromatography conditions: mobile phase A, H_2O with 0.2% HCO_2H; mobile phase B, CH_3CN with 0.2% HCO_2H; flow rate, 1.0 mL min^{-1}; injection volume, 5 μL; elution gradient, 0.0−5.0 min, 5−90% B; 5.0−7.0min, 90% B; 7.0−8.0 min, 90−100% B; 8.0−10.0 min, 100% B; 10.0−11.0 min, 100−5% B; 11.0−15.0 min, 5% B.

Supplementary Figure 42. HPLC chromatograms of compounds 1 and 2

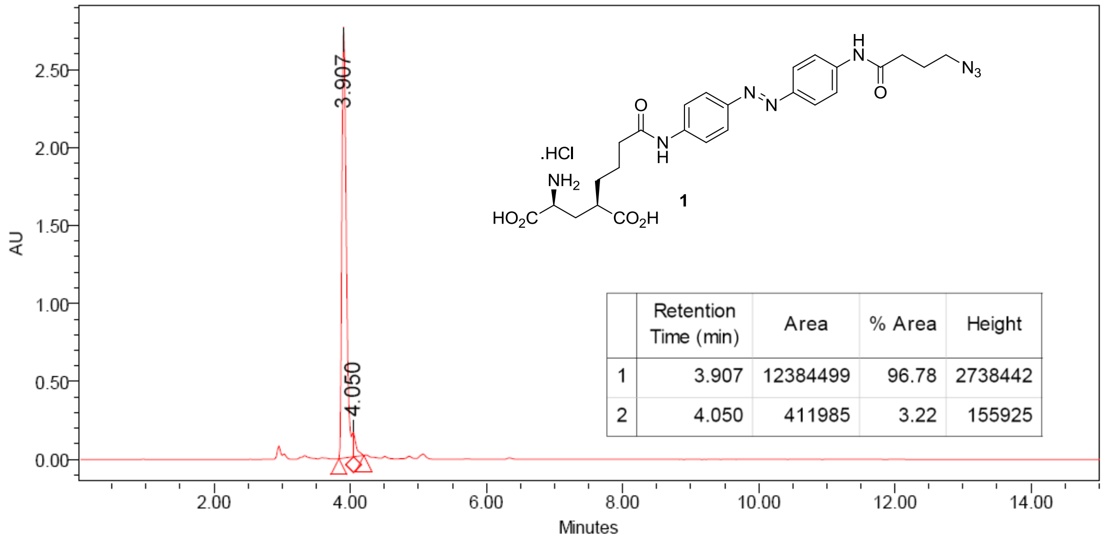

	Retention Time (min)	Area	% Area	Height
1	3.907	12384499	96.78	2738442
2	4.050	411985	3.22	155925

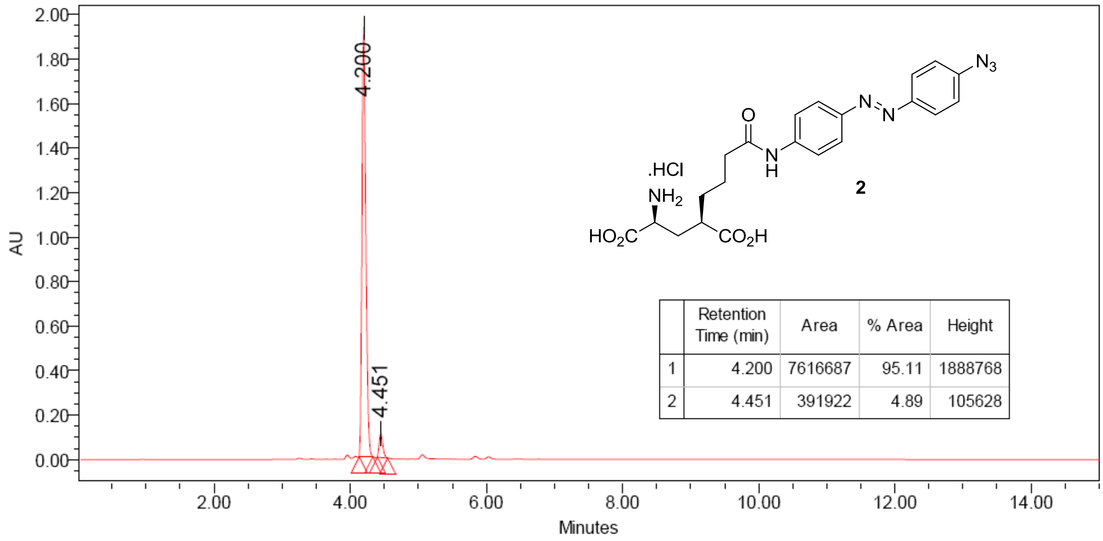

	Retention Time (min)	Area	% Area	Height
1	4.200	7616687	95.11	1888768
2	4.451	391922	4.89	105628

Supplementary Table 1. Summary of the resulting length of each PTL prepared.

		Alkyne molecule			
		**5**	**6**	**7**	**8**
**Azide molecule**	**1**	26 **(13)**	28 **(9)**	30 **(14)**	40 **(15)**
	**2**	21 **(18)**	23 **(19)**	25 **(20)**	35 **(10)**

Compound length in number of bonds (**Compound number**)

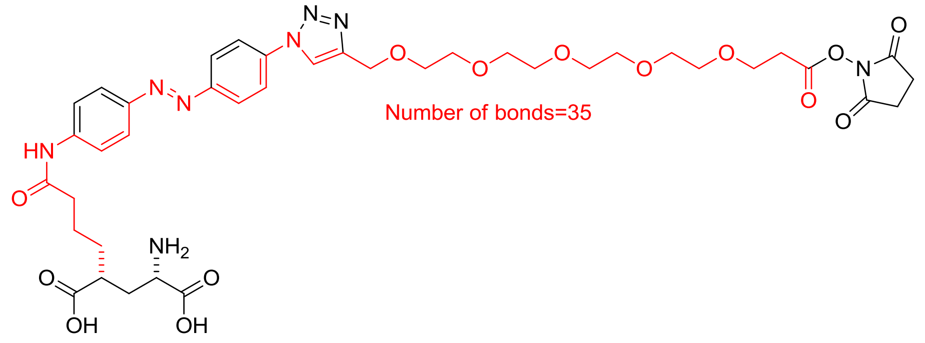

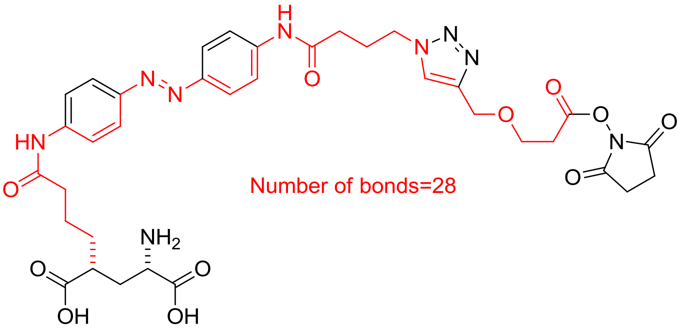

Length is calulated in number of bonds from the reactive carbonyl group to the *C*-4 of glutamate, see the bonds highlighted in red for compounds **10** and **9** (upper and lower structures respectively) in the schemes below the table.

Supplementary Table 2. Theoretical ion fragment tables

Theoretical ion fragment tables of peptides QQSALVK(**9**)NSDEGIQR,b and y ion fragments found in the MS/MS spectra (**Fig. 4b**) are showed in red and blue, respectively.

QQSALVK(**9**)NSDEGIQR

Charge: +3, Monoisotopic m/z: 774.38201 Da (+1.51 mmu/+1.95 ppm), MH+: 2321.13149 Da

Ion series

#1	b⊠	b^{2}⊠	b^{3}⊠	b^{4}⊠	Seq.	y⊠	y^{2}⊠	y^{3}⊠	y^{4}⊠	#2
1	129.06586	65.03657	43.69347	33.02192	Q					15
2	257.12444	129.06586	86.37966	65.03657	Q	2193.06838	1097.03783	731.69431	549.02255	14
3	344.15647	172.58187	115.39034	86.79457	S	2065.00980	1033.00854	689.00812	517.00791	13
4	415.19359	208.10043	139.06938	104.55385	A	1977.97777	989.49252	659.99744	495.24990	12
5	528.27766	264.64247	176.76407	132.82487	L	1906.94065	953.97396	636.31840	477.49062	11
6	627.34608	314.17668	209.78688	157.59198	V	1793.85658	897.43193	598.62371	449.21960	10
7	1403.70665	702.35696	468.57373	351.68212	K-**9**	1694.78816	847.89772	565.60090	424.45250	9
8	1517.74958	759.37843	506.58804	380.19285	N	918.42759	459.71743	306.81405	230.36236	8
9	1604.78161	802.89444	535.59872	401.95086	S	804.38466	402.69597	268.79974	201.85162	7
10	1719.80856	860.40792	573.94104	430.70760	D	717.35263	359.17995	239.78906	180.09362	6
11	1848.85116	924.92922	616.95524	462.96825	E	602.32568	301.66648	201.44674	151.33688	5
12	1905.87263	953.43995	635.96239	477.22361	G	473.28308	237.14518	158.43254	119.07623	4
13	2018.95670	1009.98199	673.65708	505.49463	I	416.26161	208.63444	139.42539	104.82086	3
14	2147.01528	1074.01128	716.34328	537.50928	Q	303.17754	152.09241	101.73070	76.54984	2
15					R	175.11896	88.06312	59.04450	44.53520	1

Neutral losses

#1	b-H_2O⊠	b-H_2O^{2}⊠	b-H_2O^{3}⊠	b-H_2O^{4}⊠	b-NH_3⊠	b-NH_3^{2}⊠	b-NH_3^{3}⊠	b-NH_3^{4}⊠	Seq.	y-H_2O⊠	y-H_2O^{2}⊠	y-H_2O^{3}⊠	y-H_2O^{4}⊠	y-NH_3⊠	y-NH_3^{2}⊠	y-NH_3^{3}⊠	y-NH_3^{4}⊠	#2
1					112.03931	56.52329	38.01795	28.76528	Q									15
2					240.09789	120.55258	80.70415	60.77993	Q	2175.05782	1088.03255	725.69079	544.51991	2176.04183	1088.52455	726.01880	544.76592	14
3	326.14590	163.57659	109.38682	82.29193	327.12992	164.06860	109.71482	82.53794	S	2046.99924	1024.00326	683.00460	512.50527	2047.98325	1024.49526	683.33260	512.75127	13
4	397.18302	199.09515	133.06586	100.05121	398.16704	199.58716	133.39386	100.29722	A	1959.96721	980.48724	653.99392	490.74726	1960.95122	980.97925	654.32193	490.99326	12
5	510.26709	255.63718	170.76055	128.32223	511.25111	256.12919	171.08855	128.56823	L	1888.93009	944.96868	630.31488	472.98798	1889.91410	945.46069	630.64289	473.23398	11
6	609.33551	305.17139	203.78336	153.08934	610.31953	305.66340	204.11136	153.33534	V	1775.84602	888.42665	592.62019	444.71696	1776.83003	888.91865	592.94820	444.96297	10
7	1385.69608	693.35168	462.57021	347.17948	1386.68010	693.84369	462.89822	347.42548	K-**9**	1676.77760	838.89244	559.59738	419.94986	1677.76161	839.38444	559.92539	420.19586	9
8	1499.73901	750.37314	500.58452	375.69021	1500.72303	750.86515	500.91253	375.93621	N	900.41703	450.71215	300.81053	225.85971	901.40104	451.20416	301.13853	226.10572	8
9	1586.77104	793.88916	529.59520	397.44822	1587.75506	794.38117	529.92320	397.69422	S	786.37410	393.69069	262.79622	197.34898	787.35811	394.18269	263.12422	197.59499	7
10	1701.79799	851.40263	567.93752	426.20496	1702.78201	851.89464	568.26552	426.45096	D	699.34207	350.17467	233.78554	175.59097	700.32608	350.66668	234.11355	175.83698	6
11	1830.84059	915.92393	610.95172	458.46561	1831.82461	916.41594	611.27972	458.71161	E	584.31512	292.66120	195.44322	146.83424	585.29913	293.15320	195.77123	147.08024	5
12	1887.86206	944.43467	629.95887	472.72097	1888.84608	944.92668	630.28688	472.96698	G					456.25653	228.63190	152.75703	114.81959	4
13	2000.94613	1000.97670	667.65356	500.99199	2001.93015	1001.46871	667.98157	501.23799	I					399.23506	200.12117	133.74987	100.56422	3
14	2129.00471	1065.00599	710.33976	533.00664	2129.98873	1065.49800	710.66776	533.25264	Q					286.15099	143.57913	96.05518	72.29321	2
15									R					158.09241	79.54984	53.36899	40.27856	1

Precursor ions

[M + 4H]^{4}⊠-H_2O-I	572.27792
[M + 4H]^{4}⊠-NH_3	576.78056
[M + 4H]^{4}⊠-H_2O	576.53456
[M + 4H]^{4}⊠	581.03720

Supplementary Table 3. Theoretical ion fragment tables

Theoretical ion fragment tables of peptides QQSALVK(**10**)NSDEGIQR, b and y ion fragments found in the MS/MS spectra (**Fig. 4b**) are showed in red and blue, respectively.

QQSALVK(**10**)NSDEGIQR

Charge: +4, Monoisotopic m/z: 603.80098 Da (+0.75 mmu/+1.25 ppm), MH+: 2412.18208 Da

Ion series

#1	b⊠	b^{2}⊠	b^{3}⊠	b^{4}⊠	Seq.	y⊠	y^{2}⊠	y^{3}⊠	y^{4}⊠	#2
1	129.06586	65.03657	43.69347	33.02192	Q					15
2	257.12444	129.06586	86.37966	65.03657	Q	2284.12048	1142.56388	762.04501	571.78558	14
3	344.15647	172.58187	115.39034	86.79457	S	2156.06190	1078.53459	719.35882	539.77093	13
4	415.19359	208.10043	139.06938	104.55385	A	2069.02987	1035.01857	690.34814	518.01293	12
5	528.27766	264.64247	176.76407	132.82487	L	1997.99275	999.50001	666.66910	500.25365	11
6	627.34608	314.17668	209.78688	157.59198	V	1884.90868	942.95798	628.97441	471.98263	10
7	1494.75875	747.88301	498.92443	374.44514	K-**10**	1785.84026	893.42377	595.95160	447.21552	9
8	1608.80168	804.90448	536.93874	402.95588	N	918.42759	459.71743	306.81405	230.36236	8
9	1695.83371	848.42049	565.94942	424.71388	S	804.38466	402.69597	268.79974	201.85162	7
10	1810.86066	905.93397	604.29174	453.47062	D	717.35263	359.17995	239.78906	180.09362	6
11	1939.90326	970.45527	647.30594	485.73127	E	602.32568	301.66648	201.44674	151.33688	5
12	1996.92473	998.96600	666.31309	499.98664	G	473.28308	237.14518	158.43254	119.07623	4
13	2110.00880	1055.50804	704.00778	528.25766	I	416.26161	208.63444	139.42539	104.82086	3
14	2238.06738	1119.53733	746.69398	560.27230	Q	303.17754	152.09241	101.73070	76.54984	2
15					R	175.11896	88.06312	59.04450	44.53520	1

Neutral losses

#1	b-H_2O⊠	b-H_2O^{2}⊠	b-H_2O^{3}⊠	b-H_2O^{4}⊠	b-NH_3⊠	b-NH_3^{2}⊠	b-NH_3^{3}⊠	b-NH_3^{4}⊠	Seq.	y-H_2O⊠	y-H_2O^{2}⊠	y-H_2O^{3}⊠	y-H_2O^{4}⊠	y-NH_3⊠	y-NH_3^{2}⊠	y-NH_3^{3}⊠	y-NH_3^{4}⊠	#2
1					112.03931	56.52329	38.01795	28.76528	Q									15
2					240.09789	120.55258	80.70415	60.77993	Q	2266.10992	1133.55860	756.04149	567.28294	2267.09393	1134.05060	756.36950	567.52894	14
3	326.14590	163.57659	109.38682	82.29193	327.12992	164.06860	109.71482	82.53794	S	2138.05134	1069.52931	713.35530	535.26829	2139.03535	1070.02131	713.68330	535.51430	13
4	397.18302	199.09515	133.06586	100.05121	398.16704	199.58716	133.39386	100.29722	A	2051.01931	1026.01329	684.34462	513.51028	2052.00332	1026.50530	684.67263	513.75629	12
5	510.26709	255.63718	170.76055	128.32223	511.25111	256.12919	171.08855	128.56823	L	1979.98219	990.49473	660.66558	495.75100	1980.96620	990.98674	660.99359	495.99701	11
6	609.33551	305.17139	203.78336	153.08934	610.31953	305.66340	204.11136	153.33534	V	1866.89812	933.95270	622.97089	467.47999	1867.88213	934.44470	623.29890	467.72599	10
7	1476.74818	738.87773	492.92091	369.94250	1477.73220	739.36974	493.24892	370.18851	K-**10**	1767.82970	884.41849	589.94808	442.71288	1768.81371	884.91049	590.27609	442.95889	9
8	1590.79111	795.89919	530.93522	398.45324	1591.77513	796.39120	531.26323	398.69924	N	900.41703	450.71215	300.81053	225.85971	901.40104	451.20416	301.13853	226.10572	8
9	1677.82314	839.41521	559.94590	420.21124	1678.80716	839.90722	560.27390	420.45725	S	786.37410	393.69069	262.79622	197.34898	787.35811	394.18269	263.12422	197.59499	7
10	1792.85009	896.92868	598.28822	448.96798	1793.83411	897.42069	598.61622	449.21398	D	699.34207	350.17467	233.78554	175.59097	700.32608	350.66668	234.11355	175.83698	6
11	1921.89269	961.44998	641.30242	481.22863	1922.87671	961.94199	641.63042	481.47463	E	584.31512	292.66120	195.44322	146.83424	585.29913	293.15320	195.77123	147.08024	5
12	1978.91416	989.96072	660.30957	495.48400	1979.89818	990.45273	660.63758	495.73000	G					456.25653	228.63190	152.75703	114.81959	4
13	2091.99823	1046.50275	698.00426	523.75502	2092.98225	1046.99476	698.33227	524.00102	I					399.23506	200.12117	133.74987	100.56422	3
14	2220.05681	1110.53204	740.69046	555.76966	2221.04083	1111.02405	741.01846	556.01566	Q					286.15099	143.57913	96.05518	72.29321	2
15									R					158.09241	79.54984	53.36899	40.27856	1

Precursor ions

$[M + 4H]^{4}$⊠-H_2O-I	595.04094
$[M + 4H]^{4}$⊠-NH_3	599.54359
$[M + 4H]^{4}$⊠-H_2O	599.29758
$[M + 4H]^{4}$⊠	603.80022

Supplementary table 4. Identified peptides

Confidence																					
dark10_01	dark10_02	dark10_03	dark9_01	dark9_02	dark9_03	Sequence	# PSMs	# Proteins	# Protein Groups	Protein Group Accessions	Modifications	MH+ [Da]	q-Value	PEP	XCorr A2	XCorr B2	XCorr C2	XCorr D2	XCorr E2	XCorr F2	# Missed Cleavages
High	High	High	High	High	High	ADLAVAPLTITYVR	234	5	1	sp		1502.85750	0	4.239E-16	3.73	4.22	3.77	3.19	4.20	5.18	0
High		High				ADLAVAPLTITYVREK	2	5	1	sp		1759.99836	0	0.01321	3.92		4.15				1
		High				ADLAVAPLTITYVREK	2	5	1	sp	K16(Comp10)	2499.29927	0	0.04026			1.76				1
High		High				ADLAVAPLTITYVREKVIDFSKPFMTLGISILYR	3	5	1	sp		3841.16470	0	3.632E-09	4.62		3.17				2
High	High	High	High	High	High	DGSTMTFFK	87	5	1	sp	M5(Oxidation)	1049.46148	0	0.0002756	2.33	2.24	2.55	2.37	2.60	2.51	0
High	High	High	High	High	High	DGSTMTFFK	134	5	1	sp		1033.47296	0	0.001573	2.66	2.69	2.85	2.61	2.83	2.71	0
High	High					DGSTMTFFKK	10	5	1	sp	M5(Oxidation)	1177.56034	0.001	0.0134	2.42	2.40					1
High	High	High	High	High	High	DGSTMTFFKK	39	5	1	sp		1161.56129	0	0.00005469	2.69	2.58	2.27	1.58	2.15	2.24	1
	High					DGSTMTFFKK	1	5	1	sp	K10(Comp10)	1900.88508	0.005	0.1132		1.72					1
High	High	High	High	High	High	DKITIAILQLQEEGK	86	6	1	sp		1698.96633	0	0.00001379	5.72	5.24	5.69	2.05	5.52	5.57	1
High	High	High				DKITIAILQLQEEGKLHMMK	20	6	1	sp	M19(Oxidation)	2355.28277	0	4.556E-16	4.59	4.87	4.64				2
High	High	High			High	DKITIAILQLQEEGKLHMMK	22	6	1	sp		2339.28507	0	0.006834	5.65	5.56	5.74			3.92	2
High	High	High				EKVIDFSKPFMTLGISILYR	23	8	1	sp		2357.30577	0	0.002908	4.99	5.13	5.13				1
	High					EKVIDFSKPFMTLGISILYR	3	8	1	sp	M11(Oxidation)	2373.29602	0	9.815E-08		4.22					1
High	High	High				EKVIDFSKPFMTLGISILYRK	18	1	1	sp		2485.39096	0	1.319E-09	3.75	4.79	4.77				2
	High					EKVIDFSKPFMTLGISILYRK	1	1	1	sp	M11(Oxidation)	2501.39394	0	3.358E-08		3.22					2
	High					EKWWRGNGCPE	2	1	1	sp	C9(Carbamidomethyl)	1418.63446	0.002	0.02199		3.37					2
High	High	High		High	High	ELIDHR	12	5	1	sp		782.41680	0.001	0.01177	1.51	1.25	1.18		1.33	1.88	0
High	High	High				ELIDHRADLAVAPLTITYVR	10	5	1	sp		2266.26054	0	0.00001571	4.73	4.74	4.38				1
High	High	High	High	High	High	ELSNILGFLYDVK	205	5	1	sp		1510.82267	0	2.703E-09	4.94	4.73	4.55	4.01	4.95	4.63	0
High		High				ELSNILGFLYDVKLVPDGK	4	5	1	sp	K13(Comp10)	2859.47620	0	0.008997	3.94		2.89				1
	High					ELSNILGFLYDVKLVPDGK	1	5	1	sp		2120.17786	0.001	0.0007667		3.14					1
				High	High	ELSNILGFLYDVKLVPDGK	2	5	1	sp	K13(Comp9)	2768.44120	0	0.02395					2.79	2.80	1
High	High	High	High	High	High	FEGYCLDLLK	68	3	2	sp;P42264-2	C5(Carbamidomethyl)	1257.62052	0	0.0001008	2.32	2.71	2.75	2.84	3.08	2.87	0
High	High	High	High	High	High	GEWNGMVK	34	5	1	sp		920.43001	0	0.0086	2.00	1.90	2.23	2.06	2.14	1.88	0
	High	High	High	High	High	GEWNGMVK	23	5	1	sp	M6(Oxidation)	936.42977	0	0.00429		1.47	1.80	1.91	2.07	1.91	0
High	High	High				GSNRTLIVTTILEEPYVMYR	16	1	1	sp		2355.24661	0	0.0001156	5.30	5.59	5.44				1
	High	High				GSNRTLIVTTILEEPYVMYR	3	1	1	sp	M18(Oxidation)	2371.24044	0	2.707E-07		4.65	3.74				1
High	High	High				GSNRTLIVTTILEEPYVMYRK	8	1	1	sp		2483.33647	0	4.556E-16	6.37	5.63	6.21				2
High	High	High	High	High	High	GTPIDSADDLAK	308	1	1	sp		1202.59009	0	0.05209	2.95	2.63	3.00	2.76	2.65	2.58	0
	High					GTPIDSADDLAKQTK	4	1	1	sp		1559.79172	0	3.019E-10		4.31					1
High	High	High	High	High	High	GYGVGTPIGSPYR	290	5	1	sp		1323.67529	0	0.00001264	3.83	4.02	3.54	3.64	3.68	3.67	0
High	High				High	GYGVGTPIGSPYRDK	21	5	1	sp		1566.79197	0	2.298E-07	3.51	4.09				3.34	1
High	High	High				GYGVGTPIGSPYRDKITIAILQLQEEGK	7	5	1	sp		3003.61490	0	8.758E-09	6.32	6.03	5.51				2
High	High	High	High	High	High	IEYGAVR	32	5	1	sp		807.43676	0.001	0.003186	1.62	1.41	1.39	1.69	1.65	1.63	0
High	High					IEYGAVRDGSTMTFFKK	6	5	1	sp		1949.98370	0	0.00001855	2.02	3.54					2
High	High	High				ISTYEKMWAFMSSR	4	5	1	sp		1736.82393	0	2.646E-10	4.69	4.58	4.12				1
High		High				ISTYEKMWAFMSSRQQSALVK	2	5	1	sp		2491.25253	0	0.0005621	3.75		3.42				2
High	High	High	High	High	High	ITIAILQLQEEGK	107	6	1	sp		1455.84413	0	0.005103	4.08	3.82	4.52	4.99	4.05	4.05	0
High	High					ITIAILQLQEEGKLHMMK	4	6	1	sp	M16(Oxidation); M17(Oxidation)	2128.15724	0	5.884E-08	2.64	2.99					1
High	High	High			High	ITIAILQLQEEGKLHMMK	28	6	1	sp	M17(Oxidation)	2112.15912	0	1.982E-07	3.01	4.30	3.01			3.01	1
High	High	High		High	High	ITIAILQLQEEGKLHMMK	29	6	1	sp		2096.16200	0	2.194E-13	5.76	6.18	5.72		2.01	2.87	1
	High					ITIAILQLQEEGKLHMMKEK	1	6	1	sp		2353.31193	0	8.556E-07		3.98					2
High	High	High	High	High	High	KGTPIDSADDLAK	124	1	1	sp		1330.68354	0	0.00007344	4.19	4.34	4.22	3.75	3.98	4.06	1
High	High	High				KGTPIDSADDLAK	6	1	1	sp	K1(Comp10)	2070.01194	0	0.06269	1.83	2.12	2.00				1
					High	KGTPIDSADDLAK	1	1	1	sp	K1(Comp9)	1978.94558	0.009	0.2934						1.48	1
	High					KGTPIDSADDLAKQTK	2	1	1	sp		1687.88964	0	2.909E-08		4.39					2
High	High	High				KSDKPLYGNDR	6	6	1	sp	K1(Comp10)	2031.97958	0	0.02151	1.86	1.54	2.14				1
			High	High	High	KSDKPLYGNDR	6	6	1	sp	K1(Comp9)	1940.93199	0	0.002085				2.31	2.01	2.14	1
High	High	High		High	High	KSDKPLYGNDRFEGYCLDLLK	25	1	1	sp	C16(Carbamidomethyl)	2531.25690	0	5.351E-10	3.10	3.98	2.95		3.04	3.05	2
		High				KSDKPLYGNDRFEGYCLDLLK	1	1	1	sp	K1(Comp10); C16(Carbamidomethyl)	3270.60038	0.001	0.1229			2.28				2
	High					LVPDGKYGAQNDK	2	5	1	sp		1404.71469	0	0.0002931		1.42					1
	High					LVPDGKYGAQNDK	2	5	1	sp	K6(Comp10)	2144.03389	0.004	0.04662		0.82					1
High	High					LVPDGKYGAQNDKGEWNGMVK	2	5	1	sp	K6(Comp10)	3045.45800	0.003	0.02017	1.57	1.59					2
	High					LVPDGKYGAQNDKGEWNGMVK	2	5	1	sp		2306.12616	0.001	0.0007667		3.92					2
					High	LVPDGKYGAQNDKGEWNGMVK	1	5	1	sp	K6(Comp9)	2954.38212	0	0.02157						1.26	2
High	High	High		High	High	MWAFMSSR	25	6	1	sp	M5(Oxidation)	1031.44534	0	0.001621	2.80	2.65	2.64		2.43	2.14	0
High	High	High				MWAFMSSR	7	6	1	sp	M1(Oxidation); M5(Oxidation)	1047.44160	0.001	0.1118	1.88	1.81	2.01				0
High	High	High	High	High	High	MWAFMSSR	59	6	1	sp		1015.45213	0	0.05748	2.70	2.71	2.81	2.59	2.38	2.67	0
	High					MWAFMSSRQQSALVK	1	5	1	sp		1760.88768	0.001	0.003314		3.21					1
High	High	High	High	High	High	NCNLTQIGGLIDSK	310	8	2	sp;P42264-2	C2(Carbamidomethyl)	1532.77808	0	4.071E-07	5.29	5.48	5.43	5.21	5.22	5.13	0
	High					NCNLTQIGGLIDSKGYGVGTPIGSPYR	2	5	1	sp	C2(Carbamidomethyl)	2837.42351	0	1.071E-10		5.73					1
	High					NCNLTQIGGLIDSKGYGVGTPIGSPYRDK	2	5	1	sp	C2(Carbamidomethyl)	3080.54858	0	0.0007667		5.36					2
High	High	High	High		High	NSDEGIQR	21	5	1	sp		918.42913	0	0.01725	2.38	2.41	2.63	2.26		2.04	0
	High	High				NSDEGIQRVLTTDYALLMFSTSIFYVTQR	4	5	1	sp		3332.65362	0	0.005041		4.12	3.84				1
High		High	High	High	High	QQSALVK	12	5	1	sp		773.45522	0	0.005387	1.55		1.58	1.51	1.61	1.63	0
High	High	High			High	QQSALVKNSDEGIQR	10	5	1	sp		1672.86585	0	0.0005344	2.93	3.42	3.36			2.48	1
High	High	High				QQSALVKNSDEGIQR	5	5	1	sp	K7(Comp10)	2412.18761	0	0.05553	1.68	1.51	1.99				1
			High	High	High	QQSALVKNSDEGIQR	11	5	1	sp	K7(Comp9)	2321.13630	0.001	0.04105				1.62	2.10	1.80	1
High	High	High				QTKIEYGAVR	5	5	1	sp		1164.63904	0	0.0007667	1.96	2.25	1.66				1
High	High	High	High	High	High	SDKPLYGNDR	12	6	1	sp		1164.56687	0	0.0442	1.85	2.26	2.56	2.24	0.60	2.47	0
High	High	High		High	High	SDKPLYGNDRFEGYCLDLLK	36	1	1	sp	C15(Carbamidomethyl)	2403.16669	0	0.0001979	3.49	3.86	3.72		2.77	3.66	1

High	High	High	High	High	High	TLIVTTILEEPYVMYR	106	5	1	sp	M14(Oxidation)	1957.04358	0	0.0002155	4.92	4.70	5.49	3.64	5.10	4.97	0
High	High	High	High	High	High	TLIVTTILEEPYVMYR	292	5	1	sp		1941.05454	0	0.000001102	6.32	5.96	6.79	4.28	5.82	6.27	0
High	High	High				TLIVTTILEEPYVMYRK	16	5	1	sp		2069.13358	0	0.000005528	4.66	5.18	5.01				1
	High					TLIVTTILEEPYVMYRK	4	5	1	sp	M14(Oxidation)	2085.13398	0	4.104E-11		4.11					1
High	High	High		High	High	VIDFSKPFMTLGISILYR	27	8	1	sp	M9(Oxidation)	2116.16918	0	1.264E-09	4.21	4.33	3.96		1.69	3.71	0
High	High	High	High	High	High	VIDFSKPFMTLGISILYR	147	8	1	sp		2100.17426	0	2.238E-11	5.10	5.47	5.22	3.26	5.18	5.00	0
High	High	High				VIDFSKPFMTLGISILYRK	16	1	1	sp		2228.26838	0	1.196E-09	4.08	4.60	3.94				1
	High					VIDFSKPFMTLGISILYRK	3	1	1	sp	M9(Oxidation)	2244.25916	0	5.816E-09		4.58					1
High	High	High				VIDFSKPFMTLGISILYRKGTPIDSADDLAK	8	1	1	sp		3411.84183	0	0.0001552	4.74	5.18	5.52				2
High	High	High	High	High	High	VLTTDYALLMESTSIEYVTQR	84	5	1	sp	M10(Oxidation)	2449.23923	0	4.239E-16	4.97	4.72	5.00	4.09	4.94	5.38	0
High	High	High	High	High	High	VLTTDYALLMESTSIEYVTQR	456	5	1	sp		2433.23876	0	4.575E-14	5.52	5.34	5.39	4.61	5.17	5.13	0
High	High	High				VLTTDYALLMESTSIEYVTQRNCNLTQIGGLIDSK	11	5	1	sp	C23(Carbamidomethyl)	3947.00179	0	4.928E-14	6.26	5.85	4.68				1
	High					WWRGNGCPE	1	1	1	sp	C7(Carbamidomethyl)	1161.49186	0.002	0.02584		2.46					1
		High				YGAQNDK	2	5	1	sp		795.36588	0	0.05781			2.09				0
High	High	High	High	High	High	YGAQNDKGEWNGMVK	121	5	1	sp	M13(Oxidation)	1712.77339	0	0.000001014	4.49	4.53	4.58	3.97	4.21	4.11	1
High	High	High	High	High	High	YGAQNDKGEWNGMVK	132	5	1	sp		1696.77389	0	0.0007667	5.14	5.32	5.14	5.02	5.11	5.19	1
	High	High				YGAQNDKGEWNGMVK	2	5	1	sp	K7(Comp10)	2436.10919	0.003	0.2504		0.83	0.93				1

Supplementary table 5. Selected ions for targeted analysis

Peptide Modified Sequence	Precursor Mz	Precursor Charge	RT 29/03/2016	Time window 29/03/2016
LVPDGKYGAQNDK	702.859523	2	27	25-35
LVPDGKYGAQNDK	468.908774	3	27	25-35
QQSALVKNSDEGIQR	836.934282	2	29	25-35
QQSALVKNSDEGIQR	558.291946	3	29	25-35
LVPDGKYGAQNDKGEWNGMVK	769.379315	3	39.5	35-43
LVPDGKYGAQNDKGEWNGMVK	577.286305	4	39.5	35-43
KSDKPLYGNDRFEGYC[+57]LDLLK	844.424813	3	49.6	48-54
KSDKPLYGNDRFEGYC[+57]LDLLK	633.570429	4	49.6	48-54
QQSALVK[+648.3]NSDEGIQR	1161.06709	2	49.7	48-54
QQSALVK[+648.3]NSDEGIQR	774.380483	3	49.7	48-54
KSDK[+648.3]PLYGNDRFEGYC[+57]LDLLK	1060.51335	3	56.7	54-64
K[+648.3]SDKPLYGNDRFEGYC[+57]LDLLK	1060.51335	3	56.7	54-64
GSNRTLIVTTILEEPYVMYRK	828.44907	3	57.5	54-64
GSNRTLIVTTILEEPYVMYRK	621.588621	4	57.5	54-64
TLIVTTILEEPYVM[+16]YRK	695.714864	3	58	54-64
TLIVTTILEEPYVMYRK	690.383226	3	61.2	54-64

Supplementary table 6. Integrated peak area

Integrated peak areas was based on extracted ion chromatograms (XICs) of up to 3 highest ranked MS/MS fragment ions masses

Begin Pos	End Pos	Peptide Modified Sequence	Precursor Mz	Precursor Charge	Product Mz	Product Charge	Fragment Ion	Retention Time	Height	Area	Peak Rank	File Name	Condition	BioReplicate	TechReplicate
1	21	GSNRTLIVTTILEEPYVMYRK	828.44907	3	1214.587421	1	y9	57.63	1272	14568	2	2311_NC_dark9_01_targeted.RAW	dark	1	1a
1	21	GSNRTLIVTTILEEPYVMYRK	828.44907	3	1214.587421	1	y9	57.84	1138	13747	2	2311_NC_dark9_01_targeted_b.RAW	dark	1	1b
1	21	GSNRTLIVTTILEEPYVMYRK	828.44907	3	1214.587421	1	y9	57.64	2938	32653	2	2311_NC_dark9_02_targeted.RAW	dark	2	2a
1	21	GSNRTLIVTTILEEPYVMYRK	828.44907	3	1214.587421	1	y9	57.72	1045	13384	2	2311_NC_dark9_02_targeted_b.RAW	dark	2	2b
1	21	GSNRTLIVTTILEEPYVMYRK	828.44907	3	1214.587421	1	y9	57.71	4104	45899	2	2311_NC_dark9_03_targeted.RAW	dark	3	3a
1	21	GSNRTLIVTTILEEPYVMYRK	828.44907	3	1214.587421	1	y9	57.77	3463	42625	2	2311_NC_dark9_03_targeted_b.RAW	dark	3	3b
1	21	GSNRTLIVTTILEEPYVMYRK	828.44907	3	1214.587421	1	y9	57.62	7402	101579	2	2311_NC_UV9_04_targeted.RAW	UV	4	4a
1	21	GSNRTLIVTTILEEPYVMYRK	828.44907	3	1214.587421	1	y9	57.92	4452	56001	2	2311_NC_UV9_04_targeted_b.RAW	UV	4	4b
1	21	GSNRTLIVTTILEEPYVMYRK	828.44907	3	1214.587421	1	y9	57.73	4965	65261	2	2311_NC_UV9_05_targeted.RAW	UV	5	5a
1	21	GSNRTLIVTTILEEPYVMYRK	828.44907	3	1214.587421	1	y9	57.91	3639	40962	2	2311_NC_UV9_05_targeted_b.RAW	UV	5	5b
1	21	GSNRTLIVTTILEEPYVMYRK	828.44907	3	1214.587421	1	y9	57.8	6875	66715	2	2311_NC_UV9_06_targeted.RAW	UV	6	6a
1	21	GSNRTLIVTTILEEPYVMYRK	828.44907	3	1214.587421	1	y9	57.78	2957	40044	2	2311_NC_UV9_06_targeted_b.RAW	UV	6	6b
1	21	GSNRTLIVTTILEEPYVMYRK	828.44907	3	1085.544828	1	y8	57.63	1111	12514	3	2311_NC_dark9_01_targeted.RAW	dark	1	1a
1	21	GSNRTLIVTTILEEPYVMYRK	828.44907	3	1085.544828	1	y8	57.84	823	10342	3	2311_NC_dark9_01_targeted_b.RAW	dark	1	1b
1	21	GSNRTLIVTTILEEPYVMYRK	828.44907	3	1085.544828	1	y8	57.64	2434	27593	3	2311_NC_dark9_02_targeted.RAW	dark	2	2a
1	21	GSNRTLIVTTILEEPYVMYRK	828.44907	3	1085.544828	1	y8	57.72	915	11636	3	2311_NC_dark9_02_targeted_b.RAW	dark	2	2b
1	21	GSNRTLIVTTILEEPYVMYRK	828.44907	3	1085.544828	1	y8	57.71	3675	38778	3	2311_NC_dark9_03_targeted.RAW	dark	3	3a
1	21	GSNRTLIVTTILEEPYVMYRK	828.44907	3	1085.544828	1	y8	57.77	2813	33942	3	2311_NC_dark9_03_targeted_b.RAW	dark	3	3b
1	21	GSNRTLIVTTILEEPYVMYRK	828.44907	3	1085.544828	1	y8	57.62	6603	85925	3	2311_NC_UV9_04_targeted.RAW	UV	4	4a
1	21	GSNRTLIVTTILEEPYVMYRK	828.44907	3	1085.544828	1	y8	57.92	3652	46422	3	2311_NC_UV9_04_targeted_b.RAW	UV	4	4b
1	21	GSNRTLIVTTILEEPYVMYRK	828.44907	3	1085.544828	1	y8	57.67	4453	57228	3	2311_NC_UV9_05_targeted.RAW	UV	5	5a
1	21	GSNRTLIVTTILEEPYVMYRK	828.44907	3	1085.544828	1	y8	57.91	3110	36309	3	2311_NC_UV9_05_targeted_b.RAW	UV	5	5b
1	21	GSNRTLIVTTILEEPYVMYRK	828.44907	3	1085.544828	1	y8	57.8	5973	57306	3	2311_NC_UV9_06_targeted.RAW	UV	6	6a
1	21	GSNRTLIVTTILEEPYVMYRK	828.44907	3	1085.544828	1	y8	57.84	2497	33403	3	2311_NC_UV9_06_targeted_b.RAW	UV	6	6b
1	21	GSNRTLIVTTILEEPYVMYRK	828.44907	3	956.502234	1	y7	57.63	3144	38882	1	2311_NC_dark9_01_targeted.RAW	dark	1	1a
1	21	GSNRTLIVTTILEEPYVMYRK	828.44907	3	956.502234	1	y7	57.84	2830	34909	1	2311_NC_dark9_01_targeted_b.RAW	dark	1	1b
1	21	GSNRTLIVTTILEEPYVMYRK	828.44907	3	956.502234	1	y7	57.64	7440	86250	1	2311_NC_dark9_02_targeted.RAW	dark	2	2a
1	21	GSNRTLIVTTILEEPYVMYRK	828.44907	3	956.502234	1	y7	57.78	3197	39013	1	2311_NC_dark9_02_targeted_b.RAW	dark	2	2b
1	21	GSNRTLIVTTILEEPYVMYRK	828.44907	3	956.502234	1	y7	57.71	11274	124985	1	2311_NC_dark9_03_targeted.RAW	dark	3	3a
1	21	GSNRTLIVTTILEEPYVMYRK	828.44907	3	956.502234	1	y7	57.77	8883	111153	1	2311_NC_dark9_03_targeted_b.RAW	dark	3	3b
1	21	GSNRTLIVTTILEEPYVMYRK	828.44907	3	956.502234	1	y7	57.62	21112	274433	1	2311_NC_UV9_04_targeted.RAW	UV	4	4a
1	21	GSNRTLIVTTILEEPYVMYRK	828.44907	3	956.502234	1	y7	57.92	12311	151349	1	2311_NC_UV9_04_targeted_b.RAW	UV	4	4b
1	21	GSNRTLIVTTILEEPYVMYRK	828.44907	3	956.502234	1	y7	57.67	13035	176371	1	2311_NC_UV9_05_targeted.RAW	UV	5	5a
1	21	GSNRTLIVTTILEEPYVMYRK	828.44907	3	956.502234	1	y7	57.91	9464	112397	1	2311_NC_UV9_05_targeted_b.RAW	UV	5	5b
1	21	GSNRTLIVTTILEEPYVMYRK	828.44907	3	956.502234	1	y7	57.8	18596	177993	1	2311_NC_UV9_06_targeted.RAW	UV	6	6a
1	21	GSNRTLIVTTILEEPYVMYRK	828.44907	3	956.502234	1	y7	57.78	7812	103301	1	2311_NC_UV9_06_targeted_b.RAW	UV	6	6b
1	21	GSNRTLIVTTILEEPYVMYRK	621.588621	4	771.405252	2	y12	57.69	649	8722	3	2311_NC_dark9_01_targeted.RAW	dark	1	1a
1	21	GSNRTLIVTTILEEPYVMYRK	621.588621	4	771.405252	2	y12	57.84	331	4426	3	2311_NC_dark9_01_targeted_b.RAW	dark	1	1b
1	21	GSNRTLIVTTILEEPYVMYRK	621.588621	4	771.405252	2	y12	57.58	897	15655	3	2311_NC_dark9_02_targeted.RAW	dark	2	2a
1	21	GSNRTLIVTTILEEPYVMYRK	621.588621	4	771.405252	2	y12	57.78	414	5545	3	2311_NC_dark9_02_targeted_b.RAW	dark	2	2b
1	21	GSNRTLIVTTILEEPYVMYRK	621.588621	4	771.405252	2	y12	57.65	1054	16162	3	2311_NC_dark9_03_targeted.RAW	dark	3	3a
1	21	GSNRTLIVTTILEEPYVMYRK	621.588621	4	771.405252	2	y12	57.83	822	12423	3	2311_NC_dark9_03_targeted_b.RAW	dark	3	3b
1	21	GSNRTLIVTTILEEPYVMYRK	621.588621	4	771.405252	2	y12	57.68	807	15799	3	2311_NC_UV9_04_targeted.RAW	UV	4	4a
1	21	GSNRTLIVTTILEEPYVMYRK	621.588621	4	771.405252	2	y12	57.98	819	12725	3	2311_NC_UV9_04_targeted_b.RAW	UV	4	4b
1	21	GSNRTLIVTTILEEPYVMYRK	621.588621	4	771.405252	2	y12	57.73	798	15922	3	2311_NC_UV9_05_targeted.RAW	UV	5	5a
1	21	GSNRTLIVTTILEEPYVMYRK	621.588621	4	771.405252	2	y12	57.91	742	10897	3	2311_NC_UV9_05_targeted_b.RAW	UV	5	5b
1	21	GSNRTLIVTTILEEPYVMYRK	621.588621	4	771.405252	2	y12	57.85	792	11976	3	2311_NC_UV9_06_targeted.RAW	UV	6	6a
1	21	GSNRTLIVTTILEEPYVMYRK	621.588621	4	771.405252	2	y12	57.78	617	9554	3	2311_NC_UV9_06_targeted_b.RAW	UV	6	6b
1	21	GSNRTLIVTTILEEPYVMYRK	621.588621	4	720.881412	2	y11	57.69	1037	14126	2	2311_NC_dark9_01_targeted.RAW	dark	1	1a
1	21	GSNRTLIVTTILEEPYVMYRK	621.588621	4	720.881412	2	y11	57.84	448	6353	2	2311_NC_dark9_01_targeted_b.RAW	dark	1	1b
1	21	GSNRTLIVTTILEEPYVMYRK	621.588621	4	720.881412	2	y11	57.64	1535	24991	2	2311_NC_dark9_02_targeted.RAW	dark	2	2a
1	21	GSNRTLIVTTILEEPYVMYRK	621.588621	4	720.881412	2	y11	57.78	546	6944	2	2311_NC_dark9_02_targeted_b.RAW	dark	2	2b
1	21	GSNRTLIVTTILEEPYVMYRK	621.588621	4	720.881412	2	y11	57.65	1342	23207	2	2311_NC_dark9_03_targeted.RAW	dark	3	3a
1	21	GSNRTLIVTTILEEPYVMYRK	621.588621	4	720.881412	2	y11	57.77	1081	16462	2	2311_NC_dark9_03_targeted_b.RAW	dark	3	3b
1	21	GSNRTLIVTTILEEPYVMYRK	621.588621	4	720.881412	2	y11	57.68	1238	24482	2	2311_NC_UV9_04_targeted.RAW	UV	4	4a
1	21	GSNRTLIVTTILEEPYVMYRK	621.588621	4	720.881412	2	y11	57.98	1150	18607	2	2311_NC_UV9_04_targeted_b.RAW	UV	4	4b
1	21	GSNRTLIVTTILEEPYVMYRK	621.588621	4	720.881412	2	y11	57.73	1373	26432	2	2311_NC_UV9_05_targeted.RAW	UV	5	5a

1	21	GSNRTLIVTTILEEPYVMYRK	621.588621	4	720.881412	2	y11	57.91	1027	15905	2	2311_NC_UV9_05_targeted_b.RAW	UV	5	5b
1	21	GSNRTLIVTTILEEPYVMYRK	621.588621	4	720.881412	2	y11	57.85	1099	17917	2	2311_NC_UV9_06_targeted.RAW	UV	6	6a
1	21	GSNRTLIVTTILEEPYVMYRK	621.588621	4	720.881412	2	y11	57.78	849	14089	2	2311_NC_UV9_06_targeted_b.RAW	UV	6	6b
1	21	GSNRTLIVTTILEEPYVMYRK	621.588621	4	664.33938	2	y10	57.63	3093	42389	1	2311_NC_dark9_01_targeted.RAW	dark	1	1a
1	21	GSNRTLIVTTILEEPYVMYRK	621.588621	4	664.33938	2	y10	57.78	1762	24151	1	2311_NC_dark9_01_targeted_b.RAW	dark	1	1b
1	21	GSNRTLIVTTILEEPYVMYRK	621.588621	4	664.33938	2	y10	57.64	4680	80748	1	2311_NC_dark9_02_targeted.RAW	dark	2	2a
1	21	GSNRTLIVTTILEEPYVMYRK	621.588621	4	664.33938	2	y10	57.78	1777	24523	1	2311_NC_dark9_02_targeted_b.RAW	dark	2	2b
1	21	GSNRTLIVTTILEEPYVMYRK	621.588621	4	664.33938	2	y10	57.71	4222	77758	1	2311_NC_dark9_03_targeted.RAW	dark	3	3a
1	21	GSNRTLIVTTILEEPYVMYRK	621.588621	4	664.33938	2	y10	57.77	4185	55702	1	2311_NC_dark9_03_targeted_b.RAW	dark	3	3b
1	21	GSNRTLIVTTILEEPYVMYRK	621.588621	4	664.33938	2	y10	57.68	3510	74262	1	2311_NC_UV9_04_targeted.RAW	UV	4	4a
1	21	GSNRTLIVTTILEEPYVMYRK	621.588621	4	664.33938	2	y10	57.98	3811	58782	1	2311_NC_UV9_04_targeted_b.RAW	UV	4	4b
1	21	GSNRTLIVTTILEEPYVMYRK	621.588621	4	664.33938	2	y10	57.73	3930	78739	1	2311_NC_UV9_05_targeted.RAW	UV	5	5a
1	21	GSNRTLIVTTILEEPYVMYRK	621.588621	4	664.33938	2	y10	57.91	3697	55512	1	2311_NC_UV9_05_targeted_b.RAW	UV	5	5b
1	21	GSNRTLIVTTILEEPYVMYRK	621.588621	4	664.33938	2	y10	57.85	3624	56817	1	2311_NC_UV9_06_targeted.RAW	UV	6	6a
1	21	GSNRTLIVTTILEEPYVMYRK	621.588621	4	664.33938	2	y10	57.78	3579	47944	1	2311_NC_UV9_06_targeted_b.RAW	UV	6	6b
5	21	TLIVTTILEEPYVMYRK	690.383226	3	928.00533	2	y15	61.04	25297	232640	1	2311_NC_dark9_01_targeted.RAW	dark	1	1a
5	21	TLIVTTILEEPYVMYRK	690.383226	3	928.00533	2	y15	61.37	11666	81037	1	2311_NC_dark9_01_targeted_b.RAW	dark	1	1b
5	21	TLIVTTILEEPYVMYRK	690.383226	3	928.00533	2	y15	61.34	84947	1137854	1	2311_NC_dark9_02_targeted.RAW	dark	2	2a
5	21	TLIVTTILEEPYVMYRK	690.383226	3	928.00533	2	y15	61.42	40289	583050	1	2311_NC_dark9_02_targeted_b.RAW	dark	2	2b
5	21	TLIVTTILEEPYVMYRK	690.383226	3	928.00533	2	y15	61.35	57990	1316063	1	2311_NC_dark9_03_targeted.RAW	dark	3	3a
5	21	TLIVTTILEEPYVMYRK	690.383226	3	928.00533	2	y15	61.47	62251	1087625	1	2311_NC_dark9_03_targeted_b.RAW	dark	3	3b
5	21	TLIVTTILEEPYVMYRK	690.383226	3	928.00533	2	y15	61.26	298437	3391925	1	2311_NC_UV9_04_targeted.RAW	UV	4	4a
5	21	TLIVTTILEEPYVMYRK	690.383226	3	928.00533	2	y15	61.74	44567	959078	1	2311_NC_UV9_04_targeted_b.RAW	UV	4	4b
5	21	TLIVTTILEEPYVMYRK	690.383226	3	928.00533	2	y15	61.37	63846	1467004	1	2311_NC_UV9_05_targeted.RAW	UV	5	5a
5	21	TLIVTTILEEPYVMYRK	690.383226	3	928.00533	2	y15	61.73	45383	864570	1	2311_NC_UV9_05_targeted_b.RAW	UV	5	5b
5	21	TLIVTTILEEPYVMYRK	690.383226	3	928.00533	2	y15	61.56	34631	741872	1	2311_NC_UV9_06_targeted.RAW	UV	6	6a
5	21	TLIVTTILEEPYVMYRK	690.383226	3	928.00533	2	y15	61.78	37107	608802	1	2311_NC_UV9_06_targeted_b.RAW	UV	6	6b
5	21	TLIVTTILEEPYVMYRK	690.383226	3	871.463298	2	y14	61.04	10908	101500	2	2311_NC_dark9_01_targeted.RAW	dark	1	1a
5	21	TLIVTTILEEPYVMYRK	690.383226	3	871.463298	2	y14	61.37	4882	34028	2	2311_NC_dark9_01_targeted_b.RAW	dark	1	1b
5	21	TLIVTTILEEPYVMYRK	690.383226	3	871.463298	2	y14	61.28	34982	553185	2	2311_NC_dark9_02_targeted.RAW	dark	2	2a
5	21	TLIVTTILEEPYVMYRK	690.383226	3	871.463298	2	y14	61.42	18039	252281	2	2311_NC_dark9_02_targeted_b.RAW	dark	2	2b
5	21	TLIVTTILEEPYVMYRK	690.383226	3	871.463298	2	y14	61.3	56595	725520	2	2311_NC_dark9_03_targeted.RAW	dark	3	3a
5	21	TLIVTTILEEPYVMYRK	690.383226	3	871.463298	2	y14	61.47	26013	463610	2	2311_NC_dark9_03_targeted_b.RAW	dark	3	3b
5	21	TLIVTTILEEPYVMYRK	690.383226	3	871.463298	2	y14	61.32	79179	870697	2	2311_NC_UV9_04_targeted.RAW	UV	4	4a
5	21	TLIVTTILEEPYVMYRK	690.383226	3	871.463298	2	y14	61.74	19438	474791	2	2311_NC_UV9_04_targeted_b.RAW	UV	4	4b
5	21	TLIVTTILEEPYVMYRK	690.383226	3	871.463298	2	y14	61.37	70701	862559	2	2311_NC_UV9_05_targeted.RAW	UV	5	5a
5	21	TLIVTTILEEPYVMYRK	690.383226	3	871.463298	2	y14	61.73	19359	376288	2	2311_NC_UV9_05_targeted_b.RAW	UV	5	5b
5	21	TLIVTTILEEPYVMYRK	690.383226	3	871.463298	2	y14	61.56	40622	487880	2	2311_NC_UV9_06_targeted.RAW	UV	6	6a
5	21	TLIVTTILEEPYVMYRK	690.383226	3	871.463298	2	y14	61.78	16795	270982	2	2311_NC_UV9_06_targeted_b.RAW	UV	6	6b
5	21	TLIVTTILEEPYVMYRK	690.383226	3	821.929091	2	y13	61.04	6291	56872	3	2311_NC_dark9_01_targeted.RAW	dark	1	1a
5	21	TLIVTTILEEPYVMYRK	690.383226	3	821.929091	2	y13	61.37	3195	19367	3	2311_NC_dark9_01_targeted_b.RAW	dark	1	1b
5	21	TLIVTTILEEPYVMYRK	690.383226	3	821.929091	2	y13	61.28	18921	265316	3	2311_NC_dark9_02_targeted.RAW	dark	2	2a
5	21	TLIVTTILEEPYVMYRK	690.383226	3	821.929091	2	y13	61.42	9628	138516	3	2311_NC_dark9_02_targeted_b.RAW	dark	2	2b
5	21	TLIVTTILEEPYVMYRK	690.383226	3	821.929091	2	y13	61.3	29846	391916	3	2311_NC_dark9_03_targeted.RAW	dark	3	3a
5	21	TLIVTTILEEPYVMYRK	690.383226	3	821.929091	2	y13	61.47	13821	248994	3	2311_NC_dark9_03_targeted_b.RAW	dark	3	3b
5	21	TLIVTTILEEPYVMYRK	690.383226	3	821.929091	2	y13	61.32	40750	479001	3	2311_NC_UV9_04_targeted.RAW	UV	4	4a
5	21	TLIVTTILEEPYVMYRK	690.383226	3	821.929091	2	y13	61.74	10545	247707	3	2311_NC_UV9_04_targeted_b.RAW	UV	4	4b
5	21	TLIVTTILEEPYVMYRK	690.383226	3	821.929091	2	y13	61.37	38839	507968	3	2311_NC_UV9_05_targeted.RAW	UV	5	5a
5	21	TLIVTTILEEPYVMYRK	690.383226	3	821.929091	2	y13	61.73	10661	200875	3	2311_NC_UV9_05_targeted_b.RAW	UV	5	5b
5	21	TLIVTTILEEPYVMYRK	690.383226	3	821.929091	2	y13	61.56	21988	315982	3	2311_NC_UV9_06_targeted.RAW	UV	6	6a
5	21	TLIVTTILEEPYVMYRK	690.383226	3	821.929091	2	y13	61.78	9226	147913	3	2311_NC_UV9_06_targeted_b.RAW	UV	6	6b
5	21	TLIVTTILEEPYVM[+16]YRK	695.714864	3	936.002787	2	y15	58.19	1617	39974	1	2311_NC_dark9_01_targeted.RAW	dark	1	1a
5	21	TLIVTTILEEPYVM[+16]YRK	695.714864	3	936.002787	2	y15	58.28	702	18168	1	2311_NC_dark9_01_targeted_b.RAW	dark	1	1b
5	21	TLIVTTILEEPYVM[+16]YRK	695.714864	3	936.002787	2	y15	58.13	4561	136948	1	2311_NC_dark9_02_targeted.RAW	dark	2	2a
5	21	TLIVTTILEEPYVM[+16]YRK	695.714864	3	936.002787	2	y15	58.22	1766	47604	1	2311_NC_dark9_02_targeted_b.RAW	dark	2	2b
5	21	TLIVTTILEEPYVM[+16]YRK	695.714864	3	936.002787	2	y15	58.5	6967	218467	1	2311_NC_dark9_03_targeted.RAW	dark	3	3a
5	21	TLIVTTILEEPYVM[+16]YRK	695.714864	3	936.002787	2	y15	58.32	3688	119735	1	2311_NC_dark9_03_targeted_b.RAW	dark	3	3b
5	21	TLIVTTILEEPYVM[+16]YRK	695.714864	3	936.002787	2	y15	58.17	7759	260715	1	2311_NC_UV9_04_targeted.RAW	UV	4	4a
5	21	TLIVTTILEEPYVM[+16]YRK	695.714864	3	936.002787	2	y15	58.47	4270	78558	1	2311_NC_UV9_04_targeted_b.RAW	UV	4	4b
5	21	TLIVTTILEEPYVM[+16]YRK	695.714864	3	936.002787	2	y15	58.23	5167	176343	1	2311_NC_UV9_05_targeted.RAW	UV	5	5a
5	21	TLIVTTILEEPYVM[+16]YRK	695.714864	3	936.002787	2	y15	58.46	3354	87363	1	2311_NC_UV9_05_targeted_b.RAW	UV	5	5b
5	21	TLIVTTILEEPYVM[+16]YRK	695.714864	3	936.002787	2	y15	58.41	4842	123441	1	2311_NC_UV9_06_targeted.RAW	UV	6	6a
5	21	TLIVTTILEEPYVM[+16]YRK	695.714864	3	936.002787	2	y15	58.33	3014	63480	1	2311_NC_UV9_06_targeted_b.RAW	UV	6	6b
5	21	TLIVTTILEEPYVM[+16]YRK	695.714864	3	879.460755	2	y14	58.13	844	16713	2	2311_NC_dark9_01_targeted.RAW	dark	1	1a

5	21	TLIVTTILEEPYVM[+16]YRK	695.714864	3	879.460755	2	y14	58.28	290	8885	2	2311_NC_dark9_01_targeted_b.RAW	dark	1	1b
5	21	TLIVTTILEEPYVM[+16]YRK	695.714864	3	879.460755	2	y14	58.13	1822	59116	2	2311_NC_dark9_02_targeted.RAW	dark	2	2a
5	21	TLIVTTILEEPYVM[+16]YRK	695.714864	3	879.460755	2	y14	58.22	927	20063	2	2311_NC_dark9_02_targeted_b.RAW	dark	2	2b
5	21	TLIVTTILEEPYVM[+16]YRK	695.714864	3	879.460755	2	y14	58.5	3018	94755	2	2311_NC_dark9_03_targeted.RAW	dark	3	3a
5	21	TLIVTTILEEPYVM[+16]YRK	695.714864	3	879.460755	2	y14	58.56	1599	53737	2	2311_NC_dark9_03_targeted_b.RAW	dark	3	3b
5	21	TLIVTTILEEPYVM[+16]YRK	695.714864	3	879.460755	2	y14	58.11	3339	112116	2	2311_NC_UV9_04_targeted.RAW	UV	4	4a
5	21	TLIVTTILEEPYVM[+16]YRK	695.714864	3	879.460755	2	y14	58.47	1904	34803	2	2311_NC_UV9_04_targeted_b.RAW	UV	4	4b
5	21	TLIVTTILEEPYVM[+16]YRK	695.714864	3	879.460755	2	y14	58.23	2149	75143	2	2311_NC_UV9_05_targeted.RAW	UV	5	5a
5	21	TLIVTTILEEPYVM[+16]YRK	695.714864	3	879.460755	2	y14	58.46	1401	41039	2	2311_NC_UV9_05_targeted_b.RAW	UV	5	5b
5	21	TLIVTTILEEPYVM[+16]YRK	695.714864	3	879.460755	2	y14	58.41	1980	52411	2	2311_NC_UV9_06_targeted.RAW	UV	6	6a
5	21	TLIVTTILEEPYVM[+16]YRK	695.714864	3	879.460755	2	y14	58.33	1237	25918	2	2311_NC_UV9_06_targeted_b.RAW	UV	6	6b
5	21	TLIVTTILEEPYVM[+16]YRK	695.714864	3	829.926548	2	y13	58.13	420	9085	3	2311_NC_dark9_01_targeted.RAW	dark	1	1a
5	21	TLIVTTILEEPYVM[+16]YRK	695.714864	3	829.926548	2	y13	58.28	118	4023	3	2311_NC_dark9_01_targeted_b.RAW	dark	1	1b
5	21	TLIVTTILEEPYVM[+16]YRK	695.714864	3	829.926548	2	y13	58.13	1154	31503	3	2311_NC_dark9_02_targeted.RAW	dark	2	2a
5	21	TLIVTTILEEPYVM[+16]YRK	695.714864	3	829.926548	2	y13	58.22	581	11233	3	2311_NC_dark9_02_targeted_b.RAW	dark	2	2b
5	21	TLIVTTILEEPYVM[+16]YRK	695.714864	3	829.926548	2	y13	58.44	1594	53349	3	2311_NC_dark9_03_targeted.RAW	dark	3	3a
5	21	TLIVTTILEEPYVM[+16]YRK	695.714864	3	829.926548	2	y13	58.56	859	27627	3	2311_NC_dark9_03_targeted_b.RAW	dark	3	3b
5	21	TLIVTTILEEPYVM[+16]YRK	695.714864	3	829.926548	2	y13	58.17	1728	62278	3	2311_NC_UV9_04_targeted.RAW	UV	4	4a
5	21	TLIVTTILEEPYVM[+16]YRK	695.714864	3	829.926548	2	y13	58.47	849	17582	3	2311_NC_UV9_04_targeted_b.RAW	UV	4	4b
5	21	TLIVTTILEEPYVM[+16]YRK	695.714864	3	829.926548	2	y13	58.23	1319	37004	3	2311_NC_UV9_05_targeted.RAW	UV	5	5a
5	21	TLIVTTILEEPYVM[+16]YRK	695.714864	3	829.926548	2	y13	58.46	658	20245	3	2311_NC_UV9_05_targeted_b.RAW	UV	5	5b
5	21	TLIVTTILEEPYVM[+16]YRK	695.714864	3	829.926548	2	y13	58.41	999	28197	3	2311_NC_UV9_06_targeted.RAW	UV	6	6a
5	21	TLIVTTILEEPYVM[+16]YRK	695.714864	3	829.926548	2	y13	58.33	722	15285	3	2311_NC_UV9_06_targeted_b.RAW	UV	6	6b
21	31	K[+648.3]SDKPLYGNDR	970.966113	2	1164.564377	1	y10	46.41	188	1659	3	2311_NC_dark9_01_targeted.RAW	dark	1	1a
21	31	K[+648.3]SDKPLYGNDR	970.966113	2	1164.564377	1	y10	46.12	322	3437	3	2311_NC_dark9_01_targeted_b.RAW	dark	1	1b
21	31	K[+648.3]SDKPLYGNDR	970.966113	2	1164.564377	1	y10	46.42	69	586	3	2311_NC_dark9_02_targeted.RAW	dark	2	2a
21	31	K[+648.3]SDKPLYGNDR	970.966113	2	1164.564377	1	y10	46.48	68	795	3	2311_NC_dark9_02_targeted_b.RAW	dark	2	2b
21	31	K[+648.3]SDKPLYGNDR	970.966113	2	1164.564377	1	y10	46.22	85	1018	3	2311_NC_dark9_03_targeted.RAW	dark	3	3a
21	31	K[+648.3]SDKPLYGNDR	970.966113	2	1164.564377	1	y10	46.27	204	2265	3	2311_NC_dark9_03_targeted_b.RAW	dark	3	3b
21	31	K[+648.3]SDKPLYGNDR	970.966113	2	1164.564377	1	y10	46.33	173	1428	3	2311_NC_UV9_04_targeted.RAW	UV	4	4a
21	31	K[+648.3]SDKPLYGNDR	970.966113	2	1164.564377	1	y10	46.26	117	1258	3	2311_NC_UV9_04_targeted_b.RAW	UV	4	4b
21	31	K[+648.3]SDKPLYGNDR	970.966113	2	1164.564377	1	y10	46.34	64	668	3	2311_NC_UV9_05_targeted.RAW	UV	5	5a
21	31	K[+648.3]SDKPLYGNDR	970.966113	2	1164.564377	1	y10	46.52	100	687	3	2311_NC_UV9_05_targeted_b.RAW	UV	5	5b
21	31	K[+648.3]SDKPLYGNDR	970.966113	2	1164.564377	1	y10	46.57	83	774	3	2311_NC_UV9_06_targeted.RAW	UV	6	6a
21	31	K[+648.3]SDKPLYGNDR	970.966113	2	1164.564377	1	y10	46.29	70	581	3	2311_NC_UV9_06_targeted_b.RAW	UV	6	6b
21	31	K[+648.3]SDKPLYGNDR	970.966113	2	1077.532349	1	y9	46.41	1044	9519	2	2311_NC_dark9_01_targeted.RAW	dark	1	1a
21	31	K[+648.3]SDKPLYGNDR	970.966113	2	1077.532349	1	y9	46.07	1621	17486	2	2311_NC_dark9_01_targeted_b.RAW	dark	1	1b
21	31	K[+648.3]SDKPLYGNDR	970.966113	2	1077.532349	1	y9	46.4	312	3730	2	2311_NC_dark9_02_targeted.RAW	dark	2	2a
21	31	K[+648.3]SDKPLYGNDR	970.966113	2	1077.532349	1	y9	46.46	393	4364	2	2311_NC_dark9_02_targeted_b.RAW	dark	2	2b
21	31	K[+648.3]SDKPLYGNDR	970.966113	2	1077.532349	1	y9	46.22	365	4391	2	2311_NC_dark9_03_targeted.RAW	dark	3	3a
21	31	K[+648.3]SDKPLYGNDR	970.966113	2	1077.532349	1	y9	46.25	1220	13757	2	2311_NC_dark9_03_targeted_b.RAW	dark	3	3b
21	31	K[+648.3]SDKPLYGNDR	970.966113	2	1077.532349	1	y9	46.33	832	9189	2	2311_NC_UV9_04_targeted.RAW	UV	4	4a
21	31	K[+648.3]SDKPLYGNDR	970.966113	2	1077.532349	1	y9	46.26	597	6997	2	2311_NC_UV9_04_targeted_b.RAW	UV	4	4b
21	31	K[+648.3]SDKPLYGNDR	970.966113	2	1077.532349	1	y9	46.3	314	3808	2	2311_NC_UV9_05_targeted.RAW	UV	5	5a
21	31	K[+648.3]SDKPLYGNDR	970.966113	2	1077.532349	1	y9	46.47	413	4788	2	2311_NC_UV9_05_targeted_b.RAW	UV	5	5b
21	31	K[+648.3]SDKPLYGNDR	970.966113	2	1077.532349	1	y9	46.5	445	4069	2	2311_NC_UV9_06_targeted.RAW	UV	6	6a
21	31	K[+648.3]SDKPLYGNDR	970.966113	2	1077.532349	1	y9	46.29	337	2874	2	2311_NC_UV9_06_targeted_b.RAW	UV	6	6b
21	31	K[+648.3]SDKPLYGNDR	970.966113	2	962.505406	1	y8	46.44	4100	45518	1	2311_NC_dark9_01_targeted.RAW	dark	1	1a
21	31	K[+648.3]SDKPLYGNDR	970.966113	2	962.505406	1	y8	46.07	7082	83372	1	2311_NC_dark9_01_targeted_b.RAW	dark	1	1b
21	31	K[+648.3]SDKPLYGNDR	970.966113	2	962.505406	1	y8	46.35	1521	20742	1	2311_NC_dark9_02_targeted.RAW	dark	2	2a
21	31	K[+648.3]SDKPLYGNDR	970.966113	2	962.505406	1	y8	46.48	2570	27688	1	2311_NC_dark9_02_targeted_b.RAW	dark	2	2b
21	31	K[+648.3]SDKPLYGNDR	970.966113	2	962.505406	1	y8	46.22	2177	26259	1	2311_NC_dark9_03_targeted.RAW	dark	3	3a
21	31	K[+648.3]SDKPLYGNDR	970.966113	2	962.505406	1	y8	46.25	6951	78687	1	2311_NC_dark9_03_targeted_b.RAW	dark	3	3b
21	31	K[+648.3]SDKPLYGNDR	970.966113	2	962.505406	1	y8	46.31	4891	60493	1	2311_NC_UV9_04_targeted.RAW	UV	4	4a
21	31	K[+648.3]SDKPLYGNDR	970.966113	2	962.505406	1	y8	46.29	3169	37972	1	2311_NC_UV9_04_targeted_b.RAW	UV	4	4b
21	31	K[+648.3]SDKPLYGNDR	970.966113	2	962.505406	1	y8	46.32	1927	23392	1	2311_NC_UV9_05_targeted.RAW	UV	5	5a
21	31	K[+648.3]SDKPLYGNDR	970.966113	2	962.505406	1	y8	46.52	2440	30156	1	2311_NC_UV9_05_targeted_b.RAW	UV	5	5b
21	31	K[+648.3]SDKPLYGNDR	970.966113	2	962.505406	1	y8	46.5	2394	20487	1	2311_NC_UV9_06_targeted.RAW	UV	6	6a
21	31	K[+648.3]SDKPLYGNDR	970.966113	2	962.505406	1	y8	46.29	1941	19010	1	2311_NC_UV9_06_targeted_b.RAW	UV	6	6b
21	31	KSDK[+648.3]PLYGNDR	970.966113	2	1725.797959	1	y9	46.44	77	589	2	2311_NC_dark9_01_targeted.RAW	dark	1	1a
21	31	KSDK[+648.3]PLYGNDR	970.966113	2	1725.797959	1	y9	46.12	141	1494	1	2311_NC_dark9_01_targeted_b.RAW	dark	1	1b
21	31	KSDK[+648.3]PLYGNDR	970.966113	2	1725.797959	1	y9	46.37	27	273	1	2311_NC_dark9_02_targeted.RAW	dark	2	2a
21	31	KSDK[+648.3]PLYGNDR	970.966113	2	1725.797959	1	y9	46.48	29	366	1	2311_NC_dark9_02_targeted_b.RAW	dark	2	2b
21	31	KSDK[+648.3]PLYGNDR	970.966113	2	1725.797959	1	y9	46.19	46	325	2	2311_NC_dark9_03_targeted.RAW	dark	3	3a

21	31	KSDK[+648.3]PLYGNDR	970.966113	2	1725.797959	1	y9	46.2	88	1012	1	2311_NC_dark9_03_targeted_b.RAW	dark	3	3b
21	31	KSDK[+648.3]PLYGNDR	970.966113	2	1725.797959	1	y9	46.35	75	764	1	2311_NC_UV9_04_targeted.RAW	UV	4	4a
21	31	KSDK[+648.3]PLYGNDR	970.966113	2	1725.797959	1	y9	46.24	66	538	1	2311_NC_UV9_04_targeted_b.RAW	UV	4	4b
21	31	KSDK[+648.3]PLYGNDR	970.966113	2	1725.797959	1	y9	46.32	29	269	1	2311_NC_UV9_05_targeted.RAW	UV	5	5a
21	31	KSDK[+648.3]PLYGNDR	970.966113	2	1725.797959	1	y9	46.49	45	377	1	2311_NC_UV9_05_targeted_b.RAW	UV	5	5b
21	31	KSDK[+648.3]PLYGNDR	970.966113	2	1725.797959	1	y9	46.5	46	329	1	2311_NC_UV9_06_targeted.RAW	UV	6	6a
21	31	KSDK[+648.3]PLYGNDR	970.966113	2	1725.797959	1	y9	46.28	27	211	1	2311_NC_UV9_06_targeted_b.RAW	UV	6	6b
21	31	KSDK[+648.3]PLYGNDR	970.966113	2	1610.771016	1	y8	46.32	8	19	3	2311_NC_dark9_01_targeted.RAW	dark	1	1a
21	31	KSDK[+648.3]PLYGNDR	970.966113	2	1610.771016	1	y8	46.05	13	46	3	2311_NC_dark9_01_targeted_b.RAW	dark	1	1b
21	31	KSDK[+648.3]PLYGNDR	970.966113	2	1610.771016	1	y8	46.37	17	58	3	2311_NC_dark9_02_targeted.RAW	dark	2	2a
21	31	KSDK[+648.3]PLYGNDR	970.966113	2	1610.771016	1	y8	46.45	11	73	3	2311_NC_dark9_02_targeted_b.RAW	dark	2	2b
21	31	KSDK[+648.3]PLYGNDR	970.966113	2	1610.771016	1	y8	46.26	5	22	3	2311_NC_dark9_03_targeted.RAW	dark	3	3a
21	31	KSDK[+648.3]PLYGNDR	970.966113	2	1610.771016	1	y8	46.23	7	32	3	2311_NC_dark9_03_targeted_b.RAW	dark	3	3b
21	31	KSDK[+648.3]PLYGNDR	970.966113	2	1610.771016	1	y8	46.26	6	19	3	2311_NC_UV9_04_targeted.RAW	UV	4	4a
21	31	KSDK[+648.3]PLYGNDR	970.966113	2	1610.771016	1	y8	46.19	6	15	3	2311_NC_UV9_04_targeted_b.RAW	UV	4	4b
21	31	KSDK[+648.3]PLYGNDR	970.966113	2	1610.771016	1	y8	46.46	6	53	3	2311_NC_UV9_05_targeted.RAW	UV	5	5a
21	31	KSDK[+648.3]PLYGNDR	970.966113	2	1610.771016	1	y8	46.61	5	37	3	2311_NC_UV9_05_targeted_b.RAW	UV	5	5b
21	31	KSDK[+648.3]PLYGNDR	970.966113	2	1610.771016	1	y8	46.5	21	130	3	2311_NC_UV9_06_targeted.RAW	UV	6	6a
21	31	KSDK[+648.3]PLYGNDR	970.966113	2	1610.771016	1	y8	46.24	14	86	3	2311_NC_UV9_06_targeted_b.RAW	UV	6	6b
21	31	KSDK[+648.3]PLYGNDR	970.966113	2	805.889146	2	y8	46.44	79	632	1	2311_NC_dark9_01_targeted.RAW	dark	1	1a
21	31	KSDK[+648.3]PLYGNDR	970.966113	2	805.889146	2	y8	46.07	104	1095	2	2311_NC_dark9_01_targeted_b.RAW	dark	1	1b
21	31	KSDK[+648.3]PLYGNDR	970.966113	2	805.889146	2	y8	46.37	28	269	2	2311_NC_dark9_02_targeted.RAW	dark	2	2a
21	31	KSDK[+648.3]PLYGNDR	970.966113	2	805.889146	2	y8	46.62	19	195	2	2311_NC_dark9_02_targeted_b.RAW	dark	2	2b
21	31	KSDK[+648.3]PLYGNDR	970.966113	2	805.889146	2	y8	46.24	47	474	1	2311_NC_dark9_03_targeted.RAW	dark	3	3a
21	31	KSDK[+648.3]PLYGNDR	970.966113	2	805.889146	2	y8	46.27	76	438	2	2311_NC_dark9_03_targeted_b.RAW	dark	3	3b
21	31	KSDK[+648.3]PLYGNDR	970.966113	2	805.889146	2	y8	46.35	59	522	2	2311_NC_UV9_04_targeted.RAW	UV	4	4a
21	31	KSDK[+648.3]PLYGNDR	970.966113	2	805.889146	2	y8	46.19	24	168	2	2311_NC_UV9_04_targeted_b.RAW	UV	4	4b
21	31	KSDK[+648.3]PLYGNDR	970.966113	2	805.889146	2	y8	46.29	28	234	2	2311_NC_UV9_05_targeted.RAW	UV	5	5a
21	31	KSDK[+648.3]PLYGNDR	970.966113	2	805.889146	2	y8	46.49	35	218	2	2311_NC_UV9_05_targeted_b.RAW	UV	5	5b
21	31	KSDK[+648.3]PLYGNDR	970.966113	2	805.889146	2	y8	46.62	27	248	2	2311_NC_UV9_06_targeted.RAW	UV	6	6a
21	31	KSDK[+648.3]PLYGNDR	970.966113	2	805.889146	2	y8	46.26	12	165	2	2311_NC_UV9_06_targeted_b.RAW	UV	6	6b
21	31	KSDK[+648.3]PLYGNDR	647.646501	3	863.402617	2	y9	46.46	286934	3812896	1	2311_NC_dark9_01_targeted.RAW	dark	1	1a
21	31	KSDK[+648.3]PLYGNDR	647.646501	3	863.402617	2	y9	46.12	363203	3859128	1	2311_NC_dark9_01_targeted_b.RAW	dark	1	1b
21	31	KSDK[+648.3]PLYGNDR	647.646501	3	863.402617	2	y9	46.39	164986	1657514	1	2311_NC_dark9_02_targeted.RAW	dark	2	2a
21	31	KSDK[+648.3]PLYGNDR	647.646501	3	863.402617	2	y9	46.48	116049	1394525	1	2311_NC_dark9_02_targeted_b.RAW	dark	2	2b
21	31	KSDK[+648.3]PLYGNDR	647.646501	3	863.402617	2	y9	46.24	142251	1866982	1	2311_NC_dark9_03_targeted.RAW	dark	3	3a
21	31	KSDK[+648.3]PLYGNDR	647.646501	3	863.402617	2	y9	46.23	127983	1931345	1	2311_NC_dark9_03_targeted_b.RAW	dark	3	3b
21	31	KSDK[+648.3]PLYGNDR	647.646501	3	863.402617	2	y9	46.38	162025	2740477	1	2311_NC_UV9_04_targeted.RAW	UV	4	4a
21	31	KSDK[+648.3]PLYGNDR	647.646501	3	863.402617	2	y9	46.24	158364	1812268	1	2311_NC_UV9_04_targeted_b.RAW	UV	4	4b
21	31	KSDK[+648.3]PLYGNDR	647.646501	3	863.402617	2	y9	46.32	124040	1611350	1	2311_NC_UV9_05_targeted.RAW	UV	5	5a
21	31	KSDK[+648.3]PLYGNDR	647.646501	3	863.402617	2	y9	46.49	129812	1429737	1	2311_NC_UV9_05_targeted_b.RAW	UV	5	5b
21	31	KSDK[+648.3]PLYGNDR	647.646501	3	863.402617	2	y9	46.5	153473	1494735	1	2311_NC_UV9_06_targeted.RAW	UV	6	6a
21	31	KSDK[+648.3]PLYGNDR	647.646501	3	863.402617	2	y9	46.33	64789	756114	1	2311_NC_UV9_06_targeted_b.RAW	UV	6	6b
21	41	KSDKPLYGNDRFEGYC[+57]LDLLK	844.424813	3	1101.056614	2	y18	49.83	93760	1260983	3	2311_NC_dark9_01_targeted_b.RAW	dark	1	1b
21	41	KSDKPLYGNDRFEGYC[+57]LDLLK	844.424813	3	1101.056614	2	y18	50.08	216815	3216534	2	2311_NC_dark9_02_targeted_b.RAW	dark	2	2b
21	41	KSDKPLYGNDRFEGYC[+57]LDLLK	844.424813	3	1101.056614	2	y18	49.73	356203	6792313	2	2311_NC_dark9_03_targeted_b.RAW	dark	3	3b
21	41	KSDKPLYGNDRFEGYC[+57]LDLLK	844.424813	3	1101.056614	2	y18	49.9	187844	2586033	2	2311_NC_UV9_04_targeted_b.RAW	UV	4	4b
21	41	KSDKPLYGNDRFEGYC[+57]LDLLK	844.424813	3	1101.056614	2	y18	50.19	194331	2263403	3	2311_NC_UV9_05_targeted_b.RAW	UV	5	5b
21	41	KSDKPLYGNDRFEGYC[+57]LDLLK	844.424813	3	1101.056614	2	y18	50.01	55619	653056	3	2311_NC_UV9_06_targeted_b.RAW	UV	6	6b
21	41	KSDKPLYGNDRFEGYC[+57]LDLLK	844.424813	3	1037.009132	2	y17	49.9	113725	2041118	1	2311_NC_dark9_01_targeted.RAW	dark	1	1a
21	41	KSDKPLYGNDRFEGYC[+57]LDLLK	844.424813	3	1037.009132	2	y17	49.95	162239	2773270	1	2311_NC_dark9_01_targeted_b.RAW	dark	1	1b
21	41	KSDKPLYGNDRFEGYC[+57]LDLLK	844.424813	3	1037.009132	2	y17	49.89	474424	8011850	1	2311_NC_dark9_02_targeted.RAW	dark	2	2a
21	41	KSDKPLYGNDRFEGYC[+57]LDLLK	844.424813	3	1037.009132	2	y17	50.08	520702	7686330	1	2311_NC_dark9_02_targeted_b.RAW	dark	2	2b
21	41	KSDKPLYGNDRFEGYC[+57]LDLLK	844.424813	3	1037.009132	2	y17	49.77	517106	10550088	1	2311_NC_dark9_03_targeted.RAW	dark	3	3a
21	41	KSDKPLYGNDRFEGYC[+57]LDLLK	844.424813	3	1037.009132	2	y17	49.73	830335	14496877	1	2311_NC_dark9_03_targeted_b.RAW	dark	3	3b
21	41	KSDKPLYGNDRFEGYC[+57]LDLLK	844.424813	3	1037.009132	2	y17	49.89	707578	11839024	1	2311_NC_UV9_04_targeted.RAW	UV	4	4a
21	41	KSDKPLYGNDRFEGYC[+57]LDLLK	844.424813	3	1037.009132	2	y17	49.9	472797	6670845	1	2311_NC_UV9_04_targeted_b.RAW	UV	4	4b
21	41	KSDKPLYGNDRFEGYC[+57]LDLLK	844.424813	3	1037.009132	2	y17	49.96	465999	7134055	1	2311_NC_UV9_05_targeted.RAW	UV	5	5a
21	41	KSDKPLYGNDRFEGYC[+57]LDLLK	844.424813	3	1037.009132	2	y17	50.19	458507	7321674	1	2311_NC_UV9_05_targeted_b.RAW	UV	5	5b
21	41	KSDKPLYGNDRFEGYC[+57]LDLLK	844.424813	3	1037.009132	2	y17	50.07	319261	5232489	1	2311_NC_UV9_06_targeted.RAW	UV	6	6a
21	41	KSDKPLYGNDRFEGYC[+57]LDLLK	844.424813	3	1037.009132	2	y17	50.01	115896	1412798	1	2311_NC_UV9_06_targeted_b.RAW	UV	6	6b
21	41	KSDKPLYGNDRFEGYC[+57]LDLLK	844.424813	3	1079.996753	2	b18	50.08	46439	752453	2	2311_NC_dark9_01_targeted.RAW	dark	1	1a
21	41	KSDKPLYGNDRFEGYC[+57]LDLLK	844.424813	3	1079.996753	2	b18	49.83	95008	1362271	2	2311_NC_dark9_01_targeted_b.RAW	dark	1	1b
21	41	KSDKPLYGNDRFEGYC[+57]LDLLK	844.424813	3	1079.996753	2	b18	49.89	201027	3016420	2	2311_NC_dark9_02_targeted.RAW	dark	2	2a

21	41	KSDKPLYGNDRFEGYC[+57]LDLLK	844.424813	3	1079.996753	2	b18	50.08	168978	2409145	3	2311_NC_dark9_02_targeted_b.RAW	dark	2	2b
21	41	KSDKPLYGNDRFEGYC[+57]LDLLK	844.424813	3	1079.996753	2	b18	49.72	176993	3401570	2	2311_NC_dark9_03_targeted.RAW	dark	3	3a
21	41	KSDKPLYGNDRFEGYC[+57]LDLLK	844.424813	3	1079.996753	2	b18	49.73	314020	6201203	3	2311_NC_dark9_03_targeted_b.RAW	dark	3	3b
21	41	KSDKPLYGNDRFEGYC[+57]LDLLK	844.424813	3	1079.996753	2	b18	49.89	220490	3743040	2	2311_NC_UV9_04_targeted.RAW	UV	4	4a
21	41	KSDKPLYGNDRFEGYC[+57]LDLLK	844.424813	3	1079.996753	2	b18	49.96	160318	2229910	3	2311_NC_UV9_04_targeted_b.RAW	UV	4	4b
21	41	KSDKPLYGNDRFEGYC[+57]LDLLK	844.424813	3	1079.996753	2	b18	50.02	154054	2643016	2	2311_NC_UV9_05_targeted.RAW	UV	5	5a
21	41	KSDKPLYGNDRFEGYC[+57]LDLLK	844.424813	3	1079.996753	2	b18	50.19	144745	2495375	2	2311_NC_UV9_05_targeted_b.RAW	UV	5	5b
21	41	KSDKPLYGNDRFEGYC[+57]LDLLK	844.424813	3	1079.996753	2	b18	50.07	113087	1937626	2	2311_NC_UV9_06_targeted.RAW	UV	6	6a
21	41	KSDKPLYGNDRFEGYC[+57]LDLLK	844.424813	3	1079.996753	2	b18	50.01	47321	680248	2	2311_NC_UV9_06_targeted_b.RAW	UV	6	6b
21	41	K[+648.3]SDKPLYGNDRFEGYC[+57]LDLLK	1060.513349	3	1158.570085	2	y19	56.97	209	2393	3	2311_NC_dark9_01_targeted.RAW	dark	1	1a
21	41	K[+648.3]SDKPLYGNDRFEGYC[+57]LDLLK	1060.513349	3	1158.570085	2	y19	57	282	2581	3	2311_NC_dark9_01_targeted_b.RAW	dark	1	1b
21	41	K[+648.3]SDKPLYGNDRFEGYC[+57]LDLLK	1060.513349	3	1158.570085	2	y19	56.79	408	6804	3	2311_NC_dark9_02_targeted.RAW	dark	2	2a
21	41	K[+648.3]SDKPLYGNDRFEGYC[+57]LDLLK	1060.513349	3	1158.570085	2	y19	56.94	320	5309	3	2311_NC_dark9_02_targeted_b.RAW	dark	2	2b
21	41	K[+648.3]SDKPLYGNDRFEGYC[+57]LDLLK	1060.513349	3	1158.570085	2	y19	56.87	976	15308	3	2311_NC_dark9_03_targeted.RAW	dark	3	3a
21	41	K[+648.3]SDKPLYGNDRFEGYC[+57]LDLLK	1060.513349	3	1158.570085	2	y19	56.92	903	18191	3	2311_NC_dark9_03_targeted_b.RAW	dark	3	3b
21	41	K[+648.3]SDKPLYGNDRFEGYC[+57]LDLLK	1060.513349	3	1158.570085	2	y19	56.78	977	15136	3	2311_NC_UV9_04_targeted.RAW	UV	4	4a
21	41	K[+648.3]SDKPLYGNDRFEGYC[+57]LDLLK	1060.513349	3	1158.570085	2	y19	57.19	702	12347	3	2311_NC_UV9_04_targeted_b.RAW	UV	4	4b
21	41	K[+648.3]SDKPLYGNDRFEGYC[+57]LDLLK	1060.513349	3	1158.570085	2	y19	56.83	763	11746	3	2311_NC_UV9_05_targeted.RAW	UV	5	5a
21	41	K[+648.3]SDKPLYGNDRFEGYC[+57]LDLLK	1060.513349	3	1158.570085	2	y19	57.07	776	10287	3	2311_NC_UV9_05_targeted_b.RAW	UV	5	5b
21	41	K[+648.3]SDKPLYGNDRFEGYC[+57]LDLLK	1060.513349	3	1158.570085	2	y19	56.96	737	8213	3	2311_NC_UV9_06_targeted.RAW	UV	6	6a
21	41	K[+648.3]SDKPLYGNDRFEGYC[+57]LDLLK	1060.513349	3	1158.570085	2	y19	56.99	710	9032	3	2311_NC_UV9_06_targeted_b.RAW	UV	6	6b
21	41	K[+648.3]SDKPLYGNDRFEGYC[+57]LDLLK	1060.513349	3	1101.056614	2	y18	56.85	903	9970	1	2311_NC_dark9_01_targeted.RAW	dark	1	1a
21	41	K[+648.3]SDKPLYGNDRFEGYC[+57]LDLLK	1060.513349	3	1101.056614	2	y18	57	1363	13018	1	2311_NC_dark9_01_targeted_b.RAW	dark	1	1b
21	41	K[+648.3]SDKPLYGNDRFEGYC[+57]LDLLK	1060.513349	3	1101.056614	2	y18	56.79	2134	30067	1	2311_NC_dark9_02_targeted.RAW	dark	2	2a
21	41	K[+648.3]SDKPLYGNDRFEGYC[+57]LDLLK	1060.513349	3	1101.056614	2	y18	56.88	1453	19959	1	2311_NC_dark9_02_targeted_b.RAW	dark	2	2b
21	41	K[+648.3]SDKPLYGNDRFEGYC[+57]LDLLK	1060.513349	3	1101.056614	2	y18	56.81	3847	68119	1	2311_NC_dark9_03_targeted.RAW	dark	3	3a
21	41	K[+648.3]SDKPLYGNDRFEGYC[+57]LDLLK	1060.513349	3	1101.056614	2	y18	56.92	4284	87227	1	2311_NC_dark9_03_targeted_b.RAW	dark	3	3b
21	41	K[+648.3]SDKPLYGNDRFEGYC[+57]LDLLK	1060.513349	3	1101.056614	2	y18	56.72	4830	68230	1	2311_NC_UV9_04_targeted.RAW	UV	4	4a
21	41	K[+648.3]SDKPLYGNDRFEGYC[+57]LDLLK	1060.513349	3	1101.056614	2	y18	57.13	3385	52067	1	2311_NC_UV9_04_targeted_b.RAW	UV	4	4b
21	41	K[+648.3]SDKPLYGNDRFEGYC[+57]LDLLK	1060.513349	3	1101.056614	2	y18	56.89	3232	47871	1	2311_NC_UV9_05_targeted.RAW	UV	5	5a
21	41	K[+648.3]SDKPLYGNDRFEGYC[+57]LDLLK	1060.513349	3	1101.056614	2	y18	57.01	3103	46072	1	2311_NC_UV9_05_targeted_b.RAW	UV	5	5b
21	41	K[+648.3]SDKPLYGNDRFEGYC[+57]LDLLK	1060.513349	3	1101.056614	2	y18	56.96	2941	37960	1	2311_NC_UV9_06_targeted.RAW	UV	6	6a
21	41	K[+648.3]SDKPLYGNDRFEGYC[+57]LDLLK	1060.513349	3	1101.056614	2	y18	56.99	2947	39299	1	2311_NC_UV9_06_targeted_b.RAW	UV	6	6b
21	41	K[+648.3]SDKPLYGNDRFEGYC[+57]LDLLK	1060.513349	3	1037.009132	2	y17	56.91	719	7982	2	2311_NC_dark9_01_targeted.RAW	dark	1	1a
21	41	K[+648.3]SDKPLYGNDRFEGYC[+57]LDLLK	1060.513349	3	1037.009132	2	y17	57	773	10735	2	2311_NC_dark9_01_targeted_b.RAW	dark	1	1b
21	41	K[+648.3]SDKPLYGNDRFEGYC[+57]LDLLK	1060.513349	3	1037.009132	2	y17	56.79	1765	24747	2	2311_NC_dark9_02_targeted.RAW	dark	2	2a
21	41	K[+648.3]SDKPLYGNDRFEGYC[+57]LDLLK	1060.513349	3	1037.009132	2	y17	56.88	1379	16842	2	2311_NC_dark9_02_targeted_b.RAW	dark	2	2b
21	41	K[+648.3]SDKPLYGNDRFEGYC[+57]LDLLK	1060.513349	3	1037.009132	2	y17	56.81	2996	55448	2	2311_NC_dark9_03_targeted.RAW	dark	3	3a
21	41	K[+648.3]SDKPLYGNDRFEGYC[+57]LDLLK	1060.513349	3	1037.009132	2	y17	56.98	3474	67472	2	2311_NC_dark9_03_targeted_b.RAW	dark	3	3b
21	41	K[+648.3]SDKPLYGNDRFEGYC[+57]LDLLK	1060.513349	3	1037.009132	2	y17	56.72	3936	56570	2	2311_NC_UV9_04_targeted.RAW	UV	4	4a
21	41	K[+648.3]SDKPLYGNDRFEGYC[+57]LDLLK	1060.513349	3	1037.009132	2	y17	57.19	2478	38960	2	2311_NC_UV9_04_targeted_b.RAW	UV	4	4b
21	41	K[+648.3]SDKPLYGNDRFEGYC[+57]LDLLK	1060.513349	3	1037.009132	2	y17	56.89	2750	41739	2	2311_NC_UV9_05_targeted.RAW	UV	5	5a
21	41	K[+648.3]SDKPLYGNDRFEGYC[+57]LDLLK	1060.513349	3	1037.009132	2	y17	57.01	2247	35405	2	2311_NC_UV9_05_targeted_b.RAW	UV	5	5b
21	41	K[+648.3]SDKPLYGNDRFEGYC[+57]LDLLK	1060.513349	3	1037.009132	2	y17	56.96	2518	30150	2	2311_NC_UV9_06_targeted.RAW	UV	6	6a
21	41	K[+648.3]SDKPLYGNDRFEGYC[+57]LDLLK	1060.513349	3	1037.009132	2	y17	56.99	2374	31767	2	2311_NC_UV9_06_targeted_b.RAW	UV	6	6b
21	41	KSDK[+648.3]PLYGNDRFEGYC[+57]LDLLK	1060.513349	3	1482.70289	2	y19	56.91	1928	18090	1	2311_NC_dark9_01_targeted.RAW	dark	1	1a
21	41	KSDK[+648.3]PLYGNDRFEGYC[+57]LDLLK	1060.513349	3	1482.70289	2	y19	57	2194	25134	1	2311_NC_dark9_01_targeted_b.RAW	dark	1	1b
21	41	KSDK[+648.3]PLYGNDRFEGYC[+57]LDLLK	1060.513349	3	1482.70289	2	y19	56.79	4136	62130	1	2311_NC_dark9_02_targeted.RAW	dark	2	2a
21	41	KSDK[+648.3]PLYGNDRFEGYC[+57]LDLLK	1060.513349	3	1482.70289	2	y19	56.88	2871	36477	1	2311_NC_dark9_02_targeted_b.RAW	dark	2	2b
21	41	KSDK[+648.3]PLYGNDRFEGYC[+57]LDLLK	1060.513349	3	1482.70289	2	y19	56.81	7940	125455	1	2311_NC_dark9_03_targeted.RAW	dark	3	3a
21	41	KSDK[+648.3]PLYGNDRFEGYC[+57]LDLLK	1060.513349	3	1482.70289	2	y19	56.92	7782	155320	1	2311_NC_dark9_03_targeted_b.RAW	dark	3	3b
21	41	KSDK[+648.3]PLYGNDRFEGYC[+57]LDLLK	1060.513349	3	1482.70289	2	y19	56.72	9870	147842	1	2311_NC_UV9_04_targeted.RAW	UV	4	4a
21	41	KSDK[+648.3]PLYGNDRFEGYC[+57]LDLLK	1060.513349	3	1482.70289	2	y19	57.13	6036	70663	1	2311_NC_UV9_04_targeted_b.RAW	UV	4	4b
21	41	KSDK[+648.3]PLYGNDRFEGYC[+57]LDLLK	1060.513349	3	1482.70289	2	y19	56.89	6372	89454	1	2311_NC_UV9_05_targeted.RAW	UV	5	5a
21	41	KSDK[+648.3]PLYGNDRFEGYC[+57]LDLLK	1060.513349	3	1482.70289	2	y19	57.01	5527	78991	1	2311_NC_UV9_05_targeted_b.RAW	UV	5	5b
21	41	KSDK[+648.3]PLYGNDRFEGYC[+57]LDLLK	1060.513349	3	1482.70289	2	y19	56.96	6531	76135	1	2311_NC_UV9_06_targeted.RAW	UV	6	6a
21	41	KSDK[+648.3]PLYGNDRFEGYC[+57]LDLLK	1060.513349	3	1482.70289	2	y19	56.99	5681	76603	1	2311_NC_UV9_06_targeted_b.RAW	UV	6	6b
21	41	KSDK[+648.3]PLYGNDRFEGYC[+57]LDLLK	1060.513349	3	1037.009132	2	y17	56.91	707	7671	2	2311_NC_dark9_01_targeted.RAW	dark	1	1a
21	41	KSDK[+648.3]PLYGNDRFEGYC[+57]LDLLK	1060.513349	3	1037.009132	2	y17	57	773	10735	2	2311_NC_dark9_01_targeted_b.RAW	dark	1	1b
21	41	KSDK[+648.3]PLYGNDRFEGYC[+57]LDLLK	1060.513349	3	1037.009132	2	y17	56.79	1770	25451	2	2311_NC_dark9_02_targeted.RAW	dark	2	2a
21	41	KSDK[+648.3]PLYGNDRFEGYC[+57]LDLLK	1060.513349	3	1037.009132	2	y17	56.88	1383	16544	2	2311_NC_dark9_02_targeted_b.RAW	dark	2	2b
21	41	KSDK[+648.3]PLYGNDRFEGYC[+57]LDLLK	1060.513349	3	1037.009132	2	y17	56.81	2986	51505	2	2311_NC_dark9_03_targeted.RAW	dark	3	3a
21	41	KSDK[+648.3]PLYGNDRFEGYC[+57]LDLLK	1060.513349	3	1037.009132	2	y17	56.98	3474	67356	2	2311_NC_dark9_03_targeted_b.RAW	dark	3	3b
21	41	KSDK[+648.3]PLYGNDRFEGYC[+57]LDLLK	1060.513349	3	1037.009132	2	y17	56.72	3919	56049	2	2311_NC_UV9_04_targeted.RAW	UV	4	4a

21	41	KSDK[+648.3]PLYGNDRFEGYC[+57]LDLLK	1060.513349	3	1037.009132	2	y17	57.19	2471	38742	2	2311_NC_UV9_04_targeted_b.RAW	UV	4	4b
21	41	KSDK[+648.3]PLYGNDRFEGYC[+57]LDLLK	1060.513349	3	1037.009132	2	y17	56.89	2759	39189	2	2311_NC_UV9_05_targeted.RAW	UV	5	5a
21	41	KSDK[+648.3]PLYGNDRFEGYC[+57]LDLLK	1060.513349	3	1037.009132	2	y17	57.01	2244	33515	2	2311_NC_UV9_05_targeted_b.RAW	UV	5	5b
21	41	KSDK[+648.3]PLYGNDRFEGYC[+57]LDLLK	1060.513349	3	1037.009132	2	y17	56.96	2517	30002	2	2311_NC_UV9_06_targeted.RAW	UV	6	6a
21	41	KSDK[+648.3]PLYGNDRFEGYC[+57]LDLLK	1060.513349	3	1037.009132	2	y17	56.99	2353	31444	2	2311_NC_UV9_06_targeted_b.RAW	UV	6	6b
42	54	ELSNILGFLYDVK	755.911224	2	954.529495	1	y8	67.71	1267720	19135922	2	2311_NC_dark9_01_targeted.RAW	dark	1	1a
42	54	ELSNILGFLYDVK	755.911224	2	954.529495	1	y8	67.89	1164898	18762762	2	2311_NC_dark9_01_targeted_b.RAW	dark	1	1b
42	54	ELSNILGFLYDVK	755.911224	2	954.529495	1	y8	67.72	1014443	16200940	2	2311_NC_dark9_02_targeted.RAW	dark	2	2a
42	54	ELSNILGFLYDVK	755.911224	2	954.529495	1	y8	67.89	710127	10937989	2	2311_NC_dark9_02_targeted_b.RAW	dark	2	2b
42	54	ELSNILGFLYDVK	755.911224	2	954.529495	1	y8	67.66	1560122	29733370	2	2311_NC_dark9_03_targeted.RAW	dark	3	3a
42	54	ELSNILGFLYDVK	755.911224	2	954.529495	1	y8	67.87	1303808	25694930	2	2311_NC_dark9_03_targeted_b.RAW	dark	3	3b
42	54	ELSNILGFLYDVK	755.911224	2	954.529495	1	y8	67.82	1437036	27130104	2	2311_NC_UV9_04_targeted.RAW	UV	4	4a
42	54	ELSNILGFLYDVK	755.911224	2	954.529495	1	y8	68.08	1152862	20105860	2	2311_NC_UV9_04_targeted_b.RAW	UV	4	4b
42	54	ELSNILGFLYDVK	755.911224	2	954.529495	1	y8	67.85	876589	13831175	2	2311_NC_UV9_05_targeted.RAW	UV	5	5a
42	54	ELSNILGFLYDVK	755.911224	2	954.529495	1	y8	68.07	726859	10459425	2	2311_NC_UV9_05_targeted_b.RAW	UV	5	5b
42	54	ELSNILGFLYDVK	755.911224	2	954.529495	1	y8	67.86	856227	13225796	2	2311_NC_UV9_06_targeted.RAW	UV	6	6a
42	54	ELSNILGFLYDVK	755.911224	2	954.529495	1	y8	68.06	807612	13267916	2	2311_NC_UV9_06_targeted_b.RAW	UV	6	6b
42	54	ELSNILGFLYDVK	755.911224	2	841.445431	1	y7	67.71	1419595	21944424	1	2311_NC_dark9_01_targeted.RAW	dark	1	1a
42	54	ELSNILGFLYDVK	755.911224	2	841.445431	1	y7	67.89	1291653	21301074	1	2311_NC_dark9_01_targeted_b.RAW	dark	1	1b
42	54	ELSNILGFLYDVK	755.911224	2	841.445431	1	y7	67.72	1160580	18519916	1	2311_NC_dark9_02_targeted.RAW	dark	2	2a
42	54	ELSNILGFLYDVK	755.911224	2	841.445431	1	y7	67.89	820793	12528636	1	2311_NC_dark9_02_targeted_b.RAW	dark	2	2b
42	54	ELSNILGFLYDVK	755.911224	2	841.445431	1	y7	67.66	1792190	33635396	1	2311_NC_dark9_03_targeted.RAW	dark	3	3a
42	54	ELSNILGFLYDVK	755.911224	2	841.445431	1	y7	67.87	1507011	29567060	1	2311_NC_dark9_03_targeted_b.RAW	dark	3	3b
42	54	ELSNILGFLYDVK	755.911224	2	841.445431	1	y7	67.82	1619321	31209252	1	2311_NC_UV9_04_targeted.RAW	UV	4	4a
42	54	ELSNILGFLYDVK	755.911224	2	841.445431	1	y7	68.08	1299531	23085610	1	2311_NC_UV9_04_targeted_b.RAW	UV	4	4b
42	54	ELSNILGFLYDVK	755.911224	2	841.445431	1	y7	67.85	991487	15522014	1	2311_NC_UV9_05_targeted.RAW	UV	5	5a
42	54	ELSNILGFLYDVK	755.911224	2	841.445431	1	y7	68.07	812942	12040699	1	2311_NC_UV9_05_targeted_b.RAW	UV	5	5b
42	54	ELSNILGFLYDVK	755.911224	2	841.445431	1	y7	67.9	951111	14951548	1	2311_NC_UV9_06_targeted.RAW	UV	6	6a
42	54	ELSNILGFLYDVK	755.911224	2	841.445431	1	y7	68.06	928300	15274148	1	2311_NC_UV9_06_targeted_b.RAW	UV	6	6b
42	54	ELSNILGFLYDVK	755.911224	2	637.355553	1	y5	67.71	456590	7168223	3	2311_NC_dark9_01_targeted.RAW	dark	1	1a
42	54	ELSNILGFLYDVK	755.911224	2	637.355553	1	y5	67.89	441180	7240600	3	2311_NC_dark9_01_targeted_b.RAW	dark	1	1b
42	54	ELSNILGFLYDVK	755.911224	2	637.355553	1	y5	67.75	375465	6224420	3	2311_NC_dark9_02_targeted.RAW	dark	2	2a
42	54	ELSNILGFLYDVK	755.911224	2	637.355553	1	y5	67.89	271943	4244714	3	2311_NC_dark9_02_targeted_b.RAW	dark	2	2b
42	54	ELSNILGFLYDVK	755.911224	2	637.355553	1	y5	67.66	612429	11441529	3	2311_NC_dark9_03_targeted.RAW	dark	3	3a
42	54	ELSNILGFLYDVK	755.911224	2	637.355553	1	y5	67.94	514324	9992123	3	2311_NC_dark9_03_targeted_b.RAW	dark	3	3b
42	54	ELSNILGFLYDVK	755.911224	2	637.355553	1	y5	67.82	538079	10576478	3	2311_NC_UV9_04_targeted.RAW	UV	4	4a
42	54	ELSNILGFLYDVK	755.911224	2	637.355553	1	y5	68.08	427651	7683970	3	2311_NC_UV9_04_targeted_b.RAW	UV	4	4b
42	54	ELSNILGFLYDVK	755.911224	2	637.355553	1	y5	67.85	342098	5284695	3	2311_NC_UV9_05_targeted.RAW	UV	5	5a
42	54	ELSNILGFLYDVK	755.911224	2	637.355553	1	y5	68.14	281329	4089334	3	2311_NC_UV9_05_targeted_b.RAW	UV	5	5b
42	54	ELSNILGFLYDVK	755.911224	2	637.355553	1	y5	67.9	342961	5107942	3	2311_NC_UV9_06_targeted.RAW	UV	6	6a
42	54	ELSNILGFLYDVK	755.911224	2	637.355553	1	y5	68.06	314011	5038554	3	2311_NC_UV9_06_targeted_b.RAW	UV	6	6b
42	54	ELSNILGFLYDVK	504.276575	3	841.445431	1	y7	68.18	1251	21565	2	2311_NC_dark9_01_targeted.RAW	dark	1	1a
42	54	ELSNILGFLYDVK	504.276575	3	841.445431	1	y7	68.6	928	22346	2	2311_NC_dark9_01_targeted_b.RAW	dark	1	1b
42	54	ELSNILGFLYDVK	504.276575	3	841.445431	1	y7	68.18	696	11317	2	2311_NC_dark9_02_targeted.RAW	dark	2	2a
42	54	ELSNILGFLYDVK	504.276575	3	841.445431	1	y7	68.35	763	14893	2	2311_NC_dark9_02_targeted_b.RAW	dark	2	2b
42	54	ELSNILGFLYDVK	504.276575	3	841.445431	1	y7	68.47	705	19213	2	2311_NC_dark9_03_targeted.RAW	dark	3	3a
42	54	ELSNILGFLYDVK	504.276575	3	841.445431	1	y7	68.69	550	10277	2	2311_NC_dark9_03_targeted_b.RAW	dark	3	3b
42	54	ELSNILGFLYDVK	504.276575	3	841.445431	1	y7	68.5	702	17160	2	2311_NC_UV9_04_targeted.RAW	UV	4	4a
42	54	ELSNILGFLYDVK	504.276575	3	841.445431	1	y7	68.55	737	14614	2	2311_NC_UV9_04_targeted_b.RAW	UV	4	4b
42	54	ELSNILGFLYDVK	504.276575	3	841.445431	1	y7	68.39	502	12518	2	2311_NC_UV9_05_targeted.RAW	UV	5	5a
42	54	ELSNILGFLYDVK	504.276575	3	841.445431	1	y7	68.43	482	12456	1	2311_NC_UV9_05_targeted_b.RAW	UV	5	5b
42	54	ELSNILGFLYDVK	504.276575	3	841.445431	1	y7	68.5	862	20393	2	2311_NC_UV9_06_targeted.RAW	UV	6	6a
42	54	ELSNILGFLYDVK	504.276575	3	841.445431	1	y7	68.81	560	16963	2	2311_NC_UV9_06_targeted_b.RAW	UV	6	6b
42	54	ELSNILGFLYDVK	504.276575	3	637.355553	1	y5	68.21	732	12916	3	2311_NC_dark9_01_targeted.RAW	dark	1	1a
42	54	ELSNILGFLYDVK	504.276575	3	637.355553	1	y5	68.6	491	14082	3	2311_NC_dark9_01_targeted_b.RAW	dark	1	1b
42	54	ELSNILGFLYDVK	504.276575	3	637.355553	1	y5	68.18	427	5762	3	2311_NC_dark9_02_targeted.RAW	dark	2	2a
42	54	ELSNILGFLYDVK	504.276575	3	637.355553	1	y5	68.35	485	9138	3	2311_NC_dark9_02_targeted_b.RAW	dark	2	2b
42	54	ELSNILGFLYDVK	504.276575	3	637.355553	1	y5	68.37	457	12059	3	2311_NC_dark9_03_targeted.RAW	dark	3	3a
42	54	ELSNILGFLYDVK	504.276575	3	637.355553	1	y5	68.72	344	5902	3	2311_NC_dark9_03_targeted_b.RAW	dark	3	3b
42	54	ELSNILGFLYDVK	504.276575	3	637.355553	1	y5	68.5	409	10637	3	2311_NC_UV9_04_targeted.RAW	UV	4	4a
42	54	ELSNILGFLYDVK	504.276575	3	637.355553	1	y5	68.55	428	8893	3	2311_NC_UV9_04_targeted_b.RAW	UV	4	4b
42	54	ELSNILGFLYDVK	504.276575	3	637.355553	1	y5	68.35	368	7173	3	2311_NC_UV9_05_targeted.RAW	UV	5	5a
42	54	ELSNILGFLYDVK	504.276575	3	637.355553	1	y5	68.46	283	7157	3	2311_NC_UV9_05_targeted_b.RAW	UV	5	5b
42	54	ELSNILGFLYDVK	504.276575	3	637.355553	1	y5	68.43	513	12798	3	2311_NC_UV9_06_targeted.RAW	UV	6	6a

42	54	ELSNILGFLYDVK	504.276575	3	637.355553	1	y5	68.67	401	11297	3	2311_NC_UV9_06_targeted_b.RAW	UV	6	6b
42	54	ELSNILGFLYDVK	504.276575	3	524.271489	1	y4	68.21	1251	21585	1	2311_NC_dark9_01_targeted.RAW	dark	1	1a
42	54	ELSNILGFLYDVK	504.276575	3	524.271489	1	y4	68.6	841	22536	1	2311_NC_dark9_01_targeted_b.RAW	dark	1	1b
42	54	ELSNILGFLYDVK	504.276575	3	524.271489	1	y4	68.18	813	12602	1	2311_NC_dark9_02_targeted.RAW	dark	2	2a
42	54	ELSNILGFLYDVK	504.276575	3	524.271489	1	y4	68.35	931	15799	1	2311_NC_dark9_02_targeted_b.RAW	dark	2	2b
42	54	ELSNILGFLYDVK	504.276575	3	524.271489	1	y4	68.33	821	20855	1	2311_NC_dark9_03_targeted.RAW	dark	3	3a
42	54	ELSNILGFLYDVK	504.276575	3	524.271489	1	y4	68.72	627	10370	1	2311_NC_dark9_03_targeted_b.RAW	dark	3	3b
42	54	ELSNILGFLYDVK	504.276575	3	524.271489	1	y4	68.57	701	18631	1	2311_NC_UV9_04_targeted.RAW	UV	4	4a
42	54	ELSNILGFLYDVK	504.276575	3	524.271489	1	y4	68.55	714	15742	1	2311_NC_UV9_04_targeted_b.RAW	UV	4	4b
42	54	ELSNILGFLYDVK	504.276575	3	524.271489	1	y4	68.39	555	14489	1	2311_NC_UV9_05_targeted.RAW	UV	5	5a
42	54	ELSNILGFLYDVK	504.276575	3	524.271489	1	y4	68.68	435	12437	2	2311_NC_UV9_05_targeted_b.RAW	UV	5	5b
42	54	ELSNILGFLYDVK	504.276575	3	524.271489	1	y4	68.43	906	23000	1	2311_NC_UV9_06_targeted.RAW	UV	6	6a
42	54	ELSNILGFLYDVK	504.276575	3	524.271489	1	y4	68.67	573	17578	1	2311_NC_UV9_06_targeted_b.RAW	UV	6	6b
55	67	LVPDGKYGAQNDK	702.859523	2	1192.559292	1	y11	27.28	69	1361	3	2311_NC_dark9_01_targeted.RAW	dark	1	1a
55	67	LVPDGKYGAQNDK	702.859523	2	1192.559292	1	y11	27.21	113	1712	3	2311_NC_dark9_01_targeted_b.RAW	dark	1	1b
55	67	LVPDGKYGAQNDK	702.859523	2	1192.559292	1	y11	27.1	163	2699	3	2311_NC_dark9_02_targeted.RAW	dark	2	2a
55	67	LVPDGKYGAQNDK	702.859523	2	1192.559292	1	y11	27.33	175	3337	3	2311_NC_dark9_02_targeted_b.RAW	dark	2	2b
55	67	LVPDGKYGAQNDK	702.859523	2	1192.559292	1	y11	27.1	292	4886	3	2311_NC_dark9_03_targeted.RAW	dark	3	3a
55	67	LVPDGKYGAQNDK	702.859523	2	1192.559292	1	y11	27.33	287	6112	3	2311_NC_dark9_03_targeted_b.RAW	dark	3	3b
55	67	LVPDGKYGAQNDK	702.859523	2	1192.559292	1	y11	27.28	338	5901	3	2311_NC_UV9_04_targeted.RAW	UV	4	4a
55	67	LVPDGKYGAQNDK	702.859523	2	1192.559292	1	y11	27.5	155	2467	3	2311_NC_UV9_04_targeted_b.RAW	UV	4	4b
55	67	LVPDGKYGAQNDK	702.859523	2	1192.559292	1	y11	27.28	11	161	3	2311_NC_UV9_05_targeted.RAW	UV	5	5a
55	67	LVPDGKYGAQNDK	702.859523	2	1192.559292	1	y11	27.51	393	7967	3	2311_NC_UV9_05_targeted_b.RAW	UV	5	5b
55	67	LVPDGKYGAQNDK	702.859523	2	1192.559292	1	y11	27.27	179	2618	3	2311_NC_UV9_06_targeted.RAW	UV	6	6a
55	67	LVPDGKYGAQNDK	702.859523	2	1192.559292	1	y11	27.39	2	16	3	2311_NC_UV9_06_targeted_b.RAW	UV	6	6b
55	67	LVPDGKYGAQNDK	702.859523	2	596.783284	2	y11	27.22	1077	25774	1	2311_NC_dark9_01_targeted.RAW	dark	1	1a
55	67	LVPDGKYGAQNDK	702.859523	2	596.783284	2	y11	27.21	1746	33485	1	2311_NC_dark9_01_targeted_b.RAW	dark	1	1b
55	67	LVPDGKYGAQNDK	702.859523	2	596.783284	2	y11	27.16	2766	54198	1	2311_NC_dark9_02_targeted.RAW	dark	2	2a
55	67	LVPDGKYGAQNDK	702.859523	2	596.783284	2	y11	27.33	3076	63892	1	2311_NC_dark9_02_targeted_b.RAW	dark	2	2b
55	67	LVPDGKYGAQNDK	702.859523	2	596.783284	2	y11	27.1	4419	96580	1	2311_NC_dark9_03_targeted.RAW	dark	3	3a
55	67	LVPDGKYGAQNDK	702.859523	2	596.783284	2	y11	27.33	6098	132575	1	2311_NC_dark9_03_targeted_b.RAW	dark	3	3b
55	67	LVPDGKYGAQNDK	702.859523	2	596.783284	2	y11	27.28	5130	112683	1	2311_NC_UV9_04_targeted.RAW	UV	4	4a
55	67	LVPDGKYGAQNDK	702.859523	2	596.783284	2	y11	27.5	2793	52536	1	2311_NC_UV9_04_targeted_b.RAW	UV	4	4b
55	67	LVPDGKYGAQNDK	702.859523	2	596.783284	2	y11	27.28	126	3338	1	2311_NC_UV9_05_targeted.RAW	UV	5	5a
55	67	LVPDGKYGAQNDK	702.859523	2	596.783284	2	y11	27.51	7491	145958	1	2311_NC_UV9_05_targeted_b.RAW	UV	5	5b
55	67	LVPDGKYGAQNDK	702.859523	2	596.783284	2	y11	27.27	2979	50574	1	2311_NC_UV9_06_targeted.RAW	UV	6	6a
55	67	LVPDGKYGAQNDK	702.859523	2	596.783284	2	y11	27.56	61	1362	1	2311_NC_UV9_06_targeted_b.RAW	UV	6	6b
55	67	LVPDGKYGAQNDK	702.859523	2	490.74343	2	y9	27.22	135	1897	2	2311_NC_dark9_01_targeted.RAW	dark	1	1a
55	67	LVPDGKYGAQNDK	702.859523	2	490.74343	2	y9	27.21	142	2083	2	2311_NC_dark9_01_targeted_b.RAW	dark	1	1b
55	67	LVPDGKYGAQNDK	702.859523	2	490.74343	2	y9	27.16	210	3802	2	2311_NC_dark9_02_targeted.RAW	dark	2	2a
55	67	LVPDGKYGAQNDK	702.859523	2	490.74343	2	y9	27.33	235	4099	2	2311_NC_dark9_02_targeted_b.RAW	dark	2	2b
55	67	LVPDGKYGAQNDK	702.859523	2	490.74343	2	y9	27.1	282	6054	2	2311_NC_dark9_03_targeted.RAW	dark	3	3a
55	67	LVPDGKYGAQNDK	702.859523	2	490.74343	2	y9	27.33	390	8721	2	2311_NC_dark9_03_targeted_b.RAW	dark	3	3b
55	67	LVPDGKYGAQNDK	702.859523	2	490.74343	2	y9	27.28	344	6460	2	2311_NC_UV9_04_targeted.RAW	UV	4	4a
55	67	LVPDGKYGAQNDK	702.859523	2	490.74343	2	y9	27.5	172	3321	2	2311_NC_UV9_04_targeted_b.RAW	UV	4	4b
55	67	LVPDGKYGAQNDK	702.859523	2	490.74343	2	y9	27.51	17	409	2	2311_NC_UV9_05_targeted.RAW	UV	5	5a
55	67	LVPDGKYGAQNDK	702.859523	2	490.74343	2	y9	27.45	575	10033	2	2311_NC_UV9_05_targeted_b.RAW	UV	5	5b
55	67	LVPDGKYGAQNDK	702.859523	2	490.74343	2	y9	27.27	240	3598	2	2311_NC_UV9_06_targeted.RAW	UV	6	6a
55	67	LVPDGKYGAQNDK	702.859523	2	490.74343	2	y9	27.68	10	207	2	2311_NC_UV9_06_targeted_b.RAW	UV	6	6b
55	67	LVPDGKYGAQNDK	468.908774	3	596.783284	2	y11	27.22	751	17095	1	2311_NC_dark9_01_targeted.RAW	dark	1	1a
55	67	LVPDGKYGAQNDK	468.908774	3	596.783284	2	y11	27.15	772	14732	1	2311_NC_dark9_01_targeted_b.RAW	dark	1	1b
55	67	LVPDGKYGAQNDK	468.908774	3	596.783284	2	y11	27.16	2112	37847	1	2311_NC_dark9_02_targeted.RAW	dark	2	2a
55	67	LVPDGKYGAQNDK	468.908774	3	596.783284	2	y11	27.39	1380	31808	1	2311_NC_dark9_02_targeted_b.RAW	dark	2	2b
55	67	LVPDGKYGAQNDK	468.908774	3	596.783284	2	y11	27.1	3168	68849	1	2311_NC_dark9_03_targeted.RAW	dark	3	3a
55	67	LVPDGKYGAQNDK	468.908774	3	596.783284	2	y11	27.33	2788	57280	1	2311_NC_dark9_03_targeted_b.RAW	dark	3	3b
55	67	LVPDGKYGAQNDK	468.908774	3	596.783284	2	y11	27.28	3762	73194	1	2311_NC_UV9_04_targeted.RAW	UV	4	4a
55	67	LVPDGKYGAQNDK	468.908774	3	596.783284	2	y11	27.8	411	12567	1	2311_NC_UV9_04_targeted_b.RAW	UV	4	4b
55	67	LVPDGKYGAQNDK	468.908774	3	596.783284	2	y11	27.28	44	1174	1	2311_NC_UV9_05_targeted.RAW	UV	5	5a
55	67	LVPDGKYGAQNDK	468.908774	3	596.783284	2	y11	27.45	4273	77742	1	2311_NC_UV9_05_targeted_b.RAW	UV	5	5b
55	67	LVPDGKYGAQNDK	468.908774	3	596.783284	2	y11	27.27	2341	42936	1	2311_NC_UV9_06_targeted.RAW	UV	6	6a
55	67	LVPDGKYGAQNDK	468.908774	3	596.783284	2	y11	27.62	15	315	2	2311_NC_UV9_06_targeted_b.RAW	UV	6	6b
55	67	LVPDGKYGAQNDK	468.908774	3	398.185217	2	y7	27.28	344	9225	2	2311_NC_dark9_01_targeted.RAW	dark	1	1a
55	67	LVPDGKYGAQNDK	468.908774	3	398.185217	2	y7	27.21	356	8484	2	2311_NC_dark9_01_targeted_b.RAW	dark	1	1b
55	67	LVPDGKYGAQNDK	468.908774	3	398.185217	2	y7	27.16	1030	19712	2	2311_NC_dark9_02_targeted.RAW	dark	2	2a

55	67	LVPDGKYGAQNDK	468.908774	3	398.185217	2	y7	27.39	762	17141	2	2311_NC_dark9_02_targeted_b.RAW	dark	2	2b
55	67	LVPDGKYGAQNDK	468.908774	3	398.185217	2	y7	27.1	1617	37263	2	2311_NC_dark9_03_targeted.RAW	dark	3	3a
55	67	LVPDGKYGAQNDK	468.908774	3	398.185217	2	y7	27.33	1367	30493	2	2311_NC_dark9_03_targeted_b.RAW	dark	3	3b
55	67	LVPDGKYGAQNDK	468.908774	3	398.185217	2	y7	27.28	1828	39173	2	2311_NC_UV9_04_targeted.RAW	UV	4	4a
55	67	LVPDGKYGAQNDK	468.908774	3	398.185217	2	y7	27.8	232	6777	2	2311_NC_UV9_04_targeted_b.RAW	UV	4	4b
55	67	LVPDGKYGAQNDK	468.908774	3	398.185217	2	y7	27.46	24	638	2	2311_NC_UV9_05_targeted.RAW	UV	5	5a
55	67	LVPDGKYGAQNDK	468.908774	3	398.185217	2	y7	27.45	1998	40309	2	2311_NC_UV9_05_targeted_b.RAW	UV	5	5b
55	67	LVPDGKYGAQNDK	468.908774	3	398.185217	2	y7	27.27	1290	23654	2	2311_NC_UV9_06_targeted.RAW	UV	6	6a
55	67	LVPDGKYGAQNDK	468.908774	3	398.185217	2	y7	27.68	20	337	1	2311_NC_UV9_06_targeted_b.RAW	UV	6	6b
55	67	LVPDGKYGAQNDK	468.908774	3	213.159754	1	b2	27.28	39	928	3	2311_NC_dark9_01_targeted.RAW	dark	1	1a
55	67	LVPDGKYGAQNDK	468.908774	3	213.159754	1	b2	27.21	44	768	3	2311_NC_dark9_01_targeted_b.RAW	dark	1	1b
55	67	LVPDGKYGAQNDK	468.908774	3	213.159754	1	b2	27.16	128	2214	3	2311_NC_dark9_02_targeted.RAW	dark	2	2a
55	67	LVPDGKYGAQNDK	468.908774	3	213.159754	1	b2	27.33	86	1874	3	2311_NC_dark9_02_targeted_b.RAW	dark	2	2b
55	67	LVPDGKYGAQNDK	468.908774	3	213.159754	1	b2	27.1	208	4243	3	2311_NC_dark9_03_targeted.RAW	dark	3	3a
55	67	LVPDGKYGAQNDK	468.908774	3	213.159754	1	b2	27.33	194	3819	3	2311_NC_dark9_03_targeted_b.RAW	dark	3	3b
55	67	LVPDGKYGAQNDK	468.908774	3	213.159754	1	b2	27.28	182	3763	3	2311_NC_UV9_04_targeted.RAW	UV	4	4a
55	67	LVPDGKYGAQNDK	468.908774	3	213.159754	1	b2	27.8	27	813	3	2311_NC_UV9_04_targeted_b.RAW	UV	4	4b
55	67	LVPDGKYGAQNDK	468.908774	3	213.159754	1	b2	27.63	6	163	3	2311_NC_UV9_05_targeted.RAW	UV	5	5a
55	67	LVPDGKYGAQNDK	468.908774	3	213.159754	1	b2	27.45	218	4376	3	2311_NC_UV9_05_targeted_b.RAW	UV	5	5b
55	67	LVPDGKYGAQNDK	468.908774	3	213.159754	1	b2	27.27	147	2544	3	2311_NC_UV9_06_targeted.RAW	UV	6	6a
55	67	LVPDGKYGAQNDK	468.908774	3	213.159754	1	b2	27.45	3	44	3	2311_NC_UV9_06_targeted_b.RAW	UV	6	6b
55	75	LVPDGKYGAQNDKGEWNGMVK	769.379315	3	1048.524426	1	y9	39.32	8977	55588	2	2311_NC_dark9_01_targeted.RAW	dark	1	1a
55	75	LVPDGKYGAQNDKGEWNGMVK	769.379315	3	1048.524426	1	y9	39.19	4598	64416	2	2311_NC_dark9_01_targeted_b.RAW	dark	1	1b
55	75	LVPDGKYGAQNDKGEWNGMVK	769.379315	3	1048.524426	1	y9	39.34	11626	124166	2	2311_NC_dark9_02_targeted.RAW	dark	2	2a
55	75	LVPDGKYGAQNDKGEWNGMVK	769.379315	3	1048.524426	1	y9	39.24	10876	93522	2	2311_NC_dark9_02_targeted_b.RAW	dark	2	2b
55	75	LVPDGKYGAQNDKGEWNGMVK	769.379315	3	1048.524426	1	y9	39.26	46975	358170	2	2311_NC_dark9_03_targeted.RAW	dark	3	3a
55	75	LVPDGKYGAQNDKGEWNGMVK	769.379315	3	1048.524426	1	y9	39.11	84604	623335	2	2311_NC_dark9_03_targeted_b.RAW	dark	3	3b
55	75	LVPDGKYGAQNDKGEWNGMVK	769.379315	3	1048.524426	1	y9	39.24	33526	261702	2	2311_NC_UV9_04_targeted.RAW	UV	4	4a
55	75	LVPDGKYGAQNDKGEWNGMVK	769.379315	3	1048.524426	1	y9	39.28	52346	439911	2	2311_NC_UV9_04_targeted_b.RAW	UV	4	4b
55	75	LVPDGKYGAQNDKGEWNGMVK	769.379315	3	1048.524426	1	y9	39.2	24374	252468	2	2311_NC_UV9_05_targeted.RAW	UV	5	5a
55	75	LVPDGKYGAQNDKGEWNGMVK	769.379315	3	1048.524426	1	y9	39.47	63234	800897	2	2311_NC_UV9_05_targeted_b.RAW	UV	5	5b
55	75	LVPDGKYGAQNDKGEWNGMVK	769.379315	3	1048.524426	1	y9	39.28	33691	363194	2	2311_NC_UV9_06_targeted.RAW	UV	6	6a
55	75	LVPDGKYGAQNDKGEWNGMVK	769.379315	3	1048.524426	1	y9	39.35	31104	196843	2	2311_NC_UV9_06_targeted_b.RAW	UV	6	6b
55	75	LVPDGKYGAQNDKGEWNGMVK	769.379315	3	1047.489095	2	y19	39.34	12857	164282	1	2311_NC_dark9_01_targeted.RAW	dark	1	1a
55	75	LVPDGKYGAQNDKGEWNGMVK	769.379315	3	1047.489095	2	y19	39.12	13193	148817	1	2311_NC_dark9_01_targeted_b.RAW	dark	1	1b
55	75	LVPDGKYGAQNDKGEWNGMVK	769.379315	3	1047.489095	2	y19	39.25	30680	476049	1	2311_NC_dark9_02_targeted.RAW	dark	2	2a
55	75	LVPDGKYGAQNDKGEWNGMVK	769.379315	3	1047.489095	2	y19	39.31	25255	378982	1	2311_NC_dark9_02_targeted_b.RAW	dark	2	2b
55	75	LVPDGKYGAQNDKGEWNGMVK	769.379315	3	1047.489095	2	y19	39.17	76471	1281013	1	2311_NC_dark9_03_targeted.RAW	dark	3	3a
55	75	LVPDGKYGAQNDKGEWNGMVK	769.379315	3	1047.489095	2	y19	39.11	89189	1368153	1	2311_NC_dark9_03_targeted_b.RAW	dark	3	3b
55	75	LVPDGKYGAQNDKGEWNGMVK	769.379315	3	1047.489095	2	y19	39.17	61991	931660	1	2311_NC_UV9_04_targeted.RAW	UV	4	4a
55	75	LVPDGKYGAQNDKGEWNGMVK	769.379315	3	1047.489095	2	y19	39.28	46083	519658	1	2311_NC_UV9_04_targeted_b.RAW	UV	4	4b
55	75	LVPDGKYGAQNDKGEWNGMVK	769.379315	3	1047.489095	2	y19	39.2	41912	750205	1	2311_NC_UV9_05_targeted.RAW	UV	5	5a
55	75	LVPDGKYGAQNDKGEWNGMVK	769.379315	3	1047.489095	2	y19	39.4	83252	1089608	1	2311_NC_UV9_05_targeted_b.RAW	UV	5	5b
55	75	LVPDGKYGAQNDKGEWNGMVK	769.379315	3	1047.489095	2	y19	39.26	32771	431339	1	2311_NC_UV9_06_targeted.RAW	UV	6	6a
55	75	LVPDGKYGAQNDKGEWNGMVK	769.379315	3	1047.489095	2	y19	39.33	32273	436200	1	2311_NC_UV9_06_targeted_b.RAW	UV	6	6b
55	75	LVPDGKYGAQNDKGEWNGMVK	769.379315	3	848.891028	2	y15	39.34	1037	11927	3	2311_NC_dark9_01_targeted.RAW	dark	1	1a
55	75	LVPDGKYGAQNDKGEWNGMVK	769.379315	3	848.891028	2	y15	39.09	783	10910	3	2311_NC_dark9_01_targeted_b.RAW	dark	1	1b
55	75	LVPDGKYGAQNDKGEWNGMVK	769.379315	3	848.891028	2	y15	39.25	2271	30809	3	2311_NC_dark9_02_targeted.RAW	dark	2	2a
55	75	LVPDGKYGAQNDKGEWNGMVK	769.379315	3	848.891028	2	y15	39.33	1999	27063	3	2311_NC_dark9_02_targeted_b.RAW	dark	2	2b
55	75	LVPDGKYGAQNDKGEWNGMVK	769.379315	3	848.891028	2	y15	39.12	6198	90987	3	2311_NC_dark9_03_targeted.RAW	dark	3	3a
55	75	LVPDGKYGAQNDKGEWNGMVK	769.379315	3	848.891028	2	y15	39.13	5219	85395	3	2311_NC_dark9_03_targeted_b.RAW	dark	3	3b
55	75	LVPDGKYGAQNDKGEWNGMVK	769.379315	3	848.891028	2	y15	39.19	4508	59189	3	2311_NC_UV9_04_targeted.RAW	UV	4	4a
55	75	LVPDGKYGAQNDKGEWNGMVK	769.379315	3	848.891028	2	y15	39.28	3436	39374	3	2311_NC_UV9_04_targeted_b.RAW	UV	4	4b
55	75	LVPDGKYGAQNDKGEWNGMVK	769.379315	3	848.891028	2	y15	39.15	2845	47831	3	2311_NC_UV9_05_targeted.RAW	UV	5	5a
55	75	LVPDGKYGAQNDKGEWNGMVK	769.379315	3	848.891028	2	y15	39.37	4923	73317	3	2311_NC_UV9_05_targeted_b.RAW	UV	5	5b
55	75	LVPDGKYGAQNDKGEWNGMVK	769.379315	3	848.891028	2	y15	39.4	1859	29240	3	2311_NC_UV9_06_targeted.RAW	UV	6	6a
55	75	LVPDGKYGAQNDKGEWNGMVK	769.379315	3	848.891028	2	y15	39.33	2145	30185	3	2311_NC_UV9_06_targeted_b.RAW	UV	6	6b
55	75	LVPDGK[+648.3]YGAQNDKGEWNGMVK	985.467851	3	1371.6219	2	y19	52.3	588	11430	1	2311_NC_dark9_01_targeted.RAW	dark	1	1a
55	75	LVPDGK[+648.3]YGAQNDKGEWNGMVK	985.467851	3	1371.6219	2	y19	52.52	1010	15327	1	2311_NC_dark9_01_targeted_b.RAW	dark	1	1b
55	75	LVPDGK[+648.3]YGAQNDKGEWNGMVK	985.467851	3	1371.6219	2	y19	52.46	641	11953	1	2311_NC_dark9_02_targeted.RAW	dark	2	2a
55	75	LVPDGK[+648.3]YGAQNDKGEWNGMVK	985.467851	3	1371.6219	2	y19	52.53	688	12510	1	2311_NC_dark9_02_targeted_b.RAW	dark	2	2b
55	75	LVPDGK[+648.3]YGAQNDKGEWNGMVK	985.467851	3	1371.6219	2	y19	52.29	1187	20320	1	2311_NC_dark9_03_targeted.RAW	dark	3	3a
55	75	LVPDGK[+648.3]YGAQNDKGEWNGMVK	985.467851	3	1371.6219	2	y19	52.6	1374	23624	1	2311_NC_dark9_03_targeted_b.RAW	dark	3	3b
55	75	LVPDGK[+648.3]YGAQNDKGEWNGMVK	985.467851	3	1371.6219	2	y19	52.52	556	9219	1	2311_NC_UV9_04_targeted.RAW	UV	4	4a

55	75	LVPDGK[+648.3]YGAQNDKGEWNGMVK	985.467851	3	1371.6219	2	y19	52.65	394	6659	1	2311_NC_UV9_04_targeted_b.RAW	UV	4	4b
55	75	LVPDGK[+648.3]YGAQNDKGEWNGMVK	985.467851	3	1371.6219	2	y19	52.54	410	6572	1	2311_NC_UV9_05_targeted.RAW	UV	5	5a
55	75	LVPDGK[+648.3]YGAQNDKGEWNGMVK	985.467851	3	1371.6219	2	y19	52.82	349	5393	1	2311_NC_UV9_05_targeted_b.RAW	UV	5	5b
55	75	LVPDGK[+648.3]YGAQNDKGEWNGMVK	985.467851	3	1371.6219	2	y19	52.71	158	3121	1	2311_NC_UV9_06_targeted.RAW	UV	6	6a
55	75	LVPDGK[+648.3]YGAQNDKGEWNGMVK	985.467851	3	1371.6219	2	y19	52.65	259	4209	1	2311_NC_UV9_06_targeted_b.RAW	UV	6	6b
55	75	LVPDGK[+648.3]YGAQNDKGEWNGMVK	985.467851	3	848.891028	2	y15	52.3	250	4120	2	2311_NC_dark9_01_targeted.RAW	dark	1	1a
55	75	LVPDGK[+648.3]YGAQNDKGEWNGMVK	985.467851	3	848.891028	2	y15	52.52	409	6679	3	2311_NC_dark9_01_targeted_b.RAW	dark	1	1b
55	75	LVPDGK[+648.3]YGAQNDKGEWNGMVK	985.467851	3	848.891028	2	y15	52.46	320	5798	3	2311_NC_dark9_02_targeted.RAW	dark	2	2a
55	75	LVPDGK[+648.3]YGAQNDKGEWNGMVK	985.467851	3	848.891028	2	y15	52.47	308	5882	3	2311_NC_dark9_02_targeted_b.RAW	dark	2	2b
55	75	LVPDGK[+648.3]YGAQNDKGEWNGMVK	985.467851	3	848.891028	2	y15	52.29	538	9261	2	2311_NC_dark9_03_targeted.RAW	dark	3	3a
55	75	LVPDGK[+648.3]YGAQNDKGEWNGMVK	985.467851	3	848.891028	2	y15	52.54	556	10526	2	2311_NC_dark9_03_targeted_b.RAW	dark	3	3b
55	75	LVPDGK[+648.3]YGAQNDKGEWNGMVK	985.467851	3	848.891028	2	y15	52.47	211	3337	3	2311_NC_UV9_04_targeted.RAW	UV	4	4a
55	75	LVPDGK[+648.3]YGAQNDKGEWNGMVK	985.467851	3	848.891028	2	y15	52.65	172	3007	3	2311_NC_UV9_04_targeted_b.RAW	UV	4	4b
55	75	LVPDGK[+648.3]YGAQNDKGEWNGMVK	985.467851	3	848.891028	2	y15	52.48	136	2759	3	2311_NC_UV9_05_targeted.RAW	UV	5	5a
55	75	LVPDGK[+648.3]YGAQNDKGEWNGMVK	985.467851	3	848.891028	2	y15	52.88	118	2186	3	2311_NC_UV9_05_targeted_b.RAW	UV	5	5b
55	75	LVPDGK[+648.3]YGAQNDKGEWNGMVK	985.467851	3	848.891028	2	y15	52.59	94	1643	3	2311_NC_UV9_06_targeted.RAW	UV	6	6a
55	75	LVPDGK[+648.3]YGAQNDKGEWNGMVK	985.467851	3	848.891028	2	y15	52.71	136	1928	3	2311_NC_UV9_06_targeted_b.RAW	UV	6	6b
55	75	LVPDGK[+648.3]YGAQNDKGEWNGMVK	985.467851	3	767.359364	2	y14	52.36	190	3643	3	2311_NC_dark9_01_targeted.RAW	dark	1	1a
55	75	LVPDGK[+648.3]YGAQNDKGEWNGMVK	985.467851	3	767.359364	2	y14	52.52	394	6977	2	2311_NC_dark9_01_targeted_b.RAW	dark	1	1b
55	75	LVPDGK[+648.3]YGAQNDKGEWNGMVK	985.467851	3	767.359364	2	y14	52.46	409	6196	2	2311_NC_dark9_02_targeted.RAW	dark	2	2a
55	75	LVPDGK[+648.3]YGAQNDKGEWNGMVK	985.467851	3	767.359364	2	y14	52.53	325	6298	2	2311_NC_dark9_02_targeted_b.RAW	dark	2	2b
55	75	LVPDGK[+648.3]YGAQNDKGEWNGMVK	985.467851	3	767.359364	2	y14	52.29	564	8549	3	2311_NC_dark9_03_targeted.RAW	dark	3	3a
55	75	LVPDGK[+648.3]YGAQNDKGEWNGMVK	985.467851	3	767.359364	2	y14	52.54	578	10337	3	2311_NC_dark9_03_targeted_b.RAW	dark	3	3b
55	75	LVPDGK[+648.3]YGAQNDKGEWNGMVK	985.467851	3	767.359364	2	y14	52.52	207	3560	2	2311_NC_UV9_04_targeted.RAW	UV	4	4a
55	75	LVPDGK[+648.3]YGAQNDKGEWNGMVK	985.467851	3	767.359364	2	y14	52.65	212	3063	2	2311_NC_UV9_04_targeted_b.RAW	UV	4	4b
55	75	LVPDGK[+648.3]YGAQNDKGEWNGMVK	985.467851	3	767.359364	2	y14	52.54	183	3308	2	2311_NC_UV9_05_targeted.RAW	UV	5	5a
55	75	LVPDGK[+648.3]YGAQNDKGEWNGMVK	985.467851	3	767.359364	2	y14	52.82	195	2660	2	2311_NC_UV9_05_targeted_b.RAW	UV	5	5b
55	75	LVPDGK[+648.3]YGAQNDKGEWNGMVK	985.467851	3	767.359364	2	y14	52.59	93	1690	2	2311_NC_UV9_06_targeted.RAW	UV	6	6a
55	75	LVPDGK[+648.3]YGAQNDKGEWNGMVK	985.467851	3	767.359364	2	y14	52.71	131	2068	2	2311_NC_UV9_06_targeted_b.RAW	UV	6	6b
165	179	QQSALVKNSDEGIQR	836.934282	2	1046.522512	1	y9	29.43	221	3145	2	2311_NC_dark9_01_targeted.RAW	dark	1	1a
165	179	QQSALVKNSDEGIQR	836.934282	2	1046.522512	1	y9	29.25	214	3002	3	2311_NC_dark9_01_targeted_b.RAW	dark	1	1b
165	179	QQSALVKNSDEGIQR	836.934282	2	1046.522512	1	y9	29.32	423	6537	3	2311_NC_dark9_02_targeted.RAW	dark	2	2a
165	179	QQSALVKNSDEGIQR	836.934282	2	1046.522512	1	y9	29.54	471	5563	2	2311_NC_dark9_02_targeted_b.RAW	dark	2	2b
165	179	QQSALVKNSDEGIQR	836.934282	2	1046.522512	1	y9	29.38	1218	15739	2	2311_NC_dark9_03_targeted.RAW	dark	3	3a
165	179	QQSALVKNSDEGIQR	836.934282	2	1046.522512	1	y9	29.37	1601	18896	2	2311_NC_dark9_03_targeted_b.RAW	dark	3	3b
165	179	QQSALVKNSDEGIQR	836.934282	2	1046.522512	1	y9	29.32	1255	15146	2	2311_NC_UV9_04_targeted.RAW	UV	4	4a
165	179	QQSALVKNSDEGIQR	836.934282	2	1046.522512	1	y9	29.48	813	7376	3	2311_NC_UV9_04_targeted_b.RAW	UV	4	4b
165	179	QQSALVKNSDEGIQR	836.934282	2	1046.522512	1	y9	29.32	21	397	3	2311_NC_UV9_05_targeted.RAW	UV	5	5a
165	179	QQSALVKNSDEGIQR	836.934282	2	1046.522512	1	y9	29.6	1289	15609	2	2311_NC_UV9_05_targeted_b.RAW	UV	5	5b
165	179	QQSALVKNSDEGIQR	836.934282	2	1046.522512	1	y9	29.6	400	3761	3	2311_NC_UV9_06_targeted.RAW	UV	6	6a
165	179	QQSALVKNSDEGIQR	836.934282	2	1046.522512	1	y9	29.48	5	92	3	2311_NC_UV9_06_targeted_b.RAW	UV	6	6b
165	179	QQSALVKNSDEGIQR	836.934282	2	918.427549	1	y8	29.43	215	3079	3	2311_NC_dark9_01_targeted.RAW	dark	1	1a
165	179	QQSALVKNSDEGIQR	836.934282	2	918.427549	1	y8	29.25	218	3014	2	2311_NC_dark9_01_targeted_b.RAW	dark	1	1b
165	179	QQSALVKNSDEGIQR	836.934282	2	918.427549	1	y8	29.32	478	6814	2	2311_NC_dark9_02_targeted.RAW	dark	2	2a
165	179	QQSALVKNSDEGIQR	836.934282	2	918.427549	1	y8	29.54	475	5432	3	2311_NC_dark9_02_targeted_b.RAW	dark	2	2b
165	179	QQSALVKNSDEGIQR	836.934282	2	918.427549	1	y8	29.38	1175	15198	3	2311_NC_dark9_03_targeted.RAW	dark	3	3a
165	179	QQSALVKNSDEGIQR	836.934282	2	918.427549	1	y8	29.37	1416	16961	3	2311_NC_dark9_03_targeted_b.RAW	dark	3	3b
165	179	QQSALVKNSDEGIQR	836.934282	2	918.427549	1	y8	29.32	1131	14001	3	2311_NC_UV9_04_targeted.RAW	UV	4	4a
165	179	QQSALVKNSDEGIQR	836.934282	2	918.427549	1	y8	29.48	815	7501	2	2311_NC_UV9_04_targeted_b.RAW	UV	4	4b
165	179	QQSALVKNSDEGIQR	836.934282	2	918.427549	1	y8	29.32	25	439	2	2311_NC_UV9_05_targeted.RAW	UV	5	5a
165	179	QQSALVKNSDEGIQR	836.934282	2	918.427549	1	y8	29.66	1227	14826	3	2311_NC_UV9_05_targeted_b.RAW	UV	5	5b
165	179	QQSALVKNSDEGIQR	836.934282	2	918.427549	1	y8	29.6	394	3868	2	2311_NC_UV9_06_targeted.RAW	UV	6	6a
165	179	QQSALVKNSDEGIQR	836.934282	2	918.427549	1	y8	29.66	11	142	2	2311_NC_UV9_06_targeted_b.RAW	UV	6	6b
165	179	QQSALVKNSDEGIQR	836.934282	2	602.32565	1	y5	30.32	622	15175	1	2311_NC_dark9_01_targeted.RAW	dark	1	1a
165	179	QQSALVKNSDEGIQR	836.934282	2	602.32565	1	y5	29.25	518	11447	1	2311_NC_dark9_01_targeted_b.RAW	dark	1	1b
165	179	QQSALVKNSDEGIQR	836.934282	2	602.32565	1	y5	29.32	1006	20172	1	2311_NC_dark9_02_targeted.RAW	dark	2	2a
165	179	QQSALVKNSDEGIQR	836.934282	2	602.32565	1	y5	29.54	991	12656	1	2311_NC_dark9_02_targeted_b.RAW	dark	2	2b
165	179	QQSALVKNSDEGIQR	836.934282	2	602.32565	1	y5	29.38	2203	35858	1	2311_NC_dark9_03_targeted.RAW	dark	3	3a
165	179	QQSALVKNSDEGIQR	836.934282	2	602.32565	1	y5	29.37	3331	42098	1	2311_NC_dark9_03_targeted_b.RAW	dark	3	3b
165	179	QQSALVKNSDEGIQR	836.934282	2	602.32565	1	y5	29.32	2867	33865	1	2311_NC_UV9_04_targeted.RAW	UV	4	4a
165	179	QQSALVKNSDEGIQR	836.934282	2	602.32565	1	y5	29.48	1643	20312	1	2311_NC_UV9_04_targeted_b.RAW	UV	4	4b
165	179	QQSALVKNSDEGIQR	836.934282	2	602.32565	1	y5	29.26	33	1221	1	2311_NC_UV9_05_targeted.RAW	UV	5	5a
165	179	QQSALVKNSDEGIQR	836.934282	2	602.32565	1	y5	29.66	2787	33400	1	2311_NC_UV9_05_targeted_b.RAW	UV	5	5b
165	179	QQSALVKNSDEGIQR	836.934282	2	602.32565	1	y5	29.6	930	12859	1	2311_NC_UV9_06_targeted.RAW	UV	6	6a

165	179	QQSALVKNSDEGIQR	836.934282	2	602.32565	1	y5	29.48	11	335	1	2311_NC_UV9_06_targeted_b.RAW	UV	6	6b
165	179	QQSALVKNSDEGIQR	558.291946	3	602.32565	1	y5	29.43	488	13775	3	2311_NC_dark9_01_targeted.RAW	dark	1	1a
165	179	QQSALVKNSDEGIQR	558.291946	3	602.32565	1	y5	29.25	526	8688	3	2311_NC_dark9_01_targeted_b.RAW	dark	1	1b
165	179	QQSALVKNSDEGIQR	558.291946	3	602.32565	1	y5	29.32	960	18113	3	2311_NC_dark9_02_targeted.RAW	dark	2	2a
165	179	QQSALVKNSDEGIQR	558.291946	3	602.32565	1	y5	29.54	823	11825	3	2311_NC_dark9_02_targeted_b.RAW	dark	2	2b
165	179	QQSALVKNSDEGIQR	558.291946	3	602.32565	1	y5	29.38	2261	35994	3	2311_NC_dark9_03_targeted.RAW	dark	3	3a
165	179	QQSALVKNSDEGIQR	558.291946	3	602.32565	1	y5	29.37	2468	36831	3	2311_NC_dark9_03_targeted_b.RAW	dark	3	3b
165	179	QQSALVKNSDEGIQR	558.291946	3	602.32565	1	y5	29.32	2237	31169	3	2311_NC_UV9_04_targeted.RAW	UV	4	4a
165	179	QQSALVKNSDEGIQR	558.291946	3	602.32565	1	y5	29.48	1367	18221	3	2311_NC_UV9_04_targeted_b.RAW	UV	4	4b
165	179	QQSALVKNSDEGIQR	558.291946	3	602.32565	1	y5	29.26	41	1568	3	2311_NC_UV9_05_targeted.RAW	UV	5	5a
165	179	QQSALVKNSDEGIQR	558.291946	3	602.32565	1	y5	29.6	1686	23359	3	2311_NC_UV9_05_targeted_b.RAW	UV	5	5b
165	179	QQSALVKNSDEGIQR	558.291946	3	602.32565	1	y5	29.6	993	15894	3	2311_NC_UV9_06_targeted.RAW	UV	6	6a
165	179	QQSALVKNSDEGIQR	558.291946	3	602.32565	1	y5	29.66	14	431	2	2311_NC_UV9_06_targeted_b.RAW	UV	6	6b
165	179	QQSALVKNSDEGIQR	558.291946	3	708.875704	2	y13	29.43	590	17841	2	2311_NC_dark9_01_targeted.RAW	dark	1	1a
165	179	QQSALVKNSDEGIQR	558.291946	3	708.875704	2	y13	29.25	556	9371	2	2311_NC_dark9_01_targeted_b.RAW	dark	1	1b
165	179	QQSALVKNSDEGIQR	558.291946	3	708.875704	2	y13	29.26	1178	23046	2	2311_NC_dark9_02_targeted.RAW	dark	2	2a
165	179	QQSALVKNSDEGIQR	558.291946	3	708.875704	2	y13	29.54	908	13759	2	2311_NC_dark9_02_targeted_b.RAW	dark	2	2b
165	179	QQSALVKNSDEGIQR	558.291946	3	708.875704	2	y13	29.38	2660	41055	2	2311_NC_dark9_03_targeted.RAW	dark	3	3a
165	179	QQSALVKNSDEGIQR	558.291946	3	708.875704	2	y13	29.37	2603	42607	2	2311_NC_dark9_03_targeted_b.RAW	dark	3	3b
165	179	QQSALVKNSDEGIQR	558.291946	3	708.875704	2	y13	29.32	2426	33612	2	2311_NC_UV9_04_targeted.RAW	UV	4	4a
165	179	QQSALVKNSDEGIQR	558.291946	3	708.875704	2	y13	29.48	1538	22876	2	2311_NC_UV9_04_targeted_b.RAW	UV	4	4b
165	179	QQSALVKNSDEGIQR	558.291946	3	708.875704	2	y13	30.03	46	1838	2	2311_NC_UV9_05_targeted.RAW	UV	5	5a
165	179	QQSALVKNSDEGIQR	558.291946	3	708.875704	2	y13	29.6	1767	23943	2	2311_NC_UV9_05_targeted_b.RAW	UV	5	5b
165	179	QQSALVKNSDEGIQR	558.291946	3	708.875704	2	y13	29.6	1120	18219	2	2311_NC_UV9_06_targeted.RAW	UV	6	6a
165	179	QQSALVKNSDEGIQR	558.291946	3	708.875704	2	y13	29.54	11	396	3	2311_NC_UV9_06_targeted_b.RAW	UV	6	6b
165	179	QQSALVKNSDEGIQR	558.291946	3	523.764894	2	y9	29.43	987	26581	1	2311_NC_dark9_01_targeted.RAW	dark	1	1a
165	179	QQSALVKNSDEGIQR	558.291946	3	523.764894	2	y9	29.25	982	17284	1	2311_NC_dark9_01_targeted_b.RAW	dark	1	1b
165	179	QQSALVKNSDEGIQR	558.291946	3	523.764894	2	y9	29.26	1841	36284	1	2311_NC_dark9_02_targeted.RAW	dark	2	2a
165	179	QQSALVKNSDEGIQR	558.291946	3	523.764894	2	y9	29.54	1404	21056	1	2311_NC_dark9_02_targeted_b.RAW	dark	2	2b
165	179	QQSALVKNSDEGIQR	558.291946	3	523.764894	2	y9	29.38	4145	66954	1	2311_NC_dark9_03_targeted.RAW	dark	3	3a
165	179	QQSALVKNSDEGIQR	558.291946	3	523.764894	2	y9	29.37	4485	66478	1	2311_NC_dark9_03_targeted_b.RAW	dark	3	3b
165	179	QQSALVKNSDEGIQR	558.291946	3	523.764894	2	y9	29.32	4306	55629	1	2311_NC_UV9_04_targeted.RAW	UV	4	4a
165	179	QQSALVKNSDEGIQR	558.291946	3	523.764894	2	y9	29.48	2362	34540	1	2311_NC_UV9_04_targeted_b.RAW	UV	4	4b
165	179	QQSALVKNSDEGIQR	558.291946	3	523.764894	2	y9	30.03	79	2603	1	2311_NC_UV9_05_targeted.RAW	UV	5	5a
165	179	QQSALVKNSDEGIQR	558.291946	3	523.764894	2	y9	29.6	3109	40615	1	2311_NC_UV9_05_targeted_b.RAW	UV	5	5b
165	179	QQSALVKNSDEGIQR	558.291946	3	523.764894	2	y9	29.6	1652	30263	1	2311_NC_UV9_06_targeted.RAW	UV	6	6a
165	179	QQSALVKNSDEGIQR	558.291946	3	523.764894	2	y9	29.48	27	1029	1	2311_NC_UV9_06_targeted_b.RAW	UV	6	6b
165	179	QQSALVK[+648.3]NSDEGIQR	1161.067087	2	918.427549	1	y8	49.93	1914	23763	1	2311_NC_dark9_01_targeted.RAW	dark	1	1a
165	179	QQSALVK[+648.3]NSDEGIQR	1161.067087	2	918.427549	1	y8	49.85	3188	45915	1	2311_NC_dark9_01_targeted_b.RAW	dark	1	1b
165	179	QQSALVK[+648.3]NSDEGIQR	1161.067087	2	918.427549	1	y8	49.91	2581	39182	1	2311_NC_dark9_02_targeted.RAW	dark	2	2a
165	179	QQSALVK[+648.3]NSDEGIQR	1161.067087	2	918.427549	1	y8	50.1	2783	42488	1	2311_NC_dark9_02_targeted_b.RAW	dark	2	2b
165	179	QQSALVK[+648.3]NSDEGIQR	1161.067087	2	918.427549	1	y8	49.92	2883	44755	1	2311_NC_dark9_03_targeted.RAW	dark	3	3a
165	179	QQSALVK[+648.3]NSDEGIQR	1161.067087	2	918.427549	1	y8	49.87	3509	55471	1	2311_NC_dark9_03_targeted_b.RAW	dark	3	3b
165	179	QQSALVK[+648.3]NSDEGIQR	1161.067087	2	918.427549	1	y8	49.97	3412	50568	1	2311_NC_UV9_04_targeted.RAW	UV	4	4a
165	179	QQSALVK[+648.3]NSDEGIQR	1161.067087	2	918.427549	1	y8	50.04	2662	37485	1	2311_NC_UV9_04_targeted_b.RAW	UV	4	4b
165	179	QQSALVK[+648.3]NSDEGIQR	1161.067087	2	918.427549	1	y8	49.99	2902	42803	1	2311_NC_UV9_05_targeted.RAW	UV	5	5a
165	179	QQSALVK[+648.3]NSDEGIQR	1161.067087	2	918.427549	1	y8	50.15	2765	40784	1	2311_NC_UV9_05_targeted_b.RAW	UV	5	5b
165	179	QQSALVK[+648.3]NSDEGIQR	1161.067087	2	918.427549	1	y8	50.04	2264	30733	1	2311_NC_UV9_06_targeted.RAW	UV	6	6a
165	179	QQSALVK[+648.3]NSDEGIQR	1161.067087	2	918.427549	1	y8	49.98	801	12503	1	2311_NC_UV9_06_targeted_b.RAW	UV	6	6b
165	179	QQSALVK[+648.3]NSDEGIQR	1161.067087	2	804.384622	1	y7	49.93	835	10998	2	2311_NC_dark9_01_targeted.RAW	dark	1	1a
165	179	QQSALVK[+648.3]NSDEGIQR	1161.067087	2	804.384622	1	y7	49.85	1371	19684	2	2311_NC_dark9_01_targeted_b.RAW	dark	1	1b
165	179	QQSALVK[+648.3]NSDEGIQR	1161.067087	2	804.384622	1	y7	49.91	1091	16310	2	2311_NC_dark9_02_targeted.RAW	dark	2	2a
165	179	QQSALVK[+648.3]NSDEGIQR	1161.067087	2	804.384622	1	y7	50.1	1254	19216	2	2311_NC_dark9_02_targeted_b.RAW	dark	2	2b
165	179	QQSALVK[+648.3]NSDEGIQR	1161.067087	2	804.384622	1	y7	49.86	1220	18076	2	2311_NC_dark9_03_targeted.RAW	dark	3	3a
165	179	QQSALVK[+648.3]NSDEGIQR	1161.067087	2	804.384622	1	y7	49.87	1540	23940	2	2311_NC_dark9_03_targeted_b.RAW	dark	3	3b
165	179	QQSALVK[+648.3]NSDEGIQR	1161.067087	2	804.384622	1	y7	49.97	1356	21686	2	2311_NC_UV9_04_targeted.RAW	UV	4	4a
165	179	QQSALVK[+648.3]NSDEGIQR	1161.067087	2	804.384622	1	y7	49.98	1174	16901	2	2311_NC_UV9_04_targeted_b.RAW	UV	4	4b
165	179	QQSALVK[+648.3]NSDEGIQR	1161.067087	2	804.384622	1	y7	49.99	1189	16993	2	2311_NC_UV9_05_targeted.RAW	UV	5	5a
165	179	QQSALVK[+648.3]NSDEGIQR	1161.067087	2	804.384622	1	y7	50.21	1089	17681	2	2311_NC_UV9_05_targeted_b.RAW	UV	5	5b
165	179	QQSALVK[+648.3]NSDEGIQR	1161.067087	2	804.384622	1	y7	50.04	952	13052	2	2311_NC_UV9_06_targeted.RAW	UV	6	6a
165	179	QQSALVK[+648.3]NSDEGIQR	1161.067087	2	804.384622	1	y7	49.98	352	5492	2	2311_NC_UV9_06_targeted_b.RAW	UV	6	6b
165	179	QQSALVK[+648.3]NSDEGIQR	1161.067087	2	602.32565	1	y5	49.93	737	8470	3	2311_NC_dark9_01_targeted.RAW	dark	1	1a
165	179	QQSALVK[+648.3]NSDEGIQR	1161.067087	2	602.32565	1	y5	49.85	1087	15703	3	2311_NC_dark9_01_targeted_b.RAW	dark	1	1b
165	179	QQSALVK[+648.3]NSDEGIQR	1161.067087	2	602.32565	1	y5	49.91	958	13616	3	2311_NC_dark9_02_targeted.RAW	dark	2	2a

165	179	QQSALVK[+648.3]NSDEGIQR	1161.067087	2	602.32565	1	y5	50.1	995	14971	3	2311_NC_dark9_02_targeted_b.RAW	dark	2	2b
165	179	QQSALVK[+648.3]NSDEGIQR	1161.067087	2	602.32565	1	y5	49.92	960	14690	3	2311_NC_dark9_03_targeted.RAW	dark	3	3a
165	179	QQSALVK[+648.3]NSDEGIQR	1161.067087	2	602.32565	1	y5	49.87	1304	20597	3	2311_NC_dark9_03_targeted_b.RAW	dark	3	3b
165	179	QQSALVK[+648.3]NSDEGIQR	1161.067087	2	602.32565	1	y5	49.97	1126	17602	3	2311_NC_UV9_04_targeted.RAW	UV	4	4a
165	179	QQSALVK[+648.3]NSDEGIQR	1161.067087	2	602.32565	1	y5	50.04	937	13353	3	2311_NC_UV9_04_targeted_b.RAW	UV	4	4b
165	179	QQSALVK[+648.3]NSDEGIQR	1161.067087	2	602.32565	1	y5	49.99	1041	14843	3	2311_NC_UV9_05_targeted.RAW	UV	5	5a
165	179	QQSALVK[+648.3]NSDEGIQR	1161.067087	2	602.32565	1	y5	50.15	976	14083	3	2311_NC_UV9_05_targeted_b.RAW	UV	5	5b
165	179	QQSALVK[+648.3]NSDEGIQR	1161.067087	2	602.32565	1	y5	50.04	707	10562	3	2311_NC_UV9_06_targeted.RAW	UV	6	6a
165	179	QQSALVK[+648.3]NSDEGIQR	1161.067087	2	602.32565	1	y5	49.98	277	4177	3	2311_NC_UV9_06_targeted_b.RAW	UV	6	6b
165	179	QQSALVK[+648.3]NSDEGIQR	774.380483	3	918.427549	1	y8	49.93	23190	284513	1	2311_NC_dark9_01_targeted.RAW	dark	1	1a
165	179	QQSALVK[+648.3]NSDEGIQR	774.380483	3	918.427549	1	y8	49.85	28406	322809	1	2311_NC_dark9_01_targeted_b.RAW	dark	1	1b
165	179	QQSALVK[+648.3]NSDEGIQR	774.380483	3	918.427549	1	y8	49.91	35020	427901	1	2311_NC_dark9_02_targeted.RAW	dark	2	2a
165	179	QQSALVK[+648.3]NSDEGIQR	774.380483	3	918.427549	1	y8	50.1	26569	368644	1	2311_NC_dark9_02_targeted_b.RAW	dark	2	2b
165	179	QQSALVK[+648.3]NSDEGIQR	774.380483	3	918.427549	1	y8	49.86	43976	599881	1	2311_NC_dark9_03_targeted.RAW	dark	3	3a
165	179	QQSALVK[+648.3]NSDEGIQR	774.380483	3	918.427549	1	y8	49.87	41460	594620	1	2311_NC_dark9_03_targeted_b.RAW	dark	3	3b
165	179	QQSALVK[+648.3]NSDEGIQR	774.380483	3	918.427549	1	y8	49.97	45144	601362	1	2311_NC_UV9_04_targeted.RAW	UV	4	4a
165	179	QQSALVK[+648.3]NSDEGIQR	774.380483	3	918.427549	1	y8	49.98	17201	251239	1	2311_NC_UV9_04_targeted_b.RAW	UV	4	4b
165	179	QQSALVK[+648.3]NSDEGIQR	774.380483	3	918.427549	1	y8	49.99	34209	463373	1	2311_NC_UV9_05_targeted.RAW	UV	5	5a
165	179	QQSALVK[+648.3]NSDEGIQR	774.380483	3	918.427549	1	y8	50.15	23087	345191	1	2311_NC_UV9_05_targeted_b.RAW	UV	5	5b
165	179	QQSALVK[+648.3]NSDEGIQR	774.380483	3	918.427549	1	y8	50.04	22071	295333	1	2311_NC_UV9_06_targeted.RAW	UV	6	6a
165	179	QQSALVK[+648.3]NSDEGIQR	774.380483	3	918.427549	1	y8	49.98	8889	138213	1	2311_NC_UV9_06_targeted_b.RAW	UV	6	6b
165	179	QQSALVK[+648.3]NSDEGIQR	774.380483	3	473.283057	1	y4	49.85	10630	121424	3	2311_NC_dark9_01_targeted_b.RAW	dark	1	1b
165	179	QQSALVK[+648.3]NSDEGIQR	774.380483	3	473.283057	1	y4	50.1	10199	138159	3	2311_NC_dark9_02_targeted_b.RAW	dark	2	2b
165	179	QQSALVK[+648.3]NSDEGIQR	774.380483	3	473.283057	1	y4	49.87	16000	226985	3	2311_NC_dark9_03_targeted_b.RAW	dark	3	3b
165	179	QQSALVK[+648.3]NSDEGIQR	774.380483	3	473.283057	1	y4	50.04	6794	96259	3	2311_NC_UV9_04_targeted_b.RAW	UV	4	4b
165	179	QQSALVK[+648.3]NSDEGIQR	774.380483	3	473.283057	1	y4	50.15	8908	131542	3	2311_NC_UV9_05_targeted_b.RAW	UV	5	5b
165	179	QQSALVK[+648.3]NSDEGIQR	774.380483	3	473.283057	1	y4	49.98	3113	51314	3	2311_NC_UV9_06_targeted_b.RAW	UV	6	6b
165	179	QQSALVK[+648.3]NSDEGIQR	774.380483	3	847.897699	2	y9	49.93	13577	167036	2	2311_NC_dark9_01_targeted.RAW	dark	1	1a
165	179	QQSALVK[+648.3]NSDEGIQR	774.380483	3	847.897699	2	y9	49.85	18794	210584	2	2311_NC_dark9_01_targeted_b.RAW	dark	1	1b
165	179	QQSALVK[+648.3]NSDEGIQR	774.380483	3	847.897699	2	y9	49.91	18793	256689	2	2311_NC_dark9_02_targeted.RAW	dark	2	2a
165	179	QQSALVK[+648.3]NSDEGIQR	774.380483	3	847.897699	2	y9	50.1	17542	238267	2	2311_NC_dark9_02_targeted_b.RAW	dark	2	2b
165	179	QQSALVK[+648.3]NSDEGIQR	774.380483	3	847.897699	2	y9	49.86	20118	304404	2	2311_NC_dark9_03_targeted.RAW	dark	3	3a
165	179	QQSALVK[+648.3]NSDEGIQR	774.380483	3	847.897699	2	y9	49.87	27327	383569	2	2311_NC_dark9_03_targeted_b.RAW	dark	3	3b
165	179	QQSALVK[+648.3]NSDEGIQR	774.380483	3	847.897699	2	y9	49.91	16695	275572	2	2311_NC_UV9_04_targeted.RAW	UV	4	4a
165	179	QQSALVK[+648.3]NSDEGIQR	774.380483	3	847.897699	2	y9	50.04	9905	130899	2	2311_NC_UV9_04_targeted_b.RAW	UV	4	4b
165	179	QQSALVK[+648.3]NSDEGIQR	774.380483	3	847.897699	2	y9	49.99	22357	292416	2	2311_NC_UV9_05_targeted.RAW	UV	5	5a
165	179	QQSALVK[+648.3]NSDEGIQR	774.380483	3	847.897699	2	y9	50.21	10502	176785	2	2311_NC_UV9_05_targeted_b.RAW	UV	5	5b
165	179	QQSALVK[+648.3]NSDEGIQR	774.380483	3	847.897699	2	y9	50.04	13932	173385	2	2311_NC_UV9_06_targeted.RAW	UV	6	6a
165	179	QQSALVK[+648.3]NSDEGIQR	774.380483	3	847.897699	2	y9	49.98	5338	81982	2	2311_NC_UV9_06_targeted_b.RAW	UV	6	6b

Supplementary Notes 1. Chemical Synthesis

General Methods.

Solvents were dried prior to use with alumina in a solvent purification system or distilled and dried by standard methods. FT-IR spectra are reported in cm^{-1}. ^{1}H and ^{13}C NMR spectra were obtained in $CDCl_3$, CD_3OD or DMSO-d_6 solutions at 500 MHz (for ^{1}H) and 100 MHz (for ^{13}C). Chemical shifts (δ) are reported in ppm relative to the singlet at 7.26 ppm of $CDCl_3$ for ^{1}H and in ppm relative to the center line of a triplet at 77.16 ppm of $CDCl_3$ for ^{13}C. Optical rotations were measured with a Jasco P-1030 polarimeter, and specific rotations are reported in 10^{-1} deg cm^2 g^{-1}. The HRMS spectra were recorded on a Waters LCT Premier Mass spectrometer with electrospray ionization (ESI). Melting points were measured on a Mettler Toledo MP70 melting point apparatus. UV-Vis analyses were performed in a UV-1700 PharmaSpec SHIMADZU UV-Vis spectrometer on a 50 μM solution of the corresponding compound in HPLC-quality solvent. All the starting materials for the synthesis of the azobenzenes and Alkyne-NHS Esters were obtained from commercial suppliers and were used without further purifications. 4-Azidoaniline **25** was prepared from 4-iodoaniline according to the procedure of Wei *et al*.[1] (Scheme S2). Alkyne-NHS Ester **8** and epoxide-NHS Ester **3** were purchased from Alfa Aesar and Sigma-Aldrich, respectively.

Synthesis of azobenzenes 9 – 20

The preparation of the final compounds **9** – **20** is shown in Scheme S1. The azides **1** and **2** were synthesized from azobenzenes **21** and **22**, respectively. Azobenzene **21** was prepared from commercially available 4,4'-azodianiline and azobenzene **22** was obtained using classical diazo-coupling chemistry (Scheme S2).

Amide coupling of **21** and **22** with a pyroglutamate acid derivative[2, 3] gave the advanced intermediates **23** and **24** in 78% and 57% yield, respectively. Then, these compounds were transformed into the final azides **1** and **2** after hydrolysis of the pyroglutamate with concomitant saponification of the ethyl ester and acidic removal of the *tert*-butoxycarbonyl (Boc) protecting group (Scheme S1 step b) in 73% and 43% yield, respectively. Triazole compounds **9**–**20** were obtained by click chemistry using the Cu(I) catalyzed azide-alkyne cycloaddition reaction[4] by mixing in H_2O/THF (7.5:1), 4 equivalents of sodium ascorbate (NaAsc), 2.4 equivalents of the solid Cu_2O, and 1 equivalent of azide **1** or **2**. After stirring for 5 min at room temperature, 1.1 equivalents of the alkyne compounds **3**–**8** (Scheme S1 squared figure) in THF were added. The reaction was stirred for 30 min at room temperature (step c).

Scheme S1. Synthesis of azobenzenes **9 – 20**.[a]

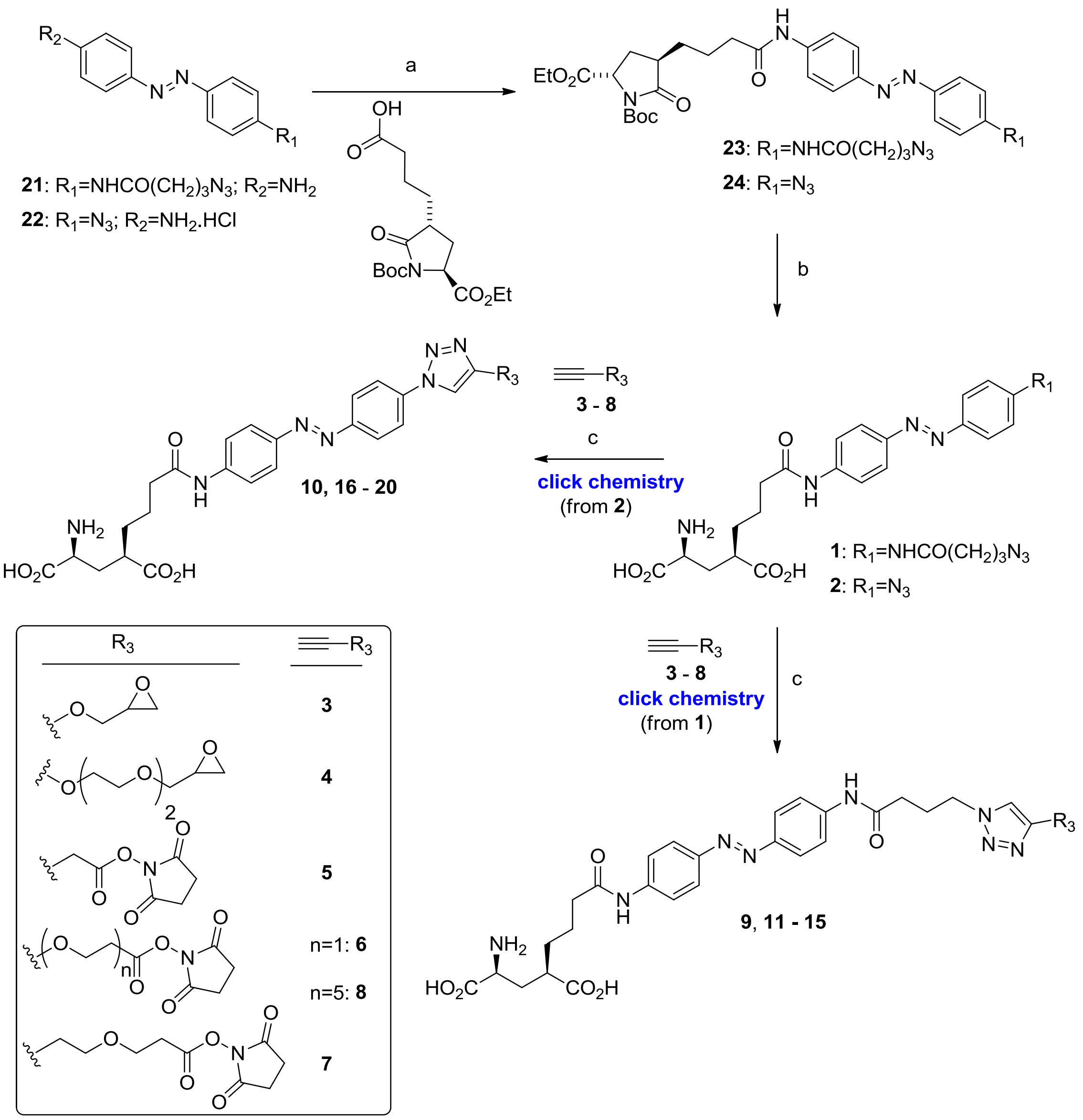

[a]Reagent and conditions: (a) HATU, DIPEA, EtOAc; (b) 1. LiOH, THF/H_2O. 2. HCl, EtOAc; (c) Cu_2O, NaAsc, THF/H_2O.

Synthesis of azobenzenes 21 and 22

The preparation of azobenzenes **21** and **22** is shown in Scheme S2. To outline the strategy of the non-commercially available azo block compounds, we used two different routes. Azo-compound **21** was synthesized by monoacylation of the commercially available 4,4'-diaminoazobenzene with 4-bromobutyryl chloride, followed by nucleophilic substitution of the bromide with sodium azide in the presence of TBAI. The azo-compound **21** was obtained in 79% yield.

Azo compound **22** was obtained from 4-iodoaniline, which was converted into 4-azidoaniline **25** by the substitution of the iodide with the azido group (Scheme S2 step

b) in a nearly quantitative yield. Then, compound **25** was coupled with nitroso compound **26** (Scheme S2 step d), which was obtained from commercially available *tert*-butyl (4-aminophenyl)carbamate (step c, 47% yield), in acetic acid media to give the resulting azo compound **27** in 91% yield. Further acidic deprotection of *tert*-butoxycarbonyl (Boc) group afforded in 90% yield the desired azo compound **22**.

Scheme S2. Synthesis of azobenzenes **21** and **22**.[a]

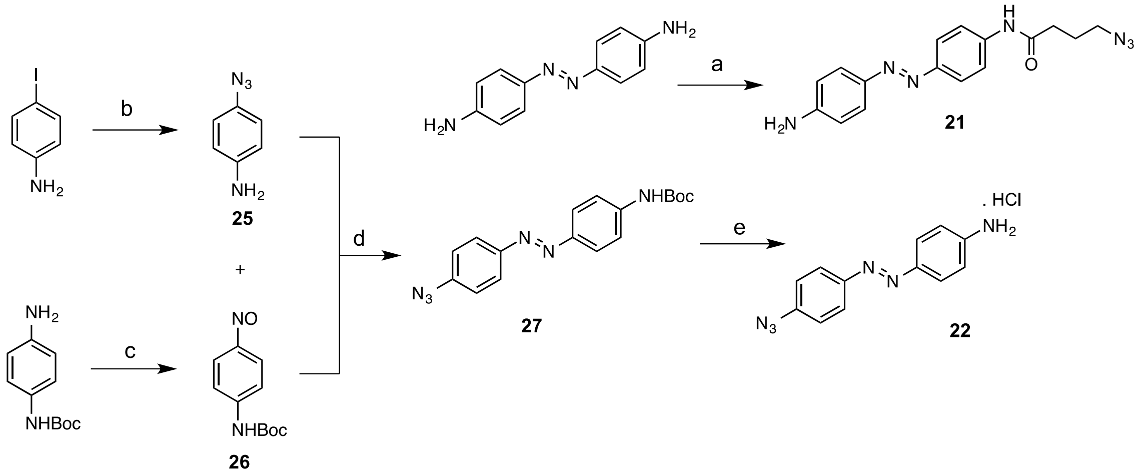

[a]Reagent and conditions: (a) 1. 4-bromobutyryl chloride, DIPEA, THF, rt. 2. NaN_3, TBAI, DMF, 70 ºC ; (b) NaN_3, 10 mol% CuI, 20 mol% L-proline, 20 mol% NaOH, DMSO, 60 ºC; (c) oxone, CH_2Cl_2, rt; (d) AcOH, rt; (e) HCl, EtOAc.

(*E*)-*N*-(4-((4-Aminophenyl)diazenyl)phenyl)-4-azidobutanamide (21).

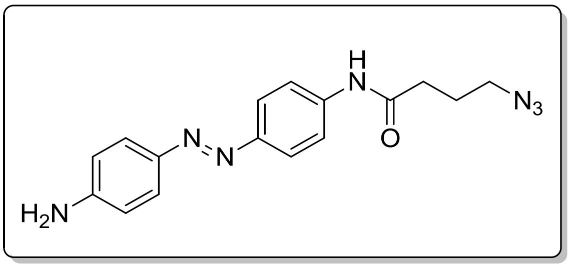

To a stirred solution of 4,4'-diaminoazobenzene (1.0 g, 4.71 mmol) and DIPEA (0.49 mL, 2.83 mmol) in THF (40 mL) was added a solution of 4-bromobutyryl chloride (0.27 mL, 2.36 mmol) in THF (20 mL) at 0 ºC. The reaction mixture was stirred at room temperature for 20 h, followed by removal of solvent *in vacuo*. The residue was suspended in H_2O and extracted with EtOAc (3 x 100 mL). The combined organic layers were washed with brine, dried over $MgSO_4$, filtered and concentrated. The resulting crude was directly used in the next step without further purification.

To a solution of the above crude in DMF (50 mL) were added tetrabutylammonium iodide (TBAI) (0.43 g, 1.18 mmol) and NaN_3 (0.61 g, 9.41 mmol). The reaction mixture was stirred at 70 ºC for 24 h. The reaction mixture was cooled to room temperature and

the solvent was removed under vacuum. The residue was taken up in EtOAc (60 mL) and washed with water (30 mL). The organic layer was washed brine (3 x 30 mL), dried over $MgSO_4$, filtered and the solvent was removed under reduced pressure. The resulting residue was purified by flash chromatography (95:5 to 10:1 CH_2Cl_2/EtOAc gradient) to afford 0.60 g (1.86 mmol, 79%) of **21**. mp: 155–157 ºC R_f: 0.5 (CH_2Cl_2/EtOAc 90:10). IR (film): υ = 3429, 3293, 3202, 2097, 1659, 1595, 1580, 1536, 1403, 1356, 1243, 848, 832, 639 cm^{-1}. ^1H NMR (*δ*, 500 MHz, CD_3OD): 7.76–7.66 (m, 6H), 6.74–6.70 (m, 2H), 3.40 (t, *J* = 6.6 Hz, 2H), 2.49 (t, *J* = 7.2 Hz, 2H), 2.02–1.93 (m, 2H). ^{13}C NMR (*δ*, 125 MHz, CD_3OD): 173.41, 153.29, 150.57, 145.75, 141.28, 125.96, 123.78, 121.19, 115.21, 51.95, 34.80, 25.94. HRMS calculated for $C_{16}H_{18}N_7O$: 324.1567 $[M+H]^+$. Found: 324.1570.

***tert*-Butyl (4-nitrosophenyl)carbamate (26).**

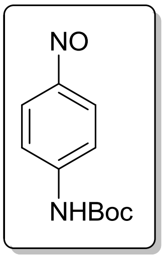

To a flask containing a solution of *tert*-butyl (4-aminophenyl)carbamate (100 mg, 0.48 mmol) in CH_2Cl_2 (4 mL) was added an aqueous solution of oxone (0.169 M, 0.72 mmol). After stirring for 3 h at room temperature, the color of the solution changes from white to brown. The mixture was diluted with CH_2Cl_2 (25 mL), washed first with 1 M HCl solution (3 x 50 mL), then with a saturated solution of $NaHCO_3$ (3 x 50 mL) and brine (3 x 50 mL). The organic layer was dried over Na_2SO_4, filtered and concentrated. The resulting residue was purified by column chromatography (CH_2Cl_2) to afford 50.3 mg (0.23 mmol, 47%) of **26** as a green olive solid. mp: 111–113 ºC. R_f: 0.6 (100% CH_2Cl_2). IR (film): υ = 3280, 2984, 1733, 1718, 1528, 1100 cm^{-1}. ^1H NMR (*δ*, 400 MHz, $CDCl_3$) 7.88 (d, *J* = 8.1 Hz, 2H), 7.58 (d, *J* = 8.8, 2H), 6.87 (s, 1H), 1.54 (s, 9H). ^{13}C NMR (*δ*, 100 MHz, $CDCl_3$): 164.12, 151.92, 145.67, 123.59, 117.37, 82.15, 28.33. HRMS calculated for $C_{11}H_{13}N_2O_3$: 221.0926 $[M–H]^-$. Found: 221.0915.

(*E*)-*tert*-Butyl (4-((4-azidophenyl)diazenyl)phenyl)carbamate (27).

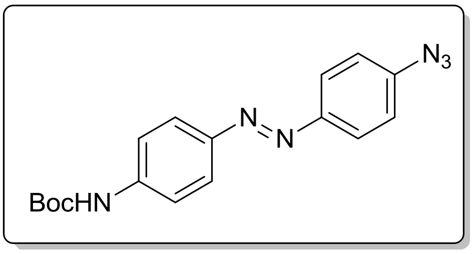

To a solution of **26** (373 mg 1.68 mmol) in 10 mL of AcOH was added 4-azidoaniline **25** (188 mg 1.40 mmol) and the reaction mixture was stirred at room temperature for 5 days. After neutralization of the acidic medium with a saturated solution of $NaHCO_3$ (140 mL), the mixture was diluted with CH_2Cl_2 (100 mL). The aqueous layer was

extracted with CH_2Cl_2 (2 x 100 mL) and the combined organic layers were washed with H_2O (3 x 100 mL) and brine (3 x 100 mL), then dried over $MgSO_4$, filtered and concentrated *in vacuo*. The crude was purified by column chromatography (9:1 Hexane/EtOAc) to afford **27** (430 mg, 91%) as a bright orange solid. mp: 161–164 ºC. R_f: 0.8 (Hexane/EtOAc 4:1). IR (film): υ = 3373, 2978, 2931, 2400, 2249, 2111, 1698, 1603, 1575, 1149 cm^{-1}. ^1H NMR (δ, 400 MHz, $CDCl_3$): 7.93–7.86 (m, 4H), 7.51 (d, J = 8.9 Hz, 2H), 7.16–7.12 (m, 2H), 6.67 (brs, 1H), 1.54 (s, 9H). ^{13}C NMR (δ, 100 MHz, $CDCl_3$): 152.44, 150.03, 148.30, 142.24, 141.24, 124.50, 124.23, 119.68, 118.41, 81.28, 28.45. HRMS calculated for $C_{17}H_{19}N_6O_2$: 339.1569 $[M+H]^+$. Found: 339.1557.

(*E*)-4-((4-Azidophenyl)diazenyl)aniline hydrochloride (22).

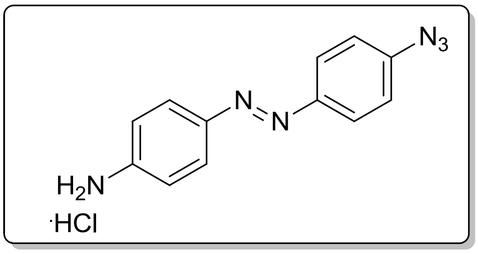

To a flask containing **27** (0.38 g, 1.12 mmol) was added a freshly prepared solution of HCl in EtOAc (20 mL) *(see General procedure for HCl in EtOAc preparation)*. After stirring for 2 h at room temperature, an argon flow was passed through the solution to remove all HCl and the solvent was removed under reduced pressure. The resulting purple solid was triturated with Et_2O (3 × 30 mL) and filtered to give **22** (0.28 g, 1.01 mmol, 90%). mp: >350 ºC. IR (film): υ = 2847, 2547, 2114, 1595, 1556, 1488, 1279 cm^{-1}. ^1H NMR (δ, 400 MHz, DMSO-d_6): 7.89–7.84 (m, 2H), 7.83–7.77 (m, 2H), 7.30–7.25 (m, 2H), 7.10–7.03 (m, 2H). ^{13}C NMR (δ, 100 MHz, DMSO-d_6): 155.62, 149.18, 141.23, 138.19, 124.76, 123.81, 120.01, 118.02. HRMS calculated for $C_{12}H_{11}N_6$: 239.1045 $[M+H]^+$. Found: 239.1019.

Synthesis of azides 1 and 2

(2*S*,4*R*)-1-(*tert*-Butyl)-2-ethyl 4-(4-((4-((*E*)-(4-(4-azidobutanamido) phenyl) diazenyl) phenyl) amino)-4-oxobutyl)-5-oxopyrrolidine-1,2-dicarboxylate (23).

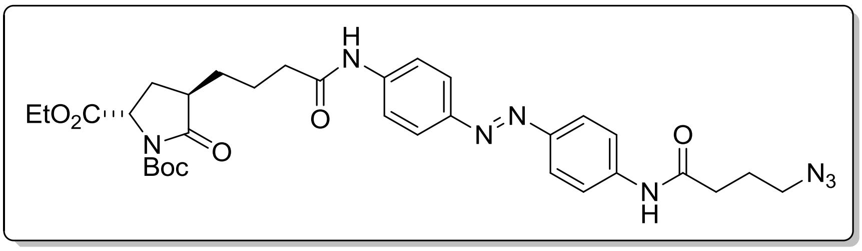

To a solution of pyroglutamate derivative[2, 3] (1.07 g, 3.09 mmol) in EtOAc (40 mL) containing DIPEA (1.10 mL, 6.18 mmol), HATU (2.35 g, 6.18 mmol) was added and the mixture was stirred at room temperature for 15 min. Consequently, a solution of **21** (1.0

g, 3.09 mmol) in EtOAc (10 mL) was added, and the reaction was stirred at 40 ºC for 16 h. The mixture was diluted with EtOAc (100 mL) and washed with H_2O (2 × 150 mL) and brine (2 × 150 mL). The organic layer was dried over $MgSO_4$, filtered and concentrated. The reaction crude was purified by column chromatography (4:1 to 2:1 Hexane/EtOAc gradient) to give **23** (1.56 g, 2.41 mmol, 78%) as an orange solid. mp: 166–169 ºC. R_f: 0.2 (Hexane/EtOAc 2:1). $[\alpha]_D$: n.d. IR (film): υ = 3309, 2981, 2934, 2096, 1788, 1742, 1657, 1590, 1528, 1296, 1244, 844 cm^{-1}. ^1H NMR (δ, 400 MHz, $CDCl_3$): 7.78–7.74 (m, 4H), 7.67–7.48 (m, 4H), 4.50 (dd, *J* = 9.6, 1.5 Hz, 1H), 4.16 (q, *J* = 7.2 Hz, 2H), 3.36 (t, *J* = 6.4 Hz, 2H), 2.64–2.56 (m, 1H), 2.45 (t, *J* = 7.2 Hz, 2H), 2.37 (td, *J* = 7.2, 2.5 Hz, 2H), 2.19 (ddd, *J* = 13.2, 8.5, 1.5 Hz, 1H), 2.00–1.89 (m, 3H), 1.88–1.79 (m, 1H), 1.77–1.69 (m, 3H), 1.41 (s, 9H), 1.22 (t, *J* = 7.2 Hz, 3H). ^{13}C NMR (δ, 100 MHz, $CDCl_3$): 175.97, 171.37, 171.34, 170.53, 149.46, 149.19, 149.02, 140.60, 140.21, 123.92, 123.89, 120.10, 84.02, 77.36, 61.97, 57.41, 50.85, 41.64, 37.26, 34.29, 29.70, 28.46, 28.00, 24.70, 22.79, 14.34. HRMS calculated for $C_{32}H_{40}N_8O_7Na$: 671.2912 $[M+Na]^+$. Found: 671.2939

(2*S*,4*R*)-1-*tert*-Butyl-2-ethyl-4-(4-((4-((*E*)-(4-azidophenyl)diazenyl)phenyl)amino)4-oxbutyl) -5-oxopyrrolidine-1,2-dicarboxylate (24).

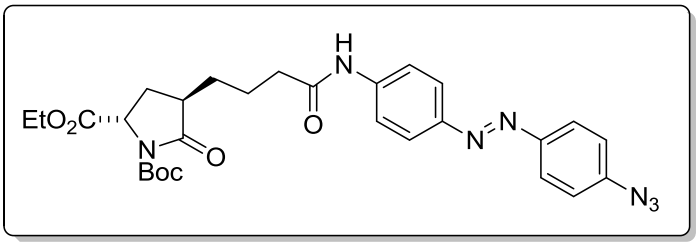

To a solution of pyroglutamate derivative[2, 3] (0.57 g, 1.65 mmol) in EtOAc (20 mL) containing DIPEA (1.16 mL, 6.62 mmol), HATU (1.26 g, 3.31 mmol) was added and the mixture was stirred at room temperature for 15 min. Consequently, a solution of **22** (0.50 g, 1.82 mmol) in EtOAc (10 mL) was added, and the reaction was stirred at 55 ºC for 18 h. The mixture was diluted with EtOAc (250 mL) and washed with $NaHCO_3$ solution (3 × 150 mL) and brine (3 × 150 mL). The organic layer was dried over Na_2SO_4, filtered and concentrated. The reaction crude was purified by column chromatography (CH_2Cl_2/EtOAc 9:1) to give **24** (0.53 g, 0.94 mmol, 57%) as an orange solid. mp: 69–73 ºC. R_f: 0.32 (CH_2Cl_2/EtOAc 4:1). $[\alpha]_D$: +79.94 (*c* = 0.87, $CHCl_3$). IR (film): υ = 3336, 2977, 2933, 2111, 1781, 1741, 1592, 1531, 1297, 1144 cm^{-1}. ^1H NMR (δ, 400 MHz, $CDCl_3$): 8.40–6.89 (m, 8H), 4.60–4.54 (m, 1H), 4.17 (q, *J* = 7.1 Hz, 2H), 2.73–2.60 (m, 1H), 2.52–2.22 (m, 3H), 2.10–1.97 (m, 1H), 1.97–1.87 (m, 1H), 1.87–1.76 (m, 2H), 1.48 (9H, s), 1.42 (1H, s), 1.29 (t, *J* = 7.1 Hz, 3H). ^{13}C NMR (δ, 100 MHz, $CDCl_3$): 175.79, 171.34, 149.98, 149.37, 148.81, 142.29, 141.11, 125.62, 124.52, 123.96, 119.97, 119.65, 61.93, 57.40, 41.68, 37.24, 30.43, 29.79, 28.51, 28.00, 22.83, 14.33. HRMS calculated for $C_{28}H_{32}N_7O_6$: 562.2414 $[M–H]^-$. Found: 562.2429.

General procedure for the ring-opening hydrolysis reaction. Synthesis of 29 as a representative example.

To a solution of **24** (67.0 mg, 0.119 mmol) in THF (1.2 mL) at 0 ºC was added 1.0 M aqueous solution of LiOH (1.2 mL). After stirring for 2 h, the mixture was acidified to pH 2 with 1.0 M HCl solution and partitioned between EtOAc (20 mL) and H_2O (20 mL). The layers were separated and the aqueous phase was extracted with EtOAc (2 x 20 mL). The combined organic layers were dried over Na_2SO_4, filtered and concentrated *in vacuo*. The residue obtained was purified by silica gel chromatography.

(2*R*,4*S*)-2-(4-((4-((*E*)-(4-(4-Azidobutanamido) phenyl) diazenyl) phenyl) amino)-4-oxobutyl)-4-((*tert*-butoxycarbonyl) amino) pentanedioic acid (28).

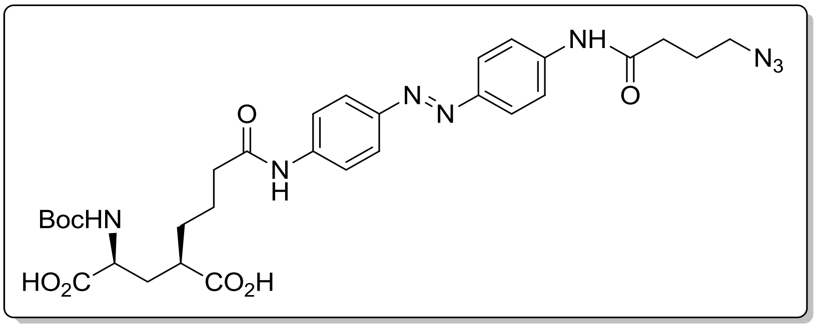

Following the general hydrolysis procedure, 1.2 g (1.87 mmol, 81% yield) of **28** as an orange solid were obtained from 1.5 g (2.31 mmol) of **23**. The compound was purified by flash chromatography (98:2 to 90:10 CH_2Cl_2/MeOH gradient with 1% AcOH). mp: 198–199 ºC. R_f: 0.2 (MeOH/CH_2Cl_2 5:95 + 1 % AcOH). $[\alpha]_D$: +8.6 (*c* = 0.25, MeOH). IR (film): υ = 3303, 2931, 2097, 1656, 1530, 1408, 1367, 1252, 1152, 845 cm^{-1}. ^1H NMR (δ, 400 MHz, CD_3OD): 7.85–7.83 (m, 4H), 7.74–7.72 (m, 4H), 4.21–4.06 (m, 1H), 3.39 (t, *J* = 6.7 Hz, 2H), 2.65–2.54 (m, 1H), 2.50 (t, *J* = 7.3 Hz, 2H), 2.46–2.38 (m, 2H), 2.28–2.14 (m, 1H), 1.98–1.93 (m, 2H), 1.82–1.56 (m, 5H), 1.43 (s, 9H). ^{13}C NMR (δ, 100 MHz, CD_3OD): 178.65, 175.96, 175.20, 174.19, 173.46, 158.11, 150.09, 142.52, 142.49, 124.52, 121.09, 121.06, 80.51, 53.48, 51.91, 43.11, 37.73, 34.80, 33.39, 28.72, 28.52, 25.85, 24.35. HRMS calculated for $C_{30}H_{37}N_8O_8$: 637.2734 $[M–H]^-$. Found: 637.2740.

(2*R*,4*S*)-2-(4-((4-((*E*)-(4-Azidophenyl)diazenyl)phenyl)amino)-4-oxobutyl)-4-((*tert*-butoxycarbonyl)amino)pentanedioic acid (29).

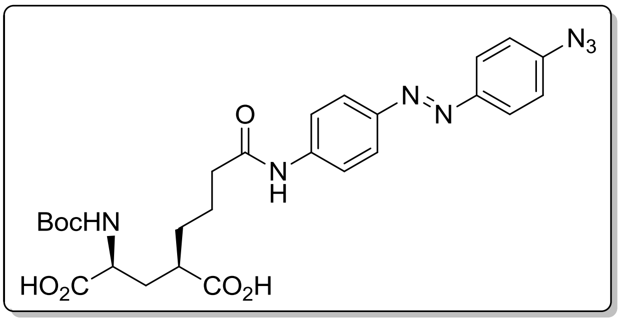

Following the general hydrolysis procedure, 43 mg (0.077 mmol, 65% yield) of **29** as an orange solid were obtained from 67 mg (0.119 mmol) of **24**. The compound was purified by flash chromatography (CH_2Cl_2/MeOH 9:1 with 1% AcOH). mp: 98–99 ºC. R_f: 0.45 (CH_2Cl_2/MeOH 9:1 with 1% AcOH). $[\alpha]_D$: –31.56 (*c* = 1.08, MeOH). IR (film): υ = 3317, 2927, 2407, 2113, 1703, 1595, 1530, 1153 cm^{-1}. ^1H NMR (*δ*, 400 MHz, CD_3OD): 7.78 (d, *J* = 8.5 Hz, 2H), 7.74 (d, *J* = 8.8 Hz, 2H) 7.64 (d, *J* = 8.8 Hz, 2H), 7.07 (d, *J* = 8.5 Hz, 2H), 4.14–3.91 (m, 1H), 2.58–2.40 (m, 1H), 2.39–2.23 (m, 2H), 2.20–2.03 (m, 1H), 1.78–1.45 (m, 5H), 1.34 (s, 9H). ^{13}C NMR (*δ*, 100 MHz, CD_3OD): 178.62, 175.90, 175.18, 174.19, 158.10, 151.15, 149.92, 143.83, 142.82, 125.41, 124.68, 121.04, 120.66, 43.10, 37.77, 34.81, 33.39, 28.73, 24.33, 20.75. HRMS calculated for $C_{26}H_{30}N_7O_7$: 552.2207 $[M–H]^-$. Found: 552.2219.

General procedure for the NH-Boc deprotection reaction. Synthesis of 1 as a representative example.

To a flask containing **28** (1.2 g, 1.87 mmol) was added a freshly prepared solution of HCl in EtOAc (50 mL) *(see General procedure for HCl in EtOAc preparation).* The color of the solution changes from orange to dark purple. After stirring for 2 h at room temperature, an argon flow passed through the solution to remove all HCl and the solvent was removed under reduced pressure. The resulting purple solid was triturated with Et_2O (3 × 30 mL) and filtered.

(2*S*,4*R*)-2-Amino-4-(4-((4-((E)-(4-(4-azidobutanamido)phenyl)diazenyl)phenyl)amino)-4-oxobutyl)pentanedioic acid hydrochloride (1).

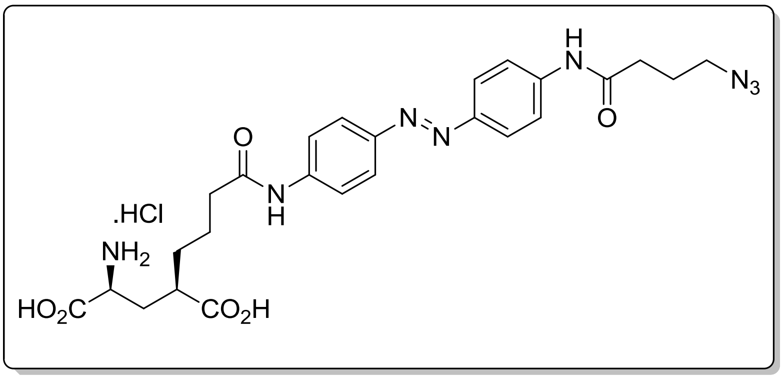

Following the general -NH-Boc deprotection procedure, 0.98 g (1.70 mmol, 91% yield) of **1** were obtained from 1.2 g (1.87 mmol) of **28**. mp: 181–183 ºC. $[\alpha]_D$: +29.9 (*c* = 0.125, MeOH). IR (film): υ = 3320, 2927, 2866, 2095, 1711, 1665, 1590, 1518, 1494, 1240, 1153, 844 cm^{-1}. ^1H NMR (*δ*, 400 MHz, CD_3OD): 7.88–7.84 (m, 4H), 7.79–7.73 (m, 4H), 4.03 (dd, *J* = 8.0, 6.0 Hz, 1H), 3.41 (t, *J* = 6.7 Hz, 2H), 2.76–2.67 (m, 1H), 2.56–2.45 (m, 4H), 2.42–2.33 (m, 1H), 2.01–1.92 (m, 3H), 1.86–1.68 (m, 4H). ^{13}C NMR (δ, 100 MHz, CD_3OD): 176.32, 172.81, 172.21, 170.16, 148.82, 148.79, 141.27, 141.19, 123.18, 119.79, 119.76, 51.18, 50.59, 41.02, 36.16, 33.48, 31.84, 31.27, 24.54, 22.49. HRMS calculated for $C_{25}H_{29}N_8O_6$: 537.2210 $[M-H]^-$. Found: 537.2200.

(2*S*,4*R*)-2-Amino-4-(4-((4-((*E*)-(4-azidophenyl)diazenyl)phenyl)amino)-4-oxobutyl)pentanedioic acid hydrochloride (2).

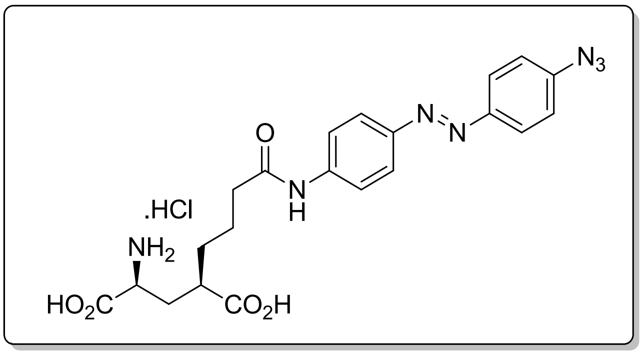

Following the general -NH-Boc deprotection procedure, 19 mg (39.02 µmol, 72% yield) of **2** were obtained from 30 mg (54.20 µmol) of **29**. mp: 188 ºC. $[\alpha]_D$: + 17.24 (*c* = 0.89, MeOH). IR (film): υ =, 2924 , 2111, 1708, 1664, 1533, 1492, 1405, 1281, 1209, 1153 cm^{-1}. ^{1}H NMR (δ, 400 MHz, DMSO-d_6): 7.94–7.81 (m, 6H), 7.38–7.25 (m, 2H), 3.79 (t, *J* = 7.0 Hz, 1H), 2.71–2.61 (m, 1H), 2.19–1.74 (m, 3H), 1.73–1.42 (m, 5H). ^{1}H NMR (δ, 400 MHz, CD_3OD): 7.95–7.91 (m, 2H), 7.90–7.85 (m, 2H), 7.81–7.74 (m, 2H), 7.20–7.25 (m, 2H), 4.02 (dt, *J* = 12.9, 6.5 Hz, 1H), 2.75–2.67 (m, 1H), 2.52–2.44 (m, 2H), 2.38 (ddd, *J* = 14.9, 9.2, 5.9 Hz, 1H), 1.96 (ddd, *J* = 14.5, 8.0, 4.9 Hz, 1H),1.65–1.87 (m, 4H). ^{13}C NMR (δ, 100 MHz, CD_3OD): 177.63, 174.16, 171.49, 151.24, 150.04, 143.99, 142.84, 125.44, 124.69, 121.10, 120.74, 52.53, 42.36, 37.51, 33.19, 32.63, 23.82. HRMS calculated for $C_{21}H_{24}N_7O_5$: 454.1839 $[M+H]^+$. Found: 454.1856.

General procedure for HCl in EtOAc preparation

To prepare a 0.5 L solution of HCl in EtOAc (2 M) for the -NH-Boc deprotection we need a closed addition system, previously dried. On the round bottom flask, acetyl chloride (71.11 mL, 1 eq.) and EtOAc (401.78 mL) was introduced and maintained at 0 ºC with an ice bath. EtOH (58.40 mL, 1 eq.) was introduced in the addition funnel and dropwise addition was kept during 45 min. Once complete, the content could be stored in the fridge for long periods. EtOAc and EtOH were previously dried with 4 Å molecular sieves.

Synthesis of alkynes 4–7

The preparation of the alkynes **4** – **7** is shown in Schemes S3 and S4. Epoxide **4** was obtained by *O*-alkylation of alcohol **30**[5] with (±)-epichlorohydrin (Scheme S3). Alkyne-NHS esters **5** – **7** were obtained by treating acids pent-4-ynoic acid, **33** or **34** with DCC and *N*-hydroxysuccinimide (Scheme S4 step c). Acids **33** and **34** were synthesized by *O*-alkylation of *t*-butyl 3-hydroxypropanoate, followed by deprotection of Boc group (Scheme S4 steps a and b).

Scheme S3. Synthesis of epoxide **4**.[a]

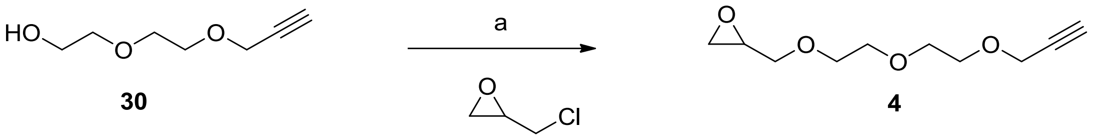

[a]Reagent and conditions: (a) NaOH, TBAHS, rt.

2-(2-(Prop-2-yn-1-yloxy)ethoxy)ethanol (30). Compound **30** was prepared from bis(2-hydroxyethyl)ether according to the procedure reported by Gerland *et al.*[5]

2-((2-(2-(Prop-2-yn-1-yloxy)ethoxy)ethoxy)methyl)oxirane (4).

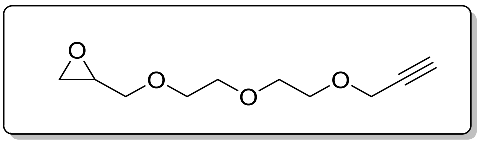

A mixture of 2-(2-(prop-2-yn-1-yloxy)ethoxy)ethanol **30** (370 mg, 2.57 mmol), tetrabutylammonium hydrogensulfate (TBAHS, 87 mg, 0.26 mmol) and 50% NaOH aq solution (2 mL) was vigorously stirred at room temperature and then cooled to 0 ºC. To this mixture was added (±)-epichlorohydrin (474 mg, 5.13 mmol). The resulting suspension was stirred for 18 h at room temperature. The reaction mixture was diluted with H_2O (20 mL) and the aqueous layer was extracted with EtOAc (3 x 20 mL). The combined organic layers were washed with brine, dried over $MgSO_4$, filtered and concentrated. The resulting clear oil was purified by flash chromatography (4:1 to 1:2 hexane/EtOAc gradient) to give **4** (363 mg, 1.81 mmol, 71%). IR (film): υ = 2873, 1351, 1251, 1091, 1032, 911, 840 cm^{-1}. ^{1}H NMR (δ, 400 MHz, $CDCl_3$): 4.20 (d, J = 2.4 Hz, 2H), 3.78 (dd, J = 11.7, 3.0 Hz, 1H), 3.63–3.72 (m, 8H), 3.43 (dd, J = 11.7, 5.9 Hz, 1H), 3.15 (ddt, J = 5.9, 4.2, 2.9 Hz, 1H), 2.78 (t, J = 4.6 Hz, 1H), 2.60 (dd, J = 5.0, 2.7 Hz, 1H), 2.42 (t, J = 2.4 Hz, 1H). ^{13}C NMR (δ, 100 MHz, $CDCl_3$): 79.77, 74.63, 72.11, 70.86, 70.75, 70.58, 69.24, 58.53, 50.94, 44.40. HRMS calculated for $C_{10}H_{16}O_4Na$: 223.0946 $[M+Na]^+$. Found: 223.0936.

Scheme S4. Synthesis of alkyne-NHS esters **5–7**.[a]

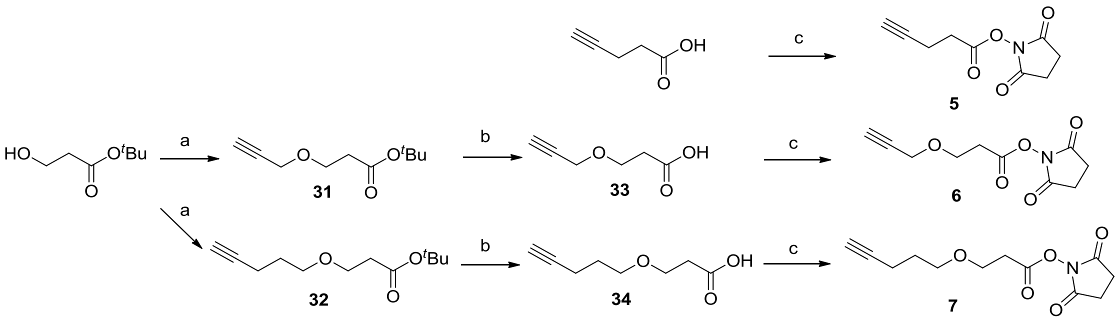

[a]Reagent and conditions: (a) TBAHS, NaOH/toluene, 5-iodopent-1-yne or 3-bromoprop-1-yne, rt, 64–74%; (b) TFA, CH_2Cl_2, rt, 99%; (c) DCC, *N*-hydroxysuccinimide, THF, 61–94%.

General procedure for the alkylation of *t*-butyl 3-hydroxypropanoate. Synthesis of 32 as a representative example.

To a solution of *t*-butyl 3-hydroxypropanoate (0.61 ml, 4.12 mmol) and 5-iodopent-1-yne (0.4 ml, 2.06 mmol) in toluene (5 mL) was added a solution of NaOH (1.48 g, 37.1 mmol) in H_2O (3 mL) and tetrabutylammonium hydrogensulfate (TBAHS, 0.70 g, 2.06 mmol) at room temperature. After stirring for 4 h at room temperature, the solvents were removed under reduced pressure. The resulting residue was dissolved in EtOAc (30 mL) and washed with H_2O (15 mL). The aqueous phase was extracted with EtOAc (3 × 30 mL). The combined organic layers were washed with brine, dried over $MgSO_4$, and evaporated to dryness to give a residue, which was purified by flash chromatography (hexane/EtOAc 20:1) to give the desired products.

***t*-Butyl 3-(prop-2-yn-1-yloxy)propanoate (31).**

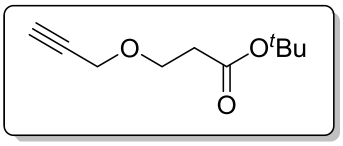

Following the general procedure, 317 mg (1.72 mmol, 64% yield) of **31** were obtained from 0.3 mL (2.71 mmol) of 3-bromoprop-1-yne. IR (film): υ = 2960, 2924, 2855, 1731, 1466, 1368, 1260, 1159 cm^{-1}. ^1H NMR (δ, 400 MHz, $CDCl_3$): 4.13–4.18 (m, 2H), 3.71–3.80 (m, 2H), 2.49–2.55 (m, 2H), 2.42 (t, *J* = 2.4 Hz, 1H), 1.45 (s, 9H). ^{13}C NMR (δ, 100 MHz, $CDCl_3$): 170.78, 80.85, 79.73, 74.56, 65.76, 58.35, 36.22, 28.24. HRMS calculated for $C_{10}H_{16}O_3Na$: 207.0997 $[M+Na]^+$. Found: 207.1010.

***t*-Butyl 3-(pent-4-yn-1-yloxy)propanoate (32).**

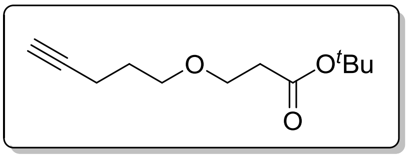

Following the general procedure, 324 mg (1.53 mmol, 74% yield) of **32** were obtained from 0.4 mL (2.06 mmol) of 5-iodopent-1-yne. IR (film): υ = 2955, 2922, 2852, 1732, 1463, 1376, 1274, 1159 cm^{-1}. ^1H NMR (δ, 400 MHz, $CDCl_3$): 3.66 (t, *J* = 6.4 Hz, 2H), 3.52 (t, *J* = 6.1 Hz, 2H), 2.47 (t, *J* = 6.4 Hz, 2H), 2.27 (td, *J* = 7.1, 2.7 Hz, 2H), 1.93 (t, *J* = 2.7 Hz, 1H), 1.73–1.80 (m, 2H), 1.45 (s, 9H). ^{13}C NMR (δ, 100 MHz, $CDCl_3$): 171.10, 84.12, 80.65, 69.33, 68.53, 66.64, 36.52, 28.69, 28.24, 15.31. HRMS calculated for $C_{12}H_{20}O_3Na$: 235.1310 $[M+Na]^+$. Found: 235.1318.

General procedure for the hydrolysis of esters 31–32. Synthesis of 34 as a representative example.

To a stirred solution of *t*-butyl 3-(pent-4-yn-1-yloxy)propanoate **32** (0.32 g, 1.53 mmol) in CH_2Cl_2 (4 mL) was added TFA (1 mL). The resulting mixture was stirred at room temperature for 4 h. Once the reaction was finished, the solvents were removed under reduced pressure. The resulting residue was dissolved in EtOAc (30 mL) and washed with HCl 1 N (20 mL). The aqueous layer was extracted with EtOAc (2 x 20 mL). The combined organic layers were dried over $MgSO_4$, and evaporated to dryness to give a residue, which was used in the next reaction without further purification.

3-(Prop-2-yn-1-yloxy)propanoic acid (33).

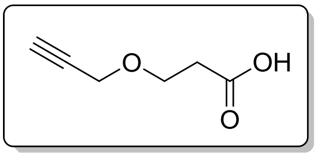

Following the general procedure, 215 mg (1.68 mmol, 99% yield) of **33** were obtained from 310 mg (1.68 mmol) of *t*-butyl 3-(prop-2-yn-1-yloxy)propanoate **31**. IR (film): υ = 3284, 2924, 2854, 1717, 1440, 1190, 1102, 1067 cm^{-1}. ^{1}H NMR (δ, 400 MHz, $CDCl_3$): 4.19 (d, J = 2.4 Hz, 2H), 3.84 (t, J = 6.2 Hz, 2H), 2.70 (t, J = 6.2 Hz, 2H), 2.46 (t, J = 2.4 Hz, 1H). ^{13}C NMR (δ, 100 MHz, $CDCl_3$): 177.44, 79.11, 75.13, 64.97, 58.52, 34.72. HRMS calculated for $C_6H_7O_3$: 127.0395 $[M–H]^-$. Found: 127.0387.

3-(Pent-4-yn-1-yloxy)propanoic acid (34).

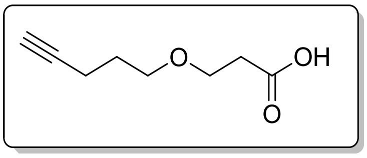

Following the general procedure, 235 mg (1.50 mmol, 99% yield) of **34** were obtained from 324 mg (1.53 mmol) of *t*-butyl 3-(pent-4-yn-1-yloxy)propanoate **32**. IR (film): υ = 3293, 2926, 2874, 1713, 1433, 1186, 1112, 1060 cm^{-1}. ^{1}H NMR (δ, 400 MHz, $CDCl_3$): 3.73 (t, J = 6.2 Hz, 2H), 3.58 (t, J = 6.2 Hz, 2H), 2.64 (t, J = 6.2 Hz, 2H), 2.28 (td, J = 7.0, 2.7 Hz, 2H), 1.95 (t, J = 2.7 Hz, 1H), 1.76–1.80 (m, 2H). ^{13}C NMR (δ, 100 MHz, $CDCl_3$): 175.43, 83.84, 69.64, 68.76, 66.00, 34.77, 28.48, 15.29. HRMS calculated for $C_8H_{11}O_3$: 155.0708 $[M–H]^-$. Found: 155.0681.

General procedure for the synthesis of NHS 5–7. Synthesis of 7 as a representative example.

To a solution of 3-(pent-4-yn-1-yloxy)propanoic acid (0.2 g, 1.28 mmol) in anhydrous THF (5 mL) were added DCC (0.26 g, 1.28 mmol) and *N*-hydroxysuccinimide (0.15 g, 1.28 mmol) at 0 ºC. The suspension was stirred at room temperature for 4 h. Crystalline dicyclohexylurea (DCU) was filtered off and washed with cold, dry THF. The filtrate was concentrated under reduced pressure, dissolved in dry THF (3 mL) and

cooled to 0 ºC for 30 minutes. The insoluble DCU was again filtered off and the process was repeated 2 more times. Filtration and evaporation afforded crude compounds, which were purified as indicated below.

2,5-Dioxopyrrolidin-1-yl pent-4-ynoate (5).

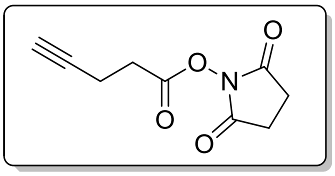

Following the general procedure, 160 mg (0.71 mmol, 61% yield) of **5** were obtained from 150 mg (1.17 mmol) of pent-4-ynoic acid. IR (film): υ = 2929, 2845, 1814, 1783, 1732, 1205, 1068 cm^{-1}. ^{1}H NMR (δ, 400 MHz, $CDCl_3$): 2.79–2.92 (m, 6H), 2.58–2.66 (m, 2H), 2.05 (t, J = 2.7 Hz, 1H). ^{13}C NMR (δ, 100 MHz, $CDCl_3$): 168.84, 166.96, 80.79, 69.99, 30.27, 25.53, 14.06. HRMS calculated for $C_9H_9NO_4Na$: 218.0429 $[M+Na]^+$. Found: 218.0435.

2,5-Dioxopyrrolidin-1-yl 3-(prop-2-yn-1-yloxy)propanoate (6).

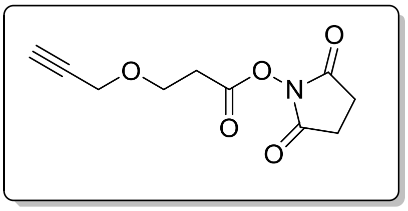

Following the general procedure, 350 mg (1.79 mmol, 88% yield) of **6** were obtained from 0.2 g (2.04 mmol) of 3-(prop-2-yn-1-yloxy)propanoic acid (**33**). The compound was purified by flash chromatography (7:2 to 7:3 hexane/EtOAc gradient). IR (film): υ = 2929, 2873, 1814, 1782, 1729, 1201, 1062 cm^{-1}. ^{1}H NMR (δ, 400 MHz, $CDCl_3$): 4.21 (d, J = 2.4 Hz, 2H), 3.90 (t, J = 6.4 Hz, 2H), 2.93 (t, J = 6.4 Hz, 2H), 2.84 (brs, 4H), 2.46 (t, J = 2.4 Hz, 1H). ^{13}C NMR (δ, 100 MHz, $CDCl_3$): 169.09, 169.07, 166.62, 79.19, 75.09, 64.30, 58.51, 31.99, 25.68. HRMS calculated for $C_{10}H_{11}NO_5Na$: 248.0535 $[M+Na]^+$. Found: 248.0515.

2,5-Dioxopyrrolidin-1-yl 3-(pent-4-yn-1-yloxy)propanoate (7).

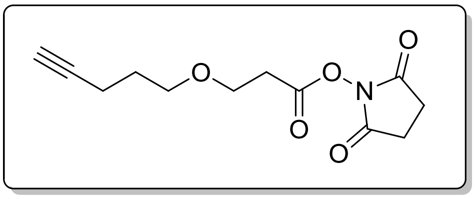

Following the general procedure, 305 mg (1.2 mmol, 94% yield) of **7** were obtained from 0.2 g (1.28 mmol) of 3-(pent-4-yn-1-yloxy)propanoic acid **34**. The compound was

purified by flash chromatography (7:3 to 6:4 hexane/EtOAc gradient). IR (film): υ = 2929, 2873, 1815, 1782, 1736, 1205, 1066 cm^{-1}. ^{1}H NMR (δ, 400 MHz, $CDCl_3$): 3.80 (t, J = 6.4 Hz, 2H), 3.57 (t, J = 6.1 Hz, 2H), 2.88 (t, J = 6.4 Hz, 2H), 2.81–2.87 (m, 4H), 2.29 (td, J = 7.1, 2.6 Hz, 2H), 1.94 (t, J = 2.7 Hz, 1H), 1.75–1.84 (m, 2H). ^{13}C NMR (δ, 100 MHz, $CDCl_3$): 169.06, 166.85, 84.08, 69.60, 68.59, 65.39, 32.29, 28.54, 25.72, 15.22. HRMS calculated for $C_{12}H_{15}NO_5Na$: 276.0848 $[M+Na]^+$. Found: 276.0843.

Supplementary References

1. Zhu, W. & Ma, D. Synthesis of aryl azides and vinyl azides via proline-promoted CuI-catalyzed coupling reactions. *Chem. Commun.* , 888-889 (2004).
2. Volgraf, M. et al. Allosteric control of an ionotropic glutamate receptor with an optical switch. *Nat. Chem. Biol.* **2**, 47-52 (2006).
3. Ezquerra, J. et al. Stereoselective reactions of lithium enolates derived from N-BOC protected pyroglutamic esters. *Tetrahedron* **49**, 8665-8678 (1993).
4. Shao, C. et al. Copper(I) oxide and benzoic acid ‘on water’: a highly practical and efficient catalytic system for copper(I)-catalyzed azide–alkyne cycloaddition. *Tetrahedron Lett.* **52**, 3782-3785 (2011).
5. Gerland, B. et al. Synthesis of a library of fucosylated glycoclusters and determination of their binding toward Pseudomonas aeruginosa lectin B (PA-IIL) using a DNA-based carbohydrate microarray. *Bioconjug. Chem.* **23**, 1534-1547 (2012).
6. Gorostiza, P. et al. Mechanisms of photoswitch conjugation and light activation of an ionotropic glutamate receptor. *Proc Natl Acad Sci U S A* **104**, 10865-10870 (2007).
7. Slijkerman, R.W. et al. The pros and cons of vertebrate animal models for functional and therapeutic research on inherited retinal dystrophies. *Prog Retin Eye Res* (2015).
